# Fruit and vegetable consumption and the risk of hypertension: a systematic review and meta-analysis of prospective studies

**DOI:** 10.1007/s00394-023-03145-5

**Published:** 2023-04-27

**Authors:** Helga Madsen, Abhijit Sen, Dagfinn Aune

**Affiliations:** 1 Department of Public Health and Nursing, Faculty of Medicine and Health Sciences, Norwegian University of Science and Technology, Trondheim, Norway; 2 Center for Oral Health Services and Research (TkMidt), Trondheim, Norway; 3 Department of Epidemiology and Biostatistics, School of Public Health, Imperial College London, St Mary’s Campus, Norfolk Place, London, W2 1PG UK; 4 Department of Nutrition, Oslo New University College, Oslo, Norway; 5 Department of Endocrinology, Morbid Obesity and Preventive Medicine, Oslo University Hospital Ullevål, Oslo, Norway

**Keywords:** Fruits, Vegetables, Hypertension, Prospective studies, Meta-analysis

## Abstract

**Purpose:**

A high fruit and vegetable intake has been associated with reduced risk of hypertension; however, results have been inconsistent and it is unclear whether specific types of fruits and vegetables are particularly beneficial. This systematic review and meta-analysis aimed to summarize the published prospective studies on fruit and vegetable consumption and risk of hypertension.

**Methods:**

Embase and PubMed databases were searched for relevant prospective studies up to 15th May 2022. Random effects models were used to calculate summary relative risks (RRs) and 95% confidence intervals (CIs) for the association between fruit and vegetable intake and risk of hypertension. Strength of evidence was assessed using World Cancer Research Fund (WCRF) criteria.

**Results:**

Eighteen prospective studies (451 291 participants, 145 492 cases) were included. The summary RR (95% CI) of hypertension per 200 g/day was 0.97 (0.95–0.99, *I*^2^ = 68%, *n* = 8) for fruits and vegetables, 0.93 (0.89–0.98, *I*^2^ = 77%, *n* = 10) for fruits, and 1.00 (0.98–1.02, *I*^2^ = 38%, *n* = 10) for vegetables. Reductions in risk were observed up to 800 g/day for fruits and vegetables, and 550 g/day for fruits, and these two associations were considered probably causal using WCRF criteria. Inverse associations were observed for apples or pears, blueberries, raisins or grapes, avocado, broccoli, carrots and lettuce, while positive associations were observed for cantaloupe, Brussels sprouts, cruciferous vegetables, and total and fried potatoes (*n* = 2–5).

**Conclusion:**

A high intake of fruit and vegetables combined, and total fruit was associated with reduced risk of hypertension, while results for fruit and vegetable subtypes were mixed and need further study.

**Supplementary Information:**

The online version contains supplementary material available at 10.1007/s00394-023-03145-5.

## Introduction

Hypertension is a growing public health concern worldwide and the number of people aged 30–79 years with hypertension doubled from 331 million women and 317 million men in 1990 to 626 million women and 652 million men in 2019, respectively [1]. The Global Burden of Disease Study ranked hypertension as the leading risk factor for mortality and disability adjusted life-years (DALYs) in 2015 [2], and elevated blood pressure accounted for a total of 9.4 million deaths globally in 2015 [3]. Hypertension is an established risk factor for a range of cardiovascular diseases including intracerebral and subarachnoid haemorrhage, stable angina, myocardial infarction, abdominal aortic aneurysm, peripheral arterial disease, overall vascular disease mortality and sudden cardiac death [3–6]. Hypertension accounts for two-thirds of strokes and half of coronary heart disease (CHD) cases each year, and can also lead to kidney failure, blindness and cognitive impairment [3, 6–8].

Dietary factors are of importance for the prevention of hypertension and a range of chronic diseases including cardiovascular disease, cancer, and type 2 diabetes, as well as all-cause mortality [9, 10]. A high intake of fruit and vegetables has been associated with reduced risk of hypertension in several [11–21], although not all [22–27], cohort studies, and some randomized trials have suggested a beneficial effect of fruit and vegetable consumption on blood pressure [28–30]. Fruit and vegetables are high in potassium which is known to reduce blood pressure [31], and in addition, several studies have found that fruit and vegetables can reduce the risk of obesity and weight gain, two very strong risk factors for hypertension [32–34].

However, the results have not been entirely consistent. Of studies that assessed the association between fruit and vegetables combined and risk of hypertension, half of the studies reported clear inverse associations [12, 14, 15, 20], while the remaining studies found no clear association [22–25]. Studies on fruit [11, 13, 14, 17, 19, 21, 24–28] have been more consistent in showing an inverse association than studies on vegetables [11, 13, 14, 17, 19, 21, 24, 25, 27, 28]. Some studies may have had limited statistical power to detect a clear association because of small sample size or short follow-up [12, 24, 28]. Whether specific types of fruits and vegetables are particularly beneficial in reducing risk of hypertension is also unclear. An analysis of three cohort studies found inverse associations between broccoli, carrots, raisins or grapes, and apples or pears and hypertension risk, while a positive association was observed for Brussels sprouts and cantaloupe [14], while another cohort found inverse associations with apples, oranges, raisins, and dark-yellow vegetables, but slight positive associations with cruciferous vegetables [25]. Some studies reported positive associations between potato consumption and hypertension [35, 36]; however, not all studies were consistent [37]. Although a previous meta-analysis suggested a beneficial impact of fruit and vegetable intake on risk of hypertension [38], additional studies have since been published [15, 17–23, 26], and subtypes of fruits and vegetables were not investigated [38]. We, therefore, conducted an updated meta-analysis of the association between fruit and vegetable intake and the risk of hypertension with the aim of clarifying the strength and shape of the dose–response relationship as well as the associations with specific subtypes of fruits and vegetables.

## Methods

This review was reported in accordance with the PRISMA (Preferred Reporting Items for Systematic reviews and Meta-Analyses) statement [39]. Both the methodology and the criteria were predefined in a protocol before the initiation of the screening phase, but the protocol has not been registered in a public registry.

### Search strategy

PubMed and Embase databases were searched for relevant studies from inception up to 15th May 2022 on fruit and vegetable intake and hypertension. The search terms used for the PubMed and Embase searches are shown in the Supplementary Text. We also searched the reference lists of previous meta-analyses [38, 40–42] as well as the included studies for any potentially missed additional studies.

### Study selection and inclusion criteria

Reference Manager version 11 was used for the literature screening. Titles and abstracts of each record were screened initially, and full texts of articles that were considered potentially relevant were obtained for a final decision on whether the study should be included. Prospective and retrospective cohort studies, nested case–control studies within cohorts and case-cohort studies which reported adjusted relative risk estimates (risk ratios, hazard ratios, incidence rate ratios, odds ratios) for the association between fruit and vegetable intake and risk of incident hypertension in generally healthy adults without hypertension at baseline were included. Retrospective case–control studies were excluded because of the potential for recall and selection biases and cross-sectional studies were excluded because of the lack of temporality between the exposure and the outcome. In addition, studies reporting unadjusted risk estimates, studies without data on fruit and vegetable consumption or hypertension or elevated blood pressure, abstract only studies, and studies reporting on blood pressure as a continuous measure, and duplicates were excluded and a list of the excluded studies and exclusion reasons is provided in Supplementary Table 1. When multiple articles were available from the same study, we used the publication with the most detailed data on fruit and vegetables and hypertension. The first part of the screening (all 17566 records) was done by HM, and the second part of the screening (366 selected records) were done in duplicate by DA and HM. Disagreements were resolved through discussion.

### Data extraction

Relevant data was extracted from each study including: name of the first author, publication year, geographic location, name of the study, recruitment and follow-up period, sample size, age, sex, number of cases, dietary assessment method, exposure (fruit and vegetables, fruit, vegetables, or subtypes), quantity or frequency of intake, relative risks (95% confidence intervals), and confounders adjusted for in the analysis. Extracted data are shown in Supplementary Table 2. The data extraction was done by HM and was checked for accuracy by DA.

### Quality assessment of included studies

A modified version of the Newcastle–Ottawa scale for cohort studies was used to assess the quality of the included observational studies [43]. The modified scale gave a total score from 0 to 8 points, and we considered a total score of 0–3, > 3–6 and > 6–8 indicating low, medium and high study quality, respectively. The score was modified by (1) deleting the point about representability, which is not really relevant for study quality, (2) giving 0.25 points per confounder that was adjusted for, up to a maximum of 2 points, rather than 1 point for each of two confounders, as studies that only adjusted for age and sex could still be given the maximum score in spite of being prone to confounding, in the original scale, and (3) by refining the scoring for the outcome assessment so that studies only using registry linkage scored 0.5 point and those with measured blood pressure at two or more time points or record linkage plus independent assessment/validated assessment scored 1 point. The study quality assessment is displayed in Supplementary Table 3.

### Assessment of strength of evidence

We used World Cancer Research Fund criteria for evaluating the strength of the evidence [44]. This grading system takes into account a range of factors including evidence from different study types, the number of studies available, heterogeneity, quality of the studies, dose–response relationship, and biological plausibility and experimental evidence. Evidence grades include (1) substantial effect on risk unlikely, (2) limited-no conclusion, (3) limited-suggestive, (4) probable and (5) convincing evidence of a causal relationship. Detailed descriptions of the criteria are found in Supplementary Table 4.

### Outcome definition

Diagnosis of hypertension was defined in most studies as SBP ≥140 mm Hg and/or DBP ≥ 90 mm Hg, or SBP ≥ 135 mm Hg and/or DBP ≥85 mm Hg, by a medical diagnosis of hypertension or if the subject received antihypertensive drug therapy. Elevated blood pressure was defined as SBP ≥130 mm Hg, and/or DBP ≥85 mm Hg [11].

### Statistical methods

The random effects model by DerSimonian and Laird, which take into account heterogeneity within and between studies, was used to calculate summary relative risks (RRs) and 95% confidence intervals (CIs) for the association between fruit and vegetable intake and hypertension [45]. The method of Greenland and Longnecker [46] was used for the linear dose–response analysis to estimate study specific slopes (linear trends) and 95% CIs from the natural logarithm of the RRs across categories of fruit and vegetable intake. For studies reporting means or medians of intake per category these estimates were used directly. If ranges of intake were reported, the width of the adjacent category was used to estimate a lower or upper cut-off point for open-ended categories. For studies reporting intakes in servings, the intakes were converted to grams using a serving size of 80 grams for fruits and vegetables combined [9], and separately, and for specific types of fruits and vegetables serving sizes reported in a pooled analysis were used [47]. Fruit and vegetable subtypes were grouped according to culinary definition, not botanical definition. Nonlinear dose–response analyses were conducted to examine the shape of dose–response relationship between fruit and vegetable intake and incidence of hypertension. The nonlinear dose–response analyses were conducted using restricted cubic splines with three knots at 10%, 50%, and 90% centiles of the distribution which were combined using multivariable meta-analysis [48].

Heterogeneity between studies was evaluated with *Q*- and *I*-squared (*I*^2^) statistics [49]. Subgroup and meta-regression analyses were conducted to investigate possible sources of heterogeneity including sex (men vs. women), duration of follow-up (≥ 10 vs. < 10 years follow-up), geographic location (Europe, America, Asia), number of cases (< 500, 500-< 1000, ≥ 1000 cases), blood pressure cut-off values for defining hypertension (≥ 140/≥ 90 mmHg vs. ≥ 135 or ≥ 130/≥85 mmHg), study quality (0–3, >3–6, >6–8 stars), and adjustment for confounding factors (age, education, family history of hypertension, smoking, alcohol, BMI, physical activity, intakes of sodium, sugar-sweetened beverages, meat, fish, whole grains, dairy products, and energy). Sensitivity analyses were conducted to investigate the robustness of the findings by excluding one study at a time from the meta-analysis to test whether the results were driven by one very large study or by outliers. *E*-values were calculated to estimate the strength of an unadjusted confounder that could explain away the observed associations [50]. The *E*-value is defined as the minimum strength an unmeasured or uncontrolled confounder would have with both the exposure and the outcome to fully explain away the observed associations. Publication bias was assessed with Egger's test [51], Begg’s test and by inspection of funnel plots [52]. All statistical analyses were conducted using STATA version 17.0 (StataCorp, College Station, TX, USA).

### Ethical considerations

The data extracted and analysed were from already published and ethically approved studies, thus ethical approval was therefore not needed for this review.

## Results

**Fig. 1:**
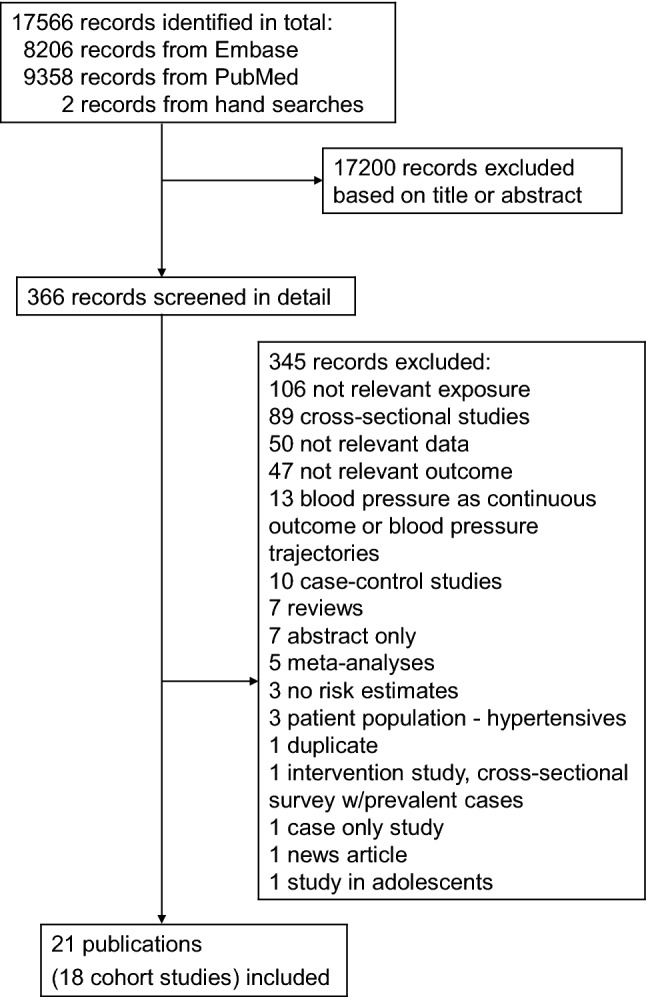
Flowchart of study selection

Out of the total 17566 records screened, 366 articles were examined and evaluated in full text. Out of these 366 articles, 345 articles were excluded for not meeting the inclusion criteria (Supplementary Table 1). Eighteen cohort studies (21 publications) met the pre-specified inclusion criteria and were included in the analysis of fruit and vegetable intake and risk of incident hypertension or elevated blood pressure [11–28, 35–37] (Fig. 1, Supplementary Table 2). The characteristics extracted from the included studies are presented in Supplementary Table 2. The total number of participants across all studies were 451 291 and there were 145 492 hypertension cases. The duration of follow-up ranged from 3 to 28 years. The age of the participants ranged from 18 to 95 years. There were four studies including only women [14, 25, 26, 36], one study (two publications) including only men [14, 36], 13 studies (17 publications) including both men and women [11–13, 15–24, 27, 28, 35, 37]. Seven studies (six publications) were from the USA [11, 14, 25–27, 36], six studies (9 publications) were from Asia [13, 16, 18–22, 28, 35], four studies (five publications) were from Europe [12, 15, 17, 24, 37], and one was from Australia [23]. Supplementary Figs. 1–78 show the high versus low, linear and nonlinear dose–response analyses of subtypes of fruits and vegetables and risk of hypertension, while Supplementary Figs. 79–81 show the funnel plots and Supplementary Figs. 82-84 show the influence analyses for fruits and vegetables combined and separately.

### Study quality assessment

The included studies had an average score of 7.0 points out of a maximum total of 8 points, with the lowest score of 6 and the highest score of 8 (see Supplementary Table 3). This indicates moderately high methodological quality of the studies. The main quality issues were self-report of hypertension diagnoses and limited reporting on adequacy of follow-up.

### Fruit and vegetable intake and risk of incident hypertension

**Fig. 2:**
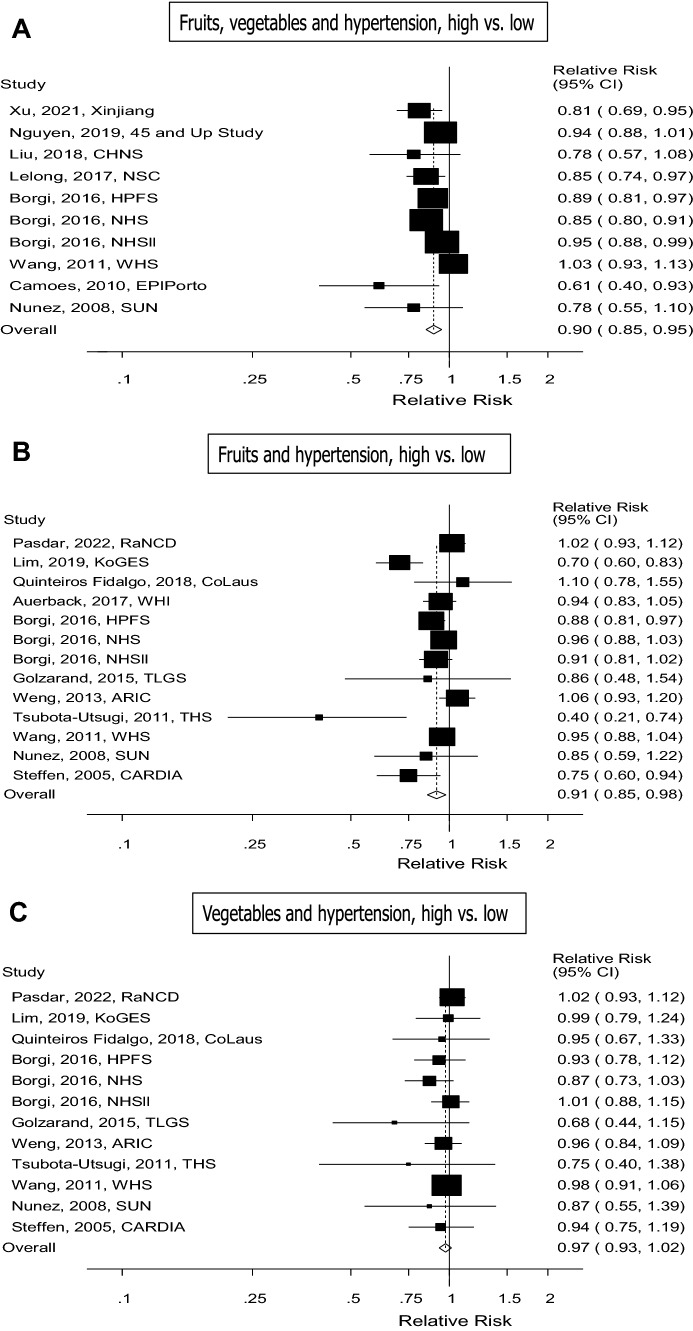
Fruits, vegetables and hypertension, high vs. low intake

**Fig. 3:**
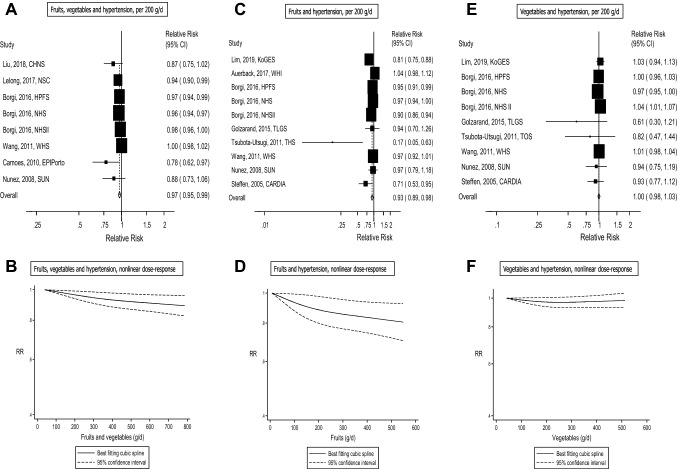
Fruits, vegetables and hypertension, dose–response analysis per 200 g/d, and nonlinear dose–response analysis

Ten cohort studies (eight publications) with 102 395 cases among 346 613 participants were included in the analysis of high vs. low intake of fruits and vegetables and risk of incident hypertension [12, 14, 15, 20, 22–25]. Of the studies, four were from America, three were from Europe, two were from Asia and one was from Australia. The summary RR for high vs. low intake was 0.90 (95% CI 0.85–0.95, *I*^2^= 58.7%, p_heterogeneity_=0.01) (see Fig. 2a). Eight cohort studies (six publications) with 94 871 cases among 308 893 participants were included in the linear dose–response analysis of fruit and vegetable intake and risk of incident hypertension [12, 14, 15, 22, 24, 25]. The summary RR per 200 g/day of fruits and vegetables was 0.97 (95% CI 0.95–0.99, *I*^2^=68.1% and p_heterogeneity_=0.004) (see Fig. 3a). In the sensitivity analyses, the summary RR ranged from 0.96 (95% CI 0.94–0.98) when excluding the study by Wang et al. [25] to 0.97 (95% CI 0.95–0.99) when excluding the Nurses’ Health Study (NHS) by Borgi et al. [14] (Supplementary Fig. 82). There was no indication of publication bias with Egger’s test (*p* = 0.13) or with Begg’s test (*p* = 0.54), although there was some asymmetry in the funnel plot (Supplementary Fig. 79). There was no evidence of a nonlinear association for fruits and vegetables, *P*_*nonlinearity*_ = 0.23, and there was an 11% reduction in risk at an intake of 800 g/day compared to 40 g/day (Fig. 3b, Supplementary Table 5).

### Fruit intake and risk of incident hypertension

Thirteen cohort studies (11 publications) with 134 798 cases among 329 029 participants were included in the analysis of high vs. low intake of fruit and risk of incident hypertension or elevated blood pressure [11, 13, 14, 17, 19, 21, 24–28]. Of the studies, seven were from America, two were from Europe and four were from Asia. The summary RR for high vs. low intake was 0.91 (95% CI 0.85–0.97, *I*^2^= 62.6%, p_heterogeneity_=0.001) (see Fig. 2b). Ten cohort studies (eight publications) with 131 281 cases and 315 742 participants were included in the linear dose-response analysis of fruit intake and hypertension risk [11, 13, 14, 19, 24–26, 28]. The summary RR per 200 g/day of fruit was 0.93 (95% CI 0.89–0.98, *I*^2^ = 77.3 % and p_heterogeneity_=<0.0001) (see Fig. 3c). In the sensitivity analysis, the summary RR ranged from 0.92 (95% CI 0.87–0.96) when excluding the study by Auerback et al. (27) to 0.95 (95% CI 0.91–1.00) when excluding the study by Lim et al. (22) (Supplementary Fig. 83). There was no evidence of publication bias with Egger’s test (*p*=0.20), Begg’s test (*p* = 0.37), although slight asymmetry was observed in the funnel plot (Supplementary Fig. 80) due to one outlying study [28], however, exclusion of this study did not substantially impact the summary estimate (Supplementary Fig. 83). There was no evidence of a nonlinear association for fruits, *P*_*nonlinearity*_ = 0.23, but there was a 19% reduction in risk at an intake of 550 g/day compared to 8 g/day (Fig. 3d, Supplementary table 5).

### Vegetables and risk of incident hypertension

Twelve cohort studies (ten publications) with 98 484 cases among 248 490 participants were included in the analysis of vegetable intake and risk of incident hypertension or elevated blood pressure [11, 13, 14, 17, 19, 21, 24, 25, 27, 28]. Of the studies, six were from America, two were from Europe and four were from Asia. The summary RR for high vs. low intake was 0.97 (95% CI 0.93–1.02, *I*^2^ = 0.0%, P_heterogeneity_ =0.89) (see Fig. 2c). Nine cohort studies (seven publications) with 94 967 cases and 235 203 participants were included in the linear dose-response analysis of vegetable intake and hypertension risk [11, 13, 14, 19, 24, 25, 28]. The summary RR per 200 g/day of vegetables was 1.00 (95% CI 0.98–1.02, *I*^2^ = 45.0 %, p_heterogeneity_ = 0.07) (see Fig. 3e). The summary RR ranged from 0.99 (95% CI 0.97–1.01) when the Nurses’ Health Study ll by Borgi et al. [14] was excluded to 1.01 (95% CI: 1.00-1.03) when the Nurses’ Health Study by Borgi et al. [14] was excluded (see supplementary Fig. 84). There was no clear evidence of publication bias with Egger’s test (*p*=0.40), Begg’s test (*p*=0.25) or by inspection of the funnel plots (see supplementary Fig. 81). There was no evidence of a nonlinear association for vegetables, *P*_*nonlinearity*_ = 0.09 (Fig. 3f, Supplementary Table 5).

### Subtypes of fruit and risk of incident hypertension

**Fig. 4:**
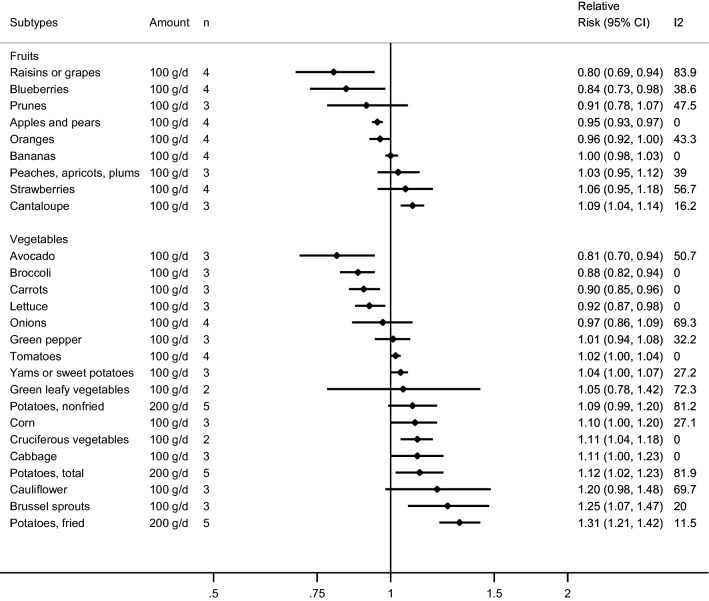
Fruit and vegetable subtypes and hypertension, linear dose–response analysis per 100 g/d

Some studies investigated the association between subtypes of fruit and hypertension (four studies, two publications) [14, 25] (Fig. 4, Table 1, Supplementary Tables 6–8, Supplementary Figs. 1–27). Among subtypes of fruit and risk of hypertension, inverse associations were observed in the dose–response meta-analyses for “apples or pears”, blueberries, and “raisins or grapes”, and positive associations were observed for cantaloupe. The summary RR per 100 g/day was 0.95 (95% CI 0.93–0.98, *I*^2^ = 0%, P_heterogeneity_ = 0.99) for “apples or pears”, 0.84 (95% CI 0.73–0.98, *I*^2^ = 38.6%, P_heterogeneity_ = 0.18) for blueberries, 0.80 (95% CI 0.69–0.94, *I*^2^ = 83.9%, P_heterogeneity_<0.0001) for “raisins or grapes”, and 1.09 (95% CI 1.04–1.14, *I*^2^ = 16.2%, P_heterogeneity_ = 0.30) for cantaloupe (Fig. 4, Table 3). No associations were observed for bananas, oranges, “peaches, apricots or plums”, prunes and strawberries in the dose–response analyses (Fig. 4, Table 3). The summary RR for high vs. low intake was 0.92 (95% CI 0.89–0.95, *I*^2^ = 0%, P_heterogeneity_ = 0.87) for apples and pears and 0.91 (95% CI 0.85–0.98, *I*^2^ = 75.3%, P_heterogeneity_ = 0.007) for “raisins or grapes”. There was no evidence of nonlinearity for the associations between “apples or pears”, bananas, blueberries, oranges, “peaches, apricots, plums”, prunes, “raisins or grapes”, and strawberries and hypertension, but a suggestive nonlinear weak positive association was observed for cantaloupe, *P*_*nonlinearity*_ = 0.05 (Supplementary Tables 6–8).

**Table 1:** Summary relative risks for subtypes of fruits and vegetables and hypertension, high vs. low and dose–response analyses

	High vs. low analysis						Dose–response analysis						
Subtype	*n*	RR (95% CI)	I^2^	P_h_	Egger	References	*n*	Increment	RR (95% CI)	I^2^	P_h_	Egger	References
Apples and pears	4	0.92 (0.89–0.95)	0	0.87	0.61	[14, 25]	4	Per 100 g/d	0.95 (0.93–0.97)	0	0.99	0.88	[14, 25]
Bananas	4	1.00 (0.96–1.03)	31.1	0.23	0.62	[14, 25]	4	Per 100 g/d	1.00 (0.98–1.03)	0	0.45	0.17	[14, 25]
Blueberries	4	0.98 (0.92–1.06)	16.5	0.31	0.30	[14;25]	4	Per 100 g/d	0.84 (0.73–0.98)	38.6	0.18	0.12	[14, 25]
Cantaloupe	3	1.06 (0.97–1.15)	50.2	0.13	0.26	[14]	3	Per 100 g/d	1.09 (1.04–1.14)	16.2	0.30	0.39	[14]
Oranges	4	0.94 (0.89–1.00)	66.1	0.03	0.02	[14, 25]	4	Per 100 g/d	0.96 (0.92–1.00)	43.3	0.15	0.20	[14, 25]
Peaches, apricots or plums	3	1.01 (0.95–1.07)	19.4	0.29	0.74	[14]	3	Per 100 g/d	1.03 (0.95–1.12)	39.0	0.19	0.60	[14]
Prunes	3	0.98 (0.88–1.08)	40.7	0.19	0.71	[14]	3	Per 100 g/d	0.91 (0.78–1.07)	47.5	0.15	0.48	[14]
Raisins or grapes	4	0.91 (0.85–0.98)	75.3	0.007	0.56	[14, 25]	4	Per 100 g/d	0.80 (0.69–0.94)	83.9	< 0.0001	0.50	[14, 25]
Strawberries	4	1.02 (0.98–1.07)	0	0.54	0.26	[14, 25]	4	Per 100 g/d	1.06 (0.95–1.18)	56.7	0.07	0.53	[14, 25]
Avocado	3	0.94 (0.77–1.14)	0	0.63	0.60	[14]	3	Per 100 g/d	0.81 (0.70–0.94)	50.7	0.13	0.40	[14]
Broccoli	3	0.94 (0.90–0.99)	50.3	0.13	0.01	[14]	3	Per 100 g/d	0.88 (0.82–0.94)	0	0.68	0.17	[14]
Brussels sprouts	3	0.99 (0.95–1.04)	50.3	0.13	0.01	[14]	3	Per 100 g/d	1.25 (1.07–1.47)	20.0	0.29	0.28	[14]
Cabbage	3	1.06 (0.98–1.15)	0	0.95	0.86	[14]	3	Per 100 g/d	1.11 (1.00–1.23)	0	0.95	0.52	[14]
Carrots	3	0.95 (0.91–1.00)	0	0.80	0.61	[14]	3	Per 100 g/d	0.90 (0.85–0.96)	0	0.98	0.79	[14]
Cauliflower	3	1.06 (0.99–1.14)	0	0.66	0.29	[14]	3	Per 100 g/d	1.20 (0.98–1.48)	69.7	0.04	0.21	[14]
Corn	3	1.05 (0.93–1.17)	64.5	0.06	0.70	[14]	3	Per 100 g/d	1.10 (1.00–1.20)	27.1	0.25	0.77	[14]
Cruciferous vegetables	2	0.94 (0.56–1.57)	71.5	0.06	NC	[14, 18]	2	Per 100 g/d	1.11 (1.04–1.18)	0	0.85	NC	[14, 18]
Green leafy vegetables	2	0.94 (0.88–1.01)	0	0.42	NC	[18, 25]	2	Per 100 g/d	1.05 (0.78–1.42)	72.3	0.06	NC	[18, 25]
Green pepper	3	1.00 (0.96–1.04)	0	0.47	0.15	[14]	3	Per 100 g/d	1.01 (0.94–1.08)	32.2	0.23	0.14	[14]
Lettuce	3	0.95 (0.84–1.07)	59.8	0.08	0.21	[14]	3	Per 100 g/d	0.92 (0.87–0.98)	0	0.46	0.85	[14]
Onions	4	0.99 (0.92–1.06)	61.1	0.05	0.34	[14, 25]	4	Per 100 g/d	0.97 (0.86–1.09)	69.3	0.02	0.25	[14, 25]
Potatoes total	5	1.10 (0.96–1.25)	61.7	0.03	0.99	[35–37]	5	Per 200 g/d	1.12 (1.02–1.23)	81.9	0.00	0.96	[35–37]
Potatoes fried	5	1.10 (1.02–1.19)	20.2	0.29	0.48	[35–37]	5	Per 200 g/d	1.31 (1.21–1.42)	11.5	0.34	0.13	[35–37]
Potatoes non-fried	5	1.25 (1.11–1.41)	79.8	0.002	0.34	[35–37]	5	Per 200 g/d	1.09 (0.99–1.20)	81.2	0.00	0.68	[35–37]
Tomatoes	4	1.01 (0.97–1.05)	0	0.57	0.67	[14, 25]	4	Per 100 g/d	1.02 (1.00–1.04)	0	0.74	0.44	[14, 25]
Yams or sweet potatoes	4	1.05 (0.96–1.14)	46.7	0.13	0.70	[14, 35]	3	Per 100 g/d	1.04 (1.00–1.07)	27.2	0.25	0.77	[14]

*n* = number of studies

*P*_*h*_ = *P* value for heterogeneity

### Subtypes of vegetables and risk of incident hypertension

Among subtypes of vegetables and risk of hypertension, inverse associations were observed in the meta-analyses of intakes of avocado, broccoli, carrots, and lettuce and hypertension, while positive associations were observed between intakes of Brussels sprouts, cabbage, corn, potatoes (fried), potatoes (total) and “yams or sweet potatoes” and hypertension [14, 18, 35–37] (Fig. 4, Table 1, Supplementary Tables 9–12, Supplementary Figs. 28–78). The summary RRs per 100 g/day was 0.81 (95% CI 0.70–0.94, *I*^2^ = 50.7%, P_heterogeneity_ = 0.13) for avocado, 0.88 (95% CI: 0.82-0.94, I^2^ = 0%, P_heterogeneity_ = 0.68) for broccoli, 0.90 (95% CI 0.85–0.96, *I*^2^ = 0%, P_heterogeneity_ = 0.98) for carrots, 0.92 (95% CI 0.87–0.98, *I*^2^ = 0%, P_heterogeneity_ = 0.46) for lettuce, 1.25 (95% CI 1.07–1.47, *I*^2^ = 20.0%, P_heterogeneity_ = 0.29) for Brussels sprouts, 1.11 (95% CI 1.04–1.18, *I*^2^ = 0%, P_heterogeneity_ = 0.85) for cruciferous vegetables, 1.11 (95% CI 1.00–1.23, *I*^2^ = 0%, P_heterogeneity_ = 0.95) for cabbage, 1.10 (95% CI 1.00–1.20, *I*^2^ = 27.1%, P_heterogeneity_ = 0.25) for corn, 1.04 (95% CI 1.00–1.07, *I*^2^ = 27.2%, P_heterogeneity_ = 0.25) for yams or sweet potatoes, and per 200 g/day was 1.12 (95% CI 1.02-1.23, *I*^2^ = 81.9%, P_heterogeneity_ = 0) for potatoes (total), and 1.31 (95% CI 1.21–1.42, *I*^2^ = 11.5%, P_heterogeneity_ = 0.34) for potatoes (fried) (Fig. 4, Table 1). Results from the high vs. low analyses are displayed in Table 1. A nonlinear positive association was observed for potatoes (fried), *P*_*nonlinearity*_ = 0.02, with a 13% increased risk at an intake of 80-100 compared to 1.2 g/day and similar results were observed for potatoes (total) (Supplementary Table 12). There was no evidence of nonlinearity in the analyses of the remaining vegetable subtypes and hypertension (Supplementary Tables 9–12).

### Subgroup and sensitivity analyses

The results were in general consistent across the various subgroup analyses stratified by duration of follow-up, sex, geographic location, number of cases, blood pressure cut-off values for defining hypertension, study quality, and adjustment for confounding factors (age, education, family history of hypertension, smoking, alcohol, BMI, physical activity, sodium, sugar-sweetened beverages, meat, fish, whole grains, dairy, energy intake). There was no between subgroup heterogeneity when using meta-regression analyses, with the exception of the subgroup analysis of fruit and vegetables and hypertension stratified by number of cases, where studies with a lower number of cases showed stronger associations than those with a larger number of cases, and for the subgroup analysis of fruits and hypertension stratified by cut-off values for hypertension, where a stronger association was observed among the studies that used lower cut-off values than among the studies using a higher cut-off value (Supplementary Table 13).

The estimated *E*-values for the highest level of intake based on the nonlinear dose–response analyses were 1.49 (lower CI 1.24) for fruits and vegetables combined (800 vs. 40 g/d) and 1.76 (lower CI 1.40) for total fruits (550 vs. 8 g/d).

### Evidence grading

Using World Cancer Research Fund criteria for evaluating evidence (42) (Supplementary Table 4), we considered the overall evidence to be supportive of a probably causal relationship between fruit and vegetable intake combined and total fruit and the risk of hypertension, and limited-no conclusion for vegetables (Supplementary Tables 14–15). A justification for these judgements is found in Supplementary Table 14 and included clear inverse associations for fruits and vegetables combined and for fruits in high vs. low, linear and nonlinear dose–response analyses, moderate heterogeneity driven mainly by differences in the size of the effect estimates, and results were in general robust in subgroup and sensitivity analyses. In addition, biologically plausible mechanisms exist and there is data from randomized controlled trials that fruits and vegetables and certain nutrients found in fruits and vegetables can reduce blood pressure. These data are also consistent with randomized trials on dietary patterns high in fruit and vegetables, such as the DASH-diet, Mediterranean diet, and vegetarian and vegan diets which have been shown to reduce blood pressure. Subtypes of fruits and vegetables were in the category of limited-suggestive and limited-no conclusion, mainly because of the few studies available.

## Discussion

The findings from this meta-analysis of 18 cohort studies suggest that high vs. low intakes of fruit and vegetables are associated with a 9–11% reduced risk of hypertension. Inverse associations were observed both for intake of fruit and vegetables combined and for total fruit in the dose–response- and high vs. low analyses, whereas the results for vegetables were null. There was no evidence of nonlinearity, but there was an 11% reduction in risk at an intake of 800 g/day compared to 40 g/day for fruit and vegetables combined, and a 19% reduction in risk at an intake of 550 g/day compared to 8 g/day for fruit. Several subtypes of fruits were inversely associated with risk of hypertension in the dose–response analyses including “apples or pears”, blueberries, and “raisins or grapes”, while a positive association was observed for cantaloupe. Among subtypes of vegetables, inverse associations were observed in the dose–response analyses for avocado, broccoli, carrots, lettuce, and positive associations were observed for Brussels sprouts, cruciferous vegetables, fried potatoes, total potatoes and hypertension. Nonlinear positive associations were observed for cantaloupe and fried potatoes.

### Comparison with previous meta-analysis

The findings from this meta-analysis are consistent with the results from two previous meta-analyses that also found inverse associations between fruit and vegetable consumption combined and risk of hypertension [38, 40]. Compared to the meta-analysis conducted by Wu and colleagues in 2016 [38], which included 9 cohort studies (6 for fruit and vegetables combined, 8 for total fruits and total vegetables separately), the present meta-analysis included 18 cohort studies (10 for fruit and vegetables combined, 13 for total fruits and 12 for total vegetables). The meta-analysis by Li and colleagues [40] included only three cohort studies and was mainly based on cross-sectional studies and case–control studies, study designs which have limitations because of a lack of temporal relation between the exposure and the outcome and potential recall and selection biases. In contrast to the previous meta-analyses, the current meta-analysis also included analyses of specific types of fruits and vegetables, which could be important with regard to dietary recommendations. The results of this meta-analysis are also consistent with a meta-analysis of two randomized controlled trials, which found reduced systolic and diastolic blood pressure with increased fruit and vegetable intake [53], the Dietary Approaches to Stop Hypertension (DASH) trials, PREDIMED trials and trials of vegetarians and vegans which have shown that dietary patterns rich in fruit and vegetables reduced blood pressure [29, 54–57], and a meta-analysis of eight RCTs which found reduced diastolic blood pressure with increased blueberry intake [58]; however, there was no association with systolic blood pressure in the latter study.

## Strengths and limitations

This study summarizes the findings from prospective cohort studies, which have the advantages of being less prone to selection bias and avoids recall bias. Combination of results from multiple cohort studies increases the statistical power to detect associations as several individual studies may have been too small and underpowered to detect an association. All the studies were considered to have moderately high methodological quality (see Supplementary Table 3). The dose–response relationship between fruit and vegetable intake and hypertension was investigated using both linear and nonlinear dose–response analyses and the results persisted across multiple subgroup and sensitivity analyses, suggesting that the overall findings were robust.

The study also has some potential limitations as it could be affected by biases that can affect observational studies. Confounding by other risk factors that have not been adjusted for or by unknown confounders is difficult to completely rule out. Fruit and vegetable consumption is typically associated with other health behaviors, such as physical activity, lower prevalence of smoking and alcohol consumption, and other dietary factors, that may themselves be associated with reduced hypertension risk, and could potentially confound the association between fruit and vegetable consumption and hypertension. Many of the included studies adjusted for a range of confounding factors and most of the results persisted in subgroup analyses when stratified by adjustment for confounding factors, and there was no evidence of heterogeneity between subgroups. In addition, some risk factors that were adjusted for in the statistical analyses may to some degree correlate with other unadjusted confounders, thus indirectly capture adjustment for some unknown or unadjusted confounders. The estimated *E*-values for the highest level of fruit and vegetable intake combined was 1.49 (lower CI 1.24) (800 vs. 40 g/d), and for total fruit was 1.76 (lower CI 1.40) (550 vs. 8 g/d), respectively, suggesting that an unadjusted confounder would have to have a moderate to strong association with both fruit and vegetable intake and hypertension to fully explain away the observed association. On the other hand, although BMI is often considered a confounder in such analyses, it is possible that BMI could be a mediator as high fruit and vegetable intake has been associated with lower risk of general and abdominal obesity, and lower weight gain over time [32–34]. If adiposity is a mediator, the observed associations may have been conservative estimates of the true relation given that the vast majority of studies adjusted for BMI. Differences between studies in the sample size, duration of follow-up and number of cases, geographic location, age, sex, detail of the dietary assessment methods, confounders adjusted for, preparation methods and types and amounts of fruits and vegetables consumed may have contributed to the observed heterogeneity. There was in general high heterogeneity in the analysis of fruit and vegetables combined and for total fruit, but heterogeneity was low in the analysis of total vegetables. For the analysis of fruit and vegetables combined, the heterogeneity was slightly lower in the subgroups of studies including both men and women, in studies from Europe and in studies with adjustment for other dietary factors, while for total fruit there was relatively high heterogeneity across subgroups. However, the heterogeneity was more driven by differences in the size of the risk estimates than differences in the direction of the associations as the vast majority of studies reported estimates in the direction of a reduced risk, and no studies reported a increase in hypertension risk with intakes of fruits and vegetables combined or separately.

There is also the risk of publication bias because studies that have statistically significant results are more likely to be published than studies with non-significant findings. Publication bias was assessed using Egger’s and Begg’s test and by inspection of funnel plots. There was no evidence of publication bias with the statistical tests used in the analyses of fruit and vegetables and hypertension, but there was indication of potential publication bias in the high vs. low analyses for fruit and hypertension, and vegetables and hypertension, however, this appeared to be driven by 1–2 outlying studies which did not substantially alter the results when excluded. There were few studies included in the analyses of subtypes of fruits and vegetables with only 2–5 studies included in each analysis. Further studies are therefore needed of specific subtypes of fruits and vegetables and hypertension risk.

The studies used different methods to assess the exposure and the outcome, and this may have influenced the results. Both measurement errors in dietary intake and changes in diet during follow-up is likely to lead to bias toward the null in cohort studies, resulting in underestimation of the association between dietary intake and the outcome of interest. However, there can also be measurement errors in the confounders, which sometimes can lead to residual confounding and overestimation of the association between the main exposure and the outcome, but presumably measurement of other risk factors such as weight, height, smoking and physical activity is less complex than diet, which typically includes 100–200 or more food items, thus potentially resulting in less error than measurement of diet. Most of the studies excluded participants with unrealistic energy at baseline that were likely to have substantially misreported their dietary intake. There were also some differences in the ways the outcome was measured. Blood pressure was either measured by the investigators or relied on self-report. Self-reported diagnosis of hypertension can be a potential limitation because there are no symptoms and cases could be underreported; however, some studies suggest the validity of this measure [30]. Although most studies used cut-off points of ≥140/≥90 mmHg for systolic/diastolic blood pressure to classify hypertension, a few studies used ≥135/≥85 or ≥130/≥85 mmHg. There was no significant heterogeneity between studies that used the various blood pressure cut-offs for the analysis of vegetables, and for fruits and vegetables combined there were no studies that used the lower cut-off point, while for fruits there was a stronger association for studies using a lower cut-off point. However, given the few studies in the latter subgroup analysis and the potential that other co-varying study characteristics could explain these findings, these findings should be interpreted with caution. There is the possibility that participants with healthy lifestyles are more likely to participate in research studies about health, and participants who consume more fruit and vegetables tend to follow other healthier lifestyles [59], however, this would mainly affect the external validity and not the internal validity of the results. Lastly, the initial literature screening was done by only one author and it is possible that studies have been missed, however, this seems less likely as we identified all studies included in previous meta-analyses. Although a protocol was developed for the project, it was not registered in a public registry.

### Mechanisms

Several possible mechanisms could explain the association between fruit and vegetable consumption and reduced risk of hypertension [29], including direct mechanisms as well as indirectly via weight reduction [60]. Fruits and vegetables are good sources of dietary fibre, vitamin C, vitamin E, folic acid, and potassium. Dietary fibre may affect blood pressure directly, or indirectly via other effects on weight change or insulin sensitivity [61]. The potential direct effects of fibres may involve vascular endothelial function, mineral absorption, effects on serum cholesterol, glycemic control, and gastrointestinal function [62]. Studies show that water-soluble fibres can reduce insulin resistance, and insulin resistance is thought to be an important mechanism for the development of hypertension [62]. Insulin is a metabolic hormone with vasodilatory actions that increases the delivery of insulin and glucose to target tissues, including skeletal muscle. Insulin can affect smooth muscles cells and endothelial function, promoting atherogenic dyslipidaemia [63]. Some randomized trials found that supplements of dietary fibres had a positive effect on weight loss, but also that dietary fibre may affect blood pressure independently of weight change [61]. Other components of fruit and vegetables that affect blood pressure are the minerals potassium and magnesium. Potassium and magnesium are associated with reduced blood pressure through regulation of vascular resistance, vasodilation [64, 65], and by improving endothelial function, modulating baroreflex sensitivity, and increasing antioxidant activity [66, 67]. A low potassium:sodium ratio decreases the synthesis of nitric oxide (NO), an important messenger molecule involved in the central regulation of blood pressure, and this leads to increased blood pressure [68]. Potassium restriction triggers cells to gain sodium [31, 69], which is associated with an increased risk of hypertension [29, 64, 70]. These nutrients have opposite effects on blood pressure, and there may be an additive effect of increasing potassium and reducing sodium intake [71]. Increasing potassium intake to correct the ratio will decrease salt sensitivity, reduce peripheral vascular resistance and lower blood pressure (16). A meta-analysis of randomized controlled trials found a U-shaped association between differences between active and control arms in urinary potassium excretion and systolic and diastolic blood pressure [72]. Fruits and vegetables are important sources of phytochemicals and antioxidants which can reduce oxidative stress, which is thought to contribute to the pathogenesis of hypertension and several other diseases [73]. Endothelial inflammation and formation of atherosclerotic plaque are the results of oxidative stress that has damaged cell membranes and lipoproteins. If circulating low-density lipoprotein cholesterol (LDL) is oxidized, it can contribute to the development of atherosclerosis, which is a risk factor for hypertension and other CVDs. Dietary antioxidants, including vitamin E, and vitamin C, may suppress activation of proinflammatory pathways through the quenching of free radicals and by enhancing the production and bioactivity of the potent vasodilator NO [74]. Folic acid is another vitamin which exerts beneficial effects on endothelial function, and has been associated with a decreased risk of hypertension in some studies [75, 76]. Meta-analyses of randomized trials have reported benefits of supplementation of vitamin C, vitamin E, and folic acid in reducing blood pressure [77–79]. A large body of evidence from observational studies and clinical trials documents that weight loss lowers BP [70]. A high fruit and vegetable intake has been associated with reduced weight gain over time [32–34] and this could contribute to a beneficial impact of fruit and vegetable consumption on risk of hypertension. Interestingly, some of the subtypes of fruits and vegetables that were most strongly associated with reduced weight gain (apples and pears, avocado, blueberries, raisins or grapes, broccoli, carrots, and lettuce) [32] were also the ones that appeared to be beneficial for the prevention of hypertension in the current analysis. Although many studies adjusted for BMI in the analysis, which might be considered an overadjustment, it is possible that fruits and vegetables could have an additional impact on hypertension through weight change over time. Lastly, increased intake of fruits and vegetables may have an overall positive impact on dietary patterns if they are consumed at the expense of other unhealthy foods. Intake of potatoes overall, and in particular fried potatoes, was associated with a significant increase in risk of hypertension, while the association for non-fried potatoes was less clear. A previous analysis found a positive association between consumption of potatoes, particularly fried potatoes (French fries) and greater weight gain [33], which is a major risk factor for hypertension. In addition, fried potatoes may have a high content of sodium, which is a known dietary risk factor for hypertension [80]. Positive associations were observed for a few other fruit and vegetable subtypes (e.g. cantaloupe, cruciferous vegetables, and Brussels sprouts), however, these analyses were based on only three studies each, and without any known biological mechanism to explain these associations, it is possible that they could be chance findings.

### Implications for research and practice

This meta-analysis provides a summary of the available evidence on fruit and vegetable consumption and risk of hypertension, and can be useful in further refining dietary guidelines given the global rise in hypertension prevalence. The results provide further support for dietary guidelines that recommend increasing the consumption of fruit and vegetables, and suggest a clear dose–response relationship with increasing intake up to 800 grams/day, which is consistent with data on other outcomes [9]. Clinicians can recommend an increased fruit and vegetable intake for patients with or at risk of hypertension (e.g., persons with overweight or obesity, or with type 2 diabetes) and the findings support recommendations to increase fruit and vegetable intake in the general population as well. Any further cohort studies should further investigate in more detail whether specific types of fruits and vegetables are particularly beneficial in reducing risk of hypertension. Previous studies have found substantial benefits of a high fruit and vegetable intake on the risk of coronary heart disease, stroke, cardiovascular disease overall and all-cause mortality, in addition to cancer [9], and the current findings of a benefit in relation to risk of hypertension is consistent with these findings.

## Conclusion

This meta-analysis found that a high intake of fruit and vegetables combined and total fruits, but not total vegetables, was associated with reduced risk of hypertension, and supports dietary recommendation to increase the consumption of fruit and vegetables as part of strategies to prevent hypertension. The association appeared to be linear up to an intake of 800 grams per day for fruits and vegetables combined. Specific types of fruits and vegetables appeared to be beneficial including apples and pears, avocado, blueberries, raisins or grapes, broccoli, carrots, lettuce, and onions, while cantaloupe, potatoes, and Brussels sprouts were associated with increased risk, however, further cohort studies are needed before firm conclusions can be drawn regarding subtypes of fruits and vegetables.

## Supplementary Information

Below is the link to the electronic supplementary material.

Supplementary file1 (PDF 792 KB)

Supplementary file2 (PDF 1323 KB)

## Data Availability

Data, material and analytical code will be made available upon reasonable request.
