# Supplementary material for: Fruit and vegetable consumption and the risk of hypertension: a systematic review and meta-analysis of prospective studies
Source: Eur J Nutr. 2023 Apr 27;62(5):1941–55. doi: 10.1007/s00394-023-03145-5 (PMC10349693; doi:10.1007/s00394-023-03145-5)
Supplement: Supplementary file 1 — Supplementary file1 (PDF 792 KB) [file 394_2023_3145_MOESM1_ESM.pdf]

Supplement for

Madsen H, Sen A, Aune D. Fruit and vegetable consumption and the risk of hypertension: a systematic review and meta-analysis of prospective studies. Eur J Nutr

https://doi.org/10.1007/s00394-023-03145-5

Supplementary text:

PubMed search

1) fruits OR vegetables OR fruit OR vegetable OR berry OR berries OR strawberries OR blueberries OR citrus OR “citrus fruits” OR orange OR apples OR pears OR banana OR cruciferae OR “cruciferous vegetables” OR broccoli OR cauliflower OR cabbages OR “allium vegetables” OR onion OR garlic OR tomato OR tomatoes OR potato OR "french fries" OR juice OR food OR "food groups"

2) hypertension OR "blood pressure"

3) “case-control” OR cohort OR cohorts OR prospective OR longitudinal OR retrospective OR “follow-up” OR “cross-sectional” OR “population-based” OR "relative risk" OR "hazard ratio" OR "incidence rate ratio" OR "odds ratio"

4) 1 AND 2 AND 3

Embase search

1) fruits OR vegetables OR fruit OR vegetable OR berry OR berries OR strawberries OR blueberries OR citrus OR citrus fruits OR orange OR apples OR pears OR banana OR cruciferae OR cruciferous vegetables OR broccoli OR cauliflower OR cabbages OR allium vegetables OR onion OR garlic OR tomato OR tomatoes OR potato OR french fries OR juice OR food OR food groups

2) fruits/ OR vegetables/ OR fruit/ OR vegetable/ OR berry/ OR berries/ OR strawberries/ OR blueberries/ OR citrus/ OR citrus fruits/ OR Orange/ OR apples/ OR pears/ OR banana/ OR cruciferae/ OR cruciferous vegetables/ OR broccoli/ OR cauliflower/ OR cabbages/ OR allium vegetables/ OR onion/ OR garlic/ OR tomato/ OR tomatoes/ OR potato/ OR french fries/ OR juice/ OR food/ OR food groups/

3) hypertension OR blood pressure

4) hypertension/ OR blood pressure/

5) case-control OR cohort OR cohorts OR prospective OR longitudinal OR retrospective OR follow-up OR cross-sectional OR hazard ratio OR incidence rate ratio OR relative risk OR odds ratio

6) 1 OR 2

7) 3 OR 4

8) 6 AND 7 AND 5

Supplementary table 1. List of studies excluded studies and exclusion reasons

Exclusion reason	Reference number
Abstract only publications	(1-7)
Case-control study	(8-17)
Case only study	(18)
Cross-sectional study	(19-107)
Duplicate	(108)
Intervention study (cross-sectional surveys included prevalent cases)	(109)
Meta-analysis	(110-114)
News	(115)
No risk estimates	(116-118)
Not relevant data	(119-168)
Not relevant exposure	(169-274)
Not relevant outcome	(275-321)
Outcome was continuous blood pressure or blood pressure trajectory	(322-334)
Patients with hypertension	(335-337)
Review	(338-344)
Study in adolescents (not adult population)	(345)

Reference List

(1) Bhanji S, Zaki S, Qidwai W, Ishaq M. (2012) Sociodemographic and lifestyle differences in known hypertensives and normotensives-results from community screening clinics. Journal of Hypertension Conference: 24th Meeting of the International Society of Hypertension Sydney, NSW Australia 30 (SUPPL 1):e48.

(2) Rodrigues PRM, Santana AMS, Ferrao MM, Gama A, Mourao I, Nogueira H, Rosado V, Padez C. (2013) Fruits consumption and high blood pressure in portuguese children. Annals of Nutrition and Metabolism Conference: Mediterranean Foods on Health and Disease - World Forum for Nutrition Research Conference

(3) Kandarina BJI, Susetyowati, Huriyati E. (2019) Fruit and vegetable consumption in rural and urban areas of Yogyakarta Indonesia. Annals of Nutrition and Metabolism Conference: 13th Asian Congress of Nutrition, ACN (3)

(4) Lipoeto NI, Putra DP, Sulastri D, Jalal F. (2020) Blood pressure and food consumption pattern of urban and rural people in west sumatra, Indonesia. Annals of Nutrition and Metabolism Conference

(5) Echeverria G, Jimenez P, D'Acuna S, Urquiaga I, Rigotti A, Leighton F. (2020) Consumption of mediterranean diet components is associated with lower cardiovascular risk. data from alimentate sano, a cross-sectional internet survey. Annals of Nutrition and Metabolism Conference

(6) Agarwal G, Pirrie M, Lee J, Angeles R, Marzanek F. (2019) Cardiovascular risk factors in social housing residents: A multi-site descriptive survey in older adults from ontario, canada. Canadian Journal of Cardiology Conference: Canadian Cardiovascular Congress (10 Supplement).

(7) Haiteng Z. (2019) The chronic non-communicable diseases prevalence, risk factors of hypertension and obesity, and nutrition evaluation for adult women in rural China: A cross-

sectional designed survey. Annals of Nutrition and Metabolism Conference: 13th Asian Congress of Nutrition, ACN (3).
(8) Lancaster KJ, Smiciklas-Wright H, Weitzel LB, Mitchell DC, Friedmann JM, Jensen GL. (2004) Hypertension-related dietary patterns of rural older adults. Prev Med 38(6):812-8.
(9) Martinez-Gonzalez MA. (2006) The SUN cohort study (Seguimiento University of Navarra). Public Health Nutr 9(1A):127-31.
(10) Kim MJ, Lee SJ, Ahn Y-H, Bowen P, Lee H. (2007) Dietary acculturation and diet quality of hypertensive Korean Americans. Journal of Advanced Nursing 58 (5):436-445.
(11) Dalai N, Cui H, Yan M, Rile G, Li S, Su X. (2014) Risk factors for the development of essential hypertension in a Mongolian population of China: a case-control study. Genet Mol Res 13(2):3283-91.
(12) Yu G, Fu H, Huang W, Zhang N, Deng D, Li G, Lei H. A (2018) Dietary Pattern of Higher Fish, Egg, Milk, Nut, Vegetable and Fruit, and Lower Salt Intake Correlates With the Prevalence and Control of Hypertension. Am J Hypertens 31(6):679-86.
(13) Zhang T, Chen Y, Qu Y, Wang M, Yin X. (2019) Diet-related factors for risk of hypertension in China: A case-control study. International Journal of Clinical and Experimental Medicine 12 (7):9399-9404.
(14) Lian F, Wang J, Huang X, Wu Y, Cao Y, Tan X, Xu X, Hong Y, Yang L, Gao X. (2015) Effect of vegetable consumption on the association between peripheral leucocyte telomere length and hypertension: a case-control study. BMJ Open 5(11):e009305.
(15) Devanaganvi AM, Shriharsha C, Natekar DS. (2020) Modifiable risk factors of hypertension: A hospital based case - control study at Bagalkot, Karnataka. Indian Journal of Public Health Research and Development 11(7):54-61.
(16) Evans RG, Subasinghe AK, Busingye D, Srikanth VK, Kartik K, Kalyanram K, Suresh O, Arabshahi S, Curkpatrick I, O'Dea K, Walker KZ, Kaye M, et al. (2021) Renal and dietary factors associated with hypertension in a setting of disadvantage in rural India. J Hum Hypertens 35(12):1118-28.
(17) Chukwu CE, Ebuehi OAT, Ajuluchukwu JNA, Olashore AHS. (2021) Anthropometric, socio-demographic and biochemical risk factors of hypertension in Lagos, Nigeria. Alexandria Journal of Medicine 57(1):44-51.
(18) Abimbola O, Ambrose I. (2016) Dietary habits of hypertensive patients in a tertiary hypertension clinic in Southern Nigeria. Journal of Medicine and Biomedical Research 15 (2):23-33.
(19) Joffres MR, Reed DM, Yano K. (1987) Relationship of magnesium intake and other dietary factors to blood pressure: the Honolulu heart study. Am J Clin Nutr 45(2):469-75.
(20) Ascherio A, Stampfer MJ, Colditz GA, Willett WC, McKinlay J. (1991) Nutrient intakes and blood pressure in normotensive males. Int J Epidemiol 20(4):886-91.
(21) Xu X, Niu T, Christiani DC, Weiss ST, Zhou Y, Chen C, Yang J, Fang Z, Jiang Z, Liang W, Zhang F. (1997) Environmental and occupational determinants of blood pressure in rural communities in China. Ann Epidemiol 7(2):95-106.
(22) La VC, Decarli A, Pagano R. (1998) Vegetable consumption and risk of chronic disease. Epidemiology 9(2):208-10.
(23) Takashima Y, Iwase Y, Yoshida M, Kokaze A, Takagi Y, Taubono Y, Tsugane S, Takahashi T, Iitoi Y, Akabane M, Watanabe S, Akamatsu T. (1998) Relationship of food intake and dietary patterns with blood pressure levels among middle-aged Japanese men. Journal of epidemiology / Japan Epidemiological Association 8(2):106-115.
(24) Yao M, Lichtenstein AH, Roberts SB, Ma G, Gao S, Tucker KL, McCrory MA. (2003) Relative influence of diet and physical activity on cardiovascular risk factors in urban Chinese adults. Int J Obes Relat Metab Disord 27(8):920-32.
(25) Alonso A, de la FC, Martin-Arnau AM, de IJ, Martinez JA, Martinez-Gonzalez MA. (2004) Fruit and vegetable consumption is inversely associated with blood pressure in a Mediterranean population with a high vegetable-fat intake: the Seguimiento Universidad de Navarra (SUN) Study. Br J Nutr 92(2):311-9.
(26) Dickson BK, Blackledge J, Hajjar IM. (2006) The impact of lifestyle behavior on hypertension awareness, treatment, and control in a southeastern population. American Journal of the Medical Sciences 332(4):211-215.

(27) Savitha MR, Krishnamurthy B, Fatthepur SS, Yashwanth Kumar AM, Khan MA. (2007) Essential hypertension in early and mid-adolescence. Indian J Pediatr 74(11):1007-11.
(28) Utsugi MT, Ohkubo T, Kikuya M, Kurimoto A, Sato RI, Suzuki K, Metoki H, Hara A, Tsubono Y, Imai Y. (2008) Fruit and vegetable consumption and the risk of hypertension determined by self measurement of blood pressure at home: the Ohasama study. Hypertens Res 31(7):1435-43.
(29) Wilson T, Singh AP, Vorsa N, Goettl CD, Kittleson KM, Roe CM, Kastello GM, Ragsdale FR. (2008) Human glycemic response and phenolic content of unsweetened cranberry juice. J Med Food 11(1):46-54.
(30) Radhika G, Sudha V, Mohan SR, Ganesan A, Mohan V. (2008) Association of fruit and vegetable intake with cardiovascular risk factors in urban south Indians. Br J Nutr 99(2):398-405.
(31) Borges HP, Cruz NDC, de Moura EC. (2008) Association between hypertension and overweight in adults in Belem, State of Para (Brazil). [Portuguese, English]. Arquivos Brasileiros de Cardiologia 91(2):99-106, 110-118.
(32) Wu H, Xu J, Zhuo L, Han L, Bao W, Rong S, Yao P, Ying C, Liu L. (2009) Comparison of risk factors associated with hypertension subtypes by classification tree method in Tongshan County of Jiangsu Province, China. Am J Hypertens 22(12):1287-94.
(33) Da Mata Ribeiro GB, Alves JGB. (2009) Prevalence of high blood pressure and associated factors in students from public schools in Greater Metropolitan Recife, Pernambuco State, Brazil, 2006. [Portuguese]. Cadernos de Saude Publica 25 (2):375-381.
(34) Carlsson AC, Wandell PE, Journath G, De FU, Hellenius M-L. (2009) Factors associated with uncontrolled hypertension and cardiovascular risk in hypertensive 60-year-old men and women-a population-based study. Hypertension Research 32(9):780-785.
(35) Celik F, Celik M, Akpolat V. (2009) Nutritional risk factors for the development of hypertension in diabetic patients. Journal of Diabetes and its Complications 23(5):304-309.
(36) de Ramirez SS, Enquobahrie DA, Nyadzi G, Mjungu D, Magombo F, Ramirez M, Sachs SE, Willett W. (2010) Prevalence and correlates of hypertension: a cross-sectional study among rural populations in sub-Saharan Africa. J Hum Hypertens 2010 24(12):786-95.
(37) Damasceno MM, de Araujo MF, de Freitas RW, de Almeida PC, Zanetti ML. (2011) The association between blood pressure in adolescents and the consumption of fruits, vegetables and fruit juice--an exploratory study. J Clin Nurs 20(11-12):1553-60.
(38) Wong SS, Beth DL, Gilbride JA, Chin WW, Kwan TW. (2011) Diet, physical activity, and cardiovascular disease risk factors among older Chinese Americans living in New York City. J Community Health 36(3):446-55.
(39) Singh RB, Fedacko J, Pella D, Macejova Z, Ghosh S, De AK, Begom R, Tumbi ZA, Haque M, Vajpeyee SK, de MF, Sergey C, et al. (2011) Prevalence and risk factors for prehypertension and hypertension in five Indian cities. Acta Cardiologica 66(1):29-37.
(40) de Souza BS, Garcia Rosa ML, Lugon JR, Yokoo EM, Mesquita ET, Rodrigues M, da Silva RC, Cagy M. (2011) Dietary habits and inadequate control of blood pressure in hypertensive adults assisted by a Brazilian Family Doctor Program. Public health nutrition 14 (12):2176-2184.
(41) Vernay M, Aidara M, Salanave B, Deschamps V, Malon A, Oleko A, Mallion JM, Hercberg S, Castetbon K. (2012) Diet and blood pressure in 18-74-year-old adults: the French Nutrition and Health Survey (ENNS, 2006-2007). J Hypertens 30(10):1920-7.
(42) Manandhar K, Koju R, Sinha NP, Humagain S. (2012) Prevalence and associated risk factors of hypertension among people aged 50 years and more in Banepa Municipality, Nepal. Kathmandu University Medical Journal 10(39):35-38.
(43) Peltzer K, Phaswana-Mafuya N. (2012) Fruit and vegetable intake and associated factors in older adults in South Africa. Global health action 5:1-8.
(44) Oude Griep LM, Stamler J, Chan Q, Van HL, Steffen LM, Miura K, Ueshima H, Okuda N, Zhao L, Daviglus ML, Elliott P. (2013) Association of raw fruit and fruit juice consumption with blood pressure: the INTERMAP Study. Am J Clin Nutr 97(5):1083-91.
(45) Chan Q, Stamler J, Brown IJ, Daviglus ML, Van HL, Dyer AR, Oude Griep LM, Miura K, Ueshima H, Zhao L, Nicholson JK, Holmes E, et al. (2013) Relations of raw and cooked vegetable consumption to blood pressure: the INTERMAP Study. Journal of Human Hypertension 21.
(46) Medina-Remon A, Vallverdu-Queralt A, Arranz S, Ros E, Martinez-Gonzalez MA, Sacanella E, Covas MI, Corella D, Salas-Salvado J, Gomez-Gracia E, Ruiz-Gutierrez V, Lapetra J, et al. (2013) Gazpacho consumption is associated with lower blood pressure and reduced hypertension in a high

cardiovascular risk cohort. Cross-sectional study of the PREDIMED trial. Nutrition, Metabolism and Cardiovascular Diseases 23(10):944-952.
(47) Kim MK, Kim K, Shin MH, Shin DH, Lee YH, Chun BY, Choi BY. (2014) The relationship of dietary sodium, potassium, fruits, and vegetables intake with blood pressure among Korean adults aged 40 and older. Nutr Res Pract 8(4):453-62.
(48) Ganesh KS, Deivanai SN. (2014) Prevalence and risk factors of hypertension among bank employees in urban Puducherry, India. International Journal of Occupational and Environmental Medicine 5(2):94-100.
(49) Helelo TP, Gelaw YA, Adane AA. (2014) Prevalence and associated factors of hypertension among adults in durame town, Southern Ethiopia. PLoS ONE 9(11):e112790.
(50) Pienovi L, Lara M, Bustos P, Amigo H. (2015) [Fruit and vegetable intake, and blood pressure. A population research]. Arch Latinoam Nutr 65(1):21-6.
(51) Chen X, Zou S, Wu X, Sun H, Ma B, Yang B, Zhang W, Shi J. (2016) Dietary Features and Blood Pressure among 18-88-Year-Old Residents in an Island Population in China. J Nutr Health Aging 20(2):107-13.
(52) Chhoun P, Tuot S, Harries AD, Kyaw NTT, Pal K, Mun P, Brody C, Mburu G, Yi S. (2017) High prevalence of non-communicable diseases and associated risk factors amongst adults living with HIV in Cambodia. PLoS ONE 12(11):e0187591.
(53) Rouhani MH, Agh F, Azadbakht L. (2018) Pickle Consumption is Associated with Body Mass Index and Blood Pressure among Iranian Female College Students: a Cross-Sectional Study. Clin Nutr Res 7(4):256-65.
(54) Liu Z, Man QQ, Li YQ, Wang JZ, Zhang J, Zhao WH. (2018) [The relationship between prevalence of hypertension and dietary factors among the elderly aged 60 years old and over in China during 2010-2012]. Zhonghua Yu Fang Yi Xue Za Zhi 52(6):622-8.
(55) Yang Y, Dong B, Zou Z, Wang S, Dong Y, Wang Z, Ma J. (2018) Association between Vegetable Consumption and Blood Pressure, Stratified by BMI, among Chinese Adolescents Aged 13-17 Years: A National Cross-Sectional Study. Nutrients 10(4).
(56) Zhou H, Wang K, Zhou X, Ruan S, Gan S, Cheng S, Lu Y. (2018) Prevalence and Gender-Specific Influencing Factors of Hypertension among Chinese Adults: A Cross-Sectional Survey Study in Nanchang, China. Int J Environ Res Public Health 15(2).
(57) Pengpid S, Vonglokham M, Kounnavong S, Sychareun V, Peltzer K. (2019) The Prevalence and Social Determinants of Fruit and Vegetable Consumption and Its Associations With Noncommunicable Diseases Risk Factors Among Adults in Laos. Asia Pac J Public Health 31(2):157-66.
(58) Sackou JK, Tiade ML, Hounsa AA, Malik SK, Coulibaly M, Desquith AA, Kadjo FK, Agoua SA, Oga S, Kouadio LK. (2019) Prevalence and factors associated with hypertension in anonkoi 3, a peri-urban area in abidjan (Cote d'ivoire). Journal of Public Health in Africa 10(2):1106.
(59) Acheampong K, Nyamari JM, Ganu D, Appiah S, Pan X, Kaminga A, Liu A. (2019) Predictors of Hypertension among Adult Female Population in Kpone-Katamanso District, Ghana. International Journal of Hypertension 1876060.
(60) Fowokan A, Punthakee Z, Waddell C, Rosin M, Morrison KM, Gupta M, Rangarajan S, Teo K, Lear S. (2019) Multifactorial correlates of blood pressure in South Asian children in Canada: A cross-sectional study. BMJ Open 9(4):e027844.
(61) Zhang S, Liu M, Wang Y, Zhang Q, Liu L, Meng G, Yao Z, Wu H, Xia Y, Bao X, Gu Y, Wang H, et al. (2020) Raw garlic consumption is inversely associated with prehypertension in a large-scale adult population. J Hum Hypertens 34(1):59-67.
(62) Fukunaga A, Inoue Y, Chandraratne N, Yamaguchi M, Kuwahara K, Indrawansa S, Gunawardena N, Mizoue T, Samarasinghe D. (2020) Healthy lifestyle index and its association with hypertension among community adults in Sri Lanka: A cross-sectional study. PLoS ONE 15(1):e0226773.
(63) Costa Filho FF, Timerman A, Saraiva JFK, Magalhaes CC, Pinto IMF, Oliveira GBF, Mattos AC, Avezum A. (2020) Independent predictors of effective blood pressure control in patients with hypertension on drug treatment in Brazil. Journal of Clinical Hypertension 20(1):125-132.
(64) Ascherio A, Stampfer MJ, Colditz GA, Willett WC, McKinlay J. (1991) Nutrient intakes and blood pressure in normotensive males. International Journal of Epidemiology 20;(4):886-91.

(65) Williams PT, Fortmann SP, Terry RB, Garay SC, Vranizan KM, Ellsworth N, Wood PD. (1987) Associations of dietary fat, regional adiposity, and blood pressure in men. JAMA 257(23):3251-6.
(66) Xu X, Byles JE, Shi Z, Hall JJ. (2018) Dietary patterns, dietary lead exposure and hypertension in the older Chinese population. Asia Pac J Clin Nutr 27(2):451-9.
(67) Abidoye RO, Izunwa RD, Akinkuade FO, Abidoye GO. (2002) Inter-relationships between lifestyle and diabetes mellitus, overweight/ obesity and hypertension in Nigeria. Nutrition and Health 16(3):203
(68) Song HJ, Paek YJ, Choi MK, Lee HJ. (2014) Gender differences in the relationship between risk of hypertension and fruit intake. Prev Med 67:154-9.
(69) Ganesh KS, Naresh AGV, Bammigatti C. (2014) Prevalence and risk factors of hypertension among male police personnel in urban puducherry, India. Kathmandu University Medical Journal 12(4):242-246.
(70) Feng L, Li P, Hu Z, Ma Y, Tang W, Ben Y, Mahapatra T, Cao X, Mahapatra S, Ling M, Gou A, Wang Y, et al. (2014) Distribution and determinants of non communicable diseases among elderly uyghur ethnic group in Xinjiang, China. PLoS ONE 9(8):e105536.
(71) Katchunga PB, Twagirumukiza M, Kluyskens Y, Kaishusha D, Baguma M, Bapolisi A, Cikomola J, Ntabure T, Callens S, M'Buyamba-Kabangu JR, Van BL. (2015) Blood pressure in the Congolese adult population of South Kivu, Democratic Republic of Congo: Preliminary results from the Bukavu Observ Cohort Study. Rev Epidemiol Sante Publique 63(6):339-45.
(72) Lelong H, Galan P, Kesse-Guyot E, Fezeu L, Hercberg S, Blacher J. (2015) Relationship between nutrition and blood pressure: A cross-sectional analysis from the Nutrinet-sante study, a French web-based cohort study. American Journal of Hypertension 28(3):362-371.
(73) Domingos TB, Pereira AF, Yokoo EM, Salles-Costa R. (2016) Low fruit consumption and omission of daily meals as risk factors for increased blood pressure in adults. Br J Nutr 116(4):683-91.
(74) Freedman MR, Fulgoni VL, III. (2016) Canned Vegetable and Fruit Consumption Is Associated with Changes in Nutrient Intake and Higher Diet Quality in Children and Adults: National Health and Nutrition Examination Survey 2001-2010. J Acad Nutr Diet 116(6):940-8.
(75) Ahola AJ, Freese R, Makimattila S, Forsblom C, Groop P-H. (2016) Dietary patterns are associated with various vascular health markers and complications in type 1 diabetes. Journal of Diabetes and its Complications 30(6):1144-1150.
(76) Hong SA, Kim MK. (2017) Relationship between fruit and vegetable intake and the risk of metabolic syndrome and its disorders in Korean women according to menopausal status. Asia Pac J Clin Nutr 26(3):514-23.
(77) Saeed KMI. (2017) Burden of Hypertension in the Capital of Afghanistan: A Cross-Sectional Study in Kabul City, 2015. International Journal of Hypertension 3483872.
(78) Danushka N, Pradeep J. (2018) Prevalence of hypertension in sri lankan rural setting. Journal of Hypertension Conference: 27th Scientific Meeting of the International Society of Hypertension, ISH (Supplement 3):October.
(79) Leung AA, Bushnik T, Hennessy D, McAlister FA, Manuel DG. (2019) Risk factors for hypertension in Canada. Health Rep 30(2):3-13.
(80) Zhou L, Feng Y, Yang Y, Zhao X, Fan Y, Rong J, Liu D, Zhao L, Yu Y. (2019) Diet behaviours and hypertension in US adults: the National Health and Nutrition Examination Survey 2013-2014. J Hypertens 37(6):1230-8.
(81) Choi A, Ha K, Joung H, Song Y. (2019) Frequency of Consumption of Whole Fruit, Not Fruit Juice, Is Associated with Reduced Prevalence of Obesity in Korean Adults. Journal of the Academy of Nutrition and Dietetics 119(11):1842-1851.
(82) AlKaabi LA, Ahmed LS, Al Attiyah MF, bdel-Rahman ME. (2020) Predicting hypertension using machine learning: Findings from Qatar Biobank Study. PLoS One 15(10):e0240370.
(83) Nepali S, Rijal A, Olsen MH, McLachlan CS, Kallestrup P, Neupane D. (2020) Factors affecting the fruit and vegetable intake in Nepal and its association with history of self-reported major cardiovascular events. BMC Cardiovasc Disord 20(1):425.
(84) Sungwa EE, Kibona SE, Dika HI, Laisser RM, Gemuhay HM, Kabalimu TK, Kidenya BR. (2020) Prevalence and factors that are associated with elevated blood pressure among primary school children in Mwanza Region, Tanzania. The Pan African medical journal 37:283.

(85) Mphekgwana PM, Malema N, Monyeki KD, Mothiba TM, Makgahlela M, Kgatla N, Makgato I, Sodi T. (2020) Hypertension prevalence and determinants among black South African adults in semi-urban and rural areas. International Journal of Environmental Research and Public Health 17(20):7463.
(86) Ongosi AN, Wilunda C, Musumari PM, Techasrivichien T, Wang C-W, Ono-Kihara M, Serrem C, Kihara M, Nakayama T. (2020) Prevalence and risk factors of elevated blood pressure and elevated blood glucose among residents of kajiado county, kenya: A population-based cross-sectional survey. International Journal of Environmental Research and Public Health 17(19):6957.
(87) Chuka A, Gutema BT, Ayele G, Megersa ND, Melketsedik ZA, Zewdie TH. (2020) Prevalence of hypertension and associated factors among adult residents in Arba Minch Health and Demographic Surveillance Site, Southern Ethiopia. PLoS ONE 15(8):e0237333.
(88) Wangdi K, Jamtsho T. (2020) Prevalence and predisposing factors for self-reported hypertension in Bhutanese adults. Nepal Journal of Epidemiology 10(1):830-840.
(89) Maksimova Z, Maksimov DM. (2020) Hypertension in working age population: Influence of gender and education'. Kardiologiya 60(2):24-32.
(90) Kim J, Jeong K, Lee S, Baek Y. (2021) Relationship between Low Vegetable Consumption, Increased High-Sensitive C-Reactive Protein Level, and Cardiometabolic Risk in Korean Adults with Tae-Eumin: A Cross-Sectional Study. Evid Based Complement Alternat Med 2021:3631445.
(91) Becerra-Tomas N, Paz-Graniel I, Tresserra-Rimbau A, Martinez-Gonzalez MÃ, Barrubes L, Corella D, Munoz-Martinez J, Romaguera D, Vioque J, Alonso-Gomez ÃM, Warnberg J, Martinez JA, et al. (2021) Fruit consumption and cardiometabolic risk in the PREDIMED-plus study: A cross-sectional analysis. Nutr Metab Cardiovasc Dis 31(6):1702-13.
(92) Elsahoryi NA, Neville CE, Patterson CC, Linden GJ, Moitry M, Biasch K, Kee F, Amouyel P, Bongard V, Dallongeville J, Ferrieres J, Woodside JV. (2021) Association between overall fruit and vegetable intake, and fruit and vegetable sub-types and blood pressure: the PRIME study (Prospective Epidemiological Study of Myocardial Infarction). Br J Nutr 125(5):557-67.
(93) Kumma WP, Lindtjorn B, Loha E. (2021) Prevalence of hypertension, and related factors among adults in Wolaita, southern Ethiopia: A community-based cross-sectional study. PLoS ONE 16(12):e0260403.
(94) Coletro HN, Bressan J, Diniz AP, Hermsdorff HHM, Pimenta AM, Meireles AL, Mendonca RDD, Carraro JCC. (2021) Total Polyphenol Intake, Polyphenol Subtypes, and Prevalence of Hypertension in the CUME Cohort. Journal of the American College of Nutrition [Epub online ahead of print]
(95) Musung JM, Kakoma PK, Kaut MC, Tshimanga SL, Munkemena Banze JP, Kaj NK, Kamuna MK, Mwamba JK, Nkulu DN, Katchunga PB, Mukuku O, Muyumba EK. (2021) Prevalence of Hypertension and Associated Factors in Lubumbashi City, Democratic Republic of Congo: A Community-Based Cross-Sectional Study. International Journal of Hypertension 6674336.
(96) Mosisa G, Regassa B, Biru B. (2021) Epidemiology of hypertension in selected towns of Wollega zones, Western Ethiopia, 2019: A community-based cross-sectional study. SAGE Open Medicine [Epub online ahead of print].
(97) Alemi S, Nakamura K, Arab AS, Mashal MO, Tashiro Y, Seino K, Hemat S. (2021) Gender-specific prevalence of risk factors for non-communicable diseases by health service use among schoolteachers in Afghanistan. International Journal of Environmental Research and Public Health 18(11):5729.
(98) Simmons SS, Hagan JE, Schack T. (2021) The influence of anthropometric indices and intermediary determinants of hypertension in bangladesh. International Journal of Environmental Research and Public Health 18(11):5646.
(99) Anjulo U, Haile D, Wolde A. (2021) Prevalence of hypertension and its associated factors among adults in Areka town, Wolaita zone, southern Ethiopia. Integrated Blood Pressure Control 14:43-54.
(100) Song S, Song YJ. (2021) Dietary fiber and its source are associated with cardiovascular risk factors in korean adults. Nutrients 13(1):160.
(101) Patterson CC, Linden GJ, Moitry M, Biasch K, Kee F, Amouyel P, Bongard V, Dallongeville J, Ferrieres J, Woodside JV. (2021) Association between overall fruit and vegetable intake, and fruit and vegetable sub-types and blood pressure: The PRIME study (Prospective Epidemiological Study of Myocardial Infarction). British Journal of Nutrition 125(5):557-567.

(102) Norris T, Girma M, Genye T, Hussen A, Pradeilles R, Bekele Z, van ZC, Samuel A. (2022) Predictors of prediabetes/diabetes and hypertension in Ethiopia: Reanalysis of the 2015 NCD STEPS survey using causal path diagrams. Matern Child Nutr e13365.
(103) Kibe LW, Bazargan M. (2022) Fruit and Vegetable Intake Among Older African American and Hispanic Adults With Cardiovascular Risk Factors. Gerontol Geriatr Med 23337214211057730.
(104) Prihartono NA, Fitria L, Ramdhan DH, Fitriyani F, Fauzia S, Woskie S. (2022) Determinants of Hypertension amongst Rice Farmers in West Java, Indonesia. Int J Environ Res Public Health 19(3):1152.
(105) Poorolajal J, Mohammadi Y, Doosti-Irani A, Khosh-Manesh S. (2022) The contribution of avoidable factors in doubling or halving the odds of hypertension. PLoS ONE 17(4):e0266689.
(106) Queralt A, Molina-Garcia J. (2012) Cardiovascular risk factors in relation to leisure-time physical activity and fruit-vegetable consumption. Journal of Science and Medicine in Sport Conference: Be Active December.
(107) Psaltopoulou T, Naska A, Orfanos P, Trichopoulos D, Mountokalakis T, Trichopoulou A. (2004) Olive oil, the Mediterranean diet, and arterial blood pressure: the Greek European Prospective Investigation into Cancer and Nutrition (EPIC) study. Am J Clin Nutr 80(4):1012-8.
(108) Li X, Zhang W, Laden F, Curhan GC, Rimm EB, Guo X, Hart JE, Wu S. (2022) Dietary nitrate intake and vegetable consumption, ambient particulate matter, and risk of hypertension in the Nurses' Health study. Environment International 161:107100.
(109) Barnidge EK, Baker EA, Schootman M, Motton F, Sawicki M, Rose F. (2015) The effect of education plus access on perceived fruit and vegetable consumption in a rural African American community intervention. Health Educ Res 30(5):773-85.
(110) Hartley L, Igbinedion E, Holmes J, Flowers N, Thorogood M, Clarke A, Stranges S, Hooper L, Rees K. (2013) Increased consumption of fruit and vegetables for the primary prevention of cardiovascular diseases. Cochrane Database Syst Rev (6):CD009874.
(111) Li B, Li F, Wang L, Zhang D. (2016) Fruit and Vegetables Consumption and Risk of Hypertension: A Meta-Analysis. J Clin Hypertens (Greenwich) 18(5):468-76.
(112) Gayer BA, Avendano EE, Edelson E, Nirmala N, Johnson EJ, Raman G. (2019) Effects of Intake of Apples, Pears, or Their Products on Cardiometabolic Risk Factors and Clinical Outcomes: A Systematic Review and Meta-Analysis. Curr Dev Nutr 3(10):nzz109.
(113) Kim J, Hoang T, Bu SY, Kim JM, Choi JH, Park E, Lee SM, Park E, Min JY, Lee IS, Youn SY. (2020) Associations of Dietary Intake with Cardiovascular Disease, Blood Pressure, and Lipid Profile in the Korean Population: a Systematic Review and Meta-Analysis. J Lipid Atheroscler 9(1):205-29.
(114) Dinu M, Sofi F, Volpe M, Strazzullo P, Bordoni A, Cairella G, Casiraghi MC, D'Elia L, del B, V, Erba D, Garbagnati F, Ghiselli A, et al. (2021) 100% Fruit juice intake and cardiovascular risk: a systematic review and meta-analysis of prospective and randomised controlled studies. European Journal of Nutrition 60(5):2449-2467.
(115) No authors listed (2016) Higher potato intake associated with hypertension risk. Nursing older people 28(6):12
(116) Al-Nooh AA, bdulabbas Abdulla AA, Wood D. (2014) The prevalence of cardiovascular disease risk factors among employees in the kingdom of Bahrain between October 2010 and March 2011: A cross-sectional study from a workplace health campaign. Cardiology Research and Practice 832421.
(117) Lim OW, Liew HH, Eng XR, Yong CK, Lim LH. (2021) Determinants of Newly-Diagnosed Raised Blood Pressure: The Malaysian Context. Malays J Med Sci 28(6):88-99.
(118) Khalesi S, Sharma S, Irwin C, Sun J. (2016) Dietary patterns, nutrition knowledge and lifestyle: associations with blood pressure in a sample of Australian adults (the Food BP study). J Hum Hypertens 30(10):581-90.
(119) Lindahl O, Lindwall L, Spangberg A, Stenram A, Ockerman PA. (1984) A vegan regimen with reduced medication in the treatment of hypertension. Br J Nutr 52(1):11-20.
(120) No authors listed. (1986) Nonpharmacological approaches to the control of high blood pressure. Final report of the Subcommittee on Nonpharmacological Therapy of the 1984 Joint National Committee on Detection, Evaluation, and Treatment of High Blood Pressure. Hypertension 8(5):444-67.

(121) Burr ML, Butland BK. (1988) Heart disease in British vegetarians. Am J Clin Nutr 48(3 Suppl):830-2.
(122) Singh RB, Rastogi SS, Singh R, Ghosh S, Niaz MA. (1992) Effects of guava intake on serum total and high-density lipoprotein cholesterol levels and on systemic blood pressure. Am J Cardiol 70(15):1287-91.
(123) Singh RB, Niaz MA, Ghosh S, Singh R, Rastogi SS. (1993) Effect on mortality and reinfarction of adding fruits and vegetables to a prudent diet in the Indian experiment of infarct survival (IEIS). J Am Coll Nutr 12(3):255-61.
(124) Knekt P, Jarvinen R, Reunanen A, Maatela J. (1996) Flavonoid intake and coronary mortality in Finland: a cohort study. BMJ 312(7029):478-81.
(125) Post GB, Kemper HC, Twisk J, Van MW. (1997) The association between dietary patterns and cardio vascular disease risk indicators in healthy youngsters: results covering fifteen years of longitudinal development. Eur J Clin Nutr 51(6):387-93.
(126) Haynes RB, Kris-Etherton P, McCarron DA, Oparil S, Chait A, Resnick LM, Morris CD, Clark S, Hatton DC, Metz JA, McMahon M, Holcomb S, et al. (1999) Nutritionally complete prepared meal plan to reduce cardiovascular risk factors: a randomized clinical trial. J Am Diet Assoc 99(9):1077-83.
(127) Stamler J, Liu K, Ruth KJ, Pryer J, Greenland P. (2002) Eight-year blood pressure change in middle-aged men: relationship to multiple nutrients. Hypertension 39(5):1000-6.
(128) Teixeira RC, Molina MC, Zandonade E, Mill JG. (2007) Cardiovascular risk in vegetarians and omnivores: a comparative study. Arq Bras Cardiol 89(4):237-44.
(129) Folsom AR, Parker ED, Harnack LJ. (2007) Degree of concordance with DASH diet guidelines and incidence of hypertension and fatal cardiovascular disease. Am J Hypertens 20(3):225-32.
(130) Danesh J, Saracci R, Berglund G, Feskens E, Overvad K, Panico S, Thompson S, Fournier A, Clavel-Chapelon F, Canonico M, Kaaks R, Linseisen J, et al. (2007) EPIC-Heart: the cardiovascular component of a prospective study of nutritional, lifestyle and biological factors in 520,000 middle-aged participants from 10 European countries. Eur J Epidemiol 22(2):129-41.
(131) Masala G, Bendinelli B, Versari D, Saieva C, Ceroti M, Santagiuliana F, Caini S, Salvini S, Sera F, Taddei S, Ghiadoni L, Palli D. (2008) Anthropometric and dietary determinants of blood pressure in over 7000 Mediterranean women: the European Prospective Investigation into Cancer and Nutrition-Florence cohort. J Hypertens 26(11):2112-20.
(132) Estruch R, Martinez-Gonzalez MA, Corella D, Basora-Gallisa J, Ruiz-Gutierrez V, Covas MI, Fiol M, Gomez-Gracia E, Lopez-Sabater MC, Escoda R, Pena MA, Diez-Espino J, et al. (2009) Effects of dietary fibre intake on risk factors for cardiovascular disease in subjects at high risk. J Epidemiol Community Health 63(7):582-8.
(133) Hostmark AT. (2010) The Oslo Health Study: a Dietary Index estimating high intake of soft drinks and low intake of fruits and vegetables was positively associated with components of the metabolic syndrome. Appl Physiol Nutr Metab 35(6):816-25.
(134) Fitzgerald KC, Chiuve SE, Buring JE, Ridker PM, Glynn RJ. (2012) Comparison of associations of adherence to a Dietary Approaches to Stop Hypertension (DASH)-style diet with risks of cardiovascular disease and venous thromboembolism. J Thromb Haemost 10(2):189-98.
(135) Matsusima A, Furuuchi R, Sakaguchi Y, Goto H, Yokoyama T, Nishida H, Hirayama M. (2013) Acute and chronic flow-mediated dilation and blood pressure responses to daily intake of boysenberry juice: a preliminary study. Int J Food Sci Nutr 64(8):988-92.
(136) van den Hil LC, Rob TH, de Jonge LL, Heppe DH, Steegers EA, Hofman A, van der Heijden AJ, Jaddoe VW. (2013) Maternal first-trimester dietary intake and childhood blood pressure: the Generation R Study. Br J Nutr 110(8):1454-64.
(137) Tielemans SM, Kromhout D, torf-van der KW, Geleijnse JM. (2014) Associations of plant and animal protein intake with 5-year changes in blood pressure: the Zutphen Elderly Study. Nutr Metab Cardiovasc Dis 24(11):1228-33.
(138) Hikmat F, Appel LJ. (2014) Effects of the DASH diet on blood pressure in patients with and without metabolic syndrome: results from the DASH trial. J Hum Hypertens 28(3):170-5.
(139) Urquiaga I, D'Acuna S, Perez D, Dicenta S, Echeverria G, Rigotti A, Leighton F. (2015) Wine grape pomace flour improves blood pressure, fasting glucose and protein damage in humans: a randomized controlled trial. Biol Res 48:49.

(140) Julia C, Fezeu LK, Ducrot P, Mejean C, Peneau S, Touvier M, Hercberg S, Kesse-Guyot E. (2015) The Nutrient Profile of Foods Consumed Using the British Food Standards Agency Nutrient Profiling System Is Associated with Metabolic Syndrome in the SU.VI.MAX Cohort. J Nutr 145(10):2355-61.
(141) Aryal KK, Mehata S, Neupane S, Vaidya A, Dhimal M, Dhakal P, Rana S, Bhusal CL, Lohani GR, Paulin FH, Garg RM, Guthold R, et al. (2015) The Burden and Determinants of Non Communicable Diseases Risk Factors in Nepal: Findings from a Nationwide STEPS Survey. PLoS One 10(8):e0134834.
(142) Krupp D, Shi L, Egert S, Wudy SA, Remer T. (2015) Prospective relevance of fruit and vegetable consumption and salt intake during adolescence for blood pressure in young adulthood. Eur J Nutr 54(8):1269-79.
(143) Buendia JR, Bradlee ML, Singer MR, Moore LL. (2015) Diets higher in protein predict lower high blood pressure risk in Framingham Offspring Study adults. Am J Hypertens 28(3):372-9.
(144) Hosseinpour-Niazi S, Mirmiran P, Mirzaei S, Azizi F. (2015) Cereal, fruit and vegetable fibre intake and the risk of the metabolic syndrome: a prospective study in the Tehran Lipid and Glucose Study. J Hum Nutr Diet 28(3):236-45.
(145) Farajian P, Panagiotakos DB, Risvas G, Micha R, Tsioufis C, Zampelas A. (2015) Dietary and lifestyle patterns in relation to high blood pressure in children: the GRECO study. J Hypertens 33(6):1174-81.
(146) Li P, Zhang M, Zhu Y, Liu W, Zhang Y, Gao Y, Huang G. (2016) Dietary patterns and changes in cardiovascular risk factors in apparently healthy Chinese women: a longitudinal study. J Clin Biochem Nutr 58(3):232-9.
(147) Julian-Almarcegui C, Vandevijvere S, Gottrand F, Beghin L, Dallongeville J, Sjostrom M, Leclercq C, Manios Y, Widhalm K, Ferreira De Morares AC, Gonzalez-Gross M, Stehle P, et al. (2016) Association of heart rate and blood pressure among European adolescents with usual food consumption: The HELENA study. Nutr Metab Cardiovasc Dis 26(6):541-8.
(148) Angelopoulos TJ, Lowndes J, Sinnett S, Rippe JM. (2016) Fructose Containing Sugars at Normal Levels of Consumption Do Not Effect Adversely Components of the Metabolic Syndrome and Risk Factors for Cardiovascular Disease. Nutrients 8(4):179.
(149) Jeong HS, Hong SJ, Cho JY, Lee TB, Kwon JW, Joo HJ, Park JH, Yu CW, Lim DS. (2016) Effects of Rubus occidentalis extract on blood pressure in patients with prehypertension: Randomized, double-blinded, placebo-controlled clinical trial. Nutrition 32(4):461-7.
(150) Park E, Edirisinghe I, Choy YY, Waterhouse A, Burton-Freeman B. (2016) Effects of grape seed extract beverage on blood pressure and metabolic indices in individuals with pre-hypertension: a randomised, double-blinded, two-arm, parallel, placebo-controlled trial. Br J Nutr 115(2):226-38.
(151) Moore LL, Singer MR, Bradlee ML, Daniels SR. (2016) Adolescent dietary intakes predict cardiometabolic risk clustering. Eur J Nutr 55(2):461-8.
(152) Godos J, Sinatra D, Blanco I, Mule S, La VM, Marranzano M. (2017) Association between Dietary Phenolic Acids and Hypertension in a Mediterranean Cohort. Nutrients 9(10):1069.
(153) Asghari G, Momenan M, Yuzbashian E, Mirmiran P, Azizi F. (2018) Dietary pattern and incidence of chronic kidney disease among adults: a population-based study. Nutr Metab (Lond) 15:88.
(154) Gu A, Yue Y, Kim J, Argulian E. (2018) The Burden of Modifiable Risk Factors in Newly Defined Categories of Blood Pressure. Am J Med 131(11):1349-58.
(155) Chai SC, Davis K, Wright RS, Kuczmarski MF, Zhang Z. (2018) Impact of tart cherry juice on systolic blood pressure and low-density lipoprotein cholesterol in older adults: a randomized controlled trial. Food Funct 9(6):3185-94.
(156) Monge A, Lajous M, Ortiz-Panozo E, Rodriguez BL, Gongora JJ, Lopez-Ridaura R. (2018) Western and Modern Mexican dietary patterns are directly associated with incident hypertension in Mexican women: a prospective follow-up study. Nutr J 17(1):21.
(157) Mellendick K, Shanahan L, Wideman L, Calkins S, Keane S, Lovelady C. (2018) Diets Rich in Fruits and Vegetables Are Associated with Lower Cardiovascular Disease Risk in Adolescents. Nutrients 10(2).
(158) Bahari T, Uemura H, Katsuura-Kamano S, Yamaguchi M, Nakamoto M, Miki K, Ishizu M, Arisawa K. (2018) Nutrient-Derived Dietary Patterns and Their Association With Metabolic Syndrome in a Japanese Population. J Epidemiol 28(4):194-201.

(159) Jones T, Dunn EL, Macdonald JH, Kubis HP, McMahon N, Sandoo A. (2019) The Effects of Beetroot Juice on Blood Pressure, Microvascular Function and Large-Vessel Endothelial Function: A Randomized, Double-Blind, Placebo-Controlled Pilot Study in Healthy Older Adults. Nutrients 11(8):1792.
(160) Curtis PJ, van dV, V, Berends L, Jennings A, Feelisch M, Umpleby AM, Evans M, Fernandez BO, Meiss MS, Minnion M, Potter J, Minihane AM, et al. (2019) Blueberries improve biomarkers of cardiometabolic function in participants with metabolic syndrome-results from a 6-month, double-blind, randomized controlled trial. Am J Clin Nutr 109(6):1535-45.
(161) Lum T, Connolly M, Marx A, Beidler J, Hooshmand S, Kern M, Liu C, Hong MY. (2019) Effects of Fresh Watermelon Consumption on the Acute Satiety Response and Cardiometabolic Risk Factors in Overweight and Obese Adults. Nutrients 11(3):595.
(162) Bawaked RA, Fernandez-Barres S, Navarrete-Munoz EM, Gonzalez-Palacios S, Guxens M, Irizar A, Lertxundi A, Sunyer J, Vioque J, Schroder H, Vrijheid M, Romaguera D. (2020) Impact of lifestyle behaviors in early childhood on obesity and cardiometabolic risk in children: Results from the Spanish INMA birth cohort study. Pediatr Obes 15(3):e12590.
(163) Zafarmand MH, Spanjer M, Nicolaou M, Wijnhoven HAH, van Schaik BDC, Uitterlinden AG, Snieder H, Vrijkotte TGM. (2020) Influence of Dietary Approaches to Stop Hypertension-Type Diet, Known Genetic Variants and Their Interplay on Blood Pressure in Early Childhood: ABCD Study. Hypertension 75(1):59-70.
(164) Yuan S, Yu HJ, Liu MW, Tang BW, Zhang J, Gasevic D, Larsson SC, He QQ. (2020) Fat Intake and Hypertension Among Adults in China: The Modifying Effects of Fruit and Vegetable Intake. Am J Prev Med 58(2):294-301.
(165) Guillermo C, Boushey CJ, Franke AA, Monroe KR, Lim U, Wilkens LR, Le ML, Maskarinec G. (2020) Diet Quality and Biomarker Profiles Related to Chronic Disease Prevention: The Multiethnic Cohort Study. J Am Coll Nutr 39(3):216-23.
(166) Bondonno CP, Dalgaard F, Blekkenhorst LC, Murray K, Lewis JR, Croft KD, KyrÃ¸ C, Torp-Pedersen C, Gislason G, TjÃ¸nneland A, Overvad K, Bondonno NP, et al. (2021) Vegetable nitrate intake, blood pressure and incident cardiovascular disease: Danish Diet, Cancer, and Health Study. Eur J Epidemiol 36(8):813-25.
(167) Amin TT, Al Sultan AI, Mostafa OA, Darwish AA, Al-Naboli MR. (2014) Profile of non-communicable disease risk factors among employees at a Saudi university. Asian Pacific journal of cancer prevention 15(18):7897-7907.
(168) Gao M, Ikeda K, Hattori H, Miura A, Nara Y, Yamori Y. (1999) Cardiovascular risk factors emerging in Chinese populations undergoing urbanization. Hypertension Research - Clinical and Experimental 22(3):209.
(169) Talreja S, Talreja A, Talreja DR. (2020) An investigation of plant-based, mediterranean, paleolithic, and dash diets study in comparison (Va beach diet study). Journal of the American College of Cardiology Conference: ACC 20 - World Congress of Cardiology United States 75 (11 Supplement 1):1900
(170) Ascherio A, Rimm EB, Giovannucci EL, Colditz GA, Rosner B, Willett WC, Sacks F, Stampfer MJ. (1992) A prospective study of nutritional factors and hypertension among US men. Circulation 86(5):1475-84.
(171) Ludwig DS, Pereira MA, Kroenke CH, Hilner JE, Van HL, Slattery ML, Jacobs DR, Jr. (1999) Dietary fiber, weight gain, and cardiovascular disease risk factors in young adults. JAMA 27;282(16):1539-46.
(172) Peacock JM, Folsom AR, Arnett DK, Eckfeldt JH, Szklo M. (1999) Relationship of serum and dietary magnesium to incident hypertension: the Atherosclerosis Risk in Communities (ARIC) Study. Ann Epidemiol 9(3):159-65.
(173) Appleby PN, Davey GK, Key TJ. (2002) Hypertension and blood pressure among meat eaters, fish eaters, vegetarians and vegans in EPIC-Oxford. Public Health Nutr 5(5):645-54.
(174) Azadbakht L, Mirmiran P, Esmaillzadeh A, Azizi F. (2006) Dietary diversity score and cardiovascular risk factors in Tehranian adults. Public Health Nutr 9(6):728-36.
(175) Alonso A, Beunza JJ, Bes-Rastrollo M, Pajares RM, Martinez-Gonzalez MA. (2006) Vegetable protein and fiber from cereal are inversely associated with the risk of hypertension in a Spanish cohort. Arch Med Res 37(6):778-86.

(176) Chen Y, Factor-Litvak P, Howe GR, Parvez F, Ahsan H. (2006) Nutritional influence on risk of high blood pressure in Bangladesh: A population-based cross-sectional study. American Journal of Clinical Nutrition 84(5):1224-1232.
(177) Angelopoulos PD, Milionis HJ, Moschonis G, Manios Y. (2006) Relations between obesity and hypertension: Preliminary data from a cross-sectional study in primary schoolchildren: The children study. European Journal of Clinical Nutrition 60(10):1226-1234.
(178) Kamso S, Rumawas JSP, Lukito W, Purwantyastuti. (2007) Determinants of blood pressure among Indonesian elderly individuals who are of normal and over-weight: A cross sectional study in an urban population. Asia Pacific Journal of Clinical Nutrition 16(3):546-553.
(179) Sadakane A, Tsutsumi A, Ojima T, Hashimoto A, Kajii E, Miyamoto H, Akiyoshi H, Yanagawa H, Matsuo H, Hiraoka J, Tsutsumi K, Kayaba K, et al. (2008) Dietary patterns and levels of blood pressure and serum lipids in a Japanese population. Journal of Epidemiology 18(2):58-67.
(180) Fuchs SC, Moreira LB, Camey SA, Moreira MB, Fuchs FD. (2008) Clustering of risk factors for cardiovascular disease among women in Southern Brazil: A population-based study. Cadernos de Saude Publica 24(SUPPL 2):S285-S293.
(181) Forman JP, Stampfer MJ, Curhan GC. (2009) Diet and lifestyle risk factors associated with incident hypertension in women. JAMA 302(4):401-11.
(182) Flint AJ, Hu FB, Glynn RJ, Jensen MK, Franz M, Sampson L, Rimm EB. (2009) Whole grains and incident hypertension in men. Am J Clin Nutr 90(3):493-8.
(183) Nunez-Cordoba JM, Valencia-Serrano F, Toledo E, Alonso A, Martinez-Gonzalez MA. (2009) The Mediterranean diet and incidence of hypertension: the Seguimiento Universidad de Navarra (SUN) Study. Am J Epidemiol 169(3):339-46.
(184) Toledo E, de AC-T, Alonso A, Puchau B, Zulet MA, Martinez JA, Martinez-Gonzalez MA. (2010) Hypothesis-oriented food patterns and incidence of hypertension: 6-year follow-up of the SUN (Seguimiento Universidad de Navarra) prospective cohort. Public Health Nutr 13(3):338-49.
(185) Nkondjock A, Bizome E. (2010) Dietary patterns associated with hypertension prevalence in the Cameroon defence forces. Eur J Clin Nutr 64(9):1014-21.
(186) Daniel CR, Prabhakaran D, Kapur K, Graubard BI, Devasenapathy N, Ramakrishnan L, George PS, Shetty H, Ferrucci LM, Yurgalevitch S, Chatterjee N, Reddy KS, et al. (2011) A cross-sectional investigation of regional patterns of diet and cardio-metabolic risk in India. Nutrition Journal 10(1):12.
(187) Bharati DR, Pal R, Rekha R, Yamuna TV, Kar S, Radjou AN. (2011) Ageing in Puducherry, South India: An overview of morbidity profile. Journal of Pharmacy and Bioallied Sciences 3(4):537-542.
(188) Myint PK, Luben RN, Wareham NJ, Khaw K-T. (2011) Association between plasma vitamin C concentrations and blood pressure in the European prospective investigation into cancer-norfolk population-based study. Hypertension 58(3):372-379.
(189) Myint PK, Luben RN, Wareham NJ, Khaw KT. (2011) The association between plasma vitamin C concentrations and blood pressure in 20,926 men and women: Cross sectional evidence from the EPIC-Norfolk general population-based study. Journal of Human Hypertension Conference October.
(190) Yang YJ, Kim YJ, Yang YK, Kim JY, Kwon O. (2012) Dietary flavan-3-ols intake and metabolic syndrome risk in Korean adults. Nutr Res Pract 6(1):68-77.
(191) Deedwania PC, Sharma K, Gupta A, Guptha S, Achari V, Asirvatham AJ, Bhansali A, Gupta B, Gupta S, Jali MV, Mahanta TG, Maheshwari A, et al. (2012) Association of Educational, Occupational and Socioeconomic Status with Cardiovascular Risk Factors in Asian Indians: A Cross-Sectional Study. PLoS ONE 7(8):e44098.
(192) Chan R, Chan D, Woo J. (2012) Associations between dietary patterns and demographics, lifestyle, anthropometry and blood pressure in Chinese community-dwelling older men and women. Journal of Nutritional Science 1:e20.
(193) Fonseca MJ, Gaio R, Lopes C, Santos AC. (2012) Association between dietary patterns and metabolic syndrome in a sample of portuguese adults. Nutrition Journal 11(1):64.
(194) Thawornchaisit P, de LF, Reid CM, Seubsman SA, Sleigh AC. (2013) Health risk factors and the incidence of hypertension: 4-year prospective findings from a national cohort of 60 569 Thai Open University students. BMJ Open 3(6):e002826.

(195) Umesawa M, Kitamura A, Kiyama M, Okada T, Shimizu Y, Imano H, Ohira T, Nakamura M, Maruyama K, Iso H. (2013) Association between dietary behavior and risk of hypertension among Japanese male workers. Hypertens Res 36(4):374-80.
(196) Akter S, Nanri A, Pham NM, Kurotani K, Mizoue T. (2013) Dietary patterns and metabolic syndrome in a Japanese working population. Nutrition and Metabolism 10(1):30.
(197) Sahay RD, Couch SC, Missoni S, Sujoldzic A, Novokmet N, Durakovic Z, Rao MB, Milanovic SM, Vuletic S, Deka R, Rudan P. (2013) Dietary patterns in adults from an Adriatic Island of Croatia and their associations with metabolic syndrome and its components. Collegium Antropologicum 37(2):335-342.
(198) Safdar NF, Bertone-Johnson E, Cordeiro L, Jafar T, Cohen N. (2013) Dietary patterns associated with hypertension among the low income urban population in Pakistan. FASEB Journal Conference: Experimental Biology April.
(199) Fitzpatrick A, Koju R, Gautam S, Mukamal KJ, Karmacharya BM, Yogal C, Shrestha A. (2013) Association between hypertension and dietary patterns in an urban community of nepal. American Journal of Epidemiology Conference: 46th Annual Society for Epidemiologic Research, SER Meeting Boston, MA United States Conference Publication 177 (SUPPL 11):S164.
(200) Arisawa K, Uemura H, Yamaguchi M, Nakamoto M, Hiyoshi M, Sawachika F, Katsuura-Kamano S. (2014) Associations of dietary patterns with metabolic syndrome and insulin resistance: a cross-sectional study in a Japanese population. J Med Invest 61(3-4):333-44.
(201) Saeed KM, Rasooly MH, Brown NJ. (2014) Prevalence and predictors of adult hypertension in Kabul, Afghanistan. BMC public health 14:386.
(202) George GM, Sharma KK, Ramakrishnan S, Gupta SK. (2014) A study of cardiovascular risk factors and its knowledge among school children of Delhi. Indian Heart Journal 66(3):263-271.
(203) Aljuraiban GS, Griep LMO, Chan Q, Daviglus ML, Stamler J, Van HL, Elliott P, Frost G. (2014) Relation to blood pressure of total and individual components of dietary fiber and sugar: The intermap study. Circulation Conference: American Heart Association's Epidemiology and Prevention/Nutrition, Physical Activity, and Metabolism 2014:25.
(204) Buijsse B, Jacobs DR, Jr., Steffen LM, Kromhout D, Gross MD. (2015) Plasma Ascorbic Acid, A Priori Diet Quality Score, and Incident Hypertension: A Prospective Cohort Study. PLoS One 10(12):e0144920.
(205) Al TM, Al Thani AA, Al-Chetachi W, Al MB, Khalifa SA, Bakri AH, Hwalla N, Nasreddine L, Naja F. (2015) Lifestyle Patterns Are Associated with Elevated Blood Pressure among Qatari Women of Reproductive Age: A Cross-Sectional National Study. Nutrients 7(9):7593-615.
(206) Borgi L, Curhan GC, Willett WC, Hu FB, Satija A, Forman JP. (2015) Long-term intake of animal flesh and risk of developing hypertension in three prospective cohort studies. J Hypertens 33(11):2231-8.
(207) Wong MC, Wang HH, Leung MC, Tsang CS, Lo SV, Griffiths SM. (2015) The rising prevalence of self-reported hypertension among Chinese subjects: a population-based study from 121 895 household interviews. QJM 2015 108(1):9-17.
(208) Jiang J, Liu M, Troy LM, Bangalore S, Hayes RB, Parekh N. (2015) Concordance with DASH diet and blood pressure change: results from the Framingham Offspring Study (1991-2008). Journal of Hypertension 33(11):2223-2230.
(209) Anteneh ZA, Yalew WA, Abitew DB. (2015) Prevalence and correlation of hypertension among adult population in Bahir Dar city, northwest Ethiopia: A community based cross-sectional study. International Journal of General Medicine 8:175-185.
(210) Howitt C, Hambleton IR, Rose AMC, Hennis A, Samuels TA, George KS, Unwin N. (2015) Social distribution of diabetes, hypertension and related risk factors in Barbados: A cross-sectional study. BMJ Open 5(12):e008869.
(211) Kanauchi M, Kanauchi K. (2015) Diet quality and adherence to a healthy diet in Japanese male workers with untreated hypertension. BMJ Open 5(7):e008404.
(212) Zheng PF, Shu L, Zhang XY, Si CJ, Yu XL, Gao W, Tong XQ, Zhang L. (2016) Association between Dietary Patterns and the Risk of Hypertension among Chinese: A Cross-Sectional Study. Nutrients 8(4):239.
(213) Castro MA, Baltar VT, Marchioni DM, Fisberg RM. (2016) Examining associations between dietary patterns and metabolic CVD risk factors: A novel use of structural equation modelling. British Journal of Nutrition 115 (9):1586-1597.

(214) Andreeva VA, Alles B, Feron G, Gonzalez R, Sulmont-Rosse C, Galan P, Hercberg S, Mejean C. (2016) Sex-specific sociodemographic correlates of dietary patterns in a large sample of French elderly individuals. Nutrients 8(8):484.
(215) Farhangi MA, Jahangiry L, sghari-Jafarabadi M, Najafi M. (2016) Association between dietary patterns and metabolic syndrome in a sample of Tehranian adults. Obesity Research and Clinical Practice 10 (Supplement1):S64-S73.
(216) Kini S, Kamath VG, Kulkarni MM, Kamath A, Shivalli S. (2016) Pre-hypertension among young adults (20-30 years) in coastal villages of Udupi District in southern India: An alarming scenario. PLoS ONE 11(4):e0154538.
(217) de Moraes ACF, Fernandez-Alvira JM, Rendo-Urteaga T, Julian-Almarcegui C, Beghin L, Kafatos A, Molnar D, De HS, Manios Y, Widhalm K, Pedrero-Chamizo R, Galfo M, et al. (2016) Effects of clustering of multiple lifestyle-related behaviors on blood pressure in adolescents from two observational studies. Preventive Medicine 82:111-117.
(218) Winkvist A, Klingberg S, Nilsson LM, Wennberg M, Renstrom F, Hallmans G, Boman K, Johansson I. (2017) Longitudinal 10-year changes in dietary intake and associations with cardio-metabolic risk factors in the Northern Sweden Health and Disease Study. Nutr J 16(1):20.
(219) Shang X, Scott D, Hodge A, English DR, Giles GG, Ebeling PR, Sanders KM. (2017) Dietary protein from different food sources, incident metabolic syndrome and changes in its components: An 11-year longitudinal study in healthy community-dwelling adults. Clin Nutr 36(6):1540-8.
(220) Mehrabani S, Asemi M, Najafian J, Sajjadi F, Maghroun M, Mohammadifard N. (2017) Association of Animal and Plant Proteins Intake with Hypertension in Iranian Adult Population: Isfahan Healthy Heart Program. Adv Biomed Res 6:112.
(221) Katalambula LK, Meyer DN, Ngoma T, Buza J, Mpolya E, Mtumwa AH, Petrucka P. (2017) Dietary pattern and other lifestyle factors as potential contributors to hypertension prevalence in Arusha City, Tanzania: a population-based descriptive study. BMC Public Health 17(1):659.
(222) Heleno P, Emerick L, Mourao N, Pereira D, Santos I, De Oliveira AS, Santos L, Bila W, Romano MC, Lamounier J. (2017) Systemic arterial hypertension, blood pressure levels and associated factors in schoolchildren. Revista da Associacao Medica Brasileira 63(10):869-875.
(223) El Bilbeisi AH, Hosseini S, Djafarian K. (2017) Dietary Patterns and Metabolic Syndrome among Type 2 Diabetes Patients in Gaza Strip, Palestine. Ethiopian journal of health sciences 27(3):227-238.
(224) Cohen JFW, Lehnerd ME, Houser RF, Rimm EB. (2017) Dietary Approaches to Stop Hypertension Diet, Weight Status, and Blood Pressure among Children and Adolescents: National Health and Nutrition Examination Surveys 2003-2012. Journal of the Academy of Nutrition and Dietetics 117(9):1437-1444.
(225) Howard G, Cushman M, Moy CS, Oparil S, Muntner P, Lackland DT, Manly JJ, Flaherty ML, Judd SE, Wadley VG, Long DL, Howard VJ. (2018) Association of Clinical and Social Factors With Excess Hypertension Risk in Black Compared With White US Adults. JAMA 320(13):1338-48.
(226) Sun B, Shi X, Wang T, Zhang D. (2018) Exploration of the Association between Dietary Fiber Intake and Hypertension among U.S. Adults Using 2017 American College of Cardiology/American Heart Association Blood Pressure Guidelines: NHANES 2007-2014. Nutrients 10(8):1091.
(227) Lee HA, Park H. (2018) Diet-Related Risk Factors for Incident Hypertension During an 11-Year Follow-Up: The Korean Genome Epidemiology Study. Nutrients 10(8):1077.
(228) Agodi A, Maugeri A, Kunzova S, Sochor O, Bauerova H, Kiacova N, Barchitta M, Vinciguerra M. (2018) Association of Dietary Patterns with Metabolic Syndrome: Results from the Kardiovize Brno 2030 Study. Nutrients 10(7):898.
(229) Zhang L, Sheng J, Xuan Y, Xuan P, Zhou J, Fan Y, Zhu X, Liu K, Yang L, Tao F, Wang S. (2018) Undernutrition in childhood resulted in bad dietary behaviors and the increased risk of hypertension in a middle-aged Chinese population. J Dev Orig Health Dis 9(5):544-51.
(230) Khaw KT, Sharp SJ, Finikarides L, Afzal I, Lentjes M, Luben R, Forouhi NG. (2018) Randomised trial of coconut oil, olive oil or butter on blood lipids and other cardiovascular risk factors in healthy men and women. BMJ Open 8(3):e020167.
(231) Song S, Kim J, Kim J. (2018) Gender Differences in the Association between Dietary Pattern and the Incidence of Hypertension in Middle-Aged and Older Adults. Nutrients 10(2):252.

(232) Arentoft JL, Hoppe C, Andersen EW, Overvad K, Tetens I. (2018) Associations between adherence to the Danish Food-Based Dietary Guidelines and cardiometabolic risk factors in a Danish adult population: the DIPI study. Br J Nutr 119(6):664-73.

(233) Grosso G, Stepaniak U, Micek A, Kozela M, Stefler D, Bobak M, Pajak A. (2018) Dietary polyphenol intake and risk of hypertension in the Polish arm of the HAPIEE study. Eur J Nutr 57(4):1535-44.

(234) Apidechkul T. (2018) Prevalence and factors associated with type 2 diabetes mellitus and hypertension among the hill tribe elderly populations in northern Thailand. BMC Public Health 18(1):694.

(235) Eriksen R, Gibson R, Lamb K, McMeel Y, Vergnaud A-C, Spear J, Aresu M, Chan Q, Elliott P, Frost G. (2018) Nutrient profiling and adherence to components of the UK national dietary guidelines association with metabolic risk factors for CVD and diabetes: Airwave Health Monitoring Study. British Journal of Nutrition 119(6):695-705.

(236) Al TM, Al Thani AA, Al-Chetachi W, Al MB, Khalifa SAH, Bakri AH, Hwalla N, Naja F, Nasreddine L. (2018) Adherence to the Qatar dietary guidelines: a cross-sectional study of the gaps, determinants and association with cardiometabolic risk amongst adults. BMC Public Health 18(1):503.

(237) Asaye S, Bekele S, Tolessa D, Cheneke W. (2018) Metabolic syndrome and associated factors among psychiatric patients in Jimma University Specialized Hospital, South West Ethiopia. Diabetes and Metabolic Syndrome: Clinical Research and Reviews 12 (5):753-760.

(238) de Souza Filho ZA, Ferreira AA, Dos SJ, Meira KC, Pierin AMG. Cardiovascular risk factors with an emphasis on hypertension in the Mura Indians from Amazonia. BMC Public Health 18(1):1251.

(239) Arghavani H, Daneshzad E, Namazi N, Larijani B, Askari M, Bellissimo N, Suitor K, Azadbakht L. (2019) Association of adherence to the dietary approach to stop hypertension diet and diet quality indices among women in Tehran: A cross- sectional study. Health Promot Perspect 9(4):291-8.

(240) Weber B, Bersch-Ferreira AC, Torreglosa CR, Marcadenti A, Lara ES, da Silva JT, Costa RP, Santos RHN, Berwanger O, Bosquetti R, Pagano R, Mota LGS, et al. (2019) Implementation of a Brazilian Cardioprotective Nutritional (BALANCE) Program for improvement on quality of diet and secondary prevention of cardiovascular events: A randomized, multicenter trial. Am Heart J 215:187-97.

(241) Zhang Y, Shi F, Yu Z, Yang A, Zeng M, Wang J, Yin H, Zhang B, Ma X. (2019) A cross-sectional study on factors associated with hypertension and genetic polymorphisms of renin-angiotensin-aldosterone system in Chinese hui pilgrims to hajj. BMC Public Health 19(1):1223.

(242) Ghosh S, Kumar M. (2019) Prevalence and associated risk factors of hypertension among persons aged 15-49 in India: A cross-sectional study. BMJ Open 9(12):e029714.

(243) Waskiewicz A, Zujko ME, Szczesniewska D, Tykarski A, Kwasniewska M, Drygas W, Witkowska AM. (2019) Polyphenols and dietary antioxidant potential, and their relationship with arterial hypertension: A cross-sectional study of the adult population in Poland (WOBASZ II). Advances in Clinical and Experimental Medicine 28(6):797-806.

(244) Hamro W, Modjo R. (2019) Correlation analysis of sleep duration, dietary habits, physical activity and knowledge with blood pressure on engineering workers, procurement, and construction (EPC). Indian Journal of Public Health Research and Development 10(2):549-552.

(245) Ezekwesili CN, Ononamadu CJ, Onyeukwu OF, Mefoh NC. (2019) Epidemiological survey of hypertension in Anambra state, Nigeria. Nigerian Journal of Clinical Practice 19(5):659-67.

(246) Francisco SC, Araujo LF, Griep RH, Chor D, Molina MDCB, Mil JG, Bensenor IM, Matos SMA, Barreto SM, Giatti L. (2020) Adherence to the Dietary Approaches to Stop Hypertension (DASH) and hypertension risk: results of the Longitudinal Study of Adult Health (ELSA-Brasil). Br J Nutr 123(9):1068-1077.

(247) Guo F, Zhang Q, Yin Y, Liu Y, Jiang H, Yan N, Lin J, Liu XH, Ma L. (2020) Legume consumption and risk of hypertension in a prospective cohort of Chinese men and women. Br J Nutr 123(5):564-73.

(248) Alae-Carew C, Scheelbeek P, Carrillo-Larco RM, Bernabe-Ortiz A, Checkley W, Miranda JJ. (2020) Analysis of dietary patterns and cross-sectional and longitudinal associations with hypertension, high BMI and type 2 diabetes in Peru. Public Health Nutr 23(6):1009-19.

(249) Abdurahman AA, Bule M, Azadbakhat L, Fallahyekta M, Parouhan A, Qorbani M, Dorosty AR. (2020) The association between diet quality and obesity-related metabolic risks. Hum Antibodies 28(1):1-9.
(250) Kim H, Lee K, Rebholz CM, Kim J. (2020) Plant-based diets and incident metabolic syndrome: Results from a South Korean prospective cohort study. PLoS Med 17(11):e1003371.
(251) Wisnuwardani RW, De HS, Forsner M, Gottrand F, Huybrechts I, Knaze V, Kersting M, Donne CL, Manios Y, Marcos A, Molnar D, Rothwell JA, et al. (2020) Polyphenol intake and metabolic syndrome risk in European adolescents: the HELENA study. Eur J Nutr 59(2):801-12.
(252) Ahmed FS, Wade AT, Guenther BA, Murphy KJ, Elias MF. (2020) Adherence to a Mediterranean diet associated with lower blood pressure in a US sample: Findings from the Maine-Syracuse Longitudinal Study. Journal of Clinical Hypertension 22(12):2276-2284.
(253) Nugroho A, Erniastutik. (2020) Determinants of hypertension incidence among middle-aged in Indonesia - A study of Indonesian family life survey 5 data. Pakistan Journal of Medical and Health Sciences 14(2):1512-1516.
(254) Bricarello LP, de Moura SA, de Almeida AM, Retondario A, Fernandes R, Santos de Moraes Trindade EB, Zanette Ramos Zeni LA, de Assis Guedes de Vasconcelos. (2020) Association between DASH diet (Dietary Approaches to Stop Hypertension) and hypertension in adolescents: A cross-sectional school-based study. Clinical Nutrition ESPEN 36:69-75.
(255) Alae-Carew C, Scheelbeek P, Carrillo-Larco RM, Bernabe-Ortiz A, Checkley W, Miranda JJ. (2020) Analysis of dietary patterns and cross-sectional and longitudinal associations with hypertension, high BMI and type 2 diabetes in Peru. Public health nutrition 23(6):1009-1019).
(256) Joy EJ, Green R, Agrawal S, Aleksandrowicz L, Bowen L, Kinra S, Macdiarmid JI, Haines A, Dangour AD. (2017) Dietary patterns and non-communicable disease risk in Indian adults: secondary analysis of Indian Migration Study data. Public health nutrition 20(11)1963-1972.
(257) Farmaki A-E, Rayner NW, Matchan A, Spiliopoulou P, Gilly A, Kariakli V, Kiagiadaki C, Tsafantakis E, Zeggini E, Dedoussis G. (2020) The mountainous Cretan dietary patterns and their relationship with cardiovascular risk factors: the Hellenic Isolated Cohorts MANOLIS study. Public health nutrition 20(6):1063-1074.
(258) Hajifaraji M, Haddad TS, Hussein PF, Houshiar RA, Abadi A. (2020) The association of metabolic syndrome and food patterns in non-menopause women. Annals of Nutrition and Metabolism Conference.
(259) Lee KW, Shin D. (2021) Relationships of Dietary Factors with Obesity, Hypertension, and Diabetes by Regional Type among Single-Person Households in Korea. Nutrients 13(4):1218.
(260) Ramezankhani A, Hosseini-Esfahani F, Mirmiran P, Azizi F, Hadaegh F. (2021) The association of priori and posteriori dietary patterns with the risk of incident hypertension: Tehran Lipid and Glucose Study. J Transl Med 2021 19(1):44.
(261) Kim J, Kim H, Giovannucci EL. (2021) Quality of plant-based diets and risk of hypertension: a Korean genome and examination study. European Journal of Nutrition 60(7):3841-3851.
(262) Shah S, MacDonald C-J, El FD, Mahamat-Saleh Y, Mancini FR, Fagherazzi G, Severi G, Boutron-Ruault M-C, Laouali N. (2021) The associations of the Palaeolithic diet alone and in combination with lifestyle factors with type 2 diabetes and hypertension risks in women in the E3N prospective cohort. European Journal of Nutrition 60(7):3935-3945.
(263) Amini MR, Shahinfar H, Djafari F, Sheikhhossein F, Naghshi S, Djafarian K, Clark CC, Shab-Bidar S. (2021) The association between plant-based diet indices and metabolic syndrome in Iranian older adults. Nutrition and health 27(4):435-444.
(264) Defago MD, Mozaffarian D, Irazola VE, Gutierrez L, Poggio R, Seron P, Mores N, Calandrelli M, Ponzo J, Rubinstein AL, Elorriaga N. (2021) Dietary patterns and blood pressure in Southern Cone of Latin America. Nutrition, Metabolism and Cardiovascular Diseases 31(12):3326-3334.
(265) Yao Z, Dai K, Meng G, Zhang Q, Liu L, Wu H, Gu Y, Sun S, Wang X, Jia Q, Song K, Li Z, et al. (2021) Low dietary quercetin intake by food frequency questionnaire analysis is not associated with hypertension occurrence. Clinical Nutrition 40(6):3748-3753.
(266) do Rosario, V, Schoenaker DAJM, Kent K, Weston-Green K, Charlton K. (2021) Association between flavonoid intake and risk of hypertension in two cohorts of Australian women: a longitudinal study. European Journal of Nutrition 60(5):2507-2519.

(267) Zhao J, Guo W, Wang J, Wang T. (2022) Exploring the Association of Dietary Patterns with the Risk of Hypertension Using Principal Balances Analysis and Principal Component Analysis. Public Health Nutr [Epub online ahead of print].
(268) Ballantyne JA, Coyle G, Sarwar S, Kuhn T. (2022) Fluoride Status and Cardiometabolic Health: Findings from a Representative Survey among Children and Adolescents. Nutrients 14(7):1459.
(269) Sathi NJ, Islam M, Ahmed M, Islam SMS. (2022) Prevalence, trends and associated factors of hypertension and diabetes mellitus in Bangladesh: Evidence from BHDS 2011 and 2017-18. PLoS ONE 17(5):e0267243.
(270) Dai S, Xiao X, Xu C, Jiao Y, Qin Z, Meng J, Zuo H, Zeng P, Tang D, Wu X, Qucuo N, Deji Q, et al. (2022) Association of Dietary Approaches to Stop Hypertension diet and Mediterranean diet with blood pressure in less-developed ethnic minority regions. Public Health Nutrition [Epub online ahead of print].
(271) Zhang Y, Wang Y, Chen Y, Zhou J, Xu L, Xu K, Wang N, Fu C, Liu T. (2021) Associations of dietary patterns and risk of hypertension in Southwest China: A prospective cohort study. International Journal of Environmental Research and Public Health 18(23):12378-1.
(272) Neves MEA, Souza MR, Gorgulho BM, Pereira RA, Cunha DB, Souza AM, Muraro AP, Ferreira MG, Rodrigues PRM. (2022) Restricted dietary pattern may contribute to lowering blood pressure in adolescents with obesity: Study of Cardiovascular Risk in Adolescents. Journal of Hypertension 40(4):785-793.
(273) Wang X, Zhao F, Zhao Q, Wang K, Kong S, Ma P, Huang B, Du C. (2021) Specific Types of Physical Exercises, Dietary Preferences, and Obesity Patterns With the Incidence of Hypertension: A 26-years Cohort Study. Int J Public Health 66:1604441.
(274) Shi Z, Riley M, Brown A, Page A. (2018) Chilli intake is inversely associated with hypertension among adults. Clin Nutr ESPEN 23:67-72.
(275) Hertog MG, Feskens EJ, Hollman PC, Katan MB, Kromhout D. (1993) Dietary antioxidant flavonoids and risk of coronary heart disease: the Zutphen Elderly Study. Lancet 342(8878):1007-11.
(276) Singh RB, Rastogi SS, Singh NK, Ghosh S, Gupta S, Niaz MA. (1993) Can guava fruit intake decrease blood pressure and blood lipids? J Hum Hypertens 7(1):33-8.
(277) Gillman MW, Cupples LA, Gagnon D, Posner BM, Ellison RC, Castelli WP, Wolf PA. (1995) Protective effect of fruits and vegetables on development of stroke in men. JAMA 273(14):1113-7.
(278) Liu S, Manson JE, Lee IM, Cole SR, Hennekens CH, Willett WC, Buring JE. (2000) Fruit and vegetable intake and risk of cardiovascular disease: the Women's Health Study. Am J Clin Nutr 72(4):922-8.
(279) Strandhagen E, Hansson PO, Bosaeus I, Isaksson B, Eriksson H. (2000) High fruit intake may reduce mortality among middle-aged and elderly men. The Study of Men Born in 1913. Eur J Clin Nutr 54(4):337-41.
(280) Beitz R, Mensink GB, Fischer B. (2003) Blood pressure and vitamin C and fruit and vegetable intake. Ann Nutr Metab 47(5):214-20.
(281) Dauchet L, Ferrieres J, Arveiler D, Yarnell JW, Gey F, Ducimetiere P, Ruidavets JB, Haas B, Evans A, Bingham A, Amouyel P, Dallongeville J. (2004) Frequency of fruit and vegetable consumption and coronary heart disease in France and Northern Ireland: the PRIME study. Br J Nutr 92(6):963-72.
(282) Miura K, Greenland P, Stamler J, Liu K, Daviglus ML, Nakagawa H. (2004) Relation of vegetable, fruit, and meat intake to 7-year blood pressure change in middle-aged men: the Chicago Western Electric Study. Am J Epidemiol 159(6):572-80.
(283) Moore LL, Singer MR, Bradlee ML, Djousse L, Proctor MH, Cupples LA, Ellison RC. (2005) Intake of fruits, vegetables, and dairy products in early childhood and subsequent blood pressure change. Epidemiology 16(1):4-11.
(284) Esmaillzadeh A, Kimiagar M, Mehrabi Y, Azadbakht L, Hu FB, Willett WC. (2006) Fruit and vegetable intakes, C-reactive protein, and the metabolic syndrome. Am J Clin Nutr 84(6):1489-97.
(285) Dauchet L, Czernichow S, Bertrais S, Blacher J, Galan P, Hercberg S. (2006) [Fruits and vegetables intake in the SU.VI.MAX study and systolic blood pressure change]. Arch Mal Coeur Vaiss 99(7-8):669-73.

(286) Dauchet L, Kesse-Guyot E, Czernichow S, Bertrais S, Estaquio C, Peneau S, Vergnaud AC, Chat-Yung S, Castetbon K, Deschamps V, Brindel P, Hercberg S. (2007) Dietary patterns and blood pressure change over 5-y follow-up in the SU.VI.MAX cohort. Am J Clin Nutr 85(6):1650-6.
(287) Wang YF, Yancy WS, Jr., Yu D, Champagne C, Appel LJ, Lin PH. (2008) The relationship between dietary protein intake and blood pressure: results from the PREMIER study. J Hum Hypertens 22(11):745-54.
(288) Chen Y, Lu J, Li LM, He PP, Yu CQ. (2010) [Effects of diet and physical activity factors on blood pressure in nine provinces of China: a longitudinal analysis]. Zhonghua Liu Xing Bing Xue Za Zhi 31(5):500-5.
(289) Aatola H, Koivistoinen T, Hutri-Kahonen N, Juonala M, Mikkila V, Lehtimaki T, Viikari JS, Raitakari OT, Kahonen M. (2010) Lifetime fruit and vegetable consumption and arterial pulse wave velocity in adulthood: the Cardiovascular Risk in Young Finns Study. Circulation 122(24):2521-8.
(290) Bendinelli B, Masala G, Saieva C, Salvini S, Calonico C, Sacerdote C, Agnoli C, Grioni S, Frasca G, Mattiello A, Chiodini P, Tumino R, et al. (2011) Fruit, vegetables, and olive oil and risk of coronary heart disease in Italian women: the EPICOR Study. Am J Clin Nutr 93(2):275-83.
(291) Hermsdorff HHM, Barbosa KBF, Volp ACP, Puchau B, Bressan J, Zulet MA, Martinez JA. (2012) Vitamin C and fibre consumption from fruits and vegetables improves oxidative stress markers in healthy young adults. British Journal of Nutrition 107(8):1119-1127.
(292) Larsson SC, Virtamo J, Wolk A. (2013) Total and specific fruit and vegetable consumption and risk of stroke: a prospective study. Atherosclerosis 227(1):147-52.
(293) Zarrazquin I, Torres-Unda J, Ruiz F, Irazusta J, Kortajarena M, Hoyos C, I, Gil J, Irazusta A. (2014) Longitudinal study: lifestyle and cardiovascular health in health science students. Nutr Hosp 30(5):1144-51.
(294) Shi L, Krupp D, Remer T. (2014) Salt, fruit and vegetable consumption and blood pressure development: a longitudinal investigation in healthy children. Br J Nutr 111(4):662-71.
(295) Chan Q, Stamler J, Brown IJ, Daviglus ML, Van HL, Dyer AR, Oude Griep LM, Miura K, Ueshima H, Zhao L, Nicholson JK, Holmes E, et al. (2014) Relation of raw and cooked vegetable consumption to blood pressure: the INTERMAP Study. J Hum Hypertens 28(6):353-9.
(296) Ruel G, Shi Z, Zhen S, Zuo H, Kroger E, Sirois C, Levesque JF, Taylor AW. (2014) Association between nutrition and the evolution of multimorbidity: the importance of fruits and vegetables and whole grain products. Clin Nutr 33(3):513-20.
(297) Filippidis FT, Gerovasili V, Majeed A. (2014) Association between cardiovascular risk factors and measurements of blood pressure and cholesterol in 27 European countries in 2009. Prev Med 67:71-4.
(298) Sawada K, Murayama N, Takemi Y, Ishida H. (2015) Cohort study examining the association between vegetable consumption and weight gain in a single year among Japanese employees at a manufacturing company. Asia Pac J Clin Nutr 24(4):633-8.
(299) Jiang J, Liu M, Parvez F, Wang B, Wu F, Eunus M, Bangalore S, Ahmed A, Islam T, Rakibuz-Zaman M, Hasan R, Sarwar G, et al. (2015) Association of major dietary patterns and blood pressure longitudinal change in Bangladesh. J Hypertens 33(6):1193-200.
(300) Alavi SS, Makarem J, Mehrdad R, Abbasi M. (2015) Metabolic syndrome: A common problem among office workers. International Journal of Occupational and Environmental Medicine 6(1):34-40.
(301) Morgenstern LB, Sanchez BN, Conley KM, Morgenstern MC, Sais E, Skolarus LE, Levine DA, Brown DL. (2016) The Association between Changes in Behavioral Risk Factors for Stroke and Changes in Blood Pressure. J Stroke Cerebrovasc Dis 25(9):2116-21.
(302) Du H, Li L, Bennett D, Guo Y, Key TJ, Bian Z, Sherliker P, Gao H, Chen Y, Yang L, Chen J, Wang S, et al. (2016) Fresh Fruit Consumption and Major Cardiovascular Disease in China. N Engl J Med 374(14):1332-43.
(303) Nouri F, Sarrafzadegan N, Mohammadifard N, Sadeghi M, Mansourian M. (2016) Intake of legumes and the risk of cardiovascular disease: frailty modeling of a prospective cohort study in the Iranian middle-aged and older population. Eur J Clin Nutr 70(2):217-21.
(304) Golzarand M, Bahadoran Z, Mirmiran P, Zadeh-Vakili A, Azizi F. (2016) Consumption of nitrate-containing vegetables is inversely associated with hypertension in adults: a prospective investigation from the Tehran Lipid and Glucose Study. J Nephrol 29(3):377-84.

(305) Cheng M, Wang H, Wang Z, Du W, Ouyang Y, Zhang B. (2017) Relationship between dietary factors and the number of altered metabolic syndrome components in Chinese adults: A cross-sectional study using data from the China Health and Nutrition Survey. BMJ Open 7(5):e014911.

(306) Rosario R, Santos R, Lopes L, gostinis-Sobrinho C, Moreira C, Mota J, Povoas S, Oliveira A, Padrao P, Moreira P, Abreu S. (2018) Fruit, vegetable consumption and blood pressure in healthy adolescents: A longitudinal analysis from the LabMed study. Nutr Metab Cardiovasc Dis 28(10):1075-80.

(307) Wen J, Hao J, Zhang Y, Liang Y, Li S, Wang F, Duan X, Yang X, Cao K, Wang B, Lu X, Wang N. (2018) Fresh fruit consumption and risk of incident albuminuria among rural Chinese adults: A village-based prospective cohort study. PLoS One 13(5):e0197917.

(308) Frimpong IP, Asante M, Maduforo AN. (2018) Dietary intake as a cardiovascular risk factor: a cross-sectional study of bank employees in Accra. South African Journal of Clinical Nutrition 44-50.

(309) Bonaccio M, Di CA, Costanzo S, Ruggiero E, De CA, Persichillo M, Tabolacci C, Facchiano F, Cerletti C, Donati MB, de GG, Iacoviello L. (2019) Chili Pepper Consumption and Mortality in Italian Adults. J Am Coll Cardiol 74(25):3139-49.

(310) Mazidi M, Katsiki N, George ES, Banach M. (2019) Tomato and Lycopene Consumption Is Inversely Associated with Total and Cause-Specific Mortality: A Population-based Cohort Study, on behalf of the International Lipid Expert Panel (ILEP). Br J Nutr 124(12):1303-1310.

(311) Stefler D, Malyutina S, Nikitin Y, Nikitenko T, Rodriguez-Artalejo F, Peasey A, Pikhart H, Sabia S, Bobak M. (2019) Fruit, vegetable intake and blood pressure trajectories in older age. J Hum Hypertens 33(9):671-8.

(312) Lule SA, Namara B, Akurut H, Lubyayi L, Nampijja M, Akello F, Tumusiime J, Aujo JC, Oduru G, Mentzer AJ, Smeeth L, Elliott AM, et al. (2019) Blood pressure risk factors in early adolescents: results from a Ugandan birth cohort. J Hum Hypertens 33(9):679-92.

(313) de Koning-Backus APM, Buitendijk GHS, Kiefte-de Jong JC, Colijn JM, Hofman A, Vingerling JR, Haverkort EB, Franco OH, Klaver CCW. (2019) Intake of Vegetables, Fruit, and Fish is Beneficial for Age-Related Macular Degeneration. Am J Ophthalmol 198:70-9.

(314) Kurihara A, Okamura T, Sugiyama D, Higashiyama A, Watanabe M, Okuda N, Kadota A, Miyagawa N, Fujiyoshi A, Yoshita K, Ohkubo T, Okayama A, et al. (2019) Vegetable Protein Intake was Inversely Associated with Cardiovascular Mortality in a 15-Year Follow-Up Study of the General Japanese Population. J Atheroscler Thromb 26(2):198-206.

(315) Nyanchoka AM, Van Stuijvenberg ME, Tambe AB, Mbhenyane XG. (2020) Fruit and vegetables consumption patterns and risk of chronic disease of lifestyle among university students in Kenya. Proceedings of the Nutrition Society Conference: Winter Conference Live (OCE1)

(316) Moriasi NA, Van Stuijvenberg ME, BetrandTambe A, Mbhenyane XG. (2020) Fruit and vegetable consumption patterns and risk of chronic disease of lifestyle among Kenyatta University students, in Kenya. Pediatric Diabetes Conference: 46th Annual Conference of the International Society for Pediatric and Adolescent Diabetes, ISPAD (SUPPL 29)

(317) Motuma A, Gobena T, Roba KT, Berhane Y, Worku A. (2020) Metabolic syndrome among working adults in eastern ethiopia. Diabetes, Metabolic Syndrome and Obesity: Targets and Therapy 13:4941-4951.

(318) Islam TMM, Banik PC, Barua L, Shariful Islam SM, Chowdhury S, Ahmed MSAM. (2020) Cardiovascular disease risk factors among school children of Bangladesh: A cross-sectional study. BMJ Open 10(10):e038077.

(319) Andersen E, van der Ploeg HP, Van MW, Gray CM, Mutrie N, van NF, Jelsma JGM, Anderson AS, Silva MN, Pereira HV, McConnachie A, Sattar N, et al. (2021) Contributions of changes in physical activity, sedentary time, diet and body weight to changes in cardiometabolic risk. International Journal of Behavioral Nutrition and Physical Activity 18(1):166.

(320) Kim J, Jeong K, Lee S, Seo B-N, Baek Y. (2021) Low nutritional status links to the prevalence of pre-metabolic syndrome and its cluster in metabolically high-risk Korean adults: A cross-sectional study. Medicine 100(20):e25905.

(321) Suarez R, Diaz P, Carrillo-Mayanquer I, Sarmiento Y, Cadena M, Frias-Toral E. (2022) Relationship between lifestyle habits and risk factors for cardiovascular and type 2 diabetes in staff workers at an Ecuadorian university: A cross-sectional study. Journal of Parenteral and Enteral

Nutrition Conference: American Society for Parenteral and Enteral Nutrition Conference, ASPEN (SUPPL 1):March.
(322) Mahmassani H, Avendano EE, Raman G, Johnson EJ. (2017) Avocado consumption and risk factors of heart disease: A systematic review and meta-analysis. FASEB Journal Conference: Experimental Biology (1 Supplement 1):April.
(323) Siervo M, Shannon O, Kandhari N, Prabhakar M, Fostier W, Kochl C, Rogathi J, Temu G, Stephan BCM, Gray WK, Haule I, Paddick S-M, et al. (2020) Nitrate-rich beetroot juice reduces blood pressure in tanzanian adults with elevated blood pressure: A double-blind randomized controlled feasibility trial. Journal of Nutrition 150(9):2460-2468.
(324) Li X, Wu C, Lu J, Chen B, Li Y, Yang Y, Hu S, Li J. (2020) Cardiovascular risk factors in China: a nationwide population-based cohort study. The Lancet Public Health 5(12):e672-e681.
(325) Jennings A, Koch M, Bang C, Franke A, Lieb W, Cassidy A. (2021) Microbial Diversity and Abundance of Parabacteroides Mediate the Associations between Higher Intake of Flavonoid-Rich Foods and Lower Blood Pressure. Hypertension 78(4):1016-1026.
(326) Ellis AC, Mehta T, Nagabooshanam VA, Dudenbostel T, Locher JL, Crowe-White KM. (2021) Daily 100% watermelon juice consumption and vascular function among postmenopausal women: A randomized controlled trial. Nutrition, Metabolism and Cardiovascular Diseases 31(10):2959-2968.
(327) Strom E, Johansson MA, Engvall J, Ostgren CJ, Nystrom FH, Wijkman M. (2021) Daily consumption of different fruits is associated with lower systolic home blood pressure in population-based study. Journal of Hypertension Conference: European Meeting on Hypertension and Cardiovascular Protection and International Society of Hypertension, ESH-ISH (SUPPL 1):April.
(328) Hadi A, Askarpour M, Miraghajani M, Symonds ME, Sheikhi A, Ghaedi E. (2019) Effects of strawberry supplementation on cardiovascular risk factors: a comprehensive systematic review and meta-analysis of randomized controlled trials. Food Funct 10(11):6987-98.
(329) Payab M, Kelishadi R, Qorbani M, Motlagh ME, Hasani-Ranjbar S, Ejtahed HS, Ardalan G, Arefirad T, Asayesh H, Heshmat R. (2019) Association of healthy foods intake with anthropometric measures and blood pressure in a national sample of Iranian children and adolescents: the CASPIAN-IV Study. Minerva Pediatr 71(5):420-30.
(330) Curtis PJ, Berends L, van dV, V, Jennings A, Haag L, Chandra P, Kay CD, Rimm EB, Cassidy A. (2022) Blueberry anthocyanin intake attenuates the postprandial cardiometabolic effect of an energy-dense food challenge: Results from a double blind, randomized controlled trial in metabolic syndrome participants. Clinical Nutrition 41(1):165-176.
(331) Ascherio A, Hennekens C, Willett WC, Sacks F, Rosner B, Manson J, Witteman J, Stampfer MJ. (1996) Prospective study of nutritional factors, blood pressure, and hypertension among US women. Hypertension 27(5):1065-72.
(332) Liu SS, Kim JY, Park JH, Kim S, Lee K, Bae WK, Lee KH, Han JS, Lee H, Jung SY. (2021) Fruit Intake and Changes of Cardio-Metabolic Risk Factors in People with Obesity. Korean J Fam Med 42(5):382-9.
(333) Zhang X, Ouyang Y, Huang F, Zhang J, Su C, Jia X, Du W, Li L, Bai J, Zhang B, Wang Z, Du S, et al. (2021) Modifiable factors of 20-year blood pressure trajectories among normotensives and their associations with hypertension : a prospective study. Br J Nutr [Online ahead of print]
(334) Ravn-Haren G, Dragsted LO, Buch-Andersen T, Jensen EN, Jensen RI, Nemeth-Balogh M, Paulovicsova B, Bergstrom A, Wilcks A, Licht TR, Markowski J, Bugel S. (2013) Intake of whole apples or clear apple juice has contrasting effects on plasma lipids in healthy volunteers. Eur J Nutr 52(8):1875-89.
(335) Colecraft EK, Asante M, Christian AK, du-Afarwuah S. (2018) Sociodemographic characteristics, dietary practices, and nutritional status of adults with hypertension in a semi-rural community in the eastern region of Ghana. International Journal of Hypertension 2815193.
(336) Umair M, Ahmad M, Saeed H, Saleem Z, Tauqeer F. (2020) Clinical efficacy of various anti-hypertensive regimens in hypertensive women of Punjab; A longitudinal cohort study. BMC Women's Health (1):161.
(337) He Z, Liu F-C, Li J-X, Cao J, Chen J-C, Hu D-S, Shen C, Gu S-J, Zhao Y-X, Huang J-F, Chen S-F, Lu X-F, et al. (2021) Influence of fruit and vegetable consumption on blood pressure

progression among hypertensive patients in rural China. [Chinese]. Chinese Journal of Disease Control and Prevention 25(3):276-283.
(338) Bazzano LA, Serdula MK, Liu S. (2003) Dietary intake of fruits and vegetables and risk of cardiovascular disease. Curr Atheroscler Rep 5(6):492-9.
(339) Ros E, Tapsell LC, Sabate J. (2010) Nuts and berries for heart health. Current Atherosclerosis Reports 12(6):397-406.
(340) Funtikova AN, Navarro E, Bawaked RA, Fito M, Schroder H. (2015) Impact of diet on cardiometabolic health in children and adolescents. Nutrition Journal 14 (1);118.
(341) Collese TS, Nascimento-Ferreira MV, de Moraes ACF, Rendo-Urteaga T, Bel-Serrat S, Moreno LA, Carvalho HB. (2017) Role of fruits and vegetables in adolescent cardiovascular health: a systematic review. Nutr Rev 75(5):339-49.
(342) Karasawa MMG, Mohan C. (2018) Fruits as Prospective Reserves of bioactive Compounds: A Review. Nat Prod Bioprospect 8(5):335-46.
(343) Ellwood L, Torun G, Bahar Z, Fernandez R. (2019) Effects of flavonoid rich fruits on hypertension in adults: a systematic review. JBI Database System Rev Implement Rep 17(10):2075-2105.
(344) Hasani H, Arab A, Hadi A, Pourmasoumi M, Ghavami A, Miraghajani M. (2019) Does ginger supplementation lower blood pressure? A systematic review and meta-analysis of clinical trials. Phytother Res 33(6):1639-47.
(345) Moore LL, Bradlee ML, Singer MR, Qureshi MM, Buendia JR, Daniels SR. Dietary Approaches to Stop Hypertension (DASH) eating pattern and risk of elevated blood pressure in adolescent girls. Br J Nutr. 2012;108(9):1678-85.

Supplementary table 2. Cohort studies of fruit and vegetable intake and incident hypertension

**Author, publication year, country/ region**	**Study name**	**Follow-up period**	**Study size, gender, age, number of cases**	**Dietary assess-ment**	**Exposure**	**Quantity**	**RR (95% CI)**	**Adjustment for confounders**
Steffen LM et al, 2005, USA	The Coronary Artery Risk Development in Young Adults (CARDIA) Study	1985-2000, 1986-2001, 15 years follow-up	4304 (1183 white women, 989 white men, 1249 black women, 883 black men), age 18- 45 years: 591 cases of incident hypertension and 997 cases of elevated BP	Diet history interview	Fresh, dried and canned fruit and fruit juice<br><br><br><br><br>Fresh, frozen, and canned vegetables	<0.2 times/day<br>0.2-0.5<br>0.5-0.9<br>0.9-1.5<br>>1.5<br><1.2 times/day<br>1.2-1.8<br>1.8-2.4<br>2.4-3.3<br>>3.3	1.00<br>0.88 (0.72-1.06)<br>0.83 (0.68-1.01)<br>0.85 (0.69-1.04)<br>0.75 (0.60-0.94)<br>1.00<br>0.83 (0.68-1.01)<br>0.94 (0.77-1.15)<br>0.78 (0.62-0.97)<br>0.94 (0.75-1.19)	Age, sex, race, center, education, energy intake, physical activity, alcohol intake, smoking, vitamin supplement use
Nunez-Cordoba JM et al, 2008, Spain	Seguimiento University of Navarra cohort (SUN)	1999-2006, 4.1 years follow-up	8594 participants (5338 women, 3256 men), age 20-95 years: 426 cases	Validated semiquant itative FFQ, 136 items	Vegetables<br><br><br><br><br>Fruit<br><br><br><br><br>Fruit and vegetables	≤1.0 serv./day<br>1.1-2.0<br>2.1-3.0<br>3.1-4.0<br>>4.0<br>≤1.0 serv./day<br>1.1-2.0<br>2.1-3.0<br>3.1-4.0<br>>4.0<br>≤2.0 serv./day<br>2.1-4.0<br>4.1-4.9<br>≥5.0	1.00<br>1.00 (0.74-1.34)<br>0.92 (0.66-1.27)<br>1.03 (0.70-1.52)<br>0.87 (0.55-1.39)<br>1.00<br>0.86 (0.66-1.13)<br>0.94 (0.70-1.27)<br>1.02 (0.72-1.45)<br>0.85 (0.59-1.22)<br>1.00<br>0.86 (0.63-1.16)<br>0.86 (0.60-1.24)<br>0.78 (0.55-1.10)	Age, gender, total energy intake, BMI, physical activity, alcohol, family history of hypertension, sodium intake, low-fat dairy intake, whole grains intake, fish intake and smoking
Camoes M et al, 2010, Portugal	EPIPorto study	1999-2008, 3.8 years follow-up	549 participants, age >40 years: 160 cases	Validated semi-quantitativ e FFQ 82 items	Fruits, vegetables and pulses	<388.9/<332.9 g/d (w/m)<br>388.9/332.9-548.5/510.1<br>>548.5/>510.1	1.00<br>0.83 (0.57-1.21)<br>0.61 (0.40-0.93)	Age, sex, education, BMI, physical activity level and baseline total energy intake
Tsubota-Utsugi M et	The Ohasama study	1998-NA, 4.1 years follow-	745 participants, age	Validated 141-item	Fruit	≤38.40 g/day<br>38.41-63.80	1.00<br>0.64 (0.36-1.15)	Age, sex, BMI, frequency of exercise, smoking status,

al, 2011,		up	<35 years: 222	FFQ		63.8–100.02	0.70 (0.39-1.26)	alcohol consumption, energy-
Japan			cases			≥100.03 g/day	0.40 (0.21-0.74)	adjusted fat and sodium
					Vegetables	≤143.41 g/day	1.00	consumption, total energy,
						143.42–211.55	0.96 (0.52-1.75)	baseline systolic home BP,
						211.56–282.75	1.11 (0.60-2.05)	and a past history of diabetes,
						≥282.76	0.75 (0.40-1.38)	hypercholesterolaemia and
								cardiovascular disease
Wang L et al,	Women's Health	1992-2007,	28082 women,	131-item	Total fruits and vegetables	<2 serv./day	1.00	Age, race, total energy intake,
2011, USA	Study (WHS)	12.9 years	age 39-89 years:	validated		2-<4	1.03 (0.95-1.12)	and randomized treatment,
		follow-up	13633 cases	semiquant		4-<6	1.02 (0.94-1.11)	smoking, daily alcohol intake,
				itative		6-<8	1.04 (0.95-1.14)	exercise, BMI,
				FFQ		≥8	1.03 (0.93-1.13)	postmenopausal status,
					Total fruits	<0.5 serv./day	1.00	postmenopausal hormone use,
						0.5-<1.5	0.99 (0.92-1.06)	multivitamin supplement use,
						1.5-<2	0.98 (0.90-1.06)	history of diabetes, history of
						2-<3	0.98 (0.91-1.06)	hypercholesterolemia, intake
						≥3	0.95 (0.88-1.04)	of whole grains, red meats,
					Total vegetables	<1.5 serv./day	1.00	low-fat dairy products, and
						1.5-<2.5	0.95 (0.89-1.01)	nuts, for fruit intake also
						2.5-<4	0.99 (0.93-1.05)	adjusted for vegetable intake
						4-<5	0.99 (0.92-1.07)	and vice versa
						≥5	0.98 (0.91-1.06)	
					Apples	None/rarely	1.00	
						1–3 serv./month	0.95 (0.89-1.02)	
						1 serv./week	0.93 (0.86-0.99)	
						2–4	0.90 (0.84-0.96)	
						>4	0.91 (0.85-0.99)	
					Bananas	None/rarely	1.00	
						1–3 serv./month	1.02 (0.96-1.09)	
						1 serv./week	1.02 (0.96-1.10)	
						2–4	1.02 (0.96-1.09)	
						>4	1.02 (0.95-1.10	

					Oranges	None/rarely	1.00	
						1–3 serv./month	0.98 (0.94-1.03)	
						1	0.96 (0.90-1.01)	
						2–4	0.95 (0.90-1.00)	
						>4	0.91 (0.85-0.98)	
					Strawberries	None/rarely	1.00	
						1–3 serv./month	1.00 (0.96-1.04)	
						1 serv./week	1.03 (0.97-1.08)	
						> 1	1.07 (0.99-1.15)	
					Blueberries	None/rarely	1.00	
						1–3 serv./month	0.97 (0.93-1.01)	
						≥1	1.02 (0.96-1.08)	
					Green-leafy vegetables	<0.2 serv./day	1.00	
						0.2-<0.4	0.99 (0.93-1.05)	
						0.4-<0.6	0.98 (0.93-1.04)	
						0.6-<1.0	0.96 (0.90-1.02)	
						≥1.0	0.94 (0.88-1.01)	
					Cruciferous vegetables	<0.2 serv./day	1.00	
						0.2-<0.4	1.06 (1.01-1.12)	
						0.4-<0.6	1.05 (0.99-1.11)	
						0.6-<1.0	1.09 (1.02-1.16)	
						≥1.0	1.14 (1.06-1.23)	
					Dark-yellow vegetables	<0.2 serv./day	1.00	
						0.2-<0.4	0.96 (0.91-1.01)	
						0.4-<0.6	0.99 (0.93-1.05)	
						0.6-<1.0	0.93 (0.87-0.99)	
						≥1.0	0.88 (0.82-0.95)	
					Onions	<0.1 serv./day	1.00	
						0.1-<0.3	1.06 (1.01-1.11)	
						0.3-<0.5	1.04 (0.99-1.10)	
						0.5-<1.0	1.09 (1.03-1.15)	
						≥1.0	1.07 (1.00-1.15)	
					Tomatoes	<0.1 serv./day	1.00	
						0.1-<0.3	1.02 (0.94-1.11)	
						0.3-<0.5	1.01 (0.93-1.11)	
						0.5-<1.0	1.02 (0.93-1.11)	
						≥1.0	1.05 (0.95-1.16)	

Weng LC et al, 2013, USA	Atherosclerosis Risk in Communities Study	1987-1989 - 1996-1998, 9 years follow-up	9913 men and women, age 45-64 years: 2853 hypertension cases	Validated FFQ, 66 food items	Fruits<br><br><br><br><br>Vegetables	1<br>2<br>3<br>4<br>5<br>1<br>2<br>3<br>4<br>5	1.00<br>1.06 (0.94-1.19)<br>0.98 (0.87-1.10)<br>1.08 (0.96-1.22)<br>1.06 (0.93-1.20)<br>1.00<br>0.97 (0.86-1.09)<br>0.94 (0.83-1.06)<br>0.97 (0.85-1.09)<br>0.96 (0.84-1.09)	Age, sex, race, education, center, energy intake, added salt, physical activity, smoking, BMI, BMI change, diabetes mellitus
Golzarand M et al, 2015, Iran	Tehran Lipid and Glucose Study	2006-2009, 2008-2011, 3 years follow-up	1546 participants, 20-70 years: 265 cases	Validated semiquantitative FFQ,168-item	Fruit<br><br><br><br>Vegetables	191 g/d<br>312<br>420<br>548<br>220 g/d<br>275<br>311<br>350	1.00<br>0.86 (0.57-1.30)<br>0.93 (0.60-1.44)<br>0.86 (0.48-1.54)<br>1.00<br>0.88 (0.59-1.33)<br>0.88 (0.57-1.35)<br>0.68 (0.44-1.15)	Age, sex, smoking status, physical activity, socioeconomic status, BMI, energy intake, total fiber, sodium, potassium, vitamins A, C and E
Borgi L et al, 2016, USA	Nurses' Health Study (NHS)	1984-2010, 26 years follow-up	62175 women, age 30-55 years: 35375 cases	Validated semiquantitative FFQ, >130 items	Total fruit<br><br><br><br><br>Total vegetables<br><br><br><br><br>Total fruit and vegetables<br><br><br><br>Raisins or grapes<br><br><br><br>Apples or pears	≤4 serv./week<br>5-6<br>1 serv./day<br>2-3<br>≥4<br>≤4 serv./ week<br>5-6<br>1 serv./day<br>2-3<br>≥4<br>≤1 serv./day<br>2-3<br>4-5<br>≥6<br><1 serv./month<br>1-3<br>1-3 serv./week<br>≥4<br><1 serv./month<br>1-3<br>1-3 serv./week	1.00<br>0.97 (0.93-1.01)<br>0.95 (0.92-0.99)<br>0.94 (0.91-0.98)<br>0.96 (0.88-1.03)<br>1.00<br>0.95 (0.77-1.16)<br>0.90 (0.76-1.07)<br>0.89 (0.75-1.05)<br>0.87 (0.73-1.03)<br>1.00<br>0.90 (0.84-0.95)<br>0.88 (0.83-0.94)<br>0.85 (0.80-0.91)<br>1.00<br>0.97 (0.94-0.99)<br>0.93 (0.90-0.96)<br>0.92 (0.86-0.97)<br>1.00<br>0.94 (0.89-0.99)<br>0.93 (0.88-0.99)	Age, race/ethnicity, body mass index, current smoking status, physical activity, weight change per food frequency questionnaire cycle, menopausal status, alcohol intake, analgesic use (nonsteroidal anti-inflammatory drugs, acetaminophen, aspirin), family history of hypertension, total energy intake, animal flesh intake (combination of processed and unprocessed red meat, poultry and seafood), whole grains, sugar-sweetened beverage intake, artificially sweetened diet beverage intake

						≥4	0.90 (0.85-0.96)	
					Strawberries	<1 serv./month	1.00	
						1-3	0.96 (0.93-0.99)	
						1-3 serv./week	0.96 (0.92-0.99)	
						≥4	0.99 (0.91-1.09)	
					Blueberries	<1 serv./month	1.00	
						1-3	0.98 (0.96 -1.00)	
						1-3 serv./week	0.94 (0.89 -0.98)	
						≥4	0.89 (0.75 -1.07)	
					Avocado	<1 serv./month	1.00	
						1-3	0.98 (0.95-1.02)	
						1-3 serv./week	0.85 (0.76-0.95)	
						≥4	0.99 (0.64-1.54)	
					Broccoli	<1 serv./month	1.00	
						1-3	1.03 (0.97 -1.09)	
						1-3 serv./week	1.00 (0.94 -1.07)	
						≥4	0.95 (0.88 -1.03)	
					Cauliflower	<1 serv./month	1.00	
						1-3	1.01 (0.98 -1.05)	
						1-3 serv./week	1.06 (1.01 -1.10)	
						≥4	1.10 (0.99 -1.22)	
					Brussel Sprouts	<1 serv./month	1.00	
						1-3	1.02 (1.00 -1.05)	
						1-3 serv./week	1.07 (1.02 -1.13)	
						≥4	1.31 (0.98 -1.75)	
					Carrots	<1 serv./month	1.00	
						1-3	1.01 (0.94 -1.08)	
						1-3 serv./week	0.98 (0.91 -1.05)	
						≥4	0.97 (0.90 -1.04)	
					Cantaloupe	<1 serv./month	1.00	
						1-3	1.02 (0.98-1.05)	
						1-3 serv./week	1.06 (1.02-1.10)	
						≥4	1.10 (1.03-1.18)	
					Corn	<1 serv./month	1.00	
						1-3	1.02 (0.98-1.06)	
						1-3 serv./week	1.03 (0.98-1.07)	
						≥4	0.93 (0.82-1.05)	
					Yams or sweet potatoes	<1 serv./month	1.00	
						1-3	1.01 (0.99-1.03)	
						1-3 serv./week	1.01 (0.95-1.07)	

						≥4	0.88 (0.62-1.24)	
					Lettuce	<1 serv./month	1.00	
						1-3	1.07 (0.92-1.24)	
						1-3 serv./week	1.05 (0.91-1.21)	
						≥4	1.03 (0.89-1.19)	
					Cabbage	<1 serv./month	1.00	
						1-3	1.00 (0.97-1.04)	
						1-3 serv./week	1.02 (0.98-1.06)	
						≥4	1.01 (0.90-1.14)	
					Eggplant or zucchini	<1 serv./month	1.00	
						1-3	0.98 (0.95-1.01)	
						1-3 serv./week	0.99 (0.96-1.03)	
						≥4	1.00 (0.94-1.07)	
					Green pepper	<1 serv./month	1.00	
						1-3	1.02 (0.99-1.05)	
						1-3 serv./week	1.02 (0.98-1.05)	
						≥4	0.98 (0.92-1.04)	
					Tomatoes	<1 serv./month	1.00	
						1-3	0.97 (0.90-1.05)	
						1-3 serv./week	1.01 (0.93-1.08)	
						≥4	1.00 (0.93-1.08)	
					Onions	<1 serv./month	1.00	
						1-3	0.98 (0.96-1.01)	
						1-3 serv./week	0.97 (0.93-1.01)	
						≥4	0.92 (0.84-1.01)	

Borgi L et al, 2016, USA	Nurses' Health Study II (NHS ll)	1991-2011, 20 years follow-up	88475 women, age 25-42 years: 25246 cases	Validated semiquantitative FFQ, >130 items	Total fruit	≤4 serv./week	1.00	Age, race/ethnicity, body mass index, current smoking status, physical activity, weight change per food frequency questionnaire cycle, menopausal status, alcohol intake, current oral contraceptive use, analgesic use (nonsteroidal anti-inflammatory drugs, acetaminophen, aspirin), family history of hypertension, total energy intake, animal flesh intake (combination of processed and unprocessed red meat, poultry and seafood), whole grains, sugar-sweetened beverage intake, artificially sweetened diet beverage intake.
						5-6	1.03 (0.99-1.07)	
						1 serv./day	0.97 (0.94-1.01)	
						2-3	0.91 (0.87-0.95)	
						≥4	0.91 (0.81-1.02)	
					Total vegetables	≤4 serv./ week	1.00	
						5-6	1.01 (0.86-1.18)	
						1 serv./day	0.96 (0.84-1.09)	
						2-3	0.97 (0.85-1.11)	
						≥4	1.01 (0.88-1.15)	
					Total fruit and vegetables	≤1 serv./day	1.00	
						2-3	0.97 (0.92-1.03)	
						4-5	0.95 (0.89-1.00)	
						≥6	0.94 (0.88-0.99)	
					Raisins or grapes	<1 serv./month	1.00	
						1-3	0.99 (0.96-1.02)	
						1-3 serv./week	0.94 (0.91-0.98)	
						≥4	0.82 (0.75-0.90)	
					Apples or pears	<1 serv./month	1.00	
						1-3	0.96 (0.91-1.02)	
						1-3 serv./week	0.95 (0.90-1.01)	
						≥4	0.93 (0.87-0.99)	
					Strawberries	<1 serv./month	1.00	
						1-3	1.03 (0.99 -1.07)	
						1-3 serv./week	1.02 (0.98 -1.07)	
						≥4	1.02 (0.93 -1.12)	
					Blueberries	<1 serv./month	1.00	
						1-3	0.96 (0.93 -0.99)	
						1-3 serv./week	0.95 (0.90 -0.99)	
						≥4	0.90 (0.76 -1.07)	
					Avocado	<1 serv./month	1.00	
						1-3	0.95 (0.92-0.98)	
						1-3 serv./week	0.91 (0.84-0.99)	
						≥4	0.77 (0.49-1.19)	
					Broccoli	<1 serv./month	1.00	
						1-3	0.98 (0.92 -1.04)	
						1-3 serv./week	0.95 (0.89 -1.01)	
						≥4	0.94 (0.86 -1.02)	

					Cauliflower	<1 serv./month	1.00	
						1-3	0.99 (0.96 -1.02)	
						1-3 serv./week	1.06 (1.02 -1.11)	
						≥4	1.04 (0.92 -1.17)	
					Brussel Sprouts	<1 serv./month	1.00	
						1-3	1.01 (0.98 -1.04)	
						1-3 serv./week	1.03 (0.97 -1.10)	
						≥4	0.98 (0.73 -1.30)	
					Carrots	<1 serv./month	1.00	
						1-3	0.98 (0.92 -1.06)	
						1-3 serv./week	0.96 (0.89 -1.03)	
						≥4	0.94 (0.87 -1.01)	
					Cantaloupe	<1 serv./month	1.00	
						1-3	1.03 (0.99-1.06)	
						1-3 serv./week	1.04 (1.00-1.09)	
						≥4	0.93 (0.80-1.08)	
					Corn	<1 serv./month	1.00	
						1-3	1.07 (1.01-1.14)	
						1-3 serv./week	1.09 (1.03-1.16)	
						≥4	1.12 (1.01-1.23)	
					Yams or sweet potatoes	<1 serv./month	1.00	
						1-3	1.02 (0.99-1.05)	
						1-3 serv./week	1.11 (1.04-1.18)	
						≥4	0.99 (0.70-1.41)	
					Lettuce	<1 serv./month	1.00	
						1-3	1.02 (0.89-1.17)	
						1-3 serv./week	0.99 (0.87-1.12)	
						≥4	0.99 (0.87-1.13)	
					Cabbage	<1 serv./month	1.00	
						1-3	1.02 (0.99-1.05)	
						1-3 serv./week	1.03 (0.98-1.08)	
						≥4	1.03 (0.88-1.20)	
					Eggplant or zucchini	<1 serv./month	1.00	
						1-3	0.97 (0.94-1.00)	
						1-3 serv./week	0.95 (0.91-0.99)	
						≥4	0.97 (0.89-1.06)	
					Green pepper	<1 serv./month	1.00	
						1-3	1.02 (0.98-1.05)	
						1-3 serv./week	1.01 (0.97-1.05)	
						≥4	0.99 (0.93-1.05)	

					Tomatoes	<1 serv./month	1.00	
						1-3	0.99 (0.93-1.04)	
						1-3 serv./week	1.04 (0.98-1.09)	
						≥4	1.03 (0.97-1.10)	
					Onions	<1 serv./month	1.00	
						1-3	1.01 (0.98-1.04)	
						1-3 serv./week	0.98 (0.95-1.02)	
						≥4	0.99 (0.93-1.06)	
Borgi L et al, 2016, USA	Health Professionals Follow-up Study (HPFS)	1986-2010, 24 years follow-up	36803 men, age 40-75 years: 16752 cases	Validated semiquantitative FFQ, >130 items	Total fruit	≤4 serv./week	1.00	Age, race/ethnicity, body mass index, current smoking status, physical activity, weight change per food frequency questionnaire cycle, alcohol intake, analgesic use (nonsteroidal anti-inflammatory drugs, acetaminophen, aspirin), family history of hypertension, total energy intake, animal flesh intake (combination of processed and unprocessed red meat, poultry and seafood), whole grains, sugar-sweetened beverage intake, artificially sweetened diet beverage intake.
						5-6	0.95 (0.89-1.00)	
						1 serv./day	0.92 (0.88-0.97)	
						2-3	0.92 (0.87-0.97)	
						≥4	0.88 (0.81-0.97)	
					Total vegetables	≤4 serv./ week	1.00	
						5-6	0.96 (0.77-1.19)	
						1 serv./day	0.92 (0.77-1.11)	
						2-3	0.94 (0.79-1.13)	
						≥4	0.93 (0.78-1.12)	
					Total fruit and vegetables	≤1 serv./day	1.00	
						2-3	0.93 (0.85-1.01)	
						4-5	0.91 (0.83-0.98)	
						≥6	0.89 (0.81-0.97)	
					Raisins or grapes	<1 serv./month	1.00	
						1-3	0.99 (0.96-1.04)	
						1-3 serv./week	0.98 (0.93-1.02)	
						≥4	1.00 (0.93-1.07)	
					Apples or pears	<1 serv./month	1.00	
						1-3	0.97 (0.91-1.04)	
						1-3 serv./week	0.96 (0.89-1.03)	
						≥4	0.93 (0.86-1.00)	
					Strawberries	<1 serv./month	1.00	
						1-3	0.98 (0.94 -1.02)	
						1-3 serv./week	1.03 (0.97 -1.09)	
						≥4	1.00 (0.85 -1.18)	
					Blueberries	<1 serv./month	1.00	
						1-3	0.95 (0.92 -0.99)	
						1-3 serv./week	0.97 (0.90 -1.05)	
						≥4	1.03 (0.81 -1.30)	
					Avocado	<1 serv./month	1.00	
						1-3	0.99 (0.95-1.02)	

						1-3 serv./week	0.95 (0.88-1.02)	
						≥4	0.98 (0.76-1.26)	
					Broccoli	<1 serv./month	1.00	
						1-3	0.96 (0.90 -1.03)	
						1-3 serv./week	0.95 (0.89 -1.02)	
						≥4	0.94 (0.84 -1.05)	
					Cauliflower	<1 serv./month	1.00	
						1-3	0.99 (0.95 -1.03)	
						1-3 serv./week	0.97 (0.92 -1.03)	
						≥4	1.02 (0.88 -1.19)	
					Brussel Sprouts	<1 serv./month	1.00	
						1-3	1.03 (0.99 -1.06)	
						1-3 serv./week	0.99 (0.93 -1.06)	
						≥4	1.46 (1.10 -1.93)	
					Carrots	<1 serv./month	1.00	
						1-3	0.97 (0.90 -1.06)	
						1-3 serv./week	0.96 (0.89 -1.05)	
						≥4	0.94 (0.86 -1.03)	
					Cantaloupe	<1 serv./month	1.00	
						1-3	1.05 (1.01-1.10)	
						1-3 serv./week	1.08 (1.02-1.14)	
						≥4	1.08 (0.97-1.19)	
					Corn	<1 serv./month	1.00	
						1-3	0.97 (0.91-1.02)	
						1-3 serv./week	0.99 (0.93-1.06)	
						≥4	1.09 (0.95-1.25)	
					Yams or sweet potatoes	<1 serv./month	1.00	
						1-3	1.00 (0.97-1.04)	
						1-3 serv./week	0.99 (0.93-1.07)	
						≥4	0.77 (0.56-1.05)	
					Lettuce	<1 serv./month	1.00	
						1-3	0.89 (0.78-1.01)	
						1-3 serv./week	0.86 (0.76-0.97)	
						≥4	0.85 (0.76-0.96)	
					Cabbage	<1 serv./month	1.00	
						1-3	1.00 (0.96-1.05)	
						1-3 serv./week	1.00 (0.95-1.05)	
						≥4	1.15 (1.00-1.31)	
					Eggplant or zucchini	<1 serv./month	1.00	
						1-3	0.99 (0.95-1.03)	

						1-3 serv./week<br>≥4	0.99 (0.94-1.04)<br>0.99 (0.90-1.08)	
					Green pepper	<1 serv./month<br>1-3<br>1-3 serv./week<br>≥4	1.00<br>0.99 (0.96-1.04)<br>1.02 (0.97-1.06)<br>1.04 (0.96-1.04)	
					Tomatoes	<1 serv./month<br>1-3<br>1-3 serv./week<br>≥4	1.00<br>0.97 (0.88-1.06)<br>0.95 (0.87-1.04)<br>0.96 (0.87-1.06)	
					Onions	<1 serv./month<br>1-3<br>1-3 serv./week<br>≥4	1.00<br>1.03 (0.99-1.07)<br>1.03 (0.97-1.09)<br>0.94 (0.82-1.07)	
Borgi L et al, 2016, USA	NHS	1984-2012, 28 years follow-up	62175 women, age 30-55 years: 35728 cases	Validated semiquant itative FFQ	Potatoes (baked, boiled or mashed)	≤1 serv./month<br>1-3<br>1-3 serv./week<br>≥4	1.00<br>1.07 (0.97-1.19)<br>1.12 (1.01-1.24)<br>1.13 (1.02-1.26)	Age, race/ethnicity (white, African-American, Asian, Hispanic, other), BMI, current smoking status, physical activity, weight change per FFQ cycle, menopausal status, alcohol intake, analgesic use (non-steroidal anti-inflammatory drugs, acetaminophen (paracetamol, aspirin), family history of hypertension, total energy intake, animal flesh intake (combination of processed and unprocessed red meat, poultry, and seafood), whole grains, sugar sweetened drink intake, artificially sweetened diet drink intake, total fruit, total vegetables
Borgi L et al, 2016, USA	NHS ll	1991-2011, 20 years follow-up	88475 women, age 25-42 years: 25246 cases	Validated semiquant itative FFQ	Potatoes (baked, boiled or mashed)	≤1 serv./month<br>1-3<br>1-3 serv./week<br>≥4	1.00<br>1.08 (0.97-1.21)<br>1.17 (1.04-1.31)<br>1.25 (1.11-1.41)	Age, race/ethnicity (white, African-American, Asian, Hispanic, other), BMI, current smoking status, physical activity, weight change per

								FFQ cycle, menopausal status, alcohol intake, current oral contraceptive use, analgesic use (non-steroidal anti-inflammatory drugs, acetaminophen (paracetamol, aspirin), family history of hypertension, total energy intake, animal flesh intake (combination of processed and unprocessed red meat, poultry, and seafood), whole grains, sugar sweetened drink intake, artificially sweetened diet drink intake, total fruit, total vegetables
Borgi L et al, 2016, USA	HPFS	1986-2012, 26 years follow-up	36803 men, age 40-75 years: 16752 cases	Validated semiquantitative FFQ	Potatoes (baked, boiled or mashed)	≤1 serv./month<br>1-3<br>1-3 serv./week<br>≥4	1.00<br>0.92 (0.82-1.04)<br>0.95 (0.84-1.07)<br>0.95 (0.84-1.08)	Age, race/ethnicity (white, African-American, Asian, Hispanic, other), BMI, current smoking status, physical activity, weight change per FFQ cycle, alcohol intake, analgesic use (non-steroidal anti-inflammatory drugs, acetaminophen (paracetamol, aspirin), family history of hypertension, total energy intake, animal flesh intake (combination of processed and unprocessed red meat, poultry, and seafood), whole grains, sugar sweetened drink intake, artificially sweetened diet drink intake, total fruit, total vegetables

Auerbach BJ et al, 2017, USA	Women's Health Initiative (WHI)	1993-2005, 12 years follow-up	80539 women, age 50–79 years: 36314 cases	Semiquantitative food frequency questionnaire (FFQ)	100% Fruit Juice<br><br><br><br><br>Whole Fruit	≤4 serv./per week<br>5-6<br>1 serv./per day<br>2-3<br>≥4<br>≤4 serv./per week<br>5-6<br>1 serv./per day<br>2-3<br>≥4	1.0<br>0.99 (0.97-1.01)<br>1.02 (0.99-1.04)<br>1.04 (0.99-1.10)<br>1.29 (1.06-1.56)<br>1.0<br>1.02 (1.00-1.04)<br>1.01 (0.98-1.05)<br>1.02 (0.98-1.05)<br>0.94 (0.83-1.05)	Age, education level, race/ethnicity, smoking status, physical activity, body mass index (BMI), hormone replacement therapy status, study arm, and total energy intake
Bahadoran Z et al, 2017, Iran	Tehran Lipid and Glucose Study (TLGS)	2006-2008 and 2012-2014, 6 years follow-up	3042 women and men, age ≥19 years: 291 cases	Validated semiquantitative FFQ, 168 items	Allium vegetables	1.0 g/week<br>10<br>39	1.00<br>0.90 (0.67-1.21)<br>0.74 (0.54-1.00)	Age, sex, BMI, smoking, type 2 diabetes, TGs to HDL-C ratio, physical activity, dietary pattern scores
Hu AE et al, 2017, Spain	Seguimiento Universidad de Navarra (SUN cohort)	1999-2003, 6.7 years follow-up	13837 participants, mean age 35.1/42.7 years (men/women): 1111 cases	Validated semiquantitative FFQ with 136 items	Potatoes	11.4 g/d<br>30.1<br>45.3<br>68.1<br>108.7	1.00<br>0.93 (0.76-1.14)<br>0.90 (0.73-1.11)<br>0.95 (0.78-1.16)<br>0.98 (0.80-1.19)	Age, sex, and recruitment period, BMI; family history of hypertension; physical activity; smoking; education; television watching; total energy intake; sodium intake; and consumption of fast food, low-fat dairy products, fruits, vegetables, tree nuts, fried foods, alcohol, olive oil, and sugar-sweetened beverages, total energy intake

Kim J, 2017, Korea	Korean Genome and Epidemiology Study (KoGES)	2001-2009, and 2002-2010, 8 years follow-up	4257 participants (2172 women, 2085 men), age 40-69 years: 1158 cases (552 women and 606 men)	Validated semiquantitative FFQ, 103 items	Total fruit (women)	Never or rarely<br>1-<2 serv./day<br>2-<4<br>≥4	1.00<br>0.71 (0.54-0.95)<br>0.44 (0.33-0.58)<br>0.33 (0.24-0.45)	Age, BMI, residential location, household income, education level, alcohol intake, smoking status, and physical activity, intakes of total energy, sodium, vegetables, meat, grains, and dairy foods
					Total fruit (men)	Never or rarely<br>1-<2 serv./day<br>2-<4<br>≥4	1.00<br>0.58 (0.45-0.75)<br>0.44 (0.34-0.57)<br>0.44 (0.32-0.60)	
					Total vegetables (women)	Never or rarely<br>1-<2 serv./day<br>2-<4<br>≥4	1.00<br>0.92 (0.61-1.39)<br>1.05 (0.71-1.56)<br>1.22 (0.80-1.85)	
					Total vegetables (men)	Never or rarely<br>1-<2 serv./day<br>2-<4<br>≥4	1.00<br>1.00 (0.66-1.51)<br>1.01 (0.67-1.51)<br>1.19 (0.77-1.85)	
					Citrus fruit (women)	Never or rarely<br>1-<2 serv./day<br>≥2	1.00<br>1.04 (0.83-1.32)<br>0.79 (0.48-1.28)	
					Citrus fruit (men)	Never or rarely<br>1-<2 serv./day<br>≥2	1.00<br>1.06 (0.83-1.35)<br>1.46 (0.85-2.50)	
					Noncitrus fruit (women)	Never or rarely<br>1-<2 serv./day<br>2-4<br>≥4	1.00<br>1.06 (0.74-1.51)<br>1.01 (0.72-1.41)<br>0.94 (0.67-1.31)	
					Noncitrus fruit (men)	Never or rarely<br>1-<2 serv./day<br>2-4<br>≥4	1.00<br>1.31 (0.94-1.83)<br>1.36 (0.99-1.87)<br>1.23 (0.88-1.72)	
					Carotene-rich fruit (women)	Never or rarely<br>1-<2 serv./day<br>2-4<br>≥4	1.00<br>1.01 (0.75-1.35)<br>0.98 (0.75-1.30)<br>0.93 (0.67-1.29)	
					Carotene-rich fruit (men)	Never or rarely<br>1-<2 serv./day<br>2-4<br>≥4	1.00<br>1.06 (0.82-1.37)<br>1.11 (0.85-1.43)<br>1.12 (0.81-1.56)	
					Cruciferous vegetables (women)	<3-4 serv./week<br>3-4 serv./week-<1serv./day	1.00<br>1.20 (0.89-1.62)	

						≥1 serv./day	0.66 (0.37-1.15)	
					Cruciferous vegetables (men)	<3-4 serv./week	1.00	
						3-4 serv./week-<1serv./day	1.07 (0.83-1.39)	
						≥1 serv./day	1.13 (0.74-1.74)	
					Carotene-rich vegetables (women)	<3-4 serv./week	1.00	
						3-4 serv./week-<1serv./day	1.07 (0.77-1.48)	
						≥1 serv./day	0.85 (0.50-1.44)	
					Carotene-rich vegetables (men)	<3-4 serv./week	1.00	
						3-4 serv./week-<1serv./day	1.22 (0.91-1.64)	
						≥1 serv./day	1.03 (0.57-1.87)	
					Mushrooms (women)	<3-4 serv./week	1.00	
						3-4 serv./week-<1serv./day	1.03 (0.74-1.44)	
						≥1 serv./day	0.73 (0.38-1.39)	
					Mushrooms (men)	<3-4 serv./week	1.00	
						3-4 serv./week-<1serv./day	1.33 (1.01-1.75)	
						≥1 serv./day	0.96 (0.51-1.79)	
					Green leafy vegetables (women)	Never or rarely	1.00	
						1-<2 serv./day	0.96 (0.74-1.24)	
						≥2 serv./day	1.42 (0.95-2.12)	
					Green leafy vegetables (men)	Never or rarely	1.00	
						1-<2 serv./day	1.11 (0.89-1.40)	
						≥2 serv./day	1.14 (0.72-1.81)	
					Other vegetables (women)	Never or rarely	1.00	
						1-<2 serv./day	1.08 (0.84-1.41)	
						2-≥4	1.00 (0.73-1.36)	
						≥4	1.14 (0.65-1.97)	
					Other vegetables (men)	Never or rarely	1.00	
						1-<2 serv./day	1.01 (0.80-1.28)	
						2-≥4	1.13 (0.82-1.55)	
						≥4	1.29 (0.64-2.61)	
Lelong H et al, 2017, France	NutriNet-Santé Cohort	2009-2015, 3.4 years follow-up	80426 women and men, age ≥18 years: 2413 cases	Three 24-hours dietary records, validated against biomarkers	Fruit and vegetables	199 g/d	1.00	Age, sex, smoking status alcohol consumption, BMI, physical activity, educational level, total energy intake, and family history of hypertension
						370	1.00 (0.88-1.13)	
						515	0.95 (0.84-1.07)	
						786	0.85 (0.74-0.97)	

Quinteiros Fidalgo ASQ et al, 2018, Switzerland	The CoLaus study	2009-2017, median 5 years follow-up	2079 participants, age 40-80 years: 370 cases	Self-administered semi quantitative FFQ, 97 items	Fruits<br><br><br><br>Vegetables	1<br>2<br>3<br>4<br>1<br>2<br>3<br>4	1.00<br>1.01 (0.73-1.40)<br>0.85 (0.61-1.20)<br>1.10 (0.78-1.55)<br>1.00<br>0.86 (0.62-1.20)<br>1.06 (0.77-1.47)<br>0.95 (0.67-1.33)	Gender, age, BMI, educational level, sedentariness, diabetes, total energy intake
Liu MW et al, 2018, China	China Health and Nutrition Survey (CHNS)	2006-2011, 5 years follow-up	3789 women and men, age 18–64 years: 866 cases	Three consecutive 24-h recalls	Fruit and vegetables	<243.33 g/d<br>243.33-<326.67<br>326.67-<405.00<br>405.00-<520.83<br>≥520.83	1.00<br>0.79 (0.58-1.07)<br>0.72 (0.53-0.99)<br>0.71 (0.52-0.97)<br>0.78 (0.57-1.08)	Age, gender, residence, baseline energy intake, smoking status, alcohol intake, sugar-sweetened beverage intake, leisure physical activity, intake of red meat and whole grains
Lim M, 2019, South Korea	Korean Genome and Epidemiology Study (KoGES)	2001-2009, 2002-2010, 8 years follow-up	4479 participants, age 40-69 years: 1460 cases	Validated semi-quantitative FFQ	Fruit<br><br><br><br>Vegetables	Never or rarely<br>1-<2 serv/d<br>2-<4<br>≥4<br>Never or rarely<br>1-<2 serv/d<br>2-<4<br>≥4	1.00<br>0.80 (0.66-0.97)<br>0.67 (0.55-0.81)<br>0.69 (0.55-0.88)<br>1.00<br>1.22 (0.89-1.67)<br>1.05 (0.77-1.43)<br>1.25 (0.90-1.73)	Age, residential location, household income, education level, smoking status, alcohol intake, physical activity, BMI, energy intake, vegetable intake, meat intake, refned grain intake, whole grain intake, and dairy intake
Huang M et al, 2019, China	China Health and Nutrition Survey (CHNS)	1989-2011, 11.3 years follow-up	11763 participants, age >20 years: 4033 cases	Three consecutive 24-hour dietary recalls	Total potatoes<br><br><br><br>Sweet potatoes<br><br>Non stir-fried potatoes	0 g/d<br>0-25<br>25.0-66.7<br>>66.7<br>0 g/d<br>>0<br>0 g/d<br>> 0	1.00<br>1.40 (1.27-1.55)<br>1.20 (1.01-1.42)<br>1.12 (0.93-1.35)<br>1.00<br>1.06 (0.90-1.23)<br>1.00<br>1.15 (1.00-1.32)	Age, sex, marital status, education levels, household income, and residency (urban, rural), BMI, physical activity, medical insurance, smoking status, and alcohol drinking status, total energy intake, total meat intake (red meat, poultry meat, and fish meat), total vegetables intake, total fruit intake, total sodium intake and total potassium intake.

Nguyen B et al, 2019, Australia	45 and Up Study	2006-2010, 4 years follow-up	32393 participants (18779 women, 13617 men), age >45 years: 5539 cases	Validated short questions	Fruit and vegetables (total)<br><br>Fruit and vegetables (women)<br>Fruit and vegetables (men)	<2/<3 vs. ≥2/≥3 serv./day (fruit/vegetables)<br><2/<3 vs. ≥2/≥3 serv./day (fruit/vegetables)<br><2/<3 vs. ≥2/≥3 serv./day (fruit/vegetables)	1.06 (0.99-1.13)<br><br>1.05 (0.96-1.15)<br><br>1.09 (0.97-1.21)	Age group, sex, follow-up time, country of birth, education, socio-economic status, family history of hypertension, omega 3 or fish oil use, aspirin use, BMI, physical activity level, fruit and vegetable intake, alcohol intake, smoking status, K10 score.<br>In separate analyses conducted in women: current use of hormonal replacement therapy, oral contraceptive use, menopausal status and number of children given birth to (women)<br>Additional covariates in parous women only: mother's age for first child, lifetime breastfeeding duration and high blood pressure during pregnancy
Xu Y et al, 2021, China	Urumqi County, Xinjiang	2008-2018 - NA, 4.5 years follow-up	5327 men and women, age ≥18 years: 1985 cases	Questionnaire	Fruits and vegetables	Yes vs. no	0.81 (0.69-0.95)	Age, sex, smoking, drinking, BMI, DBP, SBP, salt intake, yak-butter, excessive milk in tea, LDL cholesterol, total cholesterol, abdominal circumference, FH - hypertension
Pasdar Y et al, 2022, Iran	The RaNCD cohort study	2014-2021, mean 5.5 years follow-up	1295 participants, age 35-65 years: 294 cases	Validated semi quantitative FFQ, 118 items	Total fruits<br><br>Whole fruits<br><br>Total vegetables	≥0.8 vs. 0 cup equivalents/1000 kcal/d<br>≥0.4 vs. 0 cup equivalents/1000 kcal/d<br>≥1.1 vs. 0 cup equivalents/1000 kcal/d	0.93 (0.85-0.99)<br><br>0.94 (0.85-1.03)<br><br>1.02 (0.93-1.12)	Gender, age, socioeconomic status, educational level, physical activity, diabetes, BMI, energy intake

Supplementary table 3: Quality Assessment with Newcastle-Ottawa scale

Author (study), year	Selection			Comparability	Outcome			Total score out of 8 possible
	Selection of non-exposed cohort	Exposure ascertainment	Demonstration of utcome not present at start	0.25 points for each confounder adjusted for	Outcome assessment	Long enough follow-up (≥3 years)	Adequacy of follow-up (<10% lost)	
Steffen et al, 2005	1	1	1	2	1	1	0	7
Nuñez-Cordoba et al, 2008	1	1	1	2	0	1	1	7
Camoes et al, 2010	1	1	1	1.5	1	1	0	6.5
Tsubota-Utsugi et al, 2011	1	1	1	2	1	1	0	7
Wang et al, 2011	1	1	1	2	1	1	0	7
Weng et al, 2013	1	1	1	2	1	1	0	7
Golzarand et al, 2015	1	1	1	2	1	0	1	7
Borgi et al (NHS, F&V), 2016	1	1	1	2	1	1	0	7
Borgi et al (NHS2, F&V), 2016	1	1	1	2	1	1	0	7
Borgi et al (HPFS, F&V), 2016	1	1	1	2	1	1	0	7
Borgi et al (NHS, potatoes), 2016	1	1	1	2	1	1	0	7
Borgi et al (NHS2, potatoes), 2016	1	1	1	2	1	1	0	7
Borgi et al (HPFS, potatoes), 2016	1	1	1	2	1	1	0	7
Auerback et al, 2017	1	1	1	2	0.5	1	0	6.5
Bahadoran et al, 2017	1	1	1	2	1	1	0	7
Hu et al, 2017	1	1	1	2	0.5	1	1	7.5
Kim et al, 2017	1	1	1	2	1	1	1	8
Lelong et al, 2017	1	1	1	2	0.5	1	1	7.5

Liu et al, 2018	1	1	1	2	1	1	0	7
Quinteiros Fidalgo et al	1	1	1	1.75	1	1	0	6.75
Lim et al, 2019	1	1	1	2	1	1	0	7
Huang et al, 2019	1	1	1	2	1	1	0	7
Ngyuen et al, 2019	1	1	1	2	0.5	1	0	6.5
Pasdar et al, 2022	1	1	1	2	1	1	0	7
Xu et al, 2021	1	1	1	2	1	1	1	8

Supplemental Table 4. World Cancer Research Fund grading criteria

Grading	Criteria
Convincing	A convincing relationship should be robust enough to be highly unlikely to be modified in the foreseeable future as new evidence accumulates. All of the following are generally required:<br>- Evidence from more than one study type<br>- Evidence from at least two independent cohort studies<br>- No substantial unexplained heterogeneity within or between study types or in different populations relating to the presence or absence of an association, or direction of effect<br>- Good quality studies to exclude with confidence the possibility that the observed association results from random or systematic error, including confounding, measurement error, and selection bias<br>- Presence of a plausible biological gradient in the association. Such a gradient need not be linear or even in the same direction across different levels of exposure, so long as this can be explained plausibly<br>- Strong and plausible experimental evidence, either from human studies or relevant animal models, that typical human exposures can lead to relevant outcomes
Probable	All of the following are generally required:<br>- Evidence from at least two independent cohort studies, or at least five case-control studies<br>- No substantial unexplained heterogeneity within or between study types or in different populations relating to the presence or absence of an association, or direction of effect<br>- Good quality studies to exclude with confidence the possibility that the

	observed association results from random or systematic error, including confounding, measurement error, and selection bias<br><br>- Evidence for biological plausibility
Limited - suggestive	All of the following are generally required:<br><br>- Evidence from at least two independent cohort studies, or at least five case-control studies<br><br>- The direction of effect is generally consistent though some unexplained heterogeneity may be present<br><br>- Evidence for biological plausibility
Limited - no conclusion	Evidence is so limited that no firm conclusion can be made, but this does not mean that there is evidence of no relationship. The evidence might be graded "limited - no conclusion" for several reasons:<br><br>- limited number of studies<br><br>- inconsistency of direction of effect<br><br>- poor quality of studies (e.g. lack of adjustment for known confounders)<br><br>- or any combination of these factors
Substantial effect on risk unlikely	All of the following are generally required:<br><br>- Evidence from more than one study type<br><br>- Evidence from at least two independent cohort studies<br><br>- Summary estimate of effect close to 1.0 for comparison of high versus low exposure categories<br><br>- No substantial unexplained heterogeneity within or between study types or in

	different populations<br><br>- Good quality studies to exclude with confidence the possibility that the absence of association results from random or systematic error, including inadequate power, imprecision or error in exposure measurement, inadequate range of exposure, confounding, and selection bias<br><br>- Absence of a demonstrable biological gradient (dose response)<br><br>- Absence of strong and plausible experimental evidence, either from human studies or relevant animal models, that typical human exposures lead to relevant outcomes

Specific upgrading factors:

1) Presence of a plausible biological gradient (dose response) in the association. Such a gradient need not be linear or even in the same direction across the different levels of exposure, so long as this can be explained plausibly.

2) A particularly large summary effect size (an odds ratio or relative risk of 2.0 or more, depending on the unit of exposure) after appropriate control for confounders.

3) Evidence from randomised trials in humans.

4) Evidence from appropriately controlled experiments demonstrating one or more plausible and specific mechanisms actually operating in humans.

5) Robust and reproducible evidence from experimental studies in appropriate animal models showing that typical human exposures can lead to relevant health outcomes.

Supplementary Table 5. Fruit and vegetables and hypertension, relative risks and 95% confidence intervals from nonlinear dose-response analysis

	Fruits and vegetables (n=8)		Fruits (n=10)		Vegetables (n=9)
g/d	RRs (95% CIs)	g/d	RRs (95% CIs)	g/d	RRs (95% CIs)
40	1.00	8	1.00	40	1.00
100	0.99 (0.98-1.00)	100	0.94 (0.88-0.99)	100	0.99 (0.98-1.00)
200	0.97 (0.95-0.99)	200	0.89 (0.80-0.98)	200	0.97 (0.94-1.00)
300	0.95 (0.92-0.99)	300	0.86 (0.77-0.95)	300	0.97 (0.94-1.01)
400	0.93 (0.89-0.98)	400	0.84 (0.75-0.94)	400	0.98 (0.94-1.02)
500	0.92 (0.88-0.97)	500	0.82 (0.72-0.93)	500	0.98 (0.94-1.03)
600	0.91 (0.86-0.96)	550	0.81 (0.71-0.92)		
700	0.90 (0.85-0.96)				
800	0.89 (0.83-0.96)				
$p_{nonlinearity}$	0.23	$p_{nonlinearity}$	0.23	$p_{nonlinearity}$	0.09

Supplementary Table 6. Fruit subtypes and hypertension, relative risks and 95% confidence intervals from nonlinear dose-response analysis

	Apples or pears (n=4)		Avocado (n=3)		Bananas (n=4)		Blueberries (n=4)
g/d	RRs (95% CIs)	g/d	RRs (95% CIs)	g/d	RRs (95% CIs)	g/d	RRs (95% CIs)
2.1	1.00	2.25	1.00	1.71	1.00	1.1	1.00
20	0.98 (0.96-1.00)	20	0.95 (0.92-0.98)	20	1.01 (0.99-1.03)	10	0.97 (0.95-0.99)
40	0.96 (0.94-0.99)	40	0.92 (0.87-0.96)	40	1.02 (0.99-1.04)	20	0.96 (0.93-0.99)
60	0.95 (0.93-0.98)	60	0.90 (0.85-0.97)	60	1.01 (0.99-1.04)	30	0.97 (0.91-1.03)
80	0.95 (0.92-0.97)	80	0.91 (0.82-1.01)	80	1.01 (0.98-1.03)	40	0.99 (0.88-1.12)
100	0.94 (0.92-0.97)	100	0.92 (0.78-1.08)	97.6	1.00 (0.98-1.03)	50	1.03 (0.85-1.24)
$p_{nonlinearity}$	0.14	$p_{nonlinearity}$	0.11	$p_{nonlinearity}$	0.16	$p_{nonlinearity}$	0.09

Supplementary Table 7. Fruit subtypes and hypertension, relative risks and 95% confidence intervals from nonlinear dose-response analysis

	Cantaloupe (n=3)		Oranges (n=4)		Peaches, apricots, plums (n=3)		Prunes (n=3)
g/d	RRs (95% CIs)	g/d	RRs (95% CIs)	g/d	RRs (95% CIs)	g/d	RRs (95% CIs)
2.0	1.00	2.0	1.00	1.3	1.00	1.2	1.00
20	1.03 (1.02-1.05)	20	0.99 (0.97-1.01)	20	1.02 (0.99-1.04)	20	0.97 (0.93-1.02)
40	1.05 (1.03-1.08)	40	0.98 (0.96-1.01)	40	1.02 (0.99-1.05)	40	0.97 (0.91-1.03)
60	1.05 (1.01-1.10)	60	0.97 (0.94-1.00)	60	1.01 (0.96-1.06)	60	0.96 (0.88-1.06)
80	1.04 (0.97-1.12)	80	0.97 (0.94-1.00)				
96	1.03 (0.94-1.14)	93.5	0.96 (0.92-0.99)				
$p_{nonlinearity}$	0.05	$p_{nonlinearity}$	0.92	$p_{nonlinearity}$	0.20	$p_{nonlinearity}$	0.53

Supplementary Table 8. Fruit subtypes and hypertension, relative risks and 95% confidence intervals from nonlinear dose-response analysis

	Raisins or grapes (n=4)		Strawberries (n=4)
g/d	RRs (95% CIs)	g/d	RRs (95% CIs)
1.1	1.00	1.1	1.00
10	0.97 (0.96-0.99)	10	1.00 (0.98-1.02)
20	0.95 (0.92-0.97)	20	1.01 (0.98-1.04)
30	0.93 (0.90-0.96)	30	1.02 (0.98-1.05)
40	0.92 (0.87-0.97)	40	1.02 (0.99-1.06)
50	0.91 (0.84-0.98)	50	1.03 (0.99-1.07)
57	0.90 (0.82-0.99)	54	1.03 (0.98-1.08)
$p_{nonlinearity}$	0.20	$p_{nonlinearity}$	0.95

Supplementary Table 9. Vegetable subtypes and hypertension, relative risks and 95% confidence intervals from nonlinear dose-response analysis

	Avocado (n=3)		Broccoli (n=3)		Brussel sprouts (n=3)		Cabbage (n=3)
g/d	RRs (95% CIs)	g/d	RRs (95% CIs)	g/d	RRs (95% CIs)	g/d	RRs (95% CIs)
2.25	1.00	1.1	1.00	1.17	1.00	1.0	1.00
20	0.95 (0.92-0.98)	10	0.98 (0.97-1.00)	10	1.02 (1.00-1.04)	10	1.01 (0.99-1.03)
40	0.92 (0.87-0.96)	20	0.97 (0.95-0.99)	20	1.04 (1.01-1.07)	20	1.02 (0.99-1.05)
60	0.90 (0.85-0.97)	30	0.96 (0.93-0.98)	30	1.07 (1.02-1.12)	30	1.03 (1.00-1.06)
80	0.91 (0.82-1.01)	40	0.95 (0.92-0.97)	40	1.10 (1.02-1.19)	40	1.04 (0.99-1.09)
100	0.92 (0.78-1.08)	50	0.94 (0.91-0.97)	50	1.14 (1.00-1.29)	50	1.05 (0.97-1.14)
				56	1.16 (0.99-1.36)		
$p_{nonlinearity}$	0.11	$p_{nonlinearity}$	0.42	$p_{nonlinearity}$	0.64	$p_{nonlinearity}$	0.89

Supplementary Table 10. Vegetable subtypes and hypertension, relative risks and 95% confidence intervals from nonlinear dose-response analysis

	Carrot (n=3)		Cauliflower (n=3)		Corn (n=3)		Cruciferous vegetables (n=2)
g/d	RRs (95% CIs)	g/d	RRs (95% CIs)	g/d	RRs (95% CIs)	g/d	RRs (95% CIs)
0.86	1.00	0.83	1.00	1.2	1.00	3.6	1.00
10	0.98 (0.96-1.00)	10	1.02 (0.98-1.07)	10	1.01 (1.00-1.03)	20	1.05 (0.94-1.16)
20	0.96 (0.93-0.99)	20	1.04 (0.97-1.11)	20	1.03 (1.00-1.05)	40	1.09 (0.91-1.31)
30	0.96 (0.93-0.99)	30	1.05 (0.98-1.13)	30	1.03 (1.01-1.06)	60	1.10 (0.96-1.26)
40	0.95 (0.93-0.98)	40	1.07 (0.99-1.15)	40	1.03 (0.99-1.08)	80	1.07 (0.97-1.18)
				50	1.03 (0.96-1.12)	100	1.04 (0.79-1.36)
				57	1.03 (0.93-1.15)		
$p_{nonlinearity}$	0.24	$p_{nonlinearity}$	0.83	$p_{nonlinearity}$	0.54	$p_{nonlinearity}$	0.60

Supplementary Table 11. Vegetable subtypes and hypertension, relative risks and 95% confidence intervals from nonlinear dose-response analysis

	Green pepper (n=3)		Lettuce (n=3)		Onions (n=4)
g/d	RRs (95% CIs)	g/d	RRs (95% CIs)	g/d	RRs (95% CIs)
1.2	1.00	0.8	1.00	1.2	1.00
10	1.01 (0.99-1.02)	10	0.97 (0.94-1.00)	10	1.00 (0.98-1.02)
20	1.01 (0.99-1.04)	20	0.95 (0.91-1.00)	20	1.00 (0.97-1.04)
30	1.01 (0.99-1.04)	30	0.95 (0.90-0.99)	30	1.00 (0.96-1.05)
40	1.01 (0.98-1.04)	40	0.95 (0.91-0.99)	40	1.00 (0.95-1.05)
50	1.00 (0.97-1.04)			50	1.00 (0.94-1.06)
57	1.00 (0.96-1.04)			57	0.99 (0.93-1.06)
$p_{nonlinearity}$	0.18	$p_{nonlinearity}$	0.20	$p_{nonlinearity}$	0.79

Supplementary Table 12. Vegetable subtypes and hypertension, relative risks and 95% confidence intervals from nonlinear dose-response analysis

	Potatoes (total) (n=5)		Potatoes (fried) (n=5)		Potatoes (non-fried) (n=5)		Tomatoes (n=4)		Yams or sweet potatoes (n=4)
g/d	RRs (95% CIs)	g/d	RRs (95% CIs)	g/d	RRs (95% CIs)	g/d	RRs (95% CIs)	g/d	RRs (95% CIs)
0	1.00	1.2	1.00	6.1	1.00	1.8	1.00	3.0	1.00
50	1.05 (1.02-1.08)	20	1.05 (1.03-1.07)	20	1.01 (1.00-1.03)	20	1.01 (0.98-1.03)	20	1.01 (1.00-1.02)
100	1.09 (1.04-1.14)	40	1.09 (1.06-1.13)	40	1.03 (0.99-1.06)	40	1.01 (0.97-1.05)	40	1.02 (1.00-1.04)
150	1.11 (1.03-1.19)	60	1.12 (1.08-1.16)	60	1.04 (0.99-1.10)	60	1.01 (0.97-1.06)	60	1.03 (1.01-1.05)
200	1.12 (1.01-1.24)	80	1.13 (1.09-1.17)	80	1.05 (0.99-1.12)	80	1.02 (0.98-1.05)	80	1.03 (1.00-1.06)
231	1.12 (0.98-1.27)	100	1.13 (1.08-1.18)	100	1.06 (0.98-1.14)	100	1.02 (0.99-1.05)	100	1.03 (0.99-1.08)
		120	1.11 (1.04-1.20)	120	1.06 (0.98-1.16)	120	1.02 (0.99-1.05)	120	1.03 (0.97-1.11)
		140	1.10 (0.99-1.22)	140	1.07 (0.97-1.17)	140	1.02 (0.98-1.06)	140	1.03 (0.94-1.14)
$p_{nonlinearity}$	0.07	$p_{nonlinearity}$	0.02	$p_{nonlinearity}$	0.10	$p_{nonlinearity}$	0.80	$p_{nonlinearity}$	0.54

Supplementary Table 13. Subgroup analyses of fruits and vegetables and risk of hypertension (per 200 g/d)

	Fruits and vegetables					Fruits					Vegetables				
	n	RR (95% CI)	I^2	P_h^1	P_h^2	n	RR (95% CI)	I^2	P_h^1	P_h^2	n	RR (95% CI)	I^2	P_h^1	P_h^2
All studies	8	0.97 (0.95-0.99)	68.1	0.003		10	0.93 (0.89-0.98)	77.3	<0.0001		9	1.00 (0.98-1.03)	45.0	0.07	
Duration of follow-up															
<10 years follow-up	3	0.90 (0.82-1.00)	39.7	0.19	0.19	5	0.91 (0.76-1.09)	85.5	<0.0001	0.96	4	1.00 (0.90-1.10)	6.1	0.36	0.94
≥10 years follow-up	5	0.98 (0.95-1.00)	73.0	0.005		5	0.94 (0.90-0.98)	66.7	0.02		5	1.00 (0.97-1.03)	64.7	0.02	
Gender															
Men	1	0.97 (0.94-0.99)			0.30/ 0.66[3]	2	0.88 (0.74-1.04)	85.8	0.008	0.99/ 0.43[3]	1	1.00 (0.96-1.03)			0.47/ 0.92[3]
Women	3	0.98 (0.95-1.01)	83.4	0.002		5	0.95 (0.91-1.00)	81.1	<0.0001		4	1.00 (0.97-1.04)	71.7	0.01	
Men and women	4	0.91 (0.85-0.97)	24.8	0.26		4	0.80 (0.60-1.08)	67.6	0.03		4	0.91 (0.79-1.05)	0	0.66	
Geographic location															
Europe	3	0.90 (0.82-1.00)	39.7	0.19	0.41	1	0.97 (0.79-1.18)			0.15	1	0.94 (0.75-1.19)			0.67
America	4	0.98 (0.96-1.00)	76.6	0.005		6	0.95 (0.91-1.00)	74.9	0.001		5	1.00 (0.97-1.03)	64.7	0.02	
Asia	1	0.87 (0.75-1.02)				3	0.78 (0.56-1.09)	68.8	0.04		3	0.93 (0.72-1.21)	29.8	0.24	
Number of cases															
Cases <500	2	0.84 (0.73-0.96)	0	0.38	0.05	3	0.83 (0.56-1.23)	70.2	0.04	0.94	3	0.89 (0.72-1.09)	0	0.47	0.38
Cases 500-<1000	1	0.87 (0.75-1.02)				0					0				
Cases ≥1000	5	0.97 (0.95-0.99)	73.1	0.005		7	0.93 (0.89-0.98)	81.8	<0.0001		6	1.00 (0.98-1.03)	57.6	0.04	
Definition of hypertension															
≥140/≥90 mmHg	7	0.97 (0.95-0.99)	70.1	0.003	0.50	7	0.96 (0.92-0.99)	62.5	0.01	0.02	3	1.01 (0.93-1.09)	0	0.46	0.95
≥130 or ≥135/≥85 mmHg	0					3	0.70 (0.51-0.98)	67.8	0.05		6	1.00 (0.97-1.03)	61.4	0.02	

Only self-report (no cut-off)		1	0.94 (0.90-0.99)				0					0				
Study quality																
0-3 stars		0				NC	0				NC	0				0.20
>3-6		0					0					1	0.61 (0.30-1.21)			
>6-8		8	0.97 (0.95-0.99)	68.1	0.003		10	0.93 (0.89-0.98)	77.3	<0.0001		8	1.00 (0.98-1.03)	44.0	0.09	
Adjustment for confounding factors																
Age	Yes	8	0.97 (0.95-0.99)	68.1	0.003	NC	10	0.93 (0.89-0.98)	77.3	<0.0001	NC	9	1.00 (0.98-1.03)	45.0	0.07	NC
	No	0					0					0				
Education	Yes	2	0.88 (0.74-1.06)	66.1	0.09	0.28	3	0.87 (0.70-1.08)	91.9	<0.0001	0.62	2	1.01 (0.93-1.10)	5.3	0.30	0.86
	No	6	0.97 (0.95-0.99)	68.6	0.007		7	0.94 (0.91-0.98)	59.9	0.02		7	1.00 (0.97-1.03)	55.3	0.04	
Family history of hypertension	Yes	5	0.97 (0.95-0.99)	31.2	0.21	0.27	4	0.94 (0.90-0.98)	59.8	0.06	0.73	4	1.00 (0.96-1.04)	71.7	0.01	0.87
	No	3	0.91 (0.78-1.05)	75.4	0.02		6	0.90 (0.79-1.01)	84.3	<0.0001		5	1.01 (0.98-1.04)	0	0.45	
Smoking	Yes	7	0.97 (0.95-0.99)	64.5	0.01	0.11	10	0.93 (0.89-0.98)	77.3	<0.0001	NC	9	1.00 (0.98-1.03)	45.0	0.07	NC
	No	1	0.78 (0.62-0.97)				0					0				
Alcohol	Yes	7	0.97 (0.95-0.99)	64.5	0.01	0.11	8	0.92 (0.87-0.96)	76.9	<0.0001	0.23	8	1.00 (0.98-1.03)	44.0	0.09	0.58
	No	1	0.78 (0.62-0.97)				2	1.04 (0.97-1.11)	0	0.48		1	0.61 (0.30-1.21)			
Body mass index	Yes	7	0.97 (0.95-0.99)	70.1	0.003	0.33	9	0.94 (0.89-0.99)	77.7	<0.0001	0.22	8	1.00 (0.98-1.03)	49.7	0.05	0.45
	No	1	0.87 (0.75-1.02)				1	0.71 (0.53-0.95)				1	0.93 (0.77-1.12)			
Physical activity	Yes	8	0.97 (0.95-0.99)	68.1	0.003	NC	10	0.93 (0.89-0.98)	77.3	<0.0001	NC	9	1.00 (0.98-1.03)	45.0	0.07	NC
	No	0					0					0				
Sodium	Yes	1	0.88 (0.73-1.06)			0.47	3	0.83 (0.56-1.23)	70.2	0.04	0.94	3	0.89 (0.72-1.09)	0	0.47	0.30
	No	7	0.97 (0.95-0.99)	71.3	0.002		7	0.93 (0.89-0.98)	81.8	<0.0001		6	1.00 (0.98-1.03)	57.6	0.04	

Sugar-sweetened beverages	Yes	4	0.97 (0.95-0.99)	45.2	0.14	0.90	3	0.94 (0.90-0.98)	73.0	0.03	0.82	3	1.00 (0.96-1.04)	80.7	0.006	0.96
	No	4	0.95 (0.89-1.01)	75.3	0.007		7	0.91 (0.82-1.01)	81.2	<0.0001		6	1.01 (0.98-1.03)	0	0.56	
Meat	Yes	5	0.98 (0.95-1.00)	73.0	0.005	0.19	5	0.92 (0.88-0.97)	80.0	<0.0001	0.70	5	1.00 (0.98-1.03)	64.1	0.03	0.25
	No	3	0.90 (0.82-1.00)	39.7	0.19		5	0.89 (0.73-1.09)	72.0	0.006		4	0.91 (0.79-1.05)	0	0.66	
Fish	Yes	4	0.97 (0.95-0.98)	36.4	0.19	0.99	4	0.94 (0.90-0.98)	59.8	0.06	0.73	4	1.00 (0.96-1.04)	71.7	0.01	0.87
	No	4	0.94 (0.88-1.01)	77.3	0.004		6	0.90 (0.79-1.01)	84.3	<0.0001		5	1.01 (0.98-1.04)	0	0.45	
Whole grains	Yes	6	0.97 (0.95-0.99)	68.6	0.007	0.28	6	0.93 (0.89-0.97)	75.1	0.001	0.80	6	1.00 (0.98-1.03)	56.0	0.04	0.29
	No	2	0.88 (0.74-1.06)	66.1	0.09		4	0.83 (0.61-1.13)	78.7	0.003		3	0.89 (0.75-1.06)	0	0.48	
Dairy products	Yes	2	0.98 (0.88-1.08)	43.7	0.18	0.13	3	0.91 (0.79-1.04)	83.8	0.002	0.68	3	1.01 (0.98-1.04)	0	0.75	0.65
	No	6	0.96 (0.94-0.98)	51.8	0.07		7	0.94 (0.89-1.00)	77.2	<0.0001		6	1.00 (0.96-1.03)	62.9	0.02	
Energy intake	Yes	8	0.97 (0.95-0.99)	68.1	0.003	NC	10	0.93 (0.89-0.98)	77.3	<0.0001	NC	9	1.00 (0.98-1.03)	45.0	0.07	NC
	No	0					0					0				

N denotes the number of studies.
[1] P for heterogeneity within each subgroup,
[2] P for heterogeneity between subgroups with meta-regression analysis,
[3] P for heterogeneity between men and women (studies with men and women combined were excluded),
NC = not calculable

Supplemental Table 14. Justification for evidence grading for fruit and vegetables and hypertension

Requirements for grading of convincing	Fruit and vegetables	Fruits	Vegetables
Statistically significant and robust association	A statistically significant 11% reduction in risk was observed for high vs. low intake and at the highest level of intake in the nonlinear dose-response analysis, and this was robust in influence analyses. Two studies only reported dichotomous results and could not be included in the dose-response analyses.	A statistically significant 9% reduction in risk was observed for high vs. low intake and a 19% reduction in risk was observed at the highest level of intake in the nonlinear dose-response analysis. Results were robust in influence analyses. Two studies could not be included in the dose-response analysis.	No significant association was observed in high vs. low, linear and nonlinear dose-response analyses, and there is no evidence of nonlinearity. The lack of association is not driven by any single studies.
Evidence from at least two independent cohort studies	10 studies (high vs. low)<br>8 studies (dose-response)	13 studies (high vs. low)<br>10 studies (dose-response)	12 studies (high vs. low)<br>9 studies (dose-response)
No substantial unexplained heterogeneity within or between study types or in different populations relating to the presence or absence of an association, or direction of effect	There was moderate heterogeneity in the high vs. low analysis and moderate to high heterogeneity in the dose-response analysis, however, this was largely due to differences in the strength of the association (all except one study reported associations in the direction of reduced risk). The inverse associations persisted across most subgroup analyses.	Heterogeneity in high vs. low analysis was moderate and in in the linear dose-response analysis was high. All studies except one show risk estimates in the direction of reduced risk in the dose-response analysis, although not all are statistically significant. No studies show a significant increase in risk.	No to low heterogeneity in the high vs. low and dose-response analyses.<br>All studies reported no statistically significant association in the high vs. low analysis. Out of twelve studies in the high vs. low analysis, ten reported estimates in the direction of reduced risk, and two in the direction of increased risk. Results were similar and close to null for most studies in the dose-response analysis.
Good quality studies to	No indication of publication bias in	No indication of publication bias.	No indication of publication bias.

exclude with confidence the possibility that the observed association results from random or systematic error, including confounding, measurement error, and selection bias	the dose-response analysis<br><br>Results persisted in several, although not all subgroup analyses. However, there was no indication of between subgroup heterogeneity with meta-regression analyses.<br><br>No studies corrected for measurement error.<br><br>All studies excluded prevalent hypertension cases at baseline. Exposed and non-exposed participants were selected from the same populations.	Results are in general consistent in subgroup analyses, although for some subgroups there is low power due to few studies. There is no indication of significant between subgroup heterogeneity.<br><br>No studies corrected for measurement error.<br><br>All studies excluded prevalent hypertension cases at baseline. Exposed and non-exposed participants were selected from the same populations.	Subgroup analyses based on high vs. low and dose-response analyses show consistently no significant association.<br><br>No studies corrected for measurement error.<br><br>All studies excluded prevalent hypertension cases at baseline. Exposed and non-exposed participants were selected from the same populations.
Presence of a plausible biological gradient in the association. Such a gradient need not be linear or even in the same direction across different levels of exposure, so long as this can be explained	Evidence of a clear dose-response relationship up to an intake of 800 g/d and the test for nonlinearity was not significant (p=0.21).	There is strong evidence of an inverse dose-response relationship up to an intake of 550 g/d, and there is no indication of nonlinearity (p=0.31). Several subtypes of fruit are inversely associated with hypertension, while a few fruit subtypes (cantaloupe, watermelon) are slightly positively associated	There is no evidence of nonlinearity (p=0.10). Different types of vegetables show associations in opposite directions, which potentially could explain the null association for vegetables overall.

plausibly		with hypertension.	
Strong and plausible experimental evidence, either from human studies or relevant animal models, that typical human exposures can lead to relevant outcomes	There is evidence from a meta-analysis of two randomized trials that increased fruit and vegetable consumption can reduce blood pressure.<br><br>There is considerable evidence from observational studies that a high fruit and vegetable intake reduces weight gain and prevents the development of overweight and obesity. Results from randomized trials show weaker associations between fruit and vegetable intake and weight gain, but are of short duration and there may be issues with compliance.<br><br>Fruits and vegetables are important sources of dietary fiber, for which there is convincing evidence of a protective effect on adiposity and weight gain, which are major risk factors for hypertension. There is also some evidence that high fiber intake may reduce blood pressure directly independently of adiposity, e.g. by affecting vascular endothelial function, mineral absorption, serum cholesterol, glycemic control/insulin resistance, and gastrointestinal	There is evidence from a meta-analysis of two randomized trials that increased fruit and vegetable consumption can reduce blood pressure, however, these trials did not specifically look only at fruit intake.<br><br>There is considerable evidence from observational studies that a high fruit intake reduces weight gain and prevents the development of overweight and obesity. Results from randomized trials show weaker associations between fruit intake and weight gain, but are of short duration and there may be issues with compliance.<br><br>Fruits are important sources of dietary fiber, for which there is convincing evidence of a protective effect on adiposity and weight gain, which are major risk factors for hypertension. There is also some evidence that high fiber intake may reduce blood pressure directly independently of adiposity, e.g. by affecting vascular endothelial function, mineral absorption, serum cholesterol, glycemic control/insulin resistance, and gastrointestinal	There is evidence from a meta-analysis of two randomized trials that increased fruit and vegetable consumption can reduce blood pressure, however, these trials did not specifically look only at vegetable intake.<br><br>There is considerable evidence from observational studies that a high vegetable intake reduces weight gain and prevents the development of overweight and obesity. Results from randomized trials show weaker associations between vegetable intake and weight gain, but are of short duration and there may be issues with compliance.<br><br>Vegetables are important sources of dietary fiber, for which there is convincing evidence of a protective effect on adiposity and weight gain, which are major risk factors for hypertension. There is also some evidence that high fiber intake may reduce blood pressure directly independently of adiposity, e.g. by affecting vascular endothelial function, mineral absorption, serum cholesterol, glycemic control/insulin resistance, and

	function.<br><br>Fruits and vegetables have a high content of various antioxidants and nutrients including potassium, vitamin C, vitamin E and folate, which may contribute towards reduced hypertension risk. There is evidence from randomized trials that potassium supplementation has a U-shaped relation with systolic and diastolic blood pressure. There is also evidence from meta-analyses of randomized controlled trials that vitamin C supplementation can reduce systolic and diastolic blood pressure and vitamin E supplementation can reduce systolic blood pressure. Another meta-analysis found folic acid supplementation combined with blood pressure lowering medication reduces systolic and diastolic blood pressure significantly compared to using only blood pressure lowering medication.	function.<br><br>Fruits and vegetables have a high content of various antioxidants and nutrients including potassium, vitamin C, vitamin E and folate, which may contribute towards reduced hypertension risk. There is evidence from randomized trials that potassium supplementation has a U-shaped relation with systolic and diastolic blood pressure. There is also evidence from meta-analyses of randomized controlled trials that vitamin C supplementation can reduce systolic and diastolic blood pressure and vitamin E supplementation can reduce systolic blood pressure. Another meta-analysis found folic acid supplementation combined with blood pressure lowering medication reduces systolic and diastolic blood pressure significantly compared to using only blood pressure lowering medication.	gastrointestinal function.<br><br>Vegetables have a high content of various antioxidants and nutrients including potassium, vitamin C, vitamin E and folate, which may contribute towards reduced hypertension risk. There is evidence from randomized trials that potassium supplementation has a U-shaped relation with systolic and diastolic blood pressure. There is also evidence from meta-analyses of randomized controlled trials that vitamin C supplementation can reduce systolic and diastolic blood pressure and vitamin E supplementation can reduce systolic blood pressure. Another meta-analysis found folic acid supplementation combined with blood pressure lowering medication reduces systolic and diastolic blood pressure significantly compared to using only blood pressure lowering medication.
Final grading and justification for overall assessment.	Probable evidence for reduced risk with higher fruit and vegetable intake.<br><br>Justification: Clear and significant inverse associations in high vs. low,	Probable evidence for reduced hypertension risk with higher fruit intake.<br><br>Justification: Significant high vs. low, linear and nonlinear dose-	Limited-no conclusion evidence for the association between vegetables and hypertension.<br><br>Justification: This is largely based on the lack of association in both

	linear and nonlinear dose-response analyses and with evidence of a dose-response relationship. Results are in general consistent across most subgroup analyses, although not always statistically significant in every subgroup, but this is likely due to few studies. Moderate to high heterogeneity (but largely due to different strength of association in various studies), no publication bias, no indication of selection bias. Biologically plausible mechanisms exist and there is data from randomized controlled trials that fruits and vegetables and certain nutrients that can be found in fruits and vegetables can reduce blood pressure, and that dietary patterns (DASH-diet) high in fruits and vegetables can reduce blood pressure.	response analyses with a clear dose-response relationship. Moderate to high heterogeneity (which is driven by differences in the size of the effect estimates). Inverse associations are observed in many subgroup analyses as well as in influence analyses. No publication bias and no indication of selection bias. The overall data for fruits are also consistent with the results for several subtypes of fruits, which show inverse associations (although the number of studies is limited). Biologically plausible mechanisms exist and there is data from randomized controlled trials that fruits and certain nutrients that can be found in fruits can reduce blood pressure, and that dietary patterns (DASH-diet) high in fruit (and vegetables) can reduce blood pressure.	high vs. low, linear dose-response and nonlinear dose-response analyses. The null results are robust in subgroup and influence analyses. There is little heterogeneity and no indication of publication bias. Sensitivity analyses excluding one study at a time shows no single study account for the null association. No indication of selection bias. Different vegetable subtypes shows different direction of associations, which potentially could explain the overall null association. Biologically plausible mechanisms by which vegetables could reduced hypertension risk exist.

Supplemental Table 15. Evidence grading for fruit and vegetables and subtypes and hypertension

	Reduced risk	Increased risk
Convincing	-	-
Probable	Fruits and vegetables combined, total fruits	-
Limited-suggestive	Apples/pears, blueberries, grapes and raisins, avocado, broccoli, carrots, lettuce	Cantaloupe, brussel sprouts, total potatoes, fried potatoes, non-fried potatoes
Limited - no conclusion	Vegetables, bananas, oranges, peaches/plums/apricots, prunes, strawberries, cabbage, cauliflower, corn, onions, tomatoes, yams or sweet potatoes	

For fruit and vegetable subtypes these judgments were largely based on the limited number of studies published.
