# Supplementary material for: Fruit and vegetable consumption and the risk of hypertension: a systematic review and meta-analysis of prospective studies
Source: Eur J Nutr. 2023 Apr 27;62(5):1941–55. doi: 10.1007/s00394-023-03145-5 (PMC10349693; doi:10.1007/s00394-023-03145-5)
Supplement: Supplementary file 2 — Supplementary file2 (PDF 1323 KB) [file 394_2023_3145_MOESM2_ESM.pdf]

Supplement for

Madsen H, Sen A, Aune D. Fruit and vegetable consumption and the risk of hypertension: a systematic review and meta-analysis of prospective studies. Eur J Nutr
https://doi.org/10.1007/s00394-023-03145-5

Supplementary figures

Supplementary figures of subtypes of fruit, high vs. low, linear and nonlinear dose response analyses

Supplementary Figure 1. Apples and pears and hypertension, high vs. low

Supplementary Figure 2. Apples and pears and hypertension, dose-response analysis per 100 g/d

Supplementary Figure 3. Apples and pears and hypertension, nonlinear dose-response analysis

Supplementary Figure 4. Bananas and hypertension, high vs. low

Supplementary Figure 5. Bananas and hypertension, dose-response analysis per 100 g/d

Supplementary Figure 6. Bananas and hypertension, nonlinear dose-response analysis

Supplementary Figure 7. Blueberries and hypertension, high vs. low

Supplementary Figure 8. Blueberries and hypertension, dose-response analysis per 100 g/d

Supplementary Figure 9. Blueberries and hypertension, nonlinear dose-response analysis

Supplementary Figure 10. Cantaloupe and hypertension, high vs. low

Supplementary Figure 11. Cantaloupe and hypertension, dose-response analysis per 100 g/d

Supplementary Figure 12. Cantaloupe and hypertension, nonlinear dose-response analysis

Supplementary Figure 13. Oranges and hypertension, high vs. low

Supplementary Figure 14. Oranges and hypertension, dose-response per 100 g/d

Supplementary Figure 15. Oranges and hypertension, nonlinear dose-response analysis

Supplementary Figure 16. Peaches, apricots or plums and hypertension, high vs. low

Supplementary Figure 17. Peaches, apricots or plums and hypertension, dose-response per 100 g/d

Supplementary Figure 18. Peaches, apricots or plums and hypertension, nonlinear dose-response analysis

Supplementary Figure 19. Prunes and hypertension, high vs. low

Supplementary Figure 20. Prunes and hypertension, dose-response per 100 g/d

Supplementary Figure 21. Prunes and hypertension, nonlinear dose-response analysis

Supplementary Figure 22. Raisins or grapes and hypertension, high vs. low

Supplementary Figure 23. Raisins or grapes and hypertension, dose-response per 100 g/d

Supplementary Figure 24. Raisins or grapes and hypertension, nonlinear dose-response analysis

Supplementary Figure 25. Strawberries and hypertension, high vs. low

Supplementary Figure 26. Strawberries and hypertension, dose-response per 100 g/d

Supplementary Figure 27. Strawberries and hypertension, nonlinear dose-response analysis

Supplementary figures of subtypes of vegetables, high vs. low and dose-response analyses

Supplementary Figure 28. Avocado and hypertension, high vs. low

Supplementary Figure 29. Avocado and hypertension, dose-response analysis per 100 g/d

Supplementary Figure 30. Avocado and hypertension, nonlinear dose-response analysis

Supplementary Figure 31. Broccoli and hypertension, high vs. low

Supplementary Figure 32. Broccoli and hypertension, dose-response analysis per 100 g/d

Supplementary Figure 33. Broccoli and hypertension, nonlinear dose-response analysis

Supplementary Figure 34. Brussel sprouts and hypertension, high vs. low

Supplementary Figure 35. Brussel sprouts and hypertension, dose-response analysis per 100 g/d

Supplementary Figure 36. Brussel sprouts and hypertension, nonlinear dose-response analysis

Supplementary Figure 37. Cabbage and hypertension, high vs. low

Supplementary Figure 38. Cabbage and hypertension, dose-response analysis per 100 g/d

Supplementary Figure 39. Cabbage and hypertension, nonlinear dose-response analysis

Supplementary Figure 40. Carrots and hypertension, high vs. low

Supplementary Figure 41. Carrots and hypertension, dose-response analysis per 100 g/d

Supplementary Figure 42. Carrots and hypertension, nonlinear dose-response analysis

Supplementary Figure 43. Cauliflower and hypertension, high vs. low

Supplementary Figure 44. Cauliflower and hypertension, dose-response analysis per 100 g/d

Supplementary Figure 45. Cauliflower and hypertension, nonlinear dose-response analysis

Supplementary Figure 46. Corn and hypertension, high vs. low

Supplementary Figure 47. Corn and hypertension, dose-response analysis per 100 g/d

Supplementary Figure 48. Corn and hypertension, nonlinear dose-response analysis

Supplementary Figure 49. Cruciferous vegetables and hypertension, high vs. low

Supplementary Figure 50. Cruciferous vegetables and hypertension, dose-response analysis per 100 g/d

Supplementary Figure 51. Cruciferous vegetables and hypertension, nonlinear dose-response analysis

Supplementary Figure 52. Green leafy vegetables and hypertension, high vs. low

Supplementary Figure 53. Green leafy vegetables and hypertension, dose-response analysis per 100 g/d

Supplementary Figure 54. Green leafy vegetables and hypertension, nonlinear dose-response analysis

Supplementary Figure 55. Green pepper and hypertension, high vs. low

Supplementary Figure 56. Green pepper and hypertension, dose-response analysis per 100 g/d

Supplementary Figure 57. Green pepper and hypertension, nonlinear dose-response analysis

Supplementary Figure 58. Lettuce and hypertension, high vs. low

Supplementary Figure 59. Lettuce and hypertension, dose-response analysis per 100 g/d

Supplementary Figure 60. Lettuce and hypertension, nonlinear dose-response analysis

Supplementary Figure 61. Onions and hypertension, high vs. low

Supplementary Figure 62. Onions and hypertension, dose-response per 100 g/d

Supplementary Figure 63. Onions and hypertension, nonlinear dose-response analysis

Supplementary Figure 64. Potatoes total and hypertension, high vs. low

Supplementary Figure 65. Potatoes total and hypertension, dose-response per 100 g/d

Supplementary Figure 66. Potatoes total and hypertension, nonlinear dose-response analysis

Supplementary Figure 67. Potatoes fried and hypertension, high vs. low

Supplementary Figure 68. Potatoes fried and hypertension, dose-response per 100 g/d

Supplementary Figure 69. Potatoes fried and hypertension, nonlinear dose-response analysis

Supplementary Figure 70. Potatoes non-fried and hypertension, high vs. low

Supplementary Figure 71. Potatoes non-fried and hypertension, dose-response per 100 g/d

Supplementary Figure 72. Potatoes non-fried and hypertension, nonlinear dose-response analysis

Supplementary Figure 73. Tomatoes and hypertension, high vs. low

Supplementary Figure 74. Tomatoes and hypertension, dose-response per 100 g/d

Supplementary Figure 75. Tomatoes and hypertension, nonlinear dose-response analysis

Supplementary Figure 76. Yams or sweet potatoes and hypertension, high vs. low

Supplementary Figure 77. Yams or sweet potatoes and hypertension, dose-response per 100 g/d

Supplementary Figure 78. Yams or sweet potatoes and hypertension, nonlinear dose-response analysis

Funnel plots

Supplementary Figure 79. Funnel plot of fruits, vegetables and hypertension

Supplementary Figure 80. Funnel plot of fruit and hypertension

Supplementary Figure 81. Funnel plot of vegetables and hypertension

Sensitivity analyses

Supplementary Figure 82. Sensitivity analysis of fruits, vegetables and hypertension

Supplementary Figure 83. Sensitivity analysis of fruit and hypertension

Supplementary Figure 84. Sensitivity analysis of vegetables and hypertension

Supplementary Figure 1. Apples and pears and hypertension, high vs. low

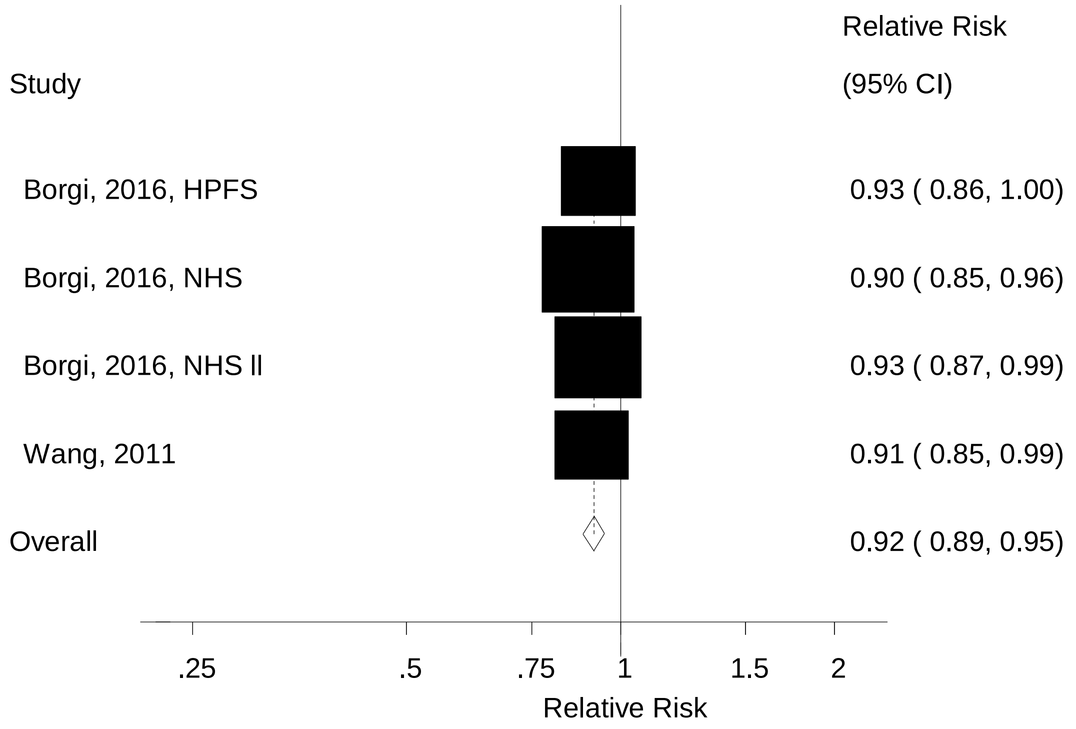

Supplementary Figure 2. Apples and pears and hypertension, dose-response analysis per 100 g/d

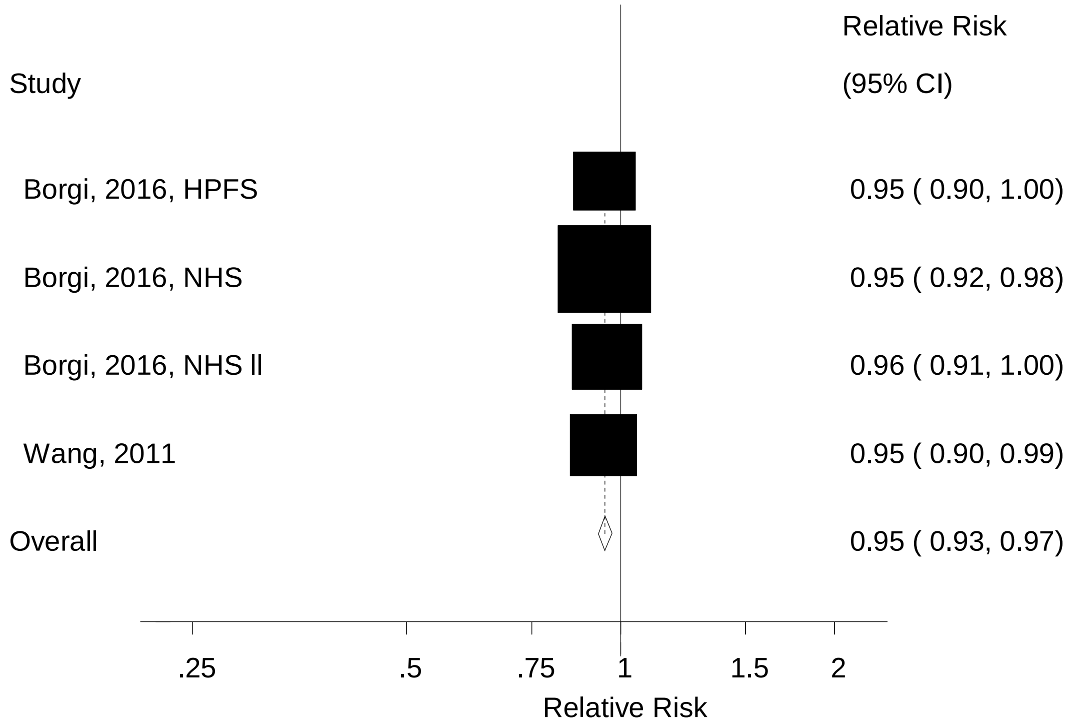

Supplementary Figure 3. Apples and pears and hypertension, nonlinear dose-response analysis

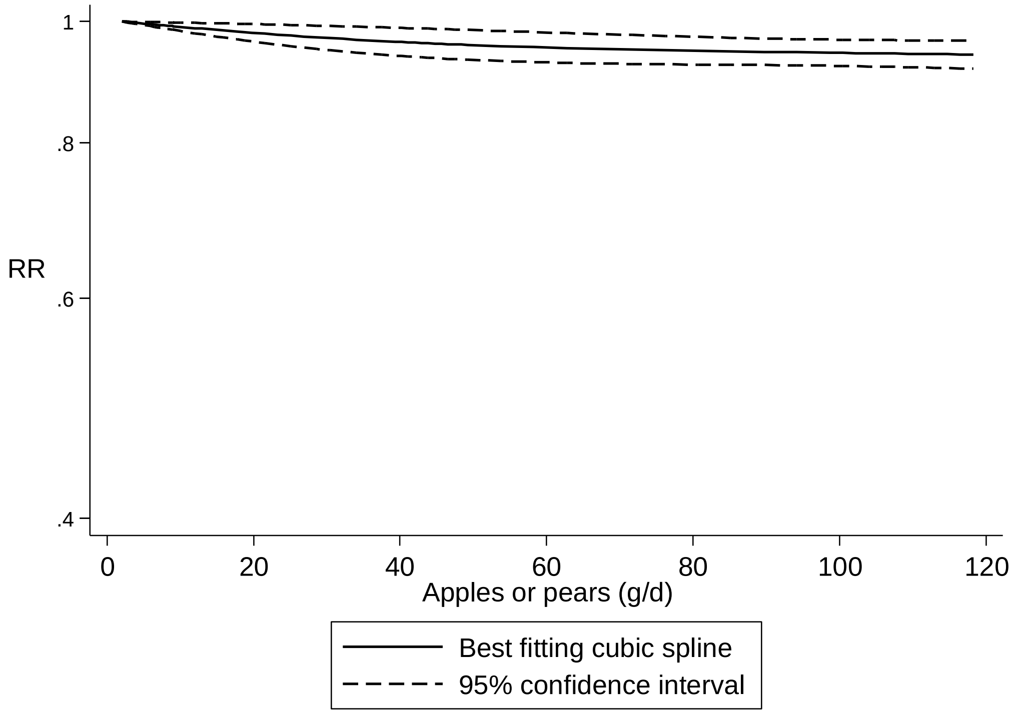

Supplementary Figure 4. Bananas and hypertension, high vs. low

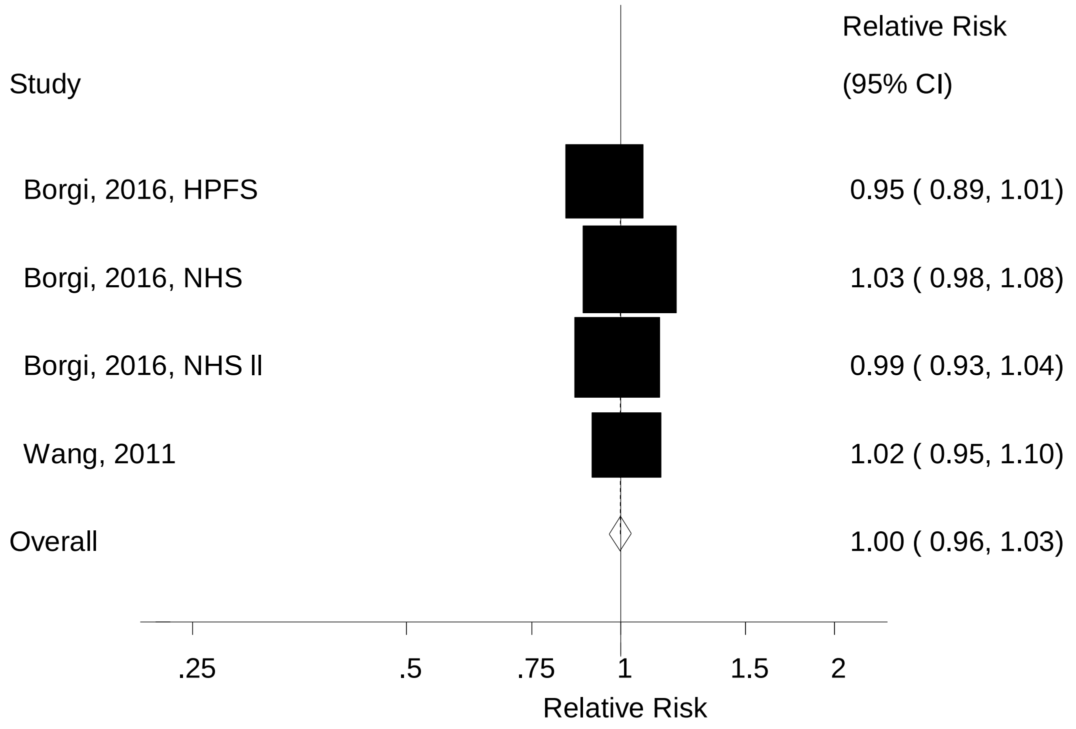

Supplementary Figure 5. Bananas and hypertension, dose-response analysis per 100 g/d

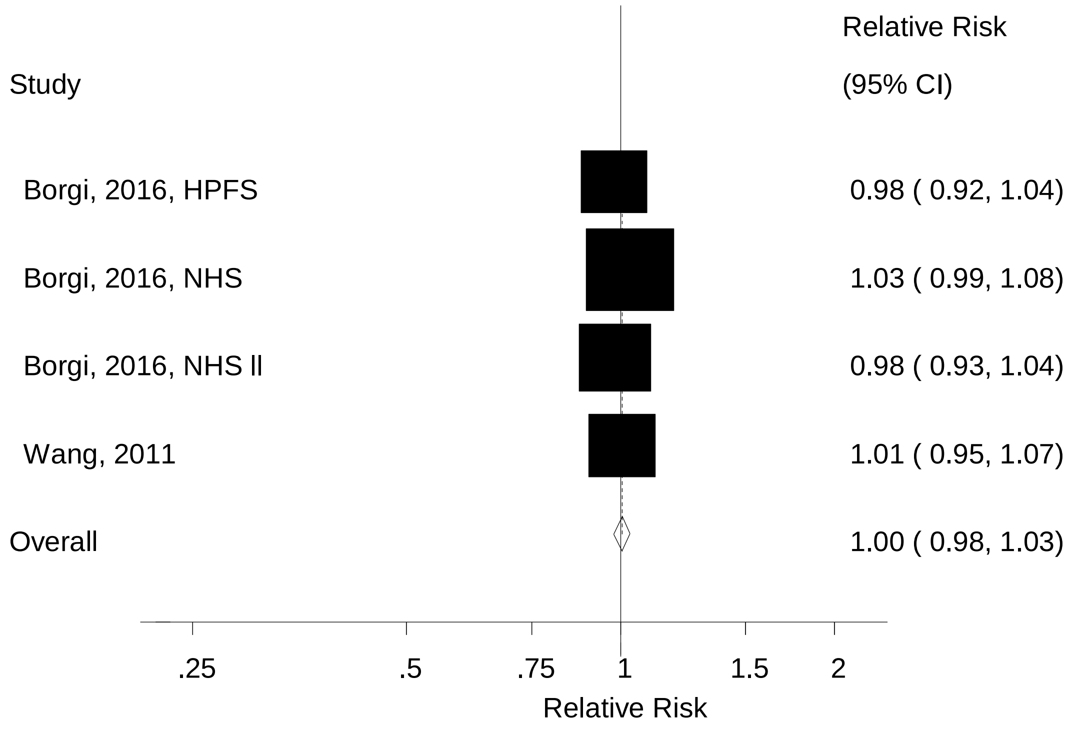

Supplementary Figure 6. Bananas and hypertension, nonlinear dose-response analysis

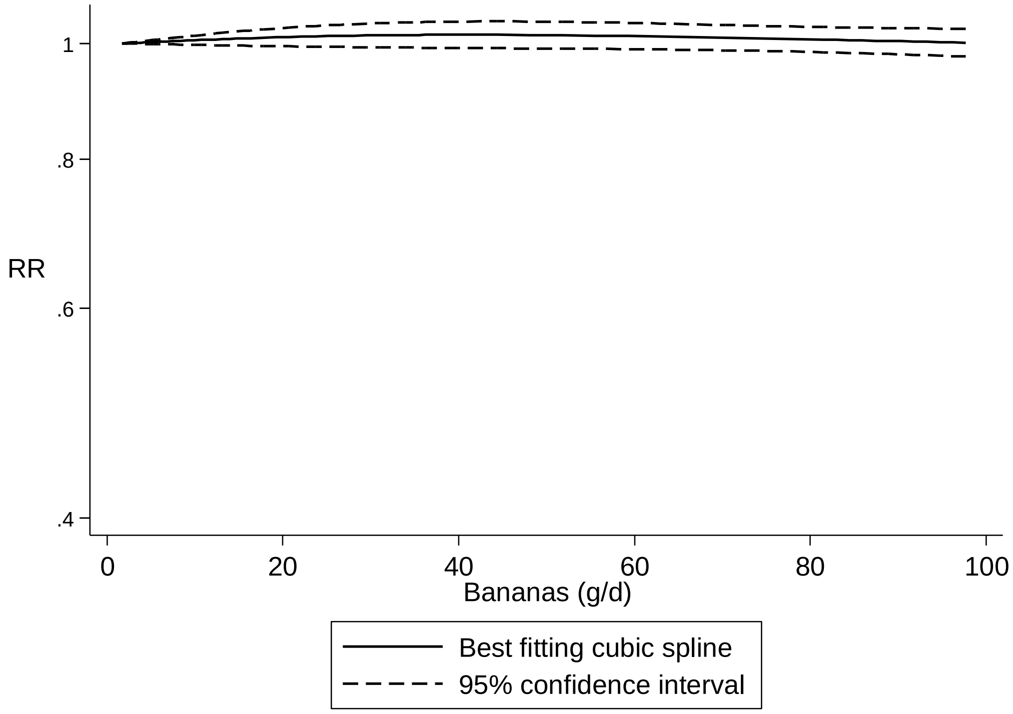

Supplementary Figure 7. Blueberries and hypertension, high vs. low

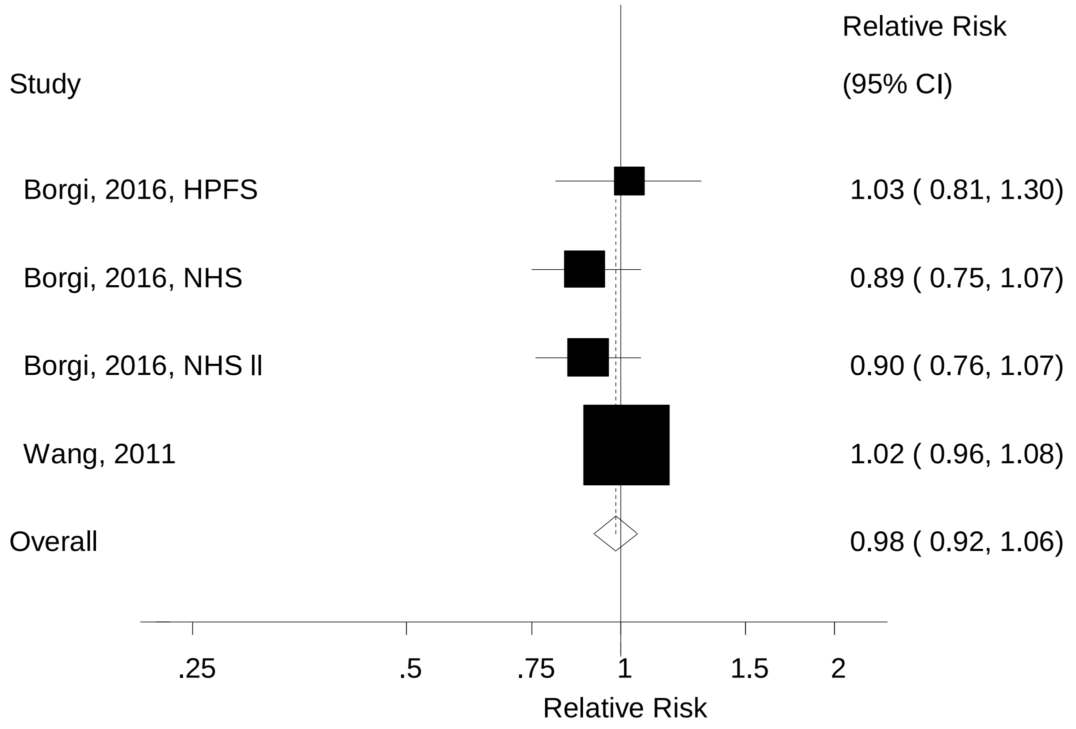

Supplementary Figure 8. Blueberries and hypertension, dose-response analysis per 100 g/d

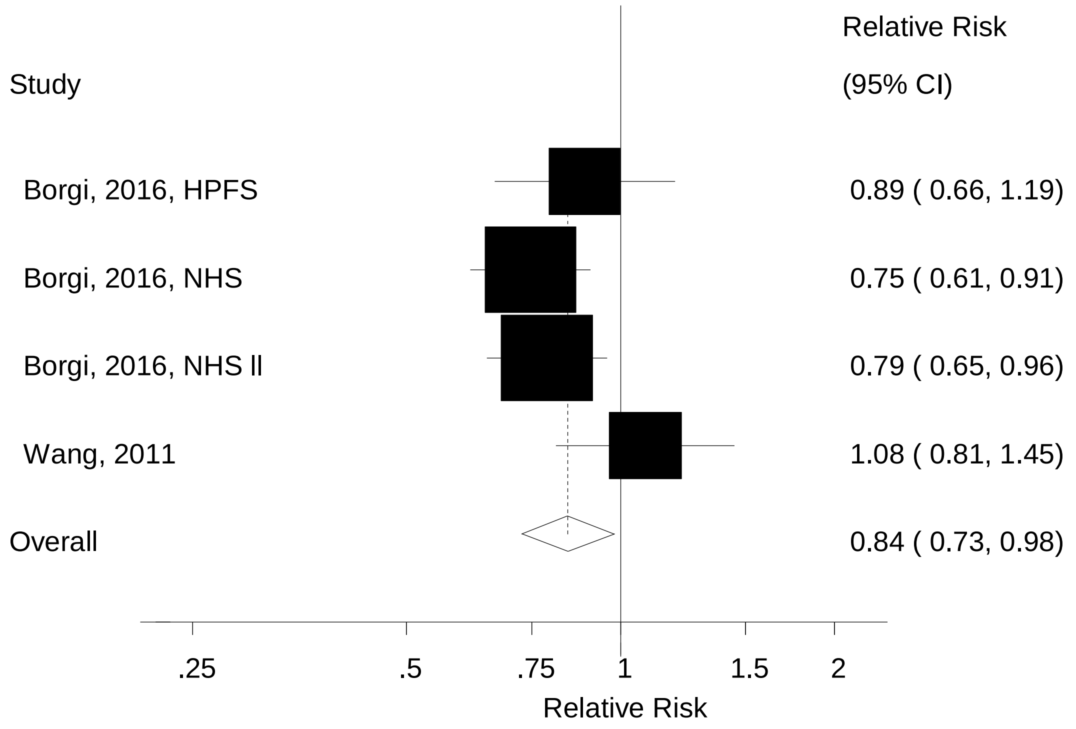

Supplementary Figure 9. Blueberries and hypertension, nonlinear dose-response analysis

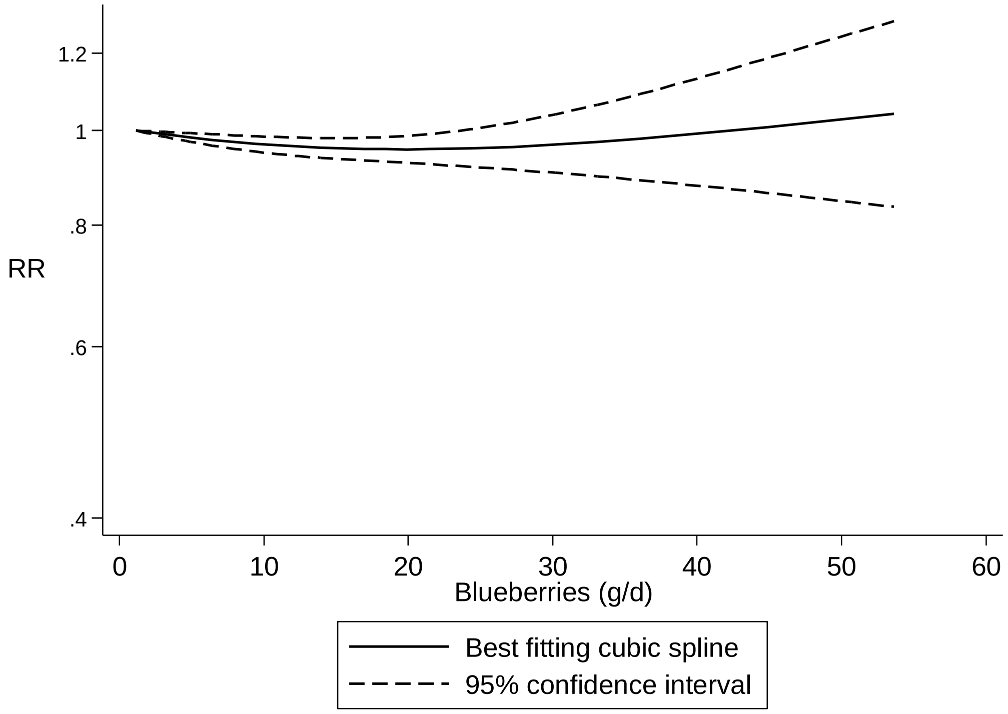

Supplementary Figure 10. Cantaloupe and hypertension, high vs. low

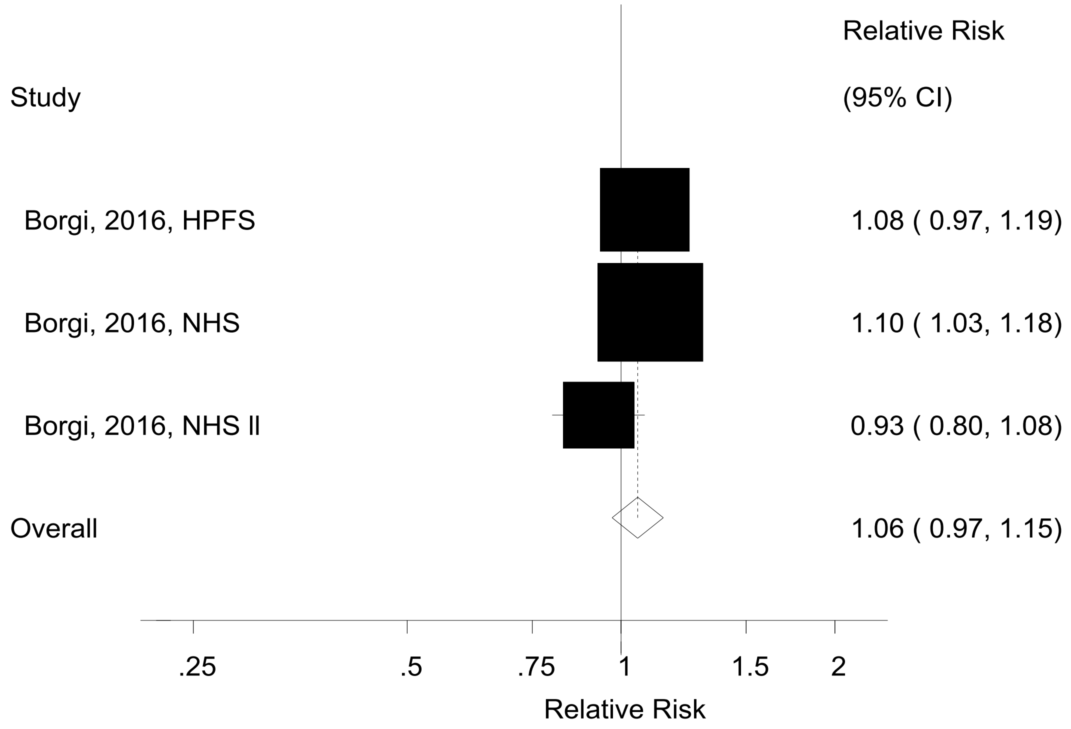

Supplementary Figure 11. Cantaloupe and hypertension, dose-response analysis per 100 g/d

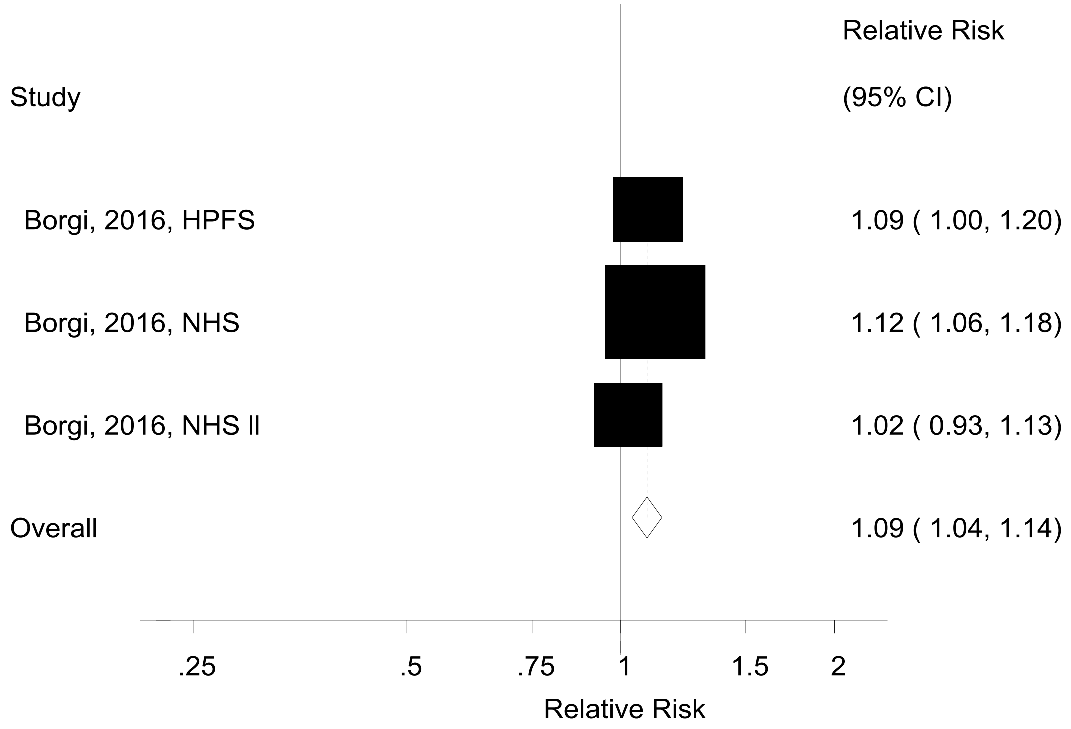

Supplementary Figure 12. Cantaloupe and hypertension, nonlinear dose-response analysis

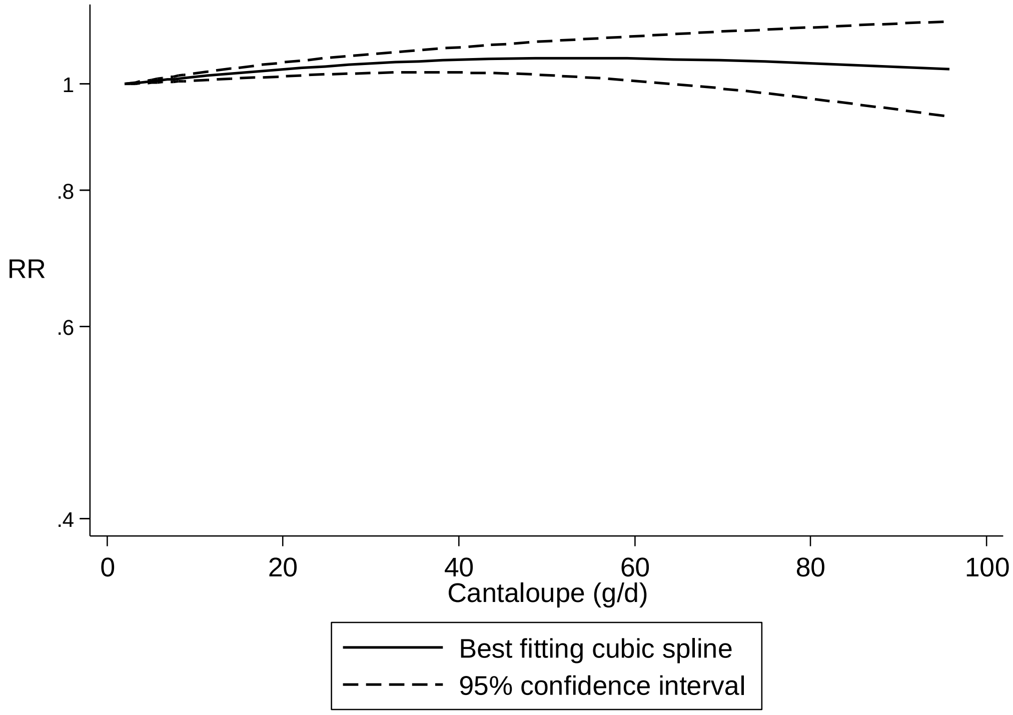

Supplementary Figure 13. Oranges and hypertension, high vs. low

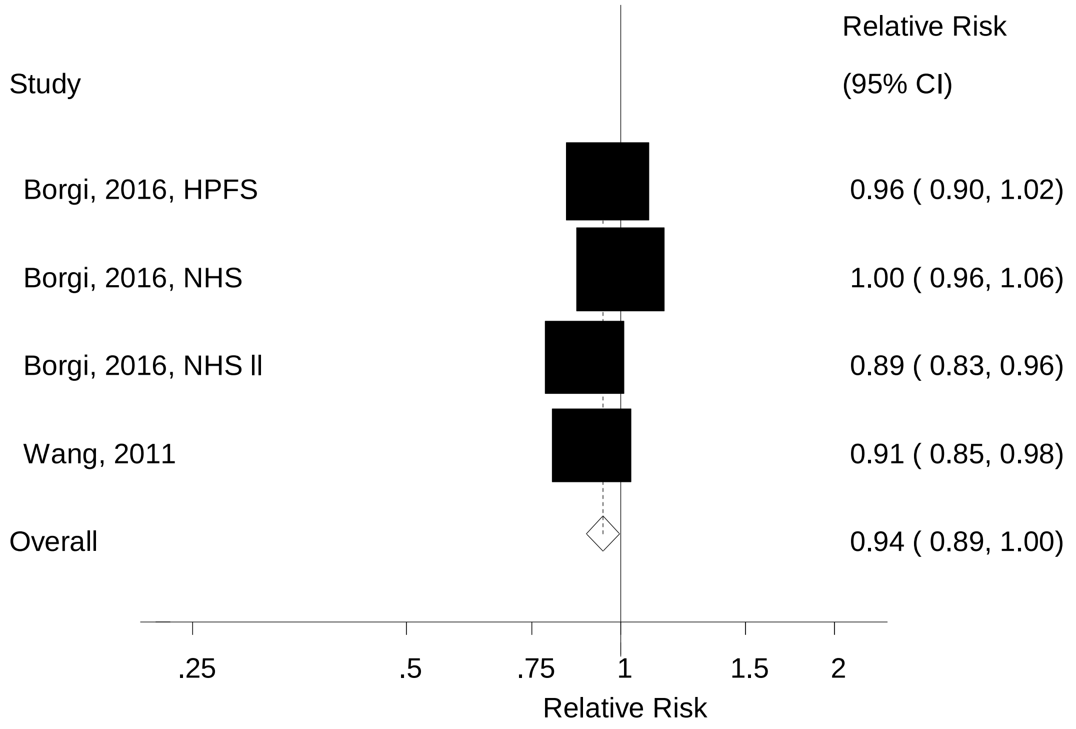

Supplementary Figure 14. Oranges and hypertension, dose-response per 100 g/d

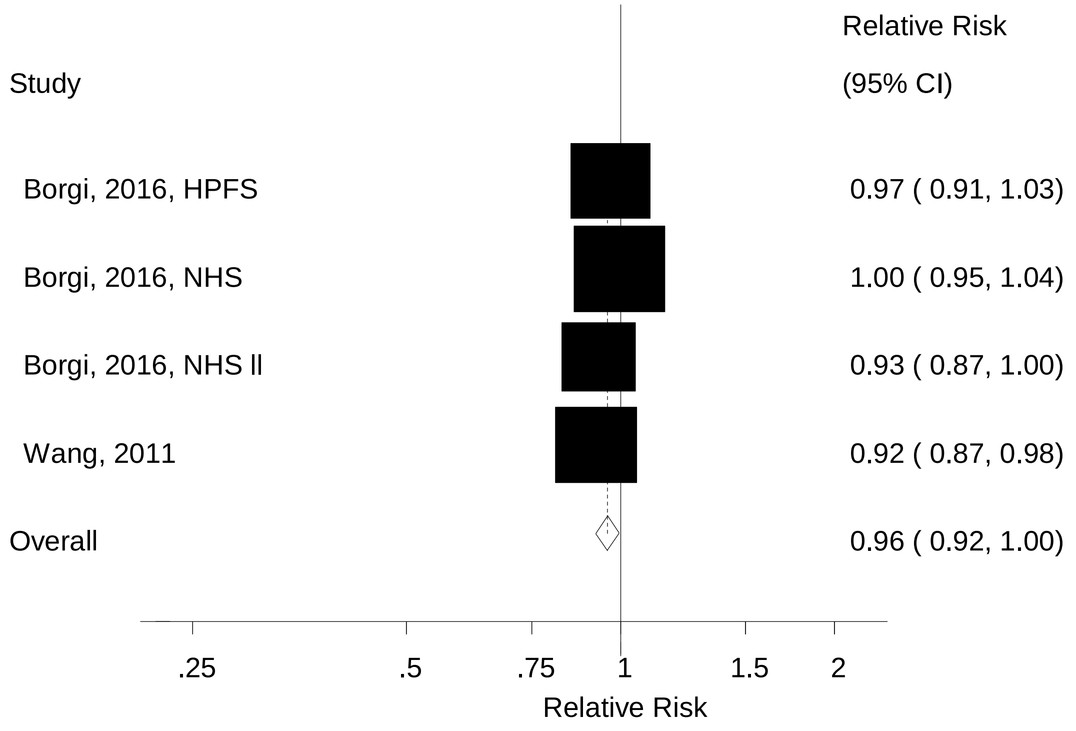

Supplementary Figure 15. Oranges and hypertension, nonlinear dose-response analysis

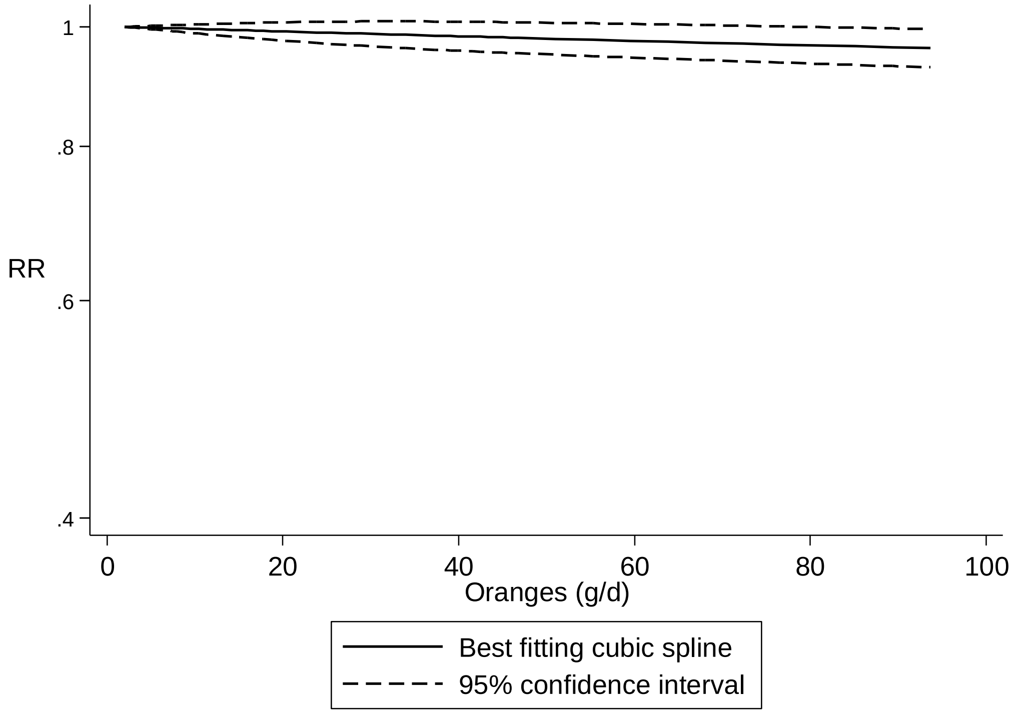

Supplementary Figure 16. Peaches, apricots or plums and hypertension, high vs. low

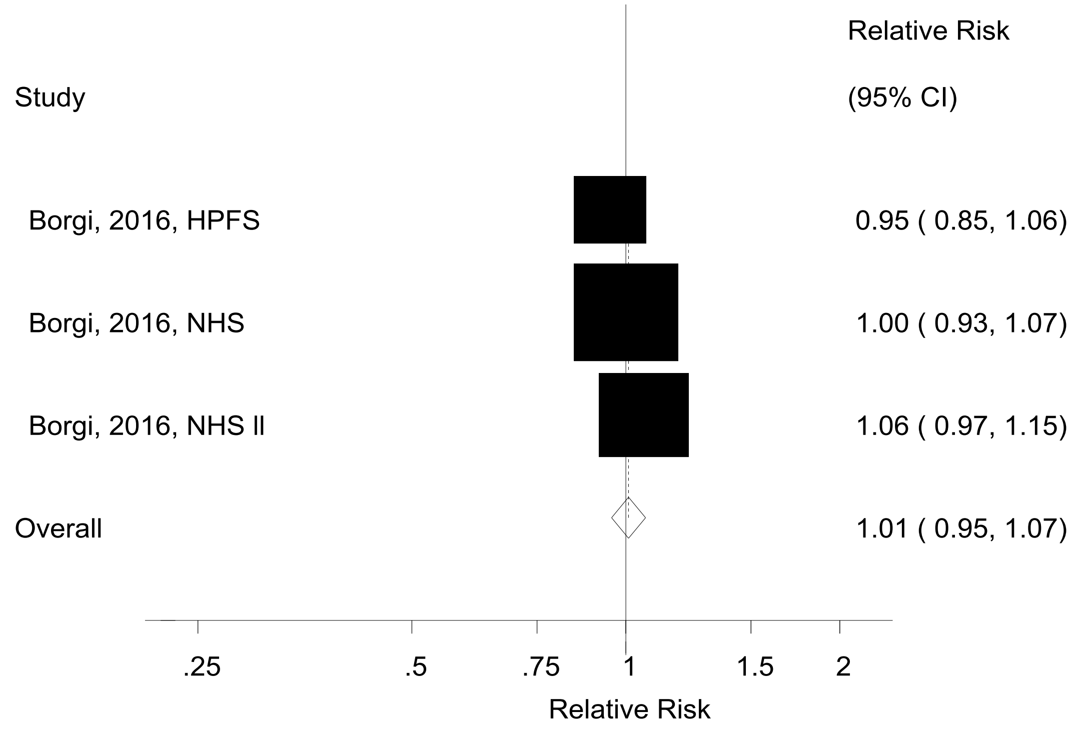

Supplementary Figure 17. Peaches, apricots or plums and hypertension, dose-response per 100 g/d

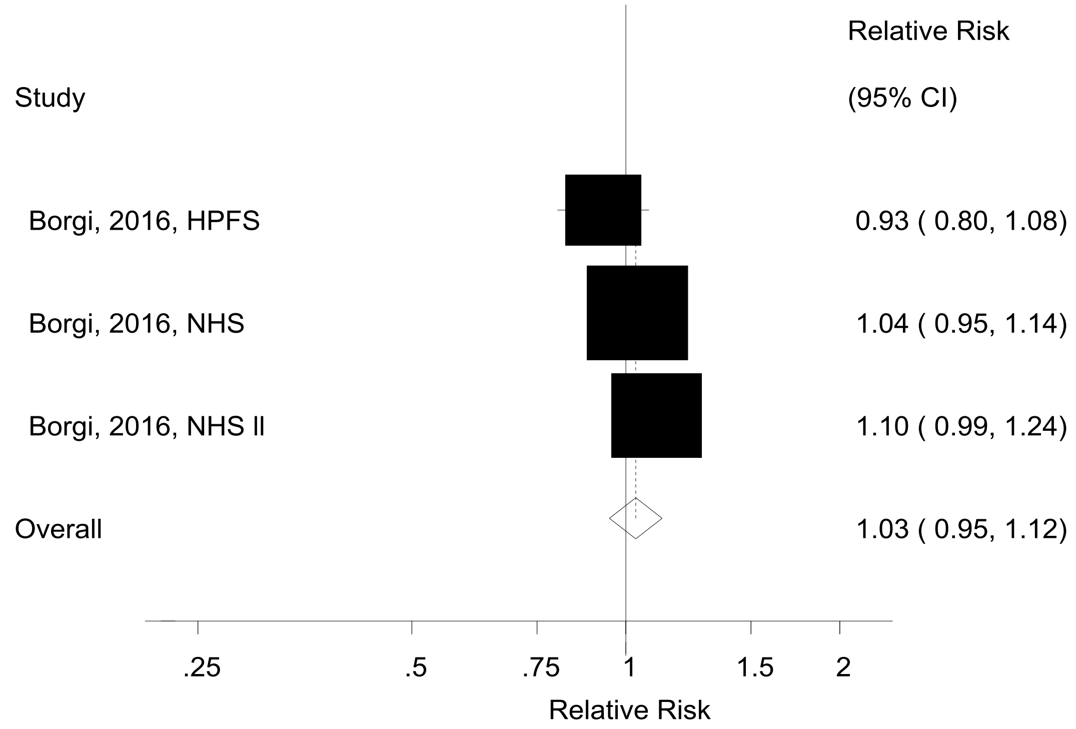

Supplementary Figure 18. Peaches, apricots or plums and hypertension, nonlinear dose-response analysis

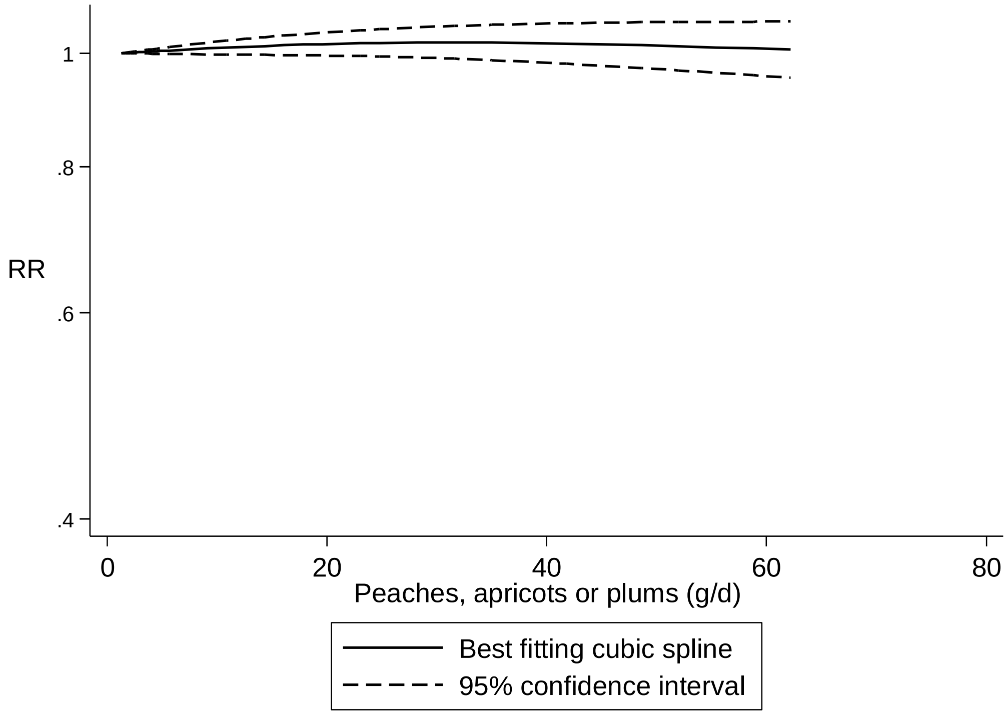

Supplementary Figure 19. Prunes and hypertension, high vs. low

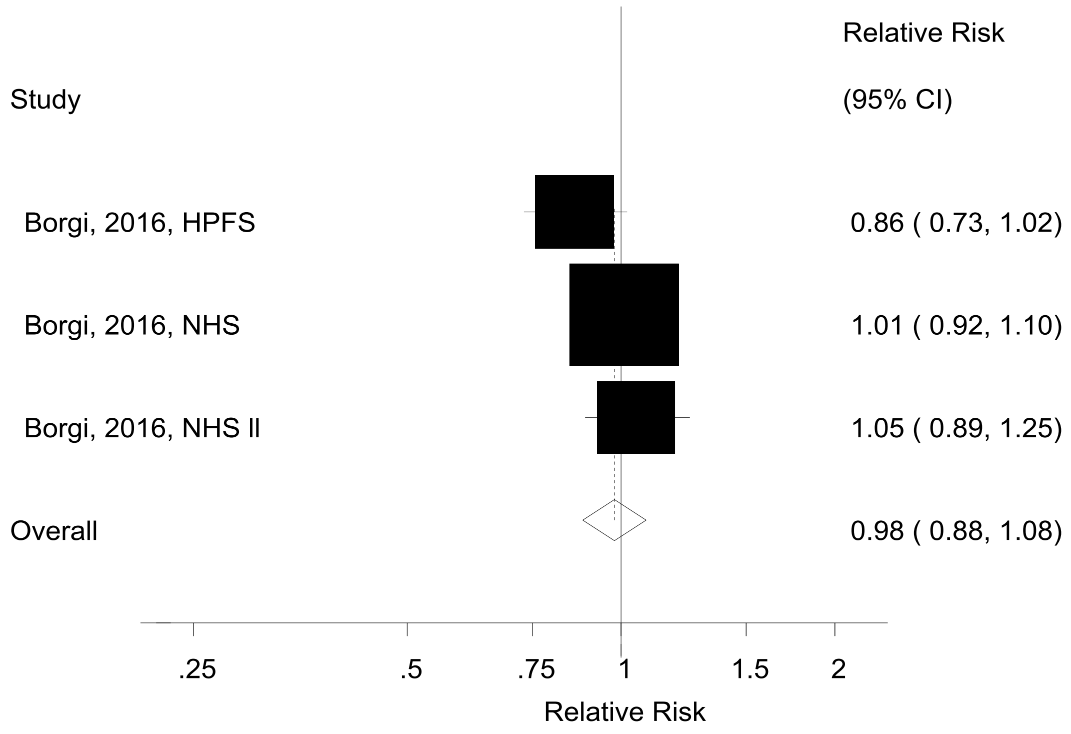

Supplementary Figure 20. Prunes and hypertension, dose-response per 100 g/d

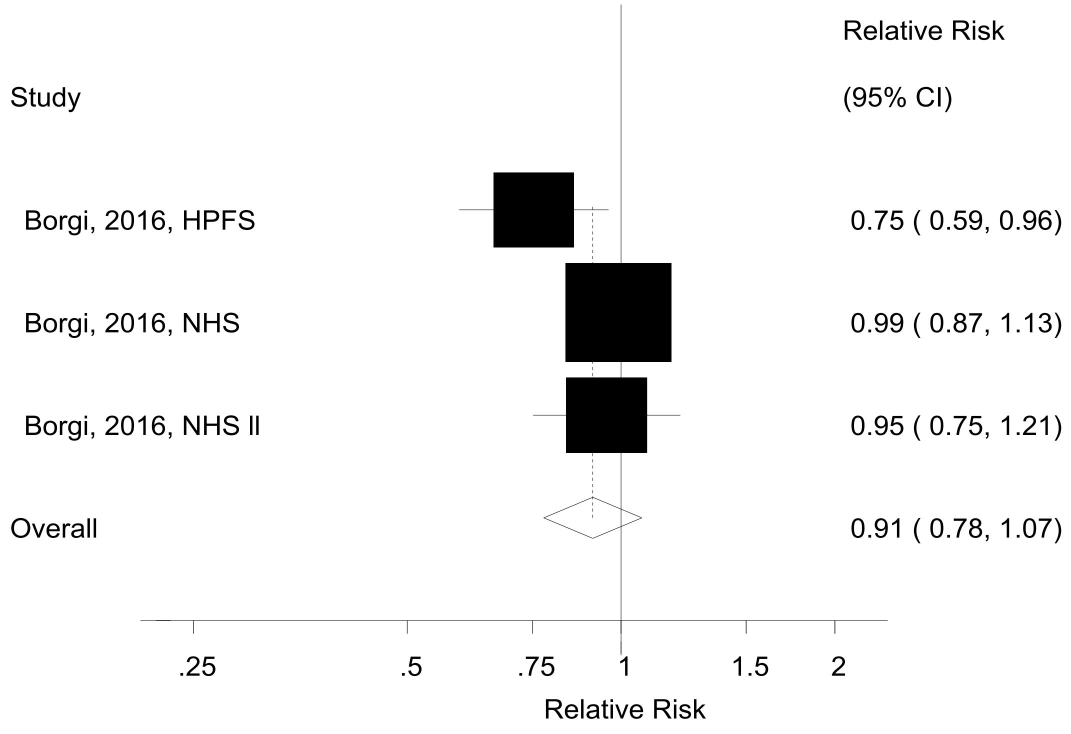

Supplementary Figure 21. Prunes and hypertension, nonlinear dose-response analysis

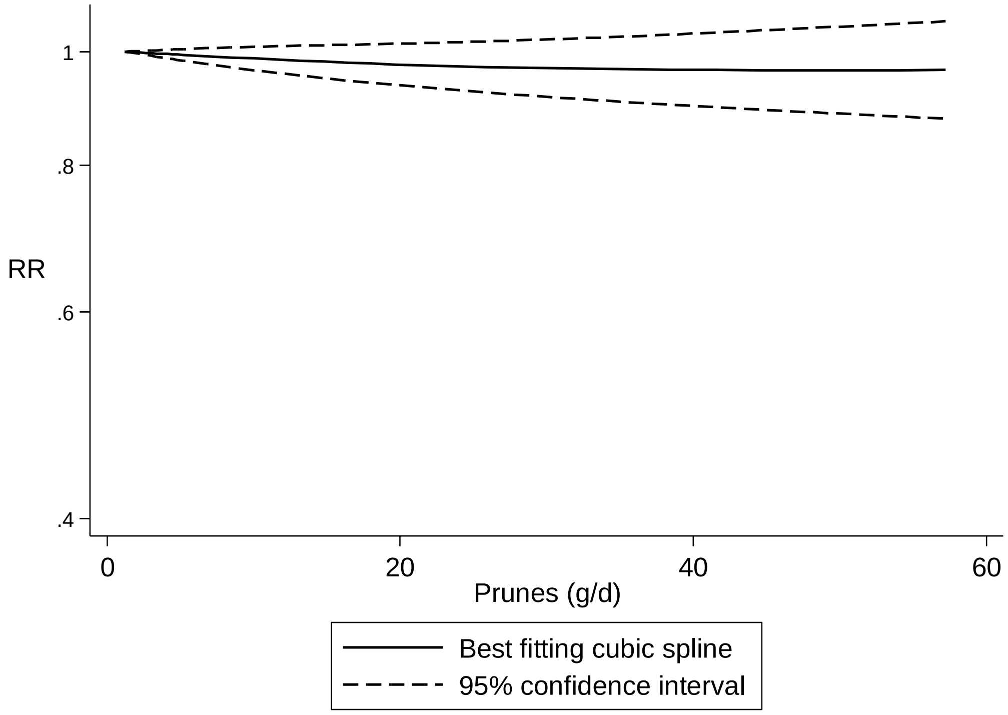

Supplementary Figure 22. Raisins or grapes and hypertension, high vs. low

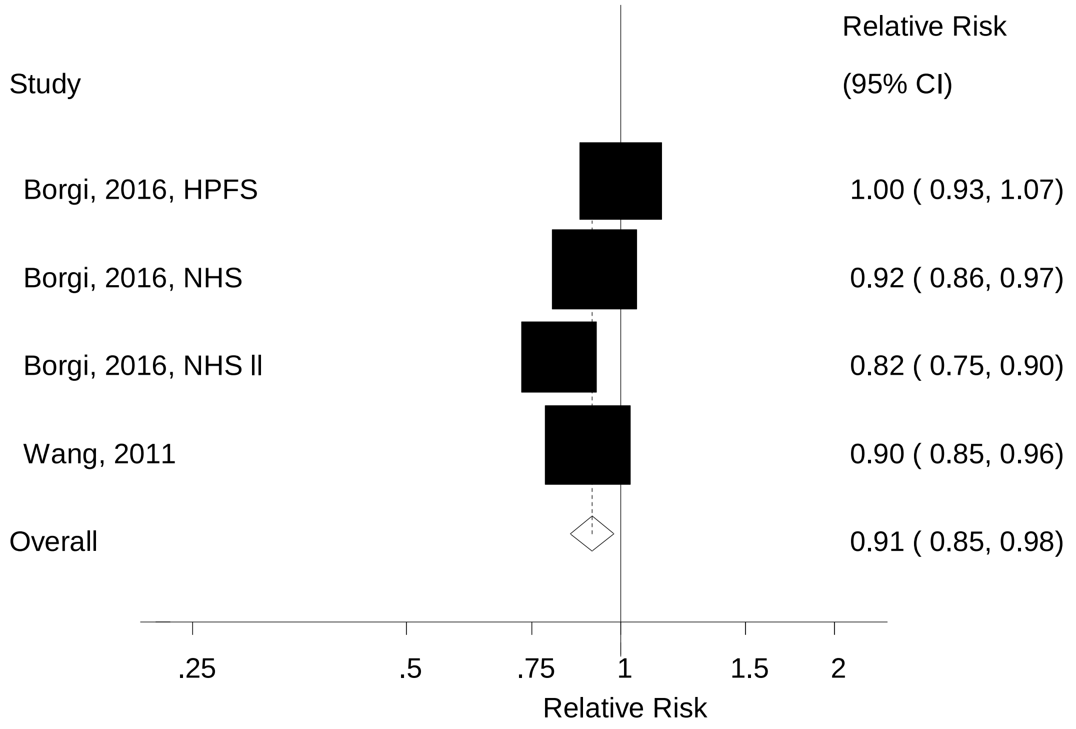

Supplementary Figure 23. Raisins or grapes and hypertension, dose-response per 100 g/d

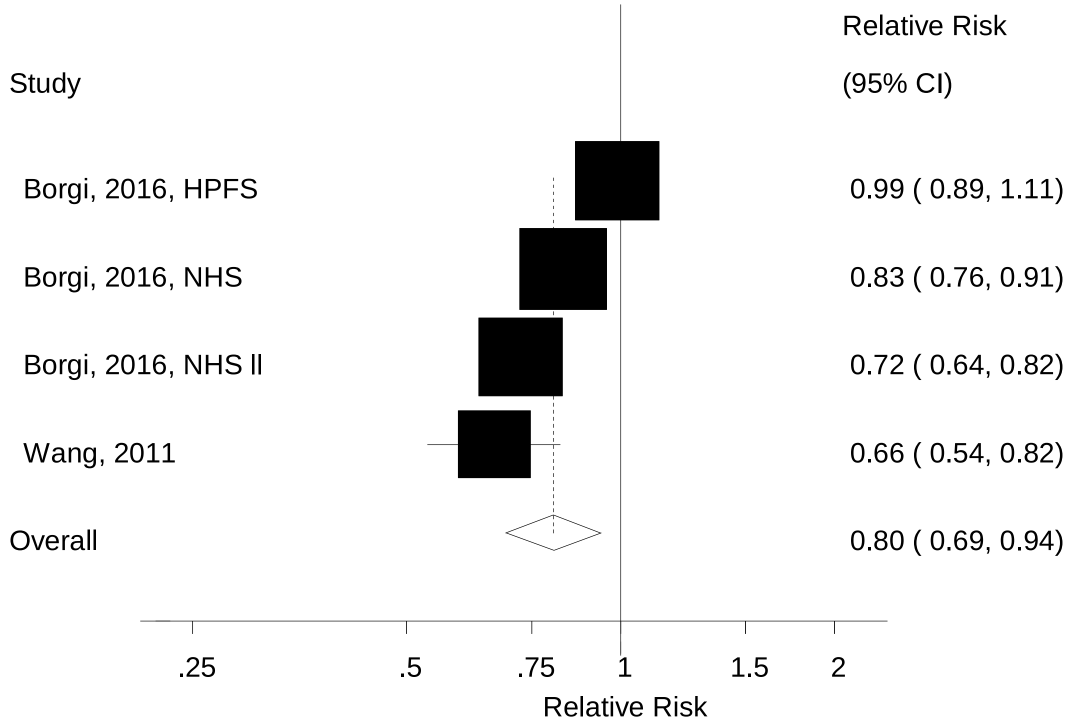

Supplementary Figure 24. Raisins or grapes and hypertension, nonlinear dose-response analysis

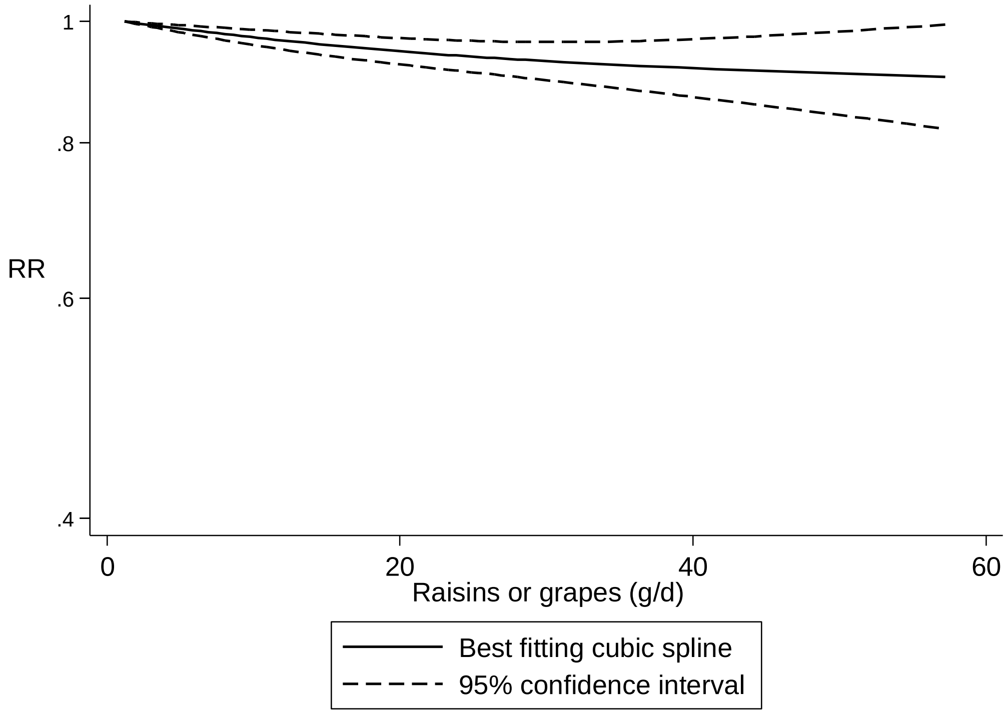

Supplementary Figure 25. Strawberries and hypertension, high vs. low

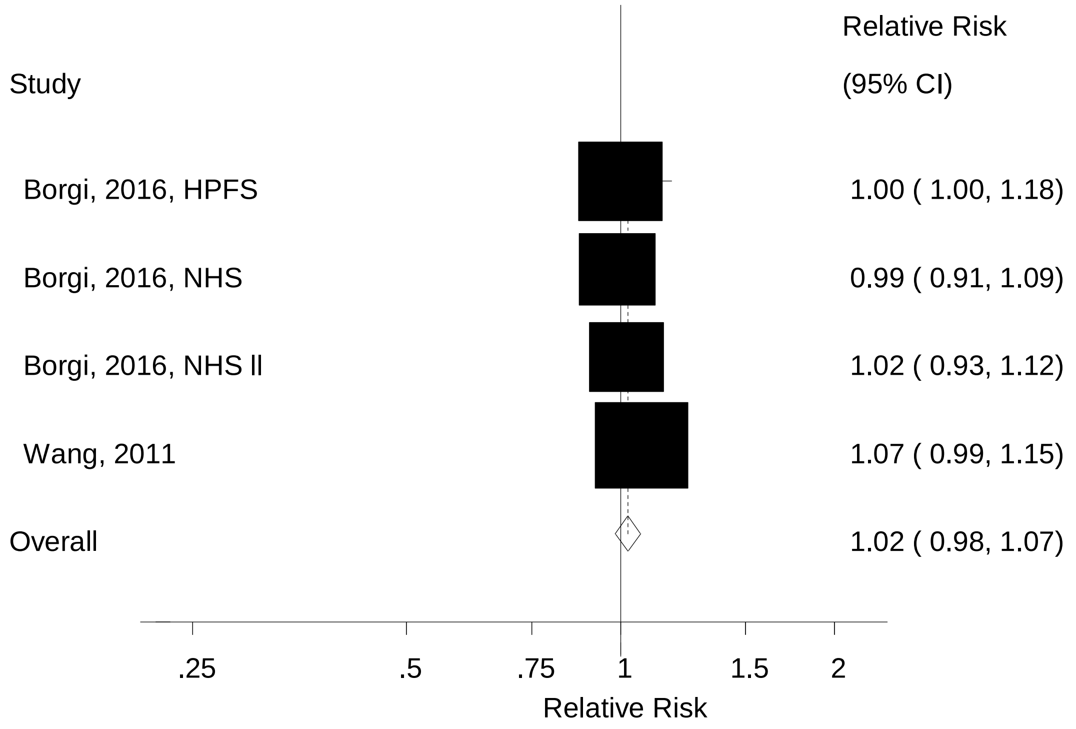

Supplementary Figure 26. Strawberries and hypertension, dose-response per 100 g/d

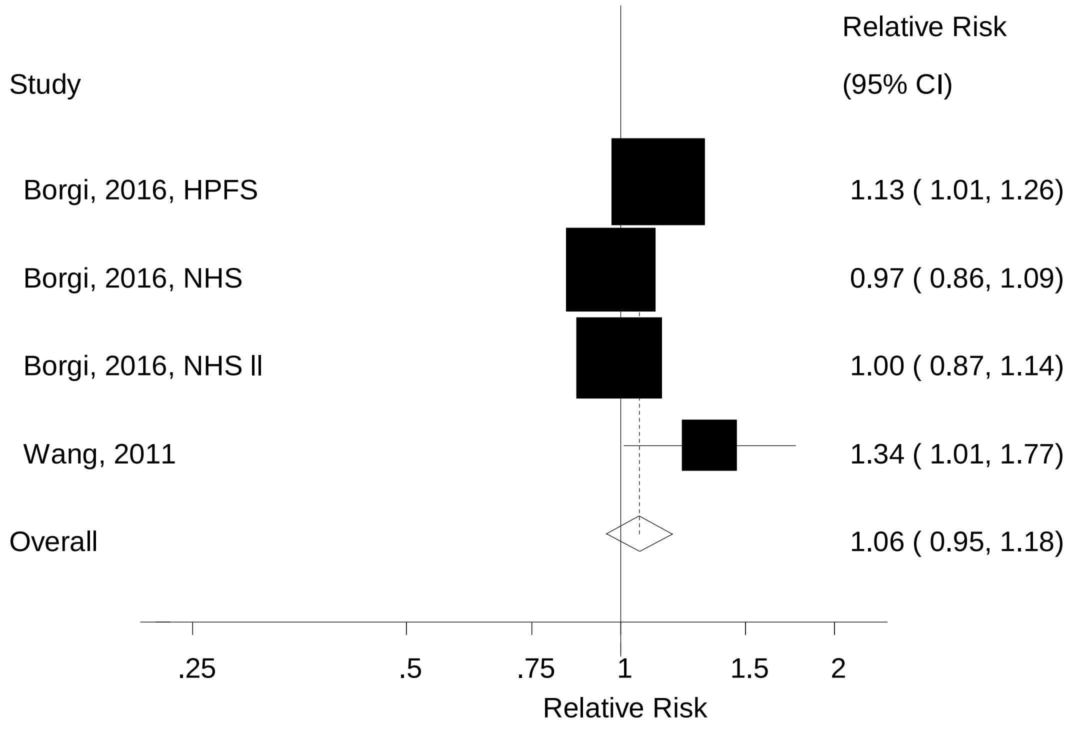

Supplementary Figure 27. Strawberries and hypertension, nonlinear dose-response analysis

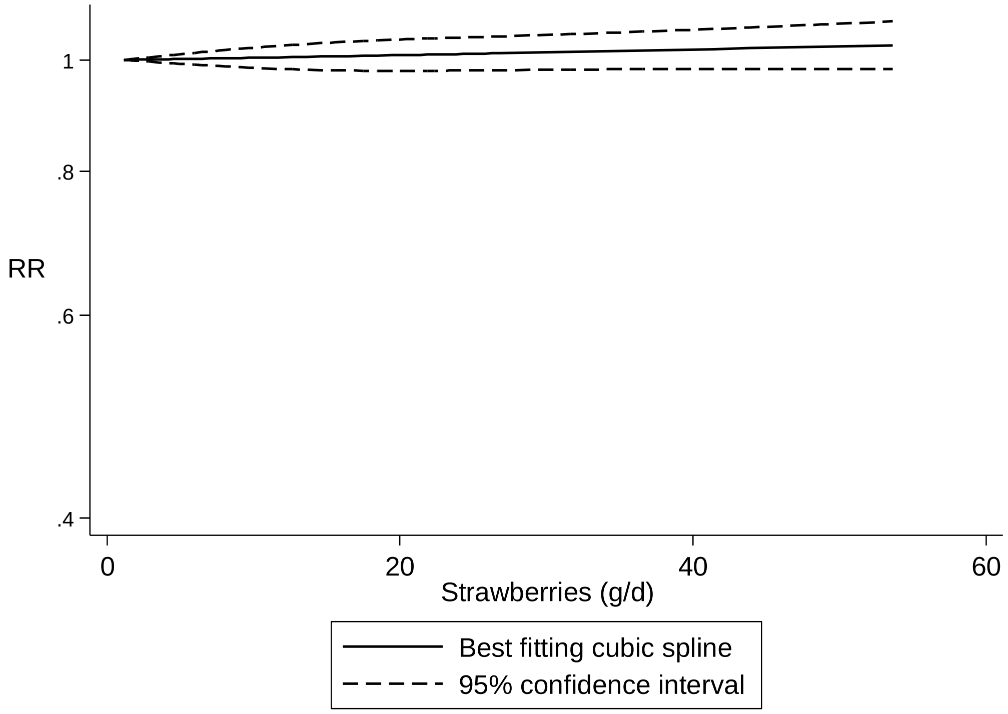

Supplementary Figure 28. Avocado and hypertension, high vs. low

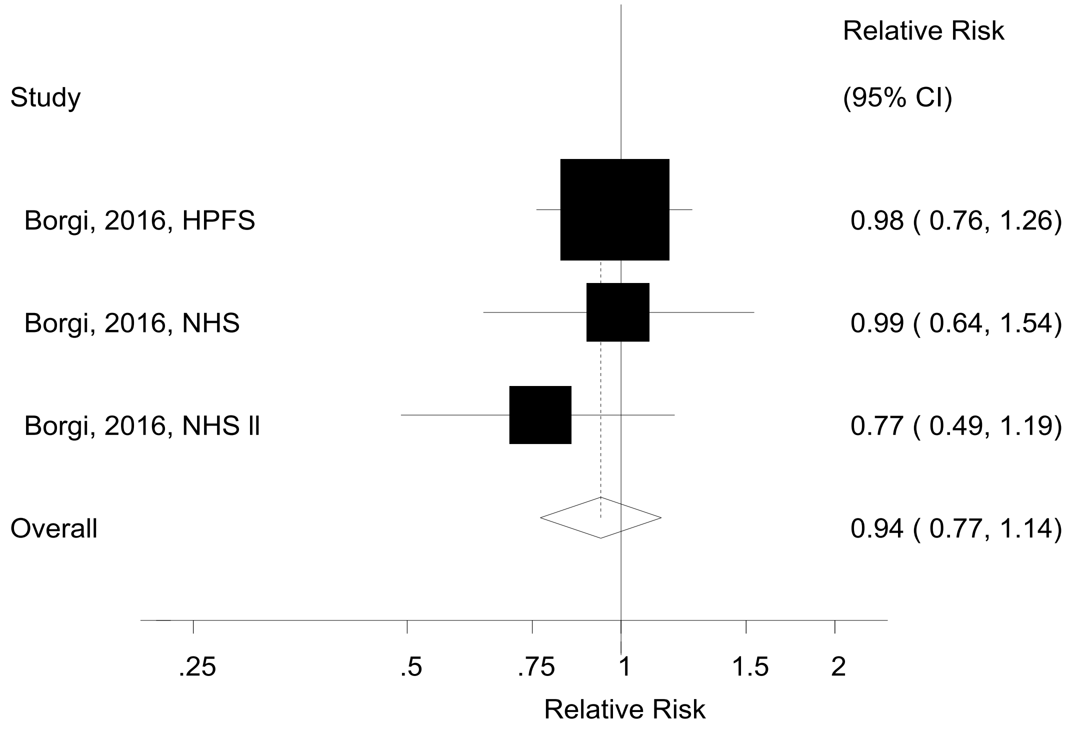

Supplementary Figure 29. Avocado and hypertension, dose-response analysis per 100 g/d

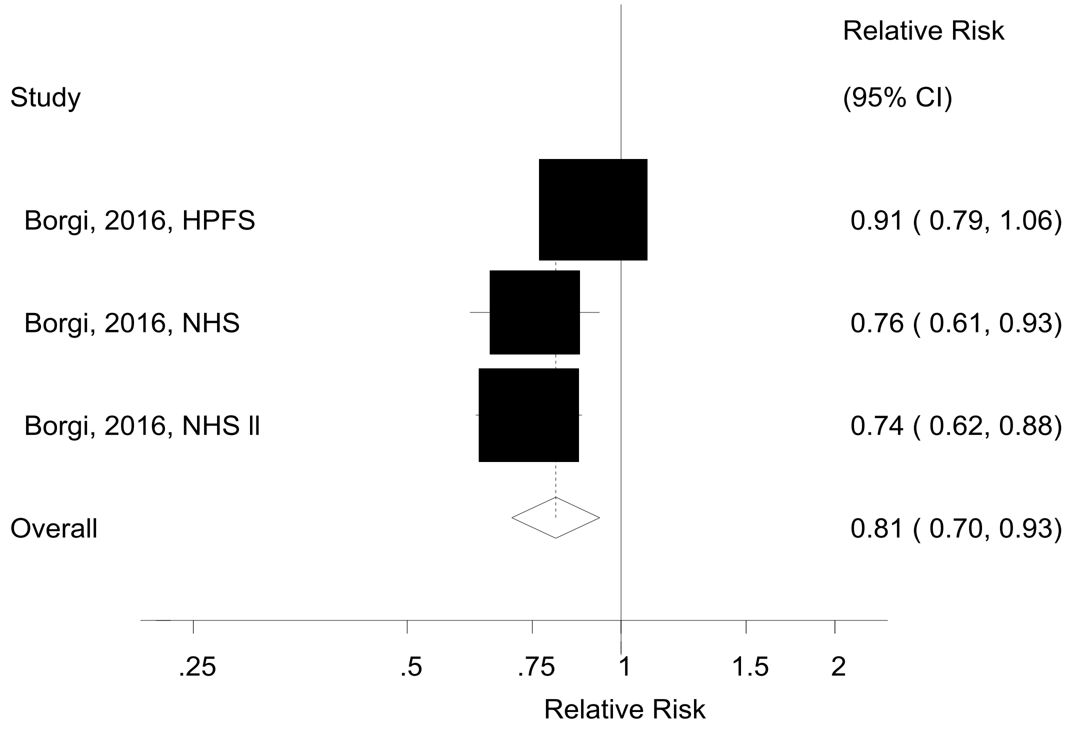

Supplementary Figure 30. Avocado and hypertension, nonlinear dose-response analysis

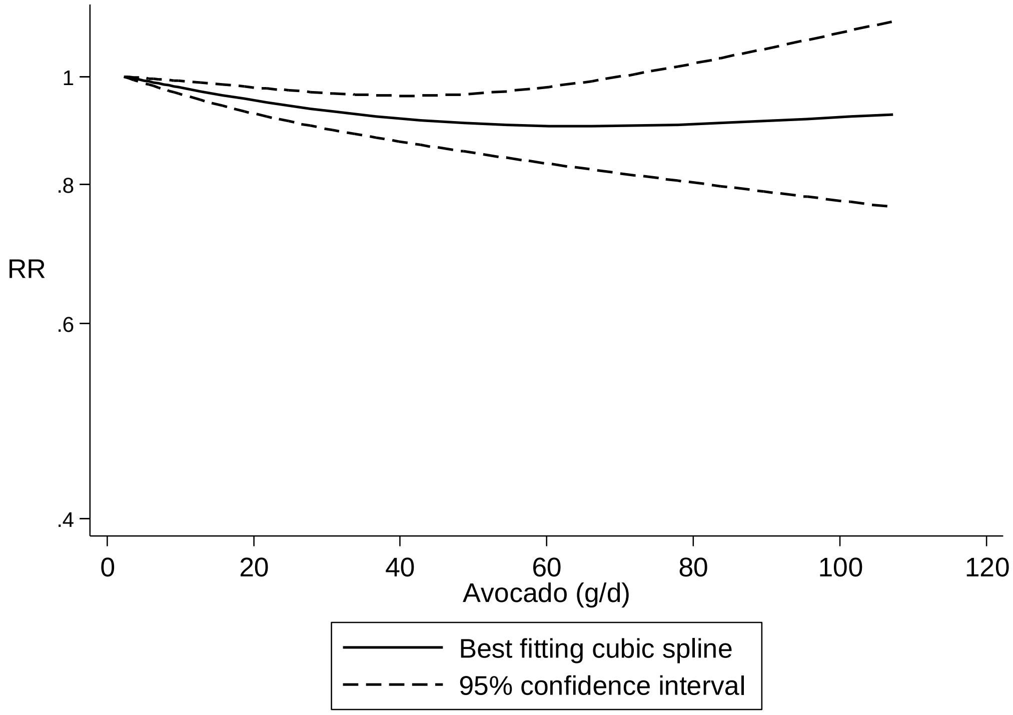

Supplementary Figure 31. Broccoli and hypertension, high vs. low

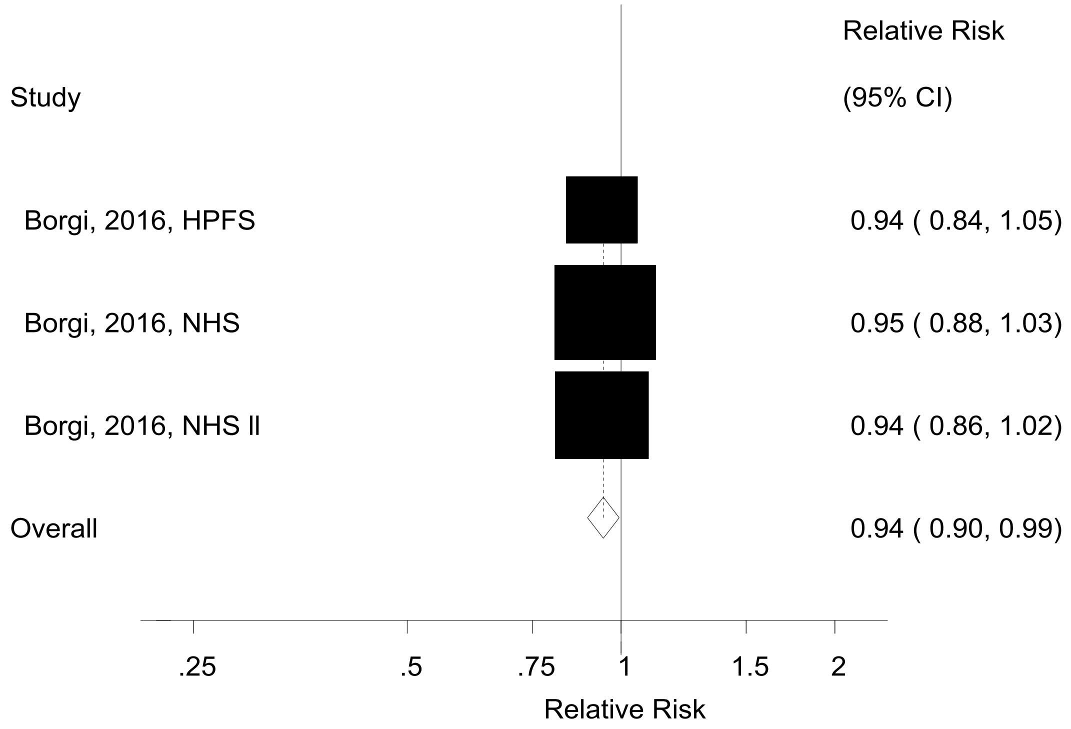

Supplementary Figure 32. Broccoli and hypertension, dose-response analysis per 100 g/d

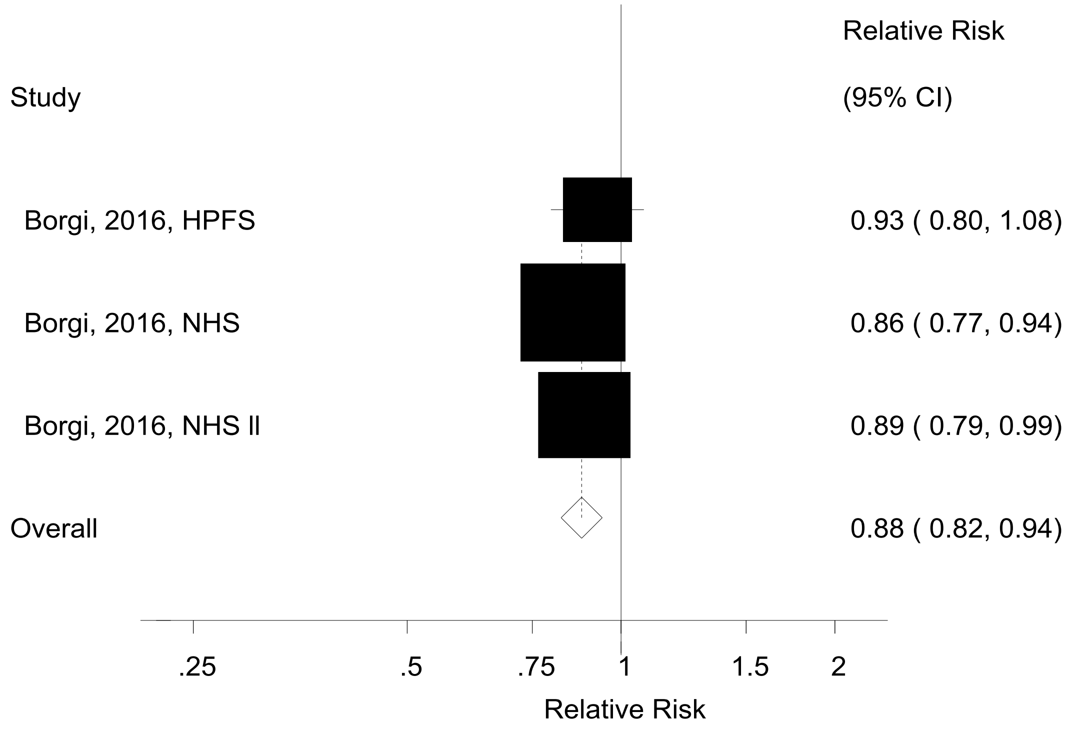

Supplementary Figure 33. Broccoli and hypertension, nonlinear dose-response analysis

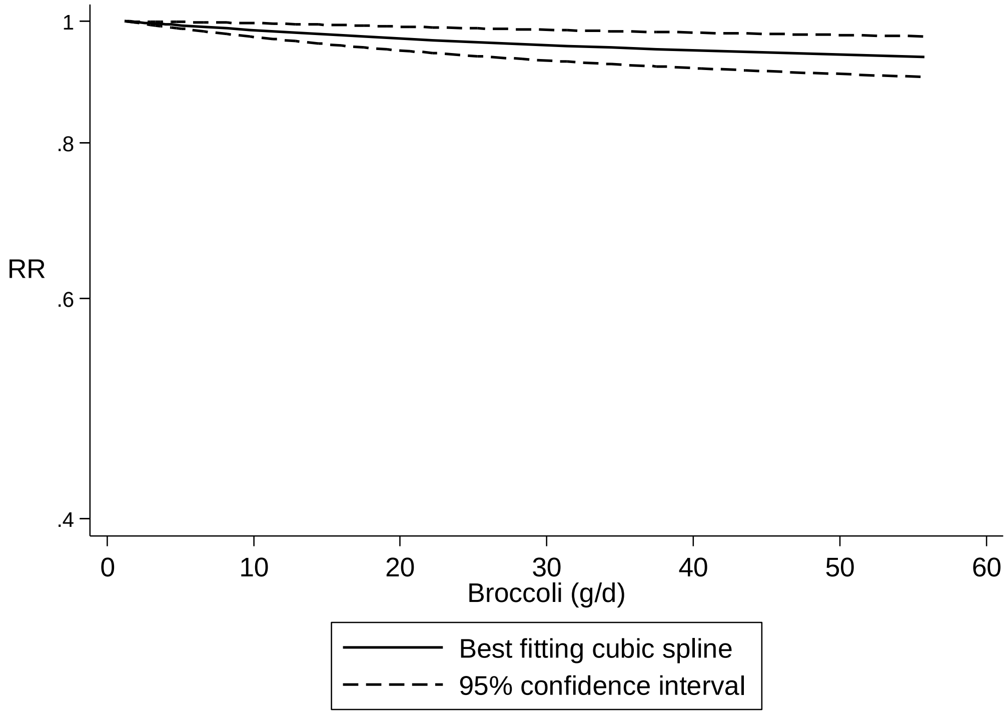

Supplementary Figure 34. Brussel sprouts and hypertension, high vs. low

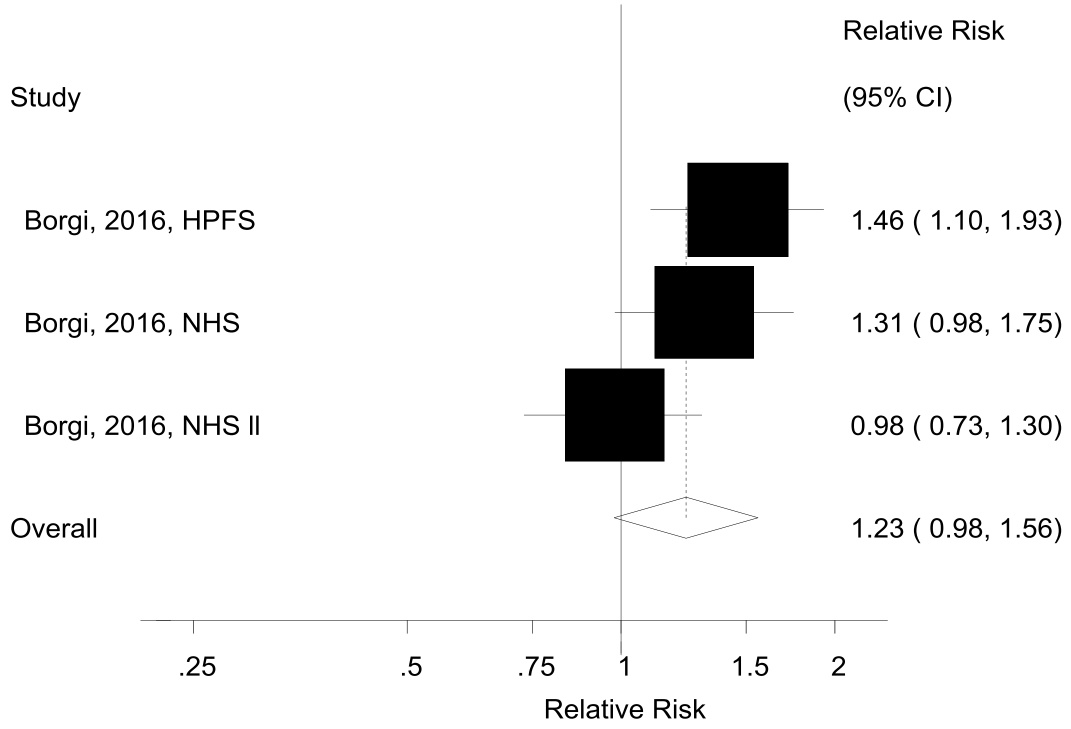

Supplementary Figure 35. Brussel sprouts and hypertension, dose-response analysis per 100 g/d

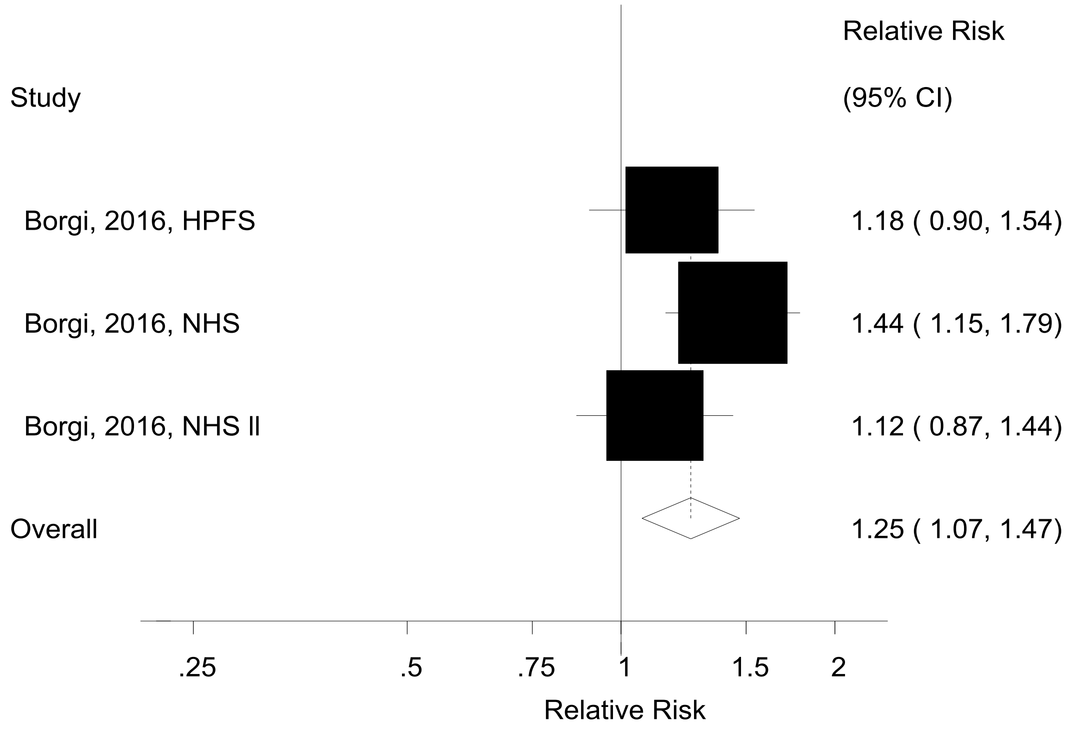

Supplementary Figure 36. Brussel sprouts and hypertension, nonlinear dose-response analysis

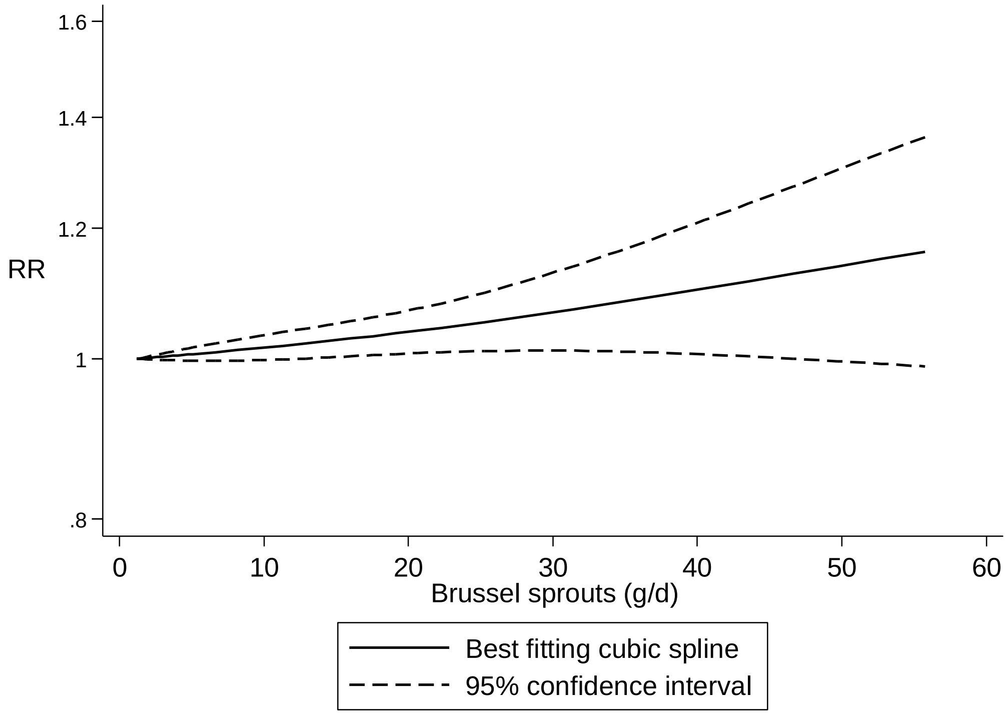

Supplementary Figure 37. Cabbage and hypertension, high vs. low

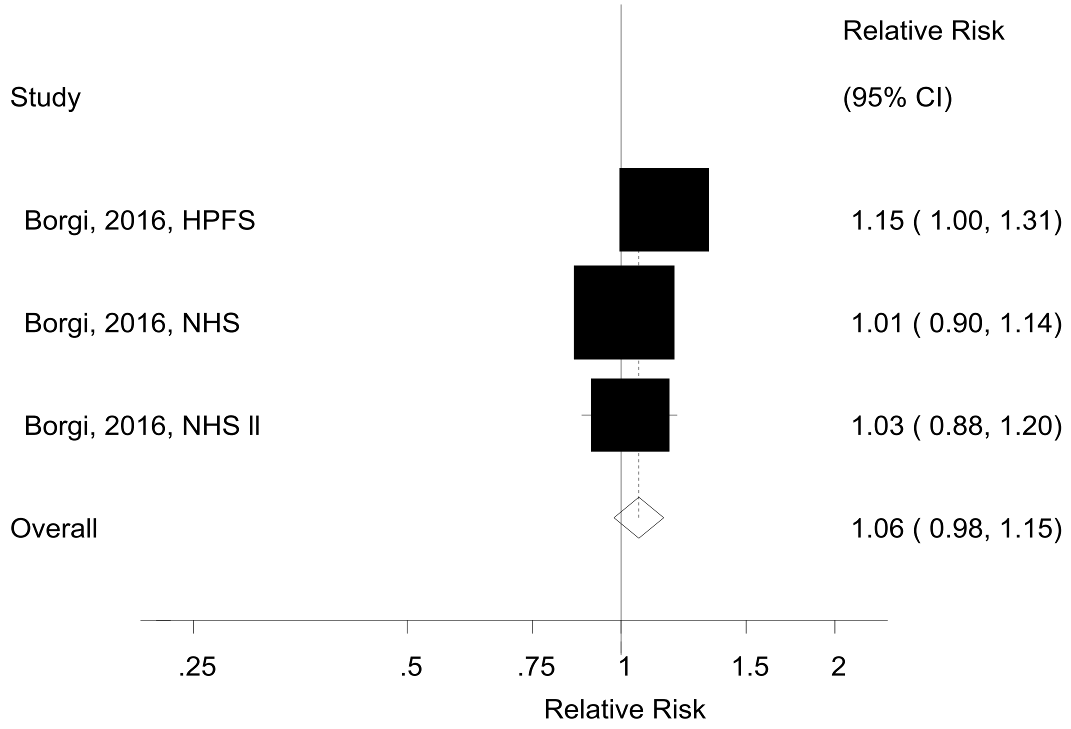

Supplementary Figure 38. Cabbage and hypertension, dose-response analysis per 100 g/d

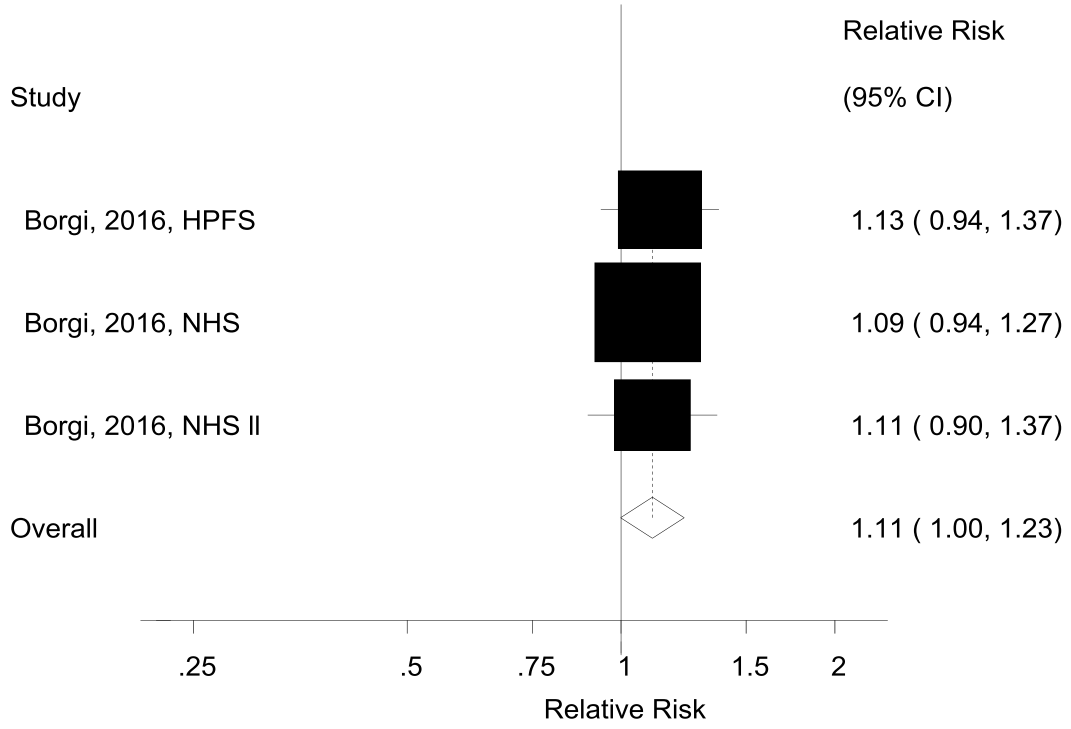

Supplementary Figure 39. Cabbage and hypertension, nonlinear dose-response analysis

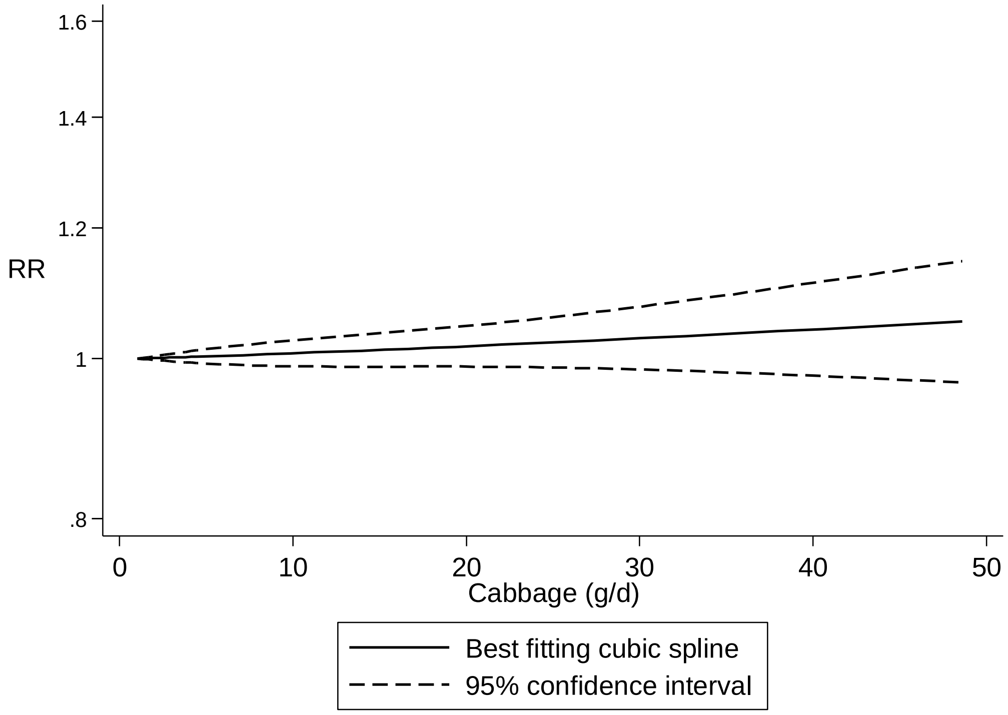

Supplementary Figure 40. Carrots and hypertension, high vs. low

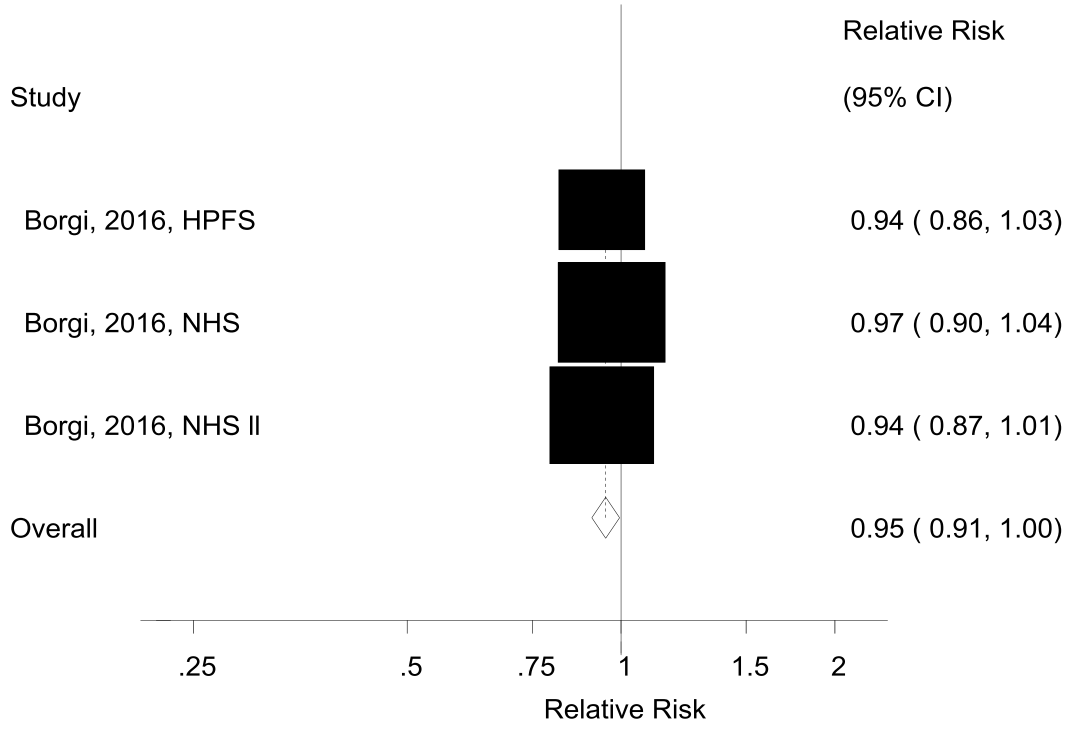

Supplementary Figure 41. Carrots and hypertension, dose-response analysis per 100 g/d

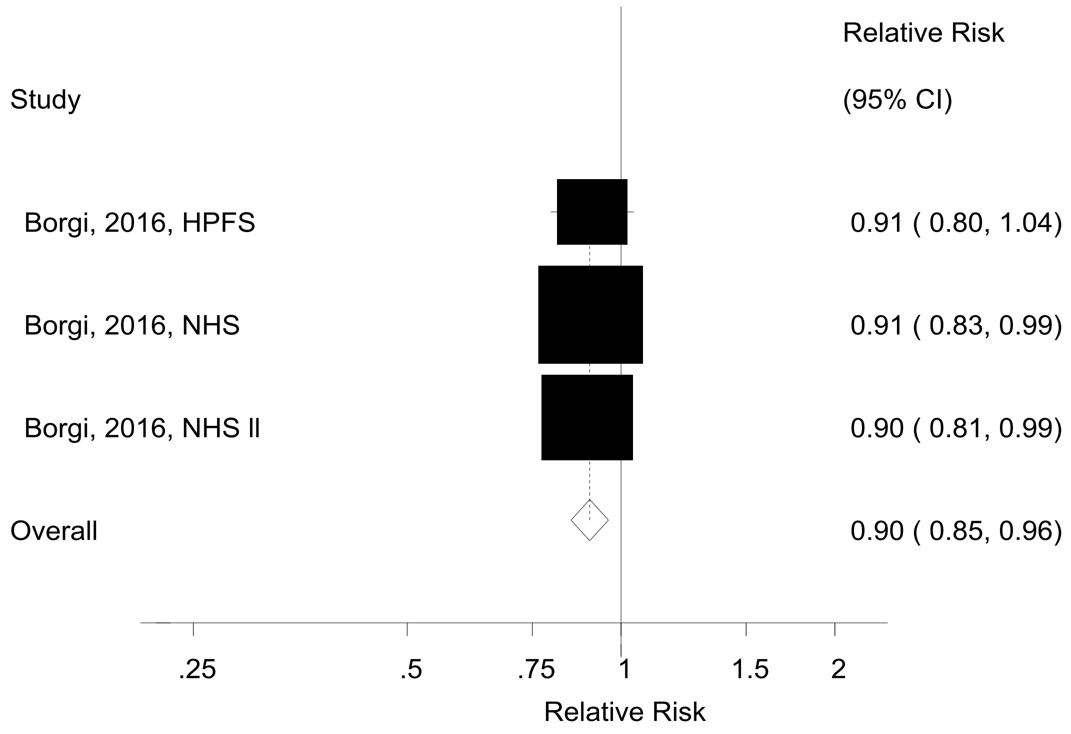

Supplementary Figure 42. Carrots and hypertension, nonlinear dose-response analysis

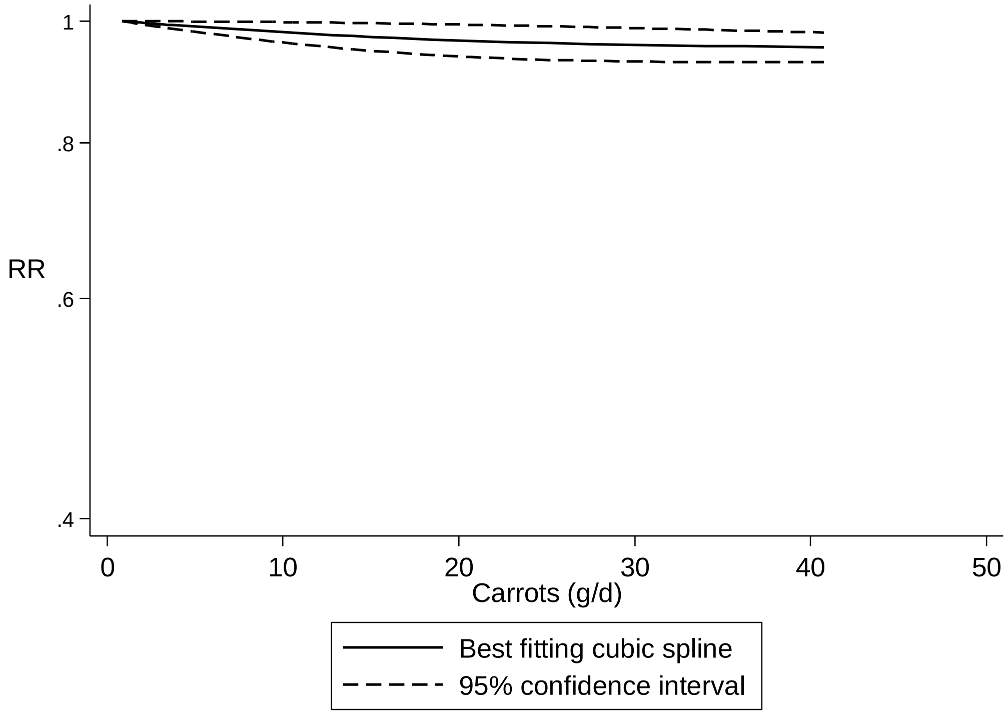

Supplementary Figure 43. Cauliflower and hypertension, high vs. low

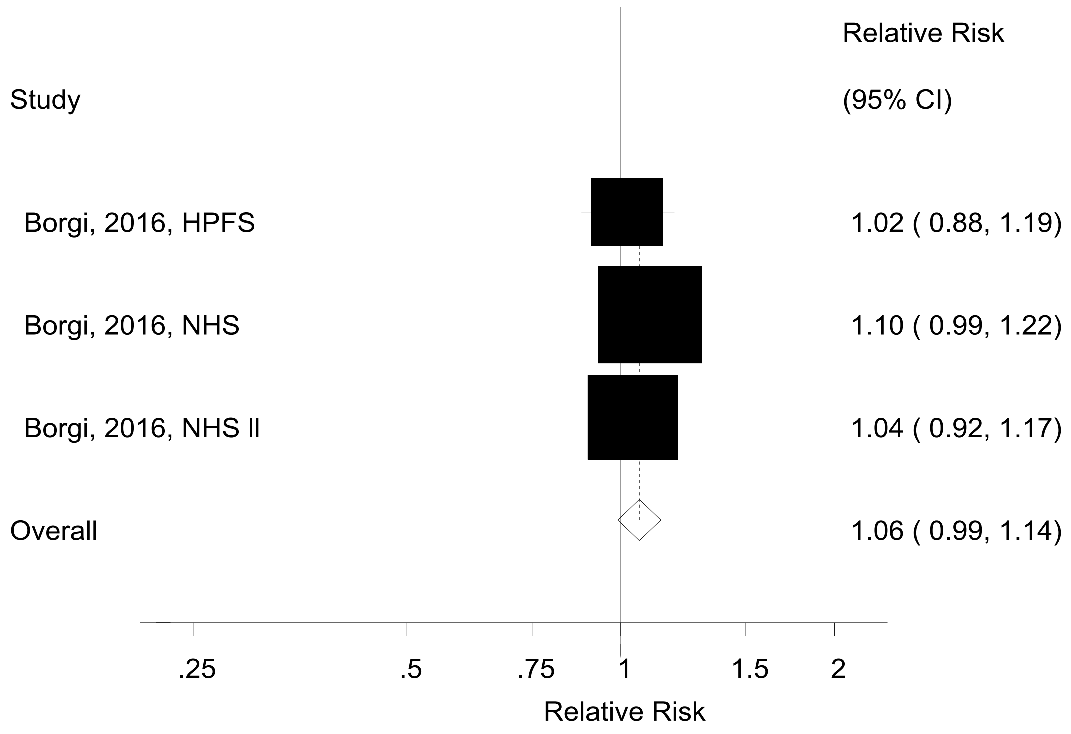

Supplementary Figure 44. Cauliflower and hypertension, dose-response analysis per 100 g/d

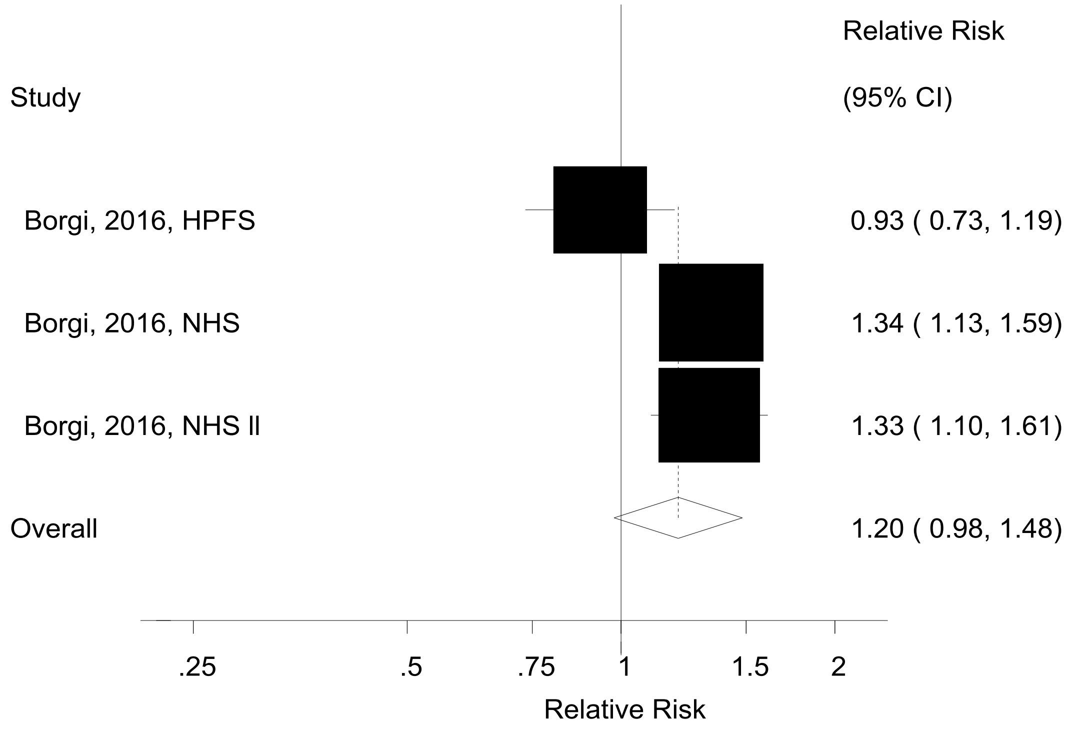

Supplementary Figure 45. Cauliflower and hypertension, nonlinear dose-response analysis

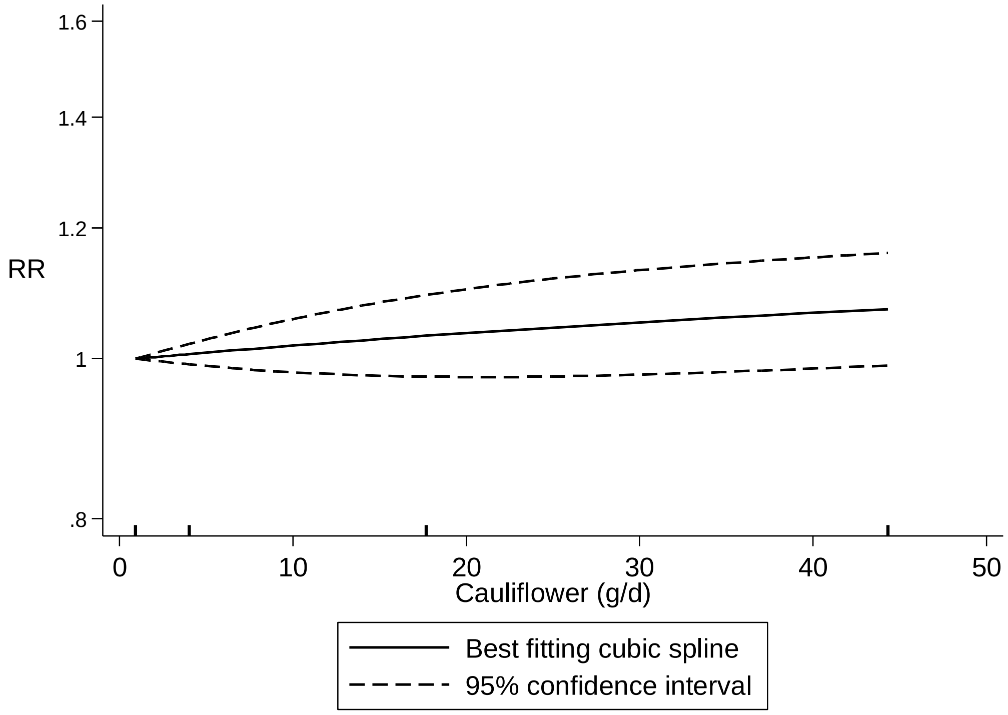

Supplementary Figure 46. Corn and hypertension, high vs. low

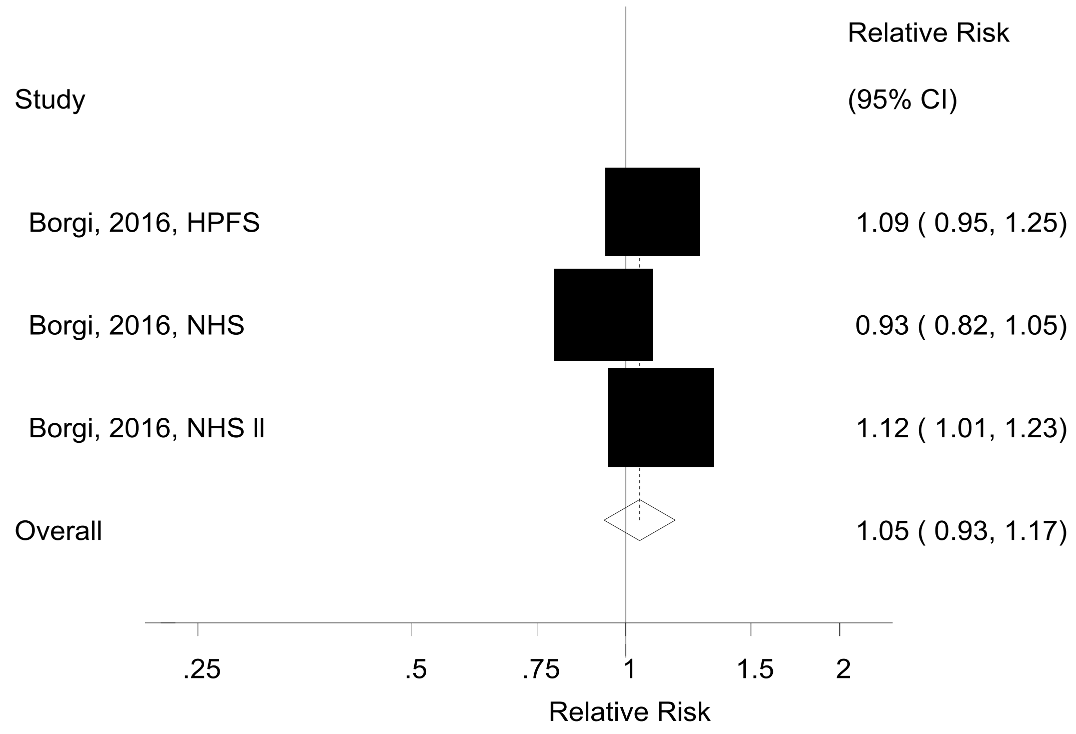

Supplementary Figure 47. Corn and hypertension, dose-response analysis per 100 g/d

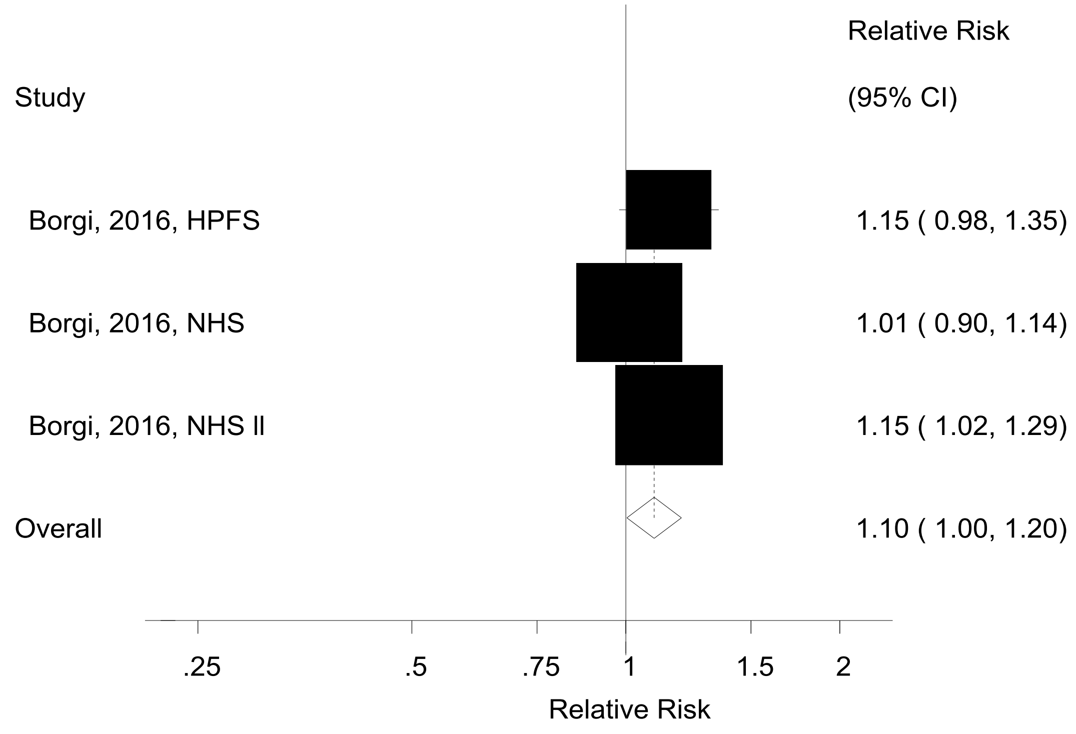

Supplementary Figure 48. Corn and hypertension, nonlinear dose-response analysis

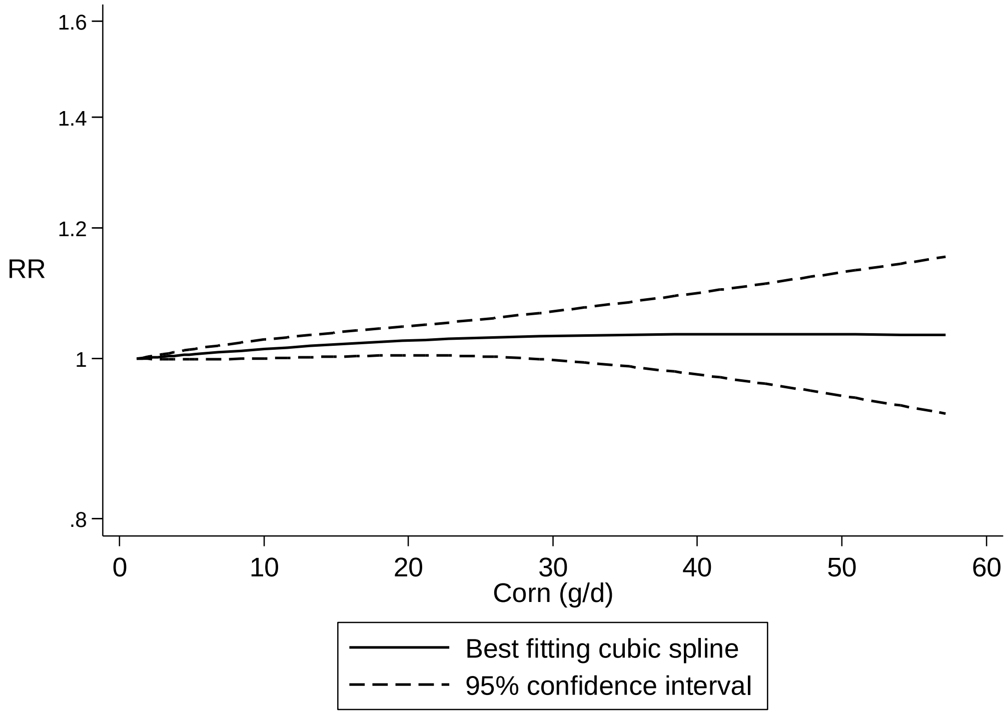

Supplementary Figure 49. Cruciferous vegetables and hypertension, high vs. low

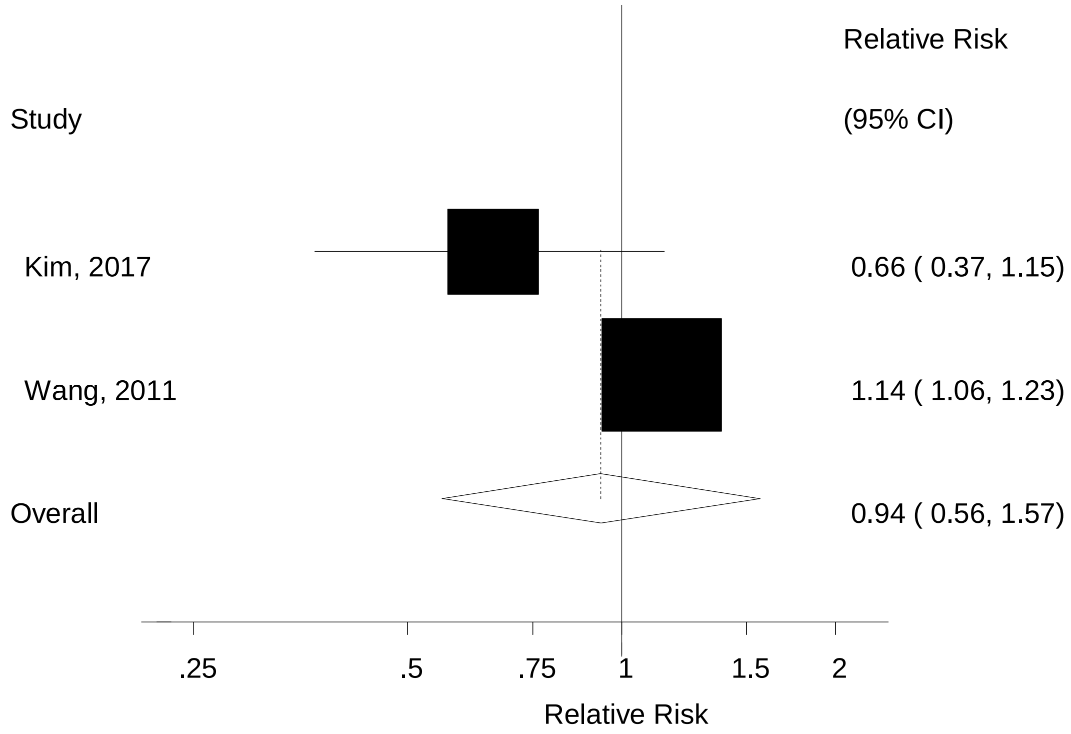

Supplementary Figure 50. Cruciferous vegetables and hypertension, dose-response analysis per 100 g/d

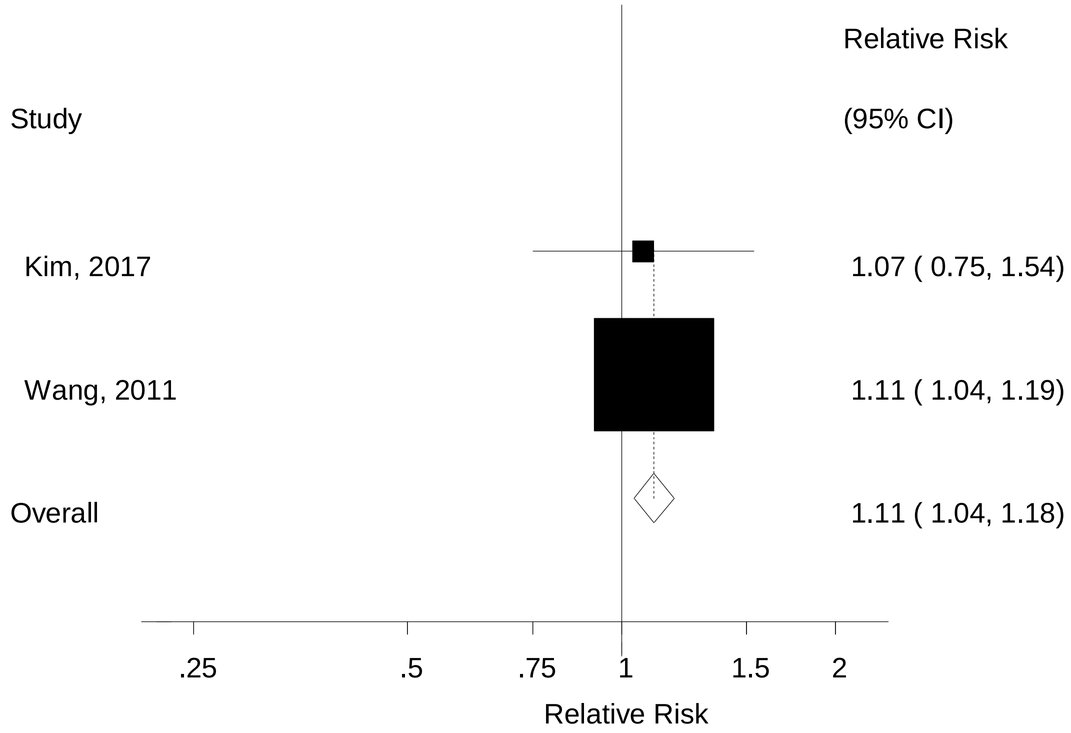

Supplementary Figure 51. Cruciferous vegetables and hypertension, nonlinear dose-response analysis

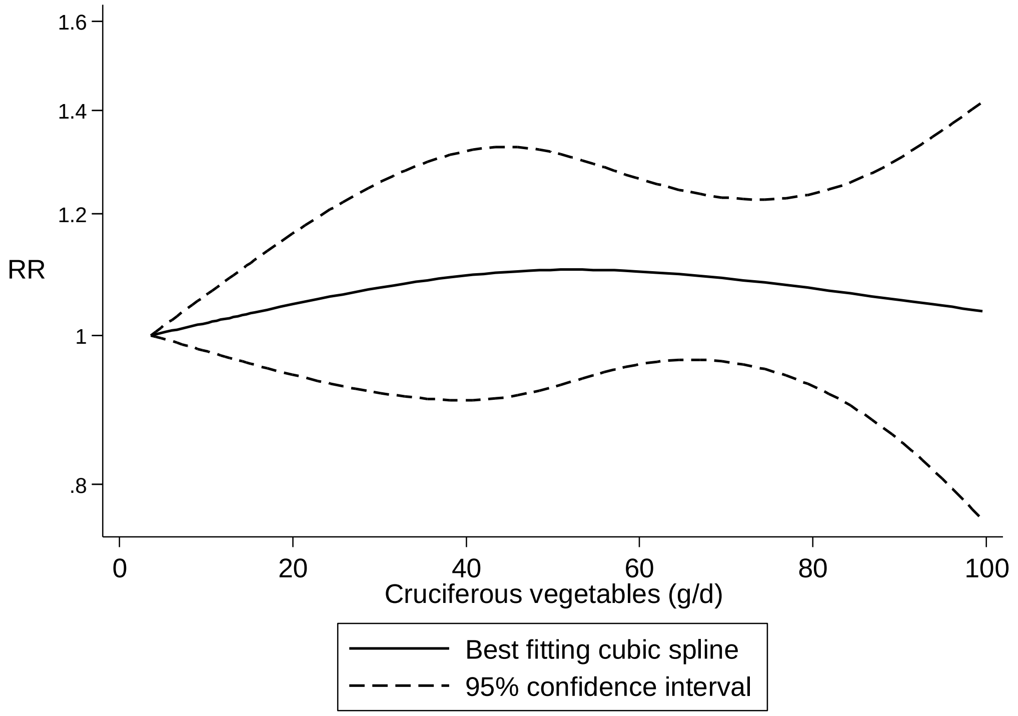

Supplementary Figure 52. Green leafy vegetables and hypertension, high vs. low

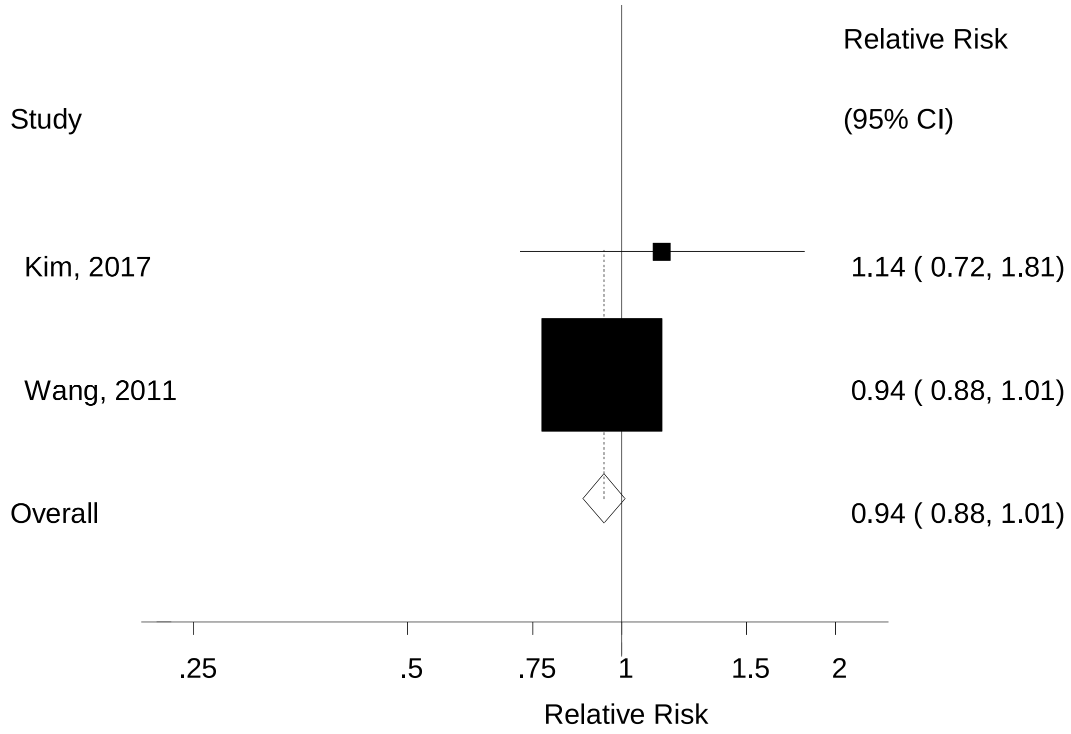

Supplementary Figure 53. Green leafy vegetables and hypertension, dose-response analysis per 100 g/d

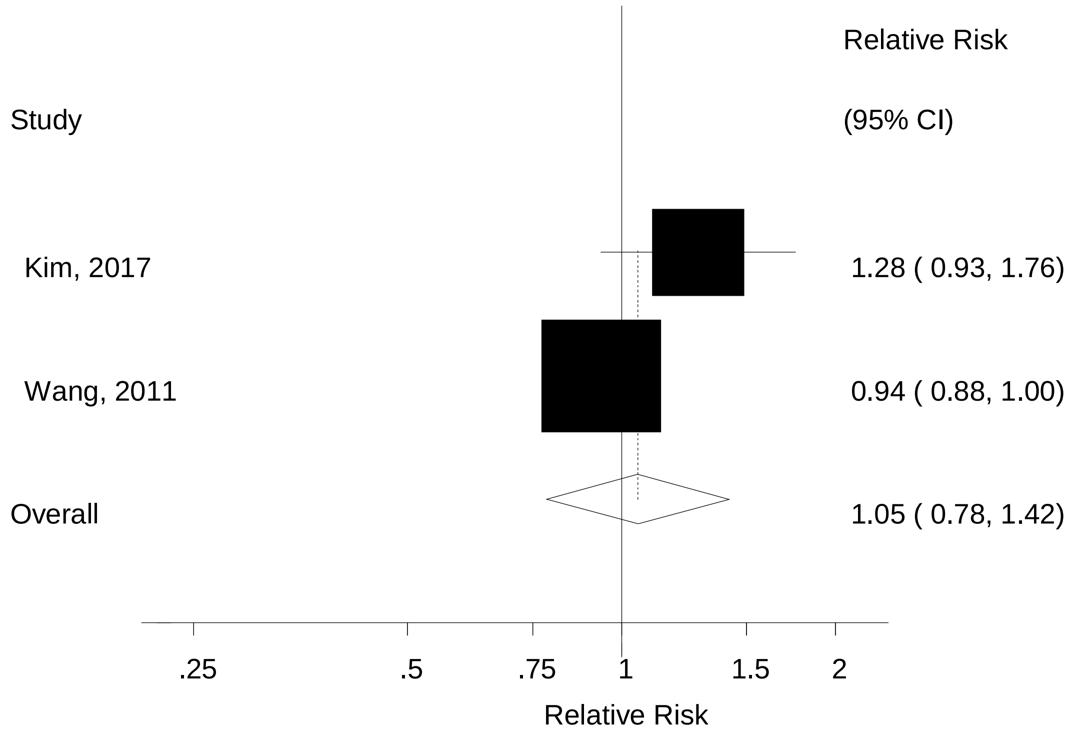

Supplementary Figure 54. Green leafy vegetables and hypertension, nonlinear dose-response analysis

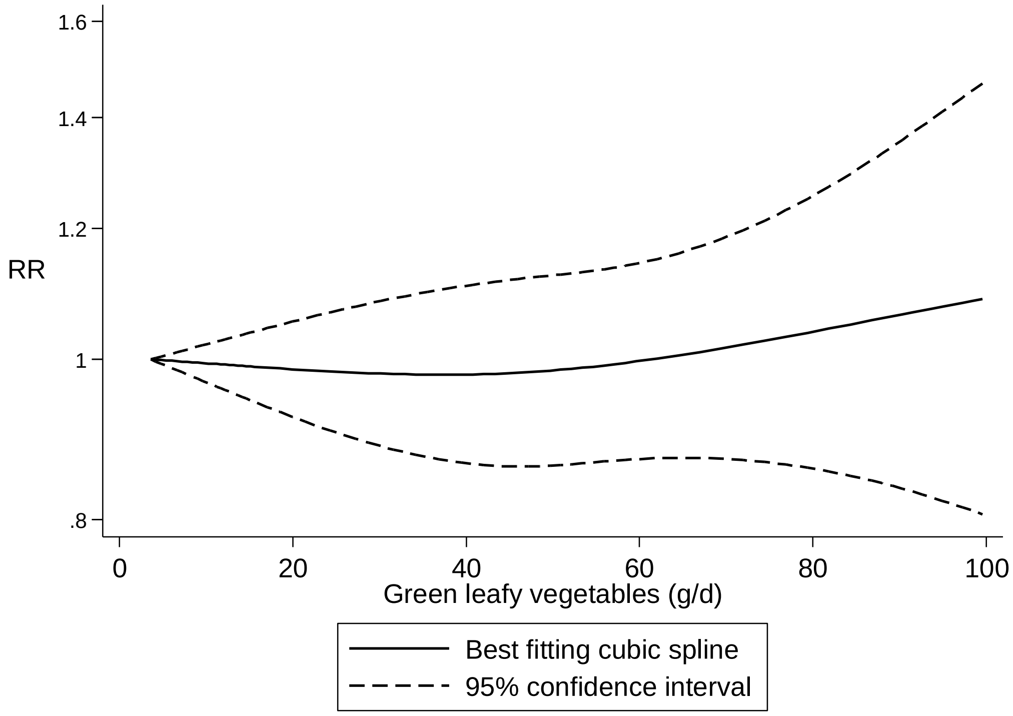

Supplementary Figure 55. Green pepper and hypertension, high vs. low

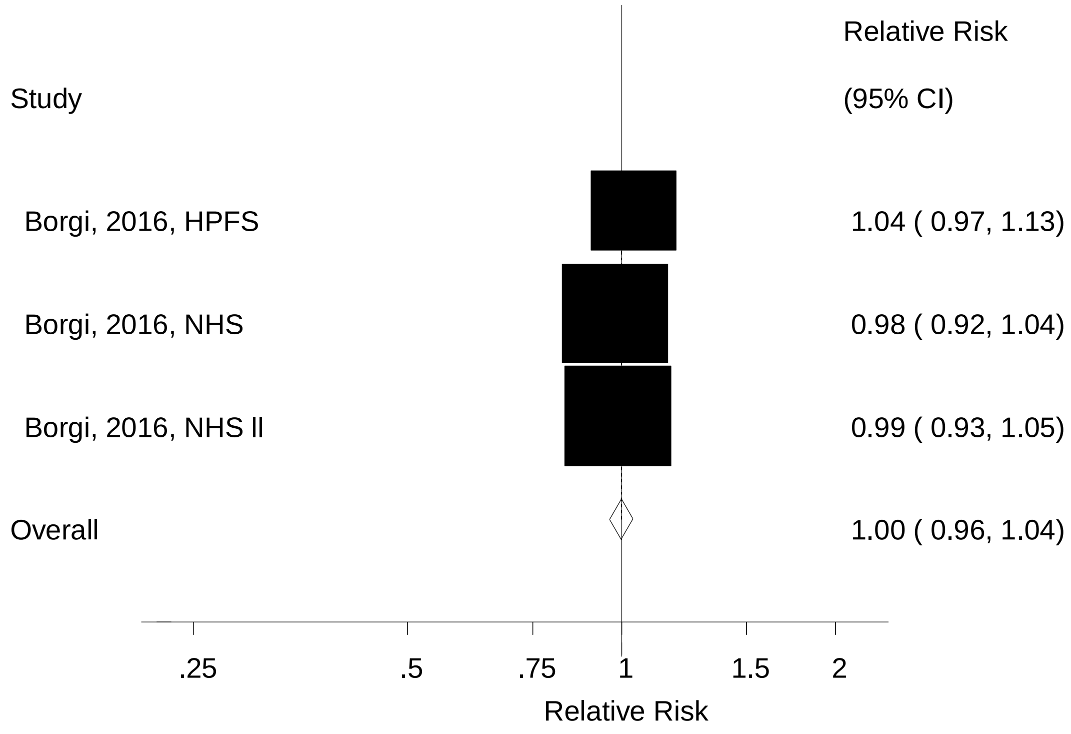

Supplementary Figure 56. Green pepper and hypertension, dose-response analysis per 100 g/d

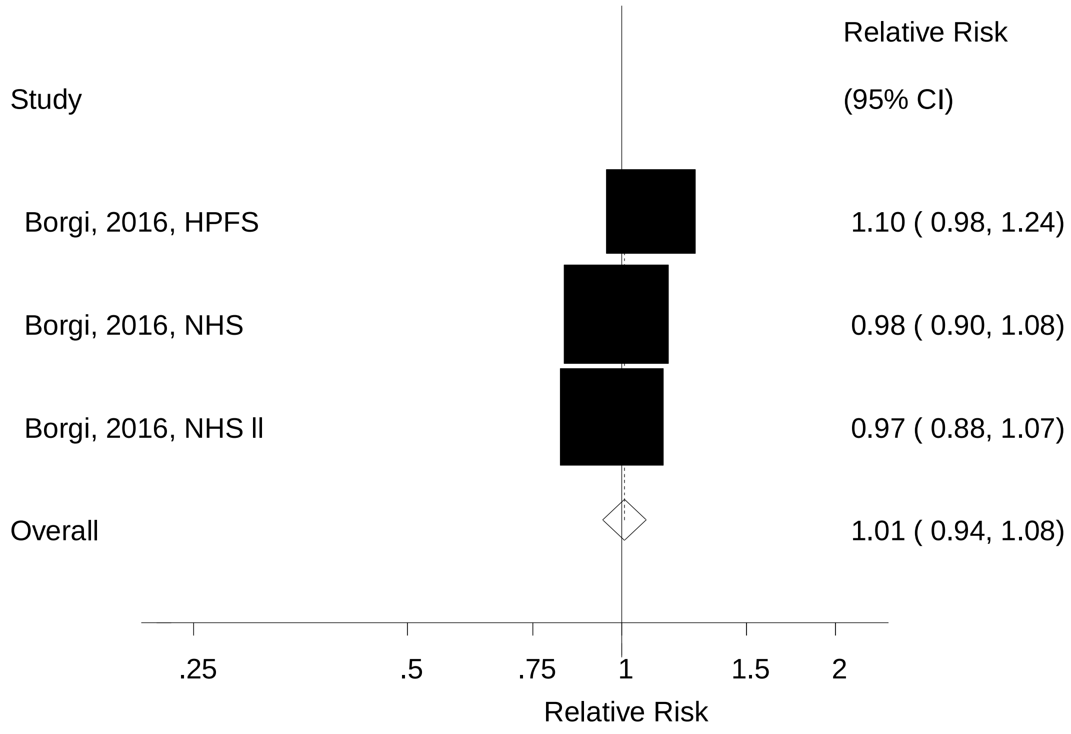

Supplementary Figure 57. Green pepper and hypertension, nonlinear dose-response analysis

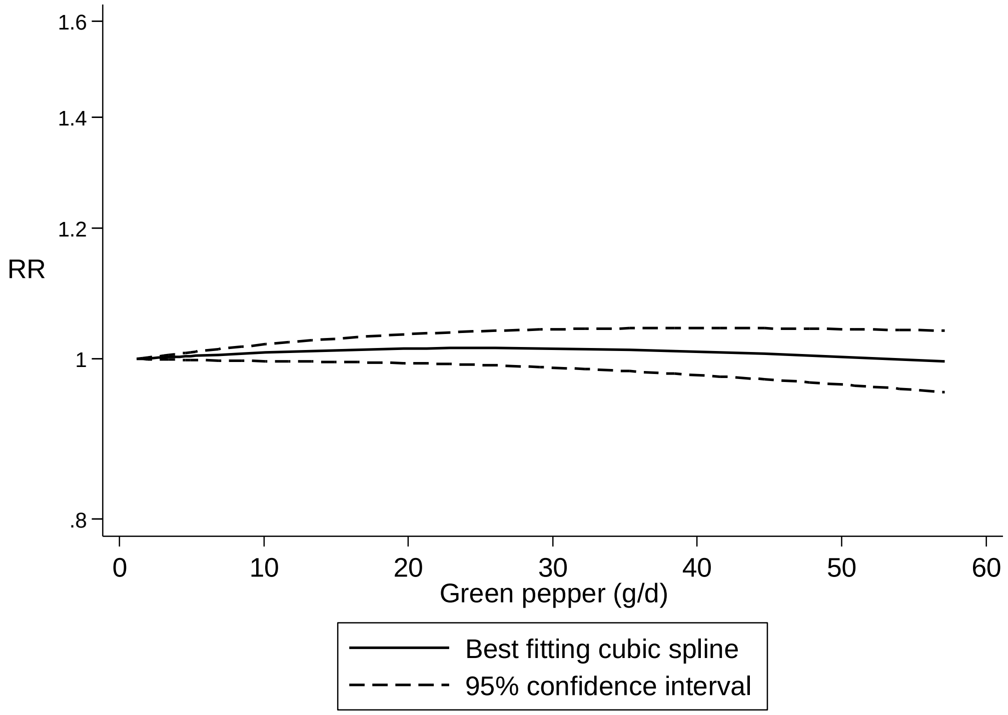

Supplementary Figure 58. Lettuce and hypertension, high vs. low

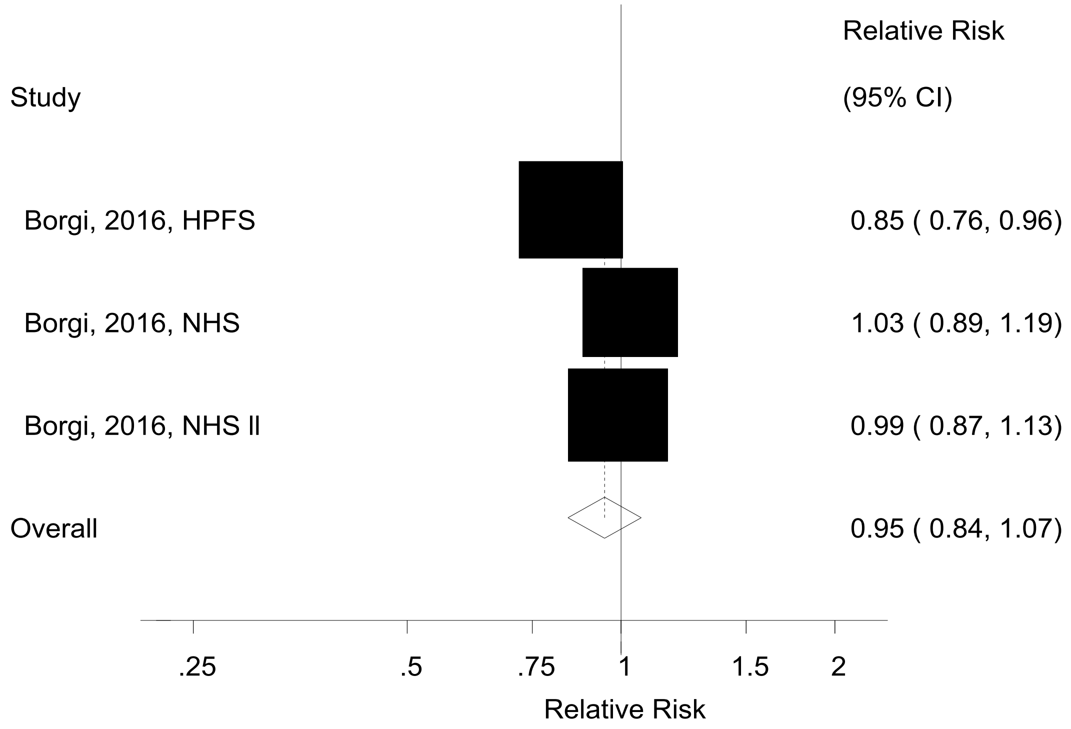

Supplementary Figure 59. Lettuce and hypertension, dose-response analysis per 100 g/d

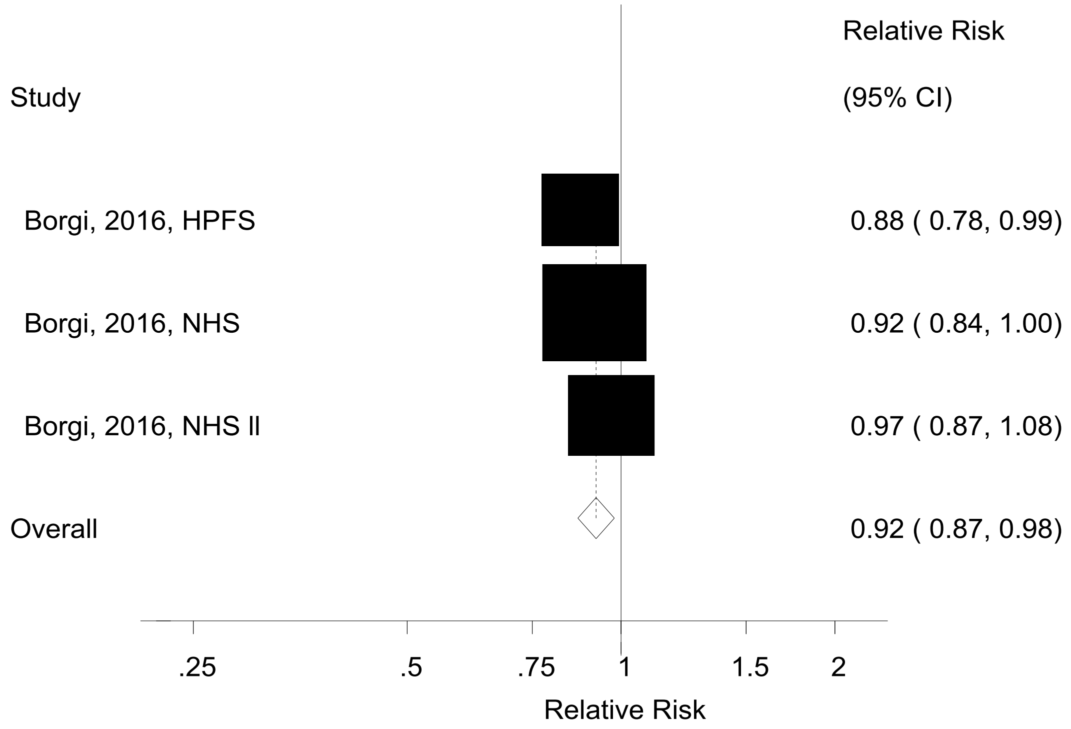

Supplementary Figure 60. Lettuce and hypertension, nonlinear dose-response analysis

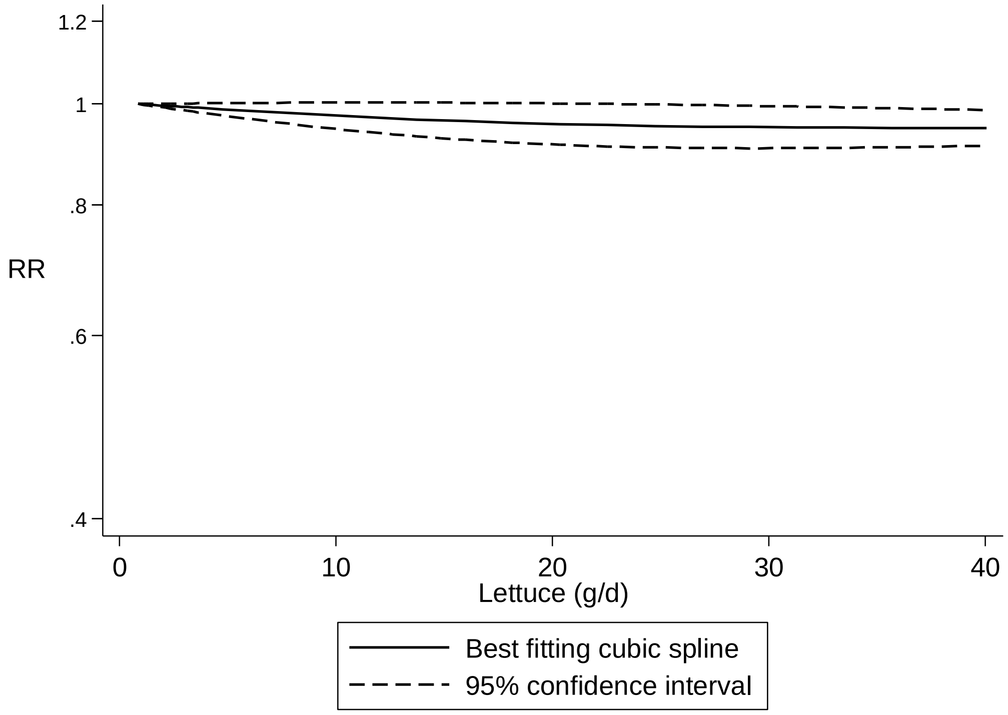

Supplementary Figure 61. Onions and hypertension, high vs. low

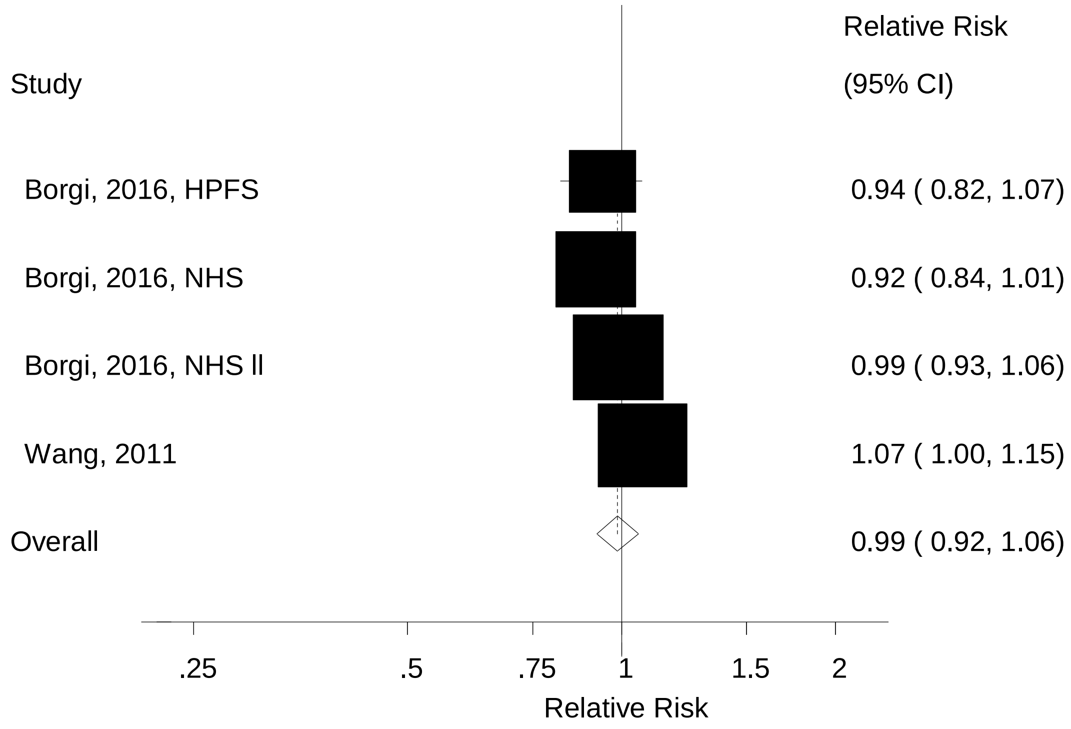

Supplementary Figure 62. Onions and hypertension, dose-response per 100 g/d

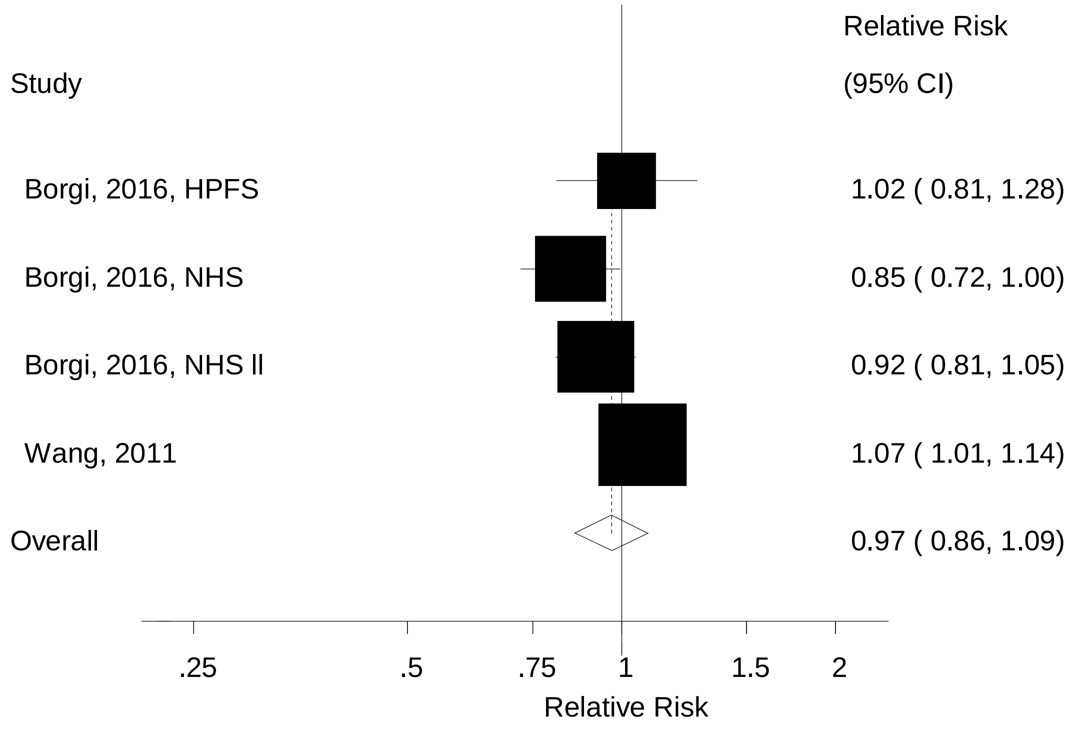

Supplementary Figure 63. Onions and hypertension, nonlinear dose-response analysis

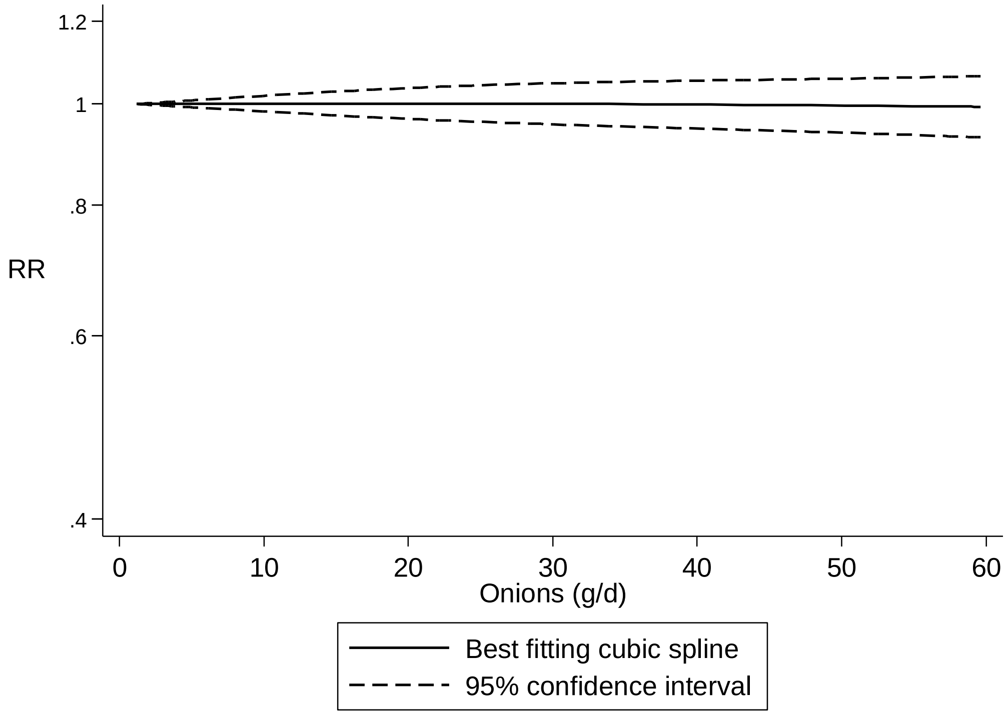

Supplementary Figure 64. Potatoes (total) and hypertension, high vs. low

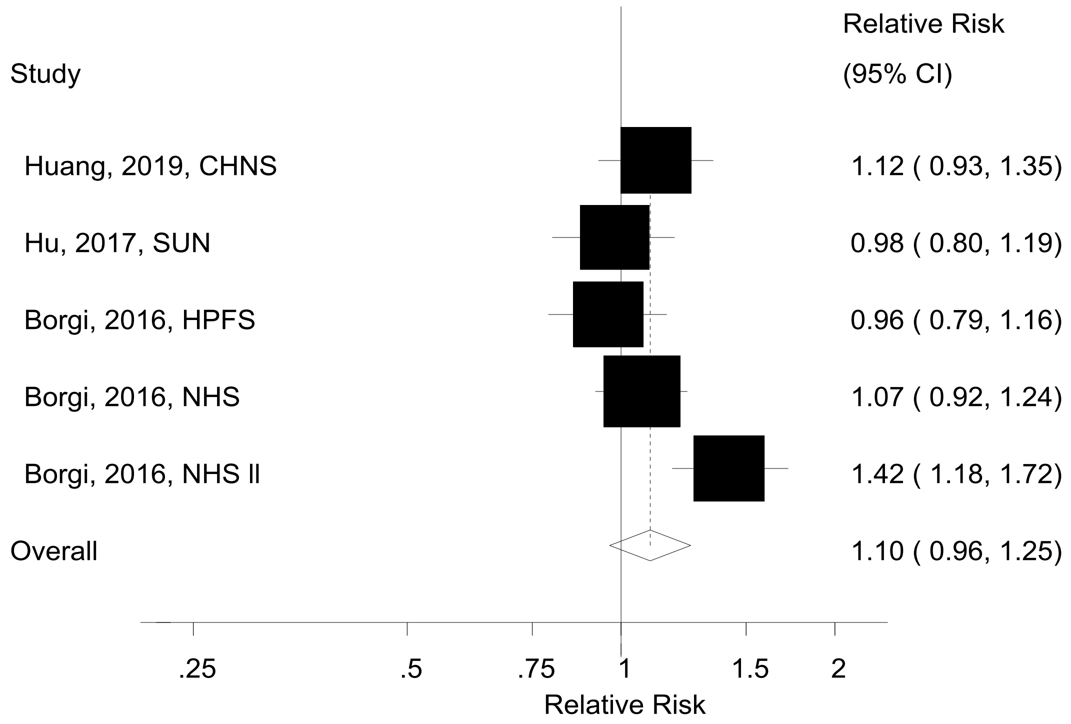

Supplementary Figure 65. Potatoes (total) and hypertension, dose-response per 100 g/d

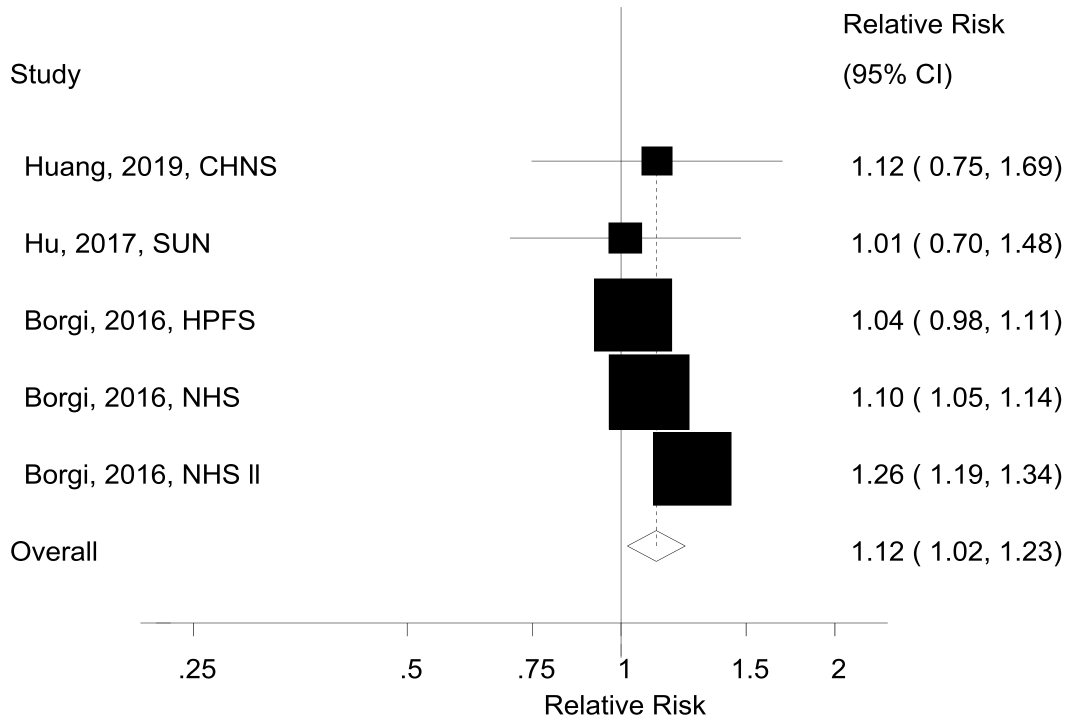

Supplementary Figure 66. Potatoes (total) and hypertension, nonlinear dose-response analysis

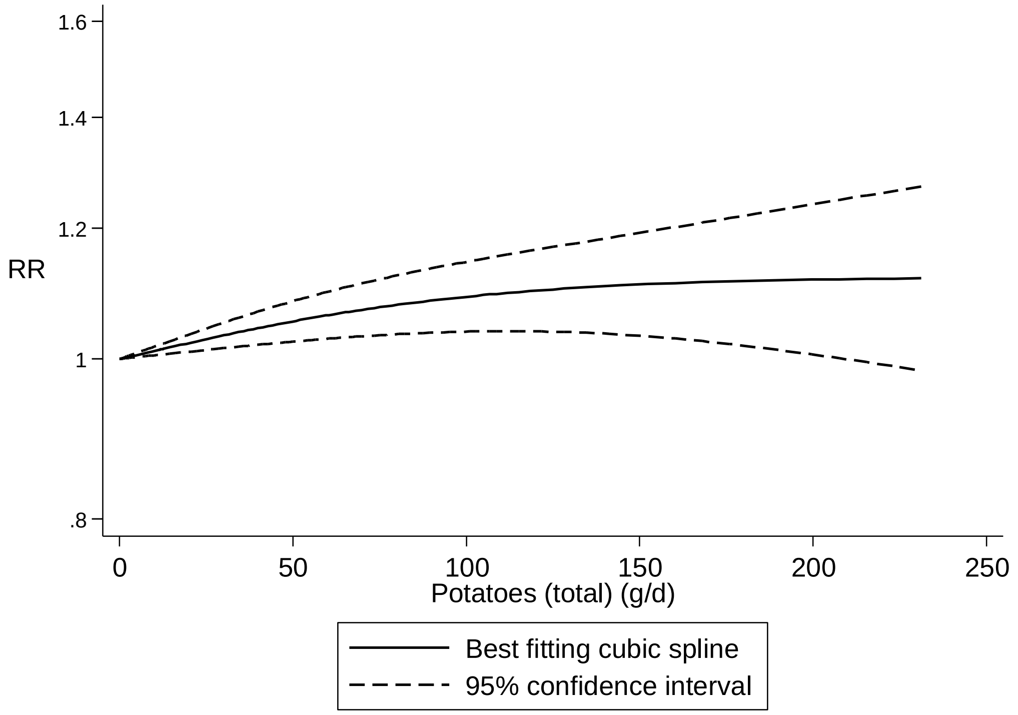

Supplementary Figure 67. Potatoes (fried) and hypertension, high vs. low

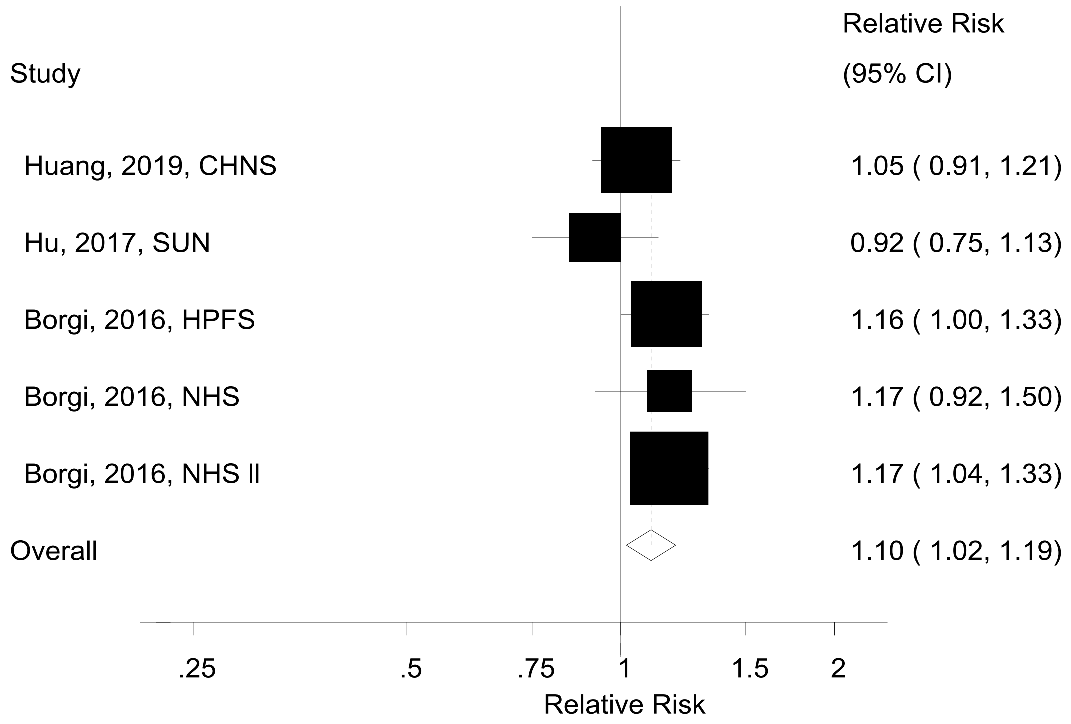

Supplementary Figure 68. Potatoes (fried) and hypertension, dose-response per 100 g/d

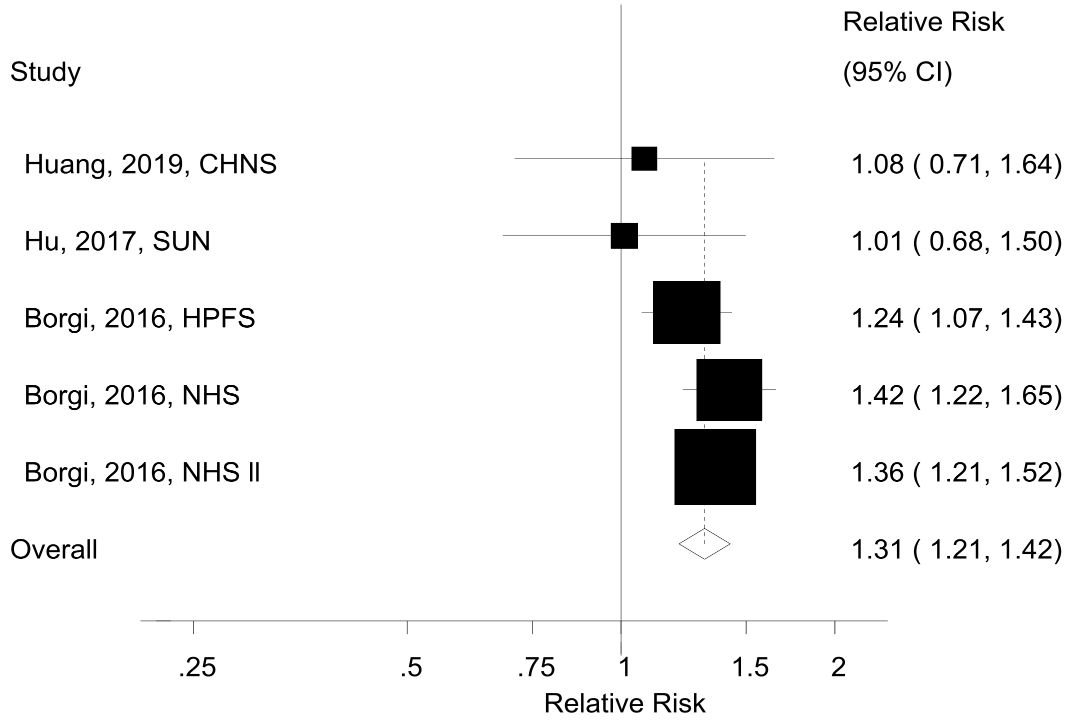

Supplementary Figure 69. Potatoes (fried) and hypertension, nonlinear dose-response analysis

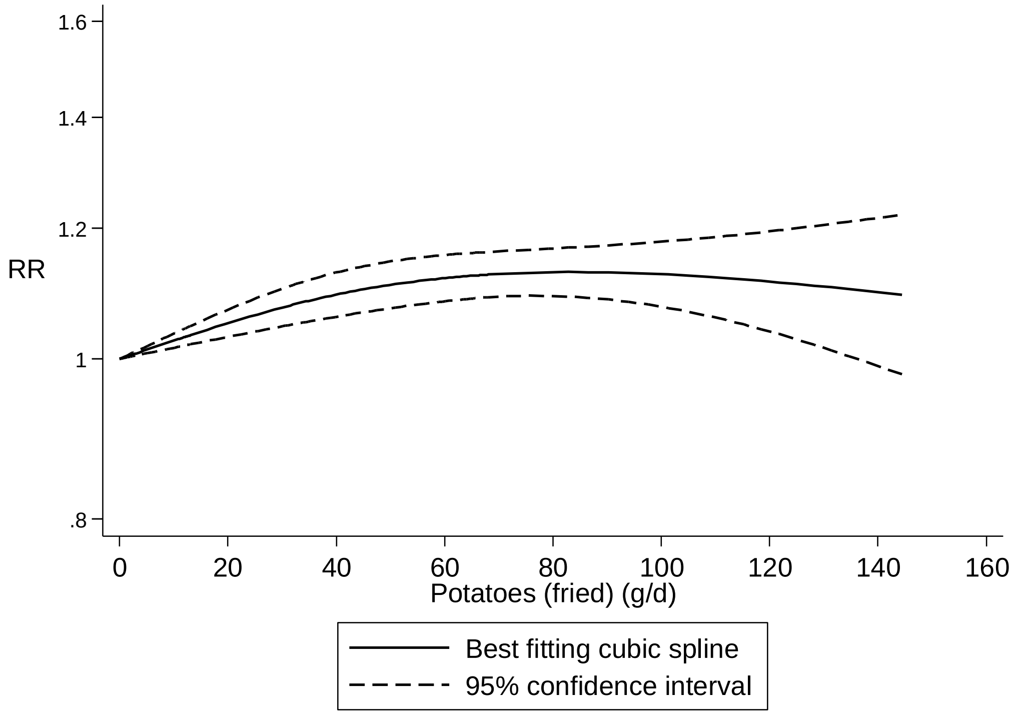

Supplementary Figure 70. Potatoes (non-fried) and hypertension, high vs. low

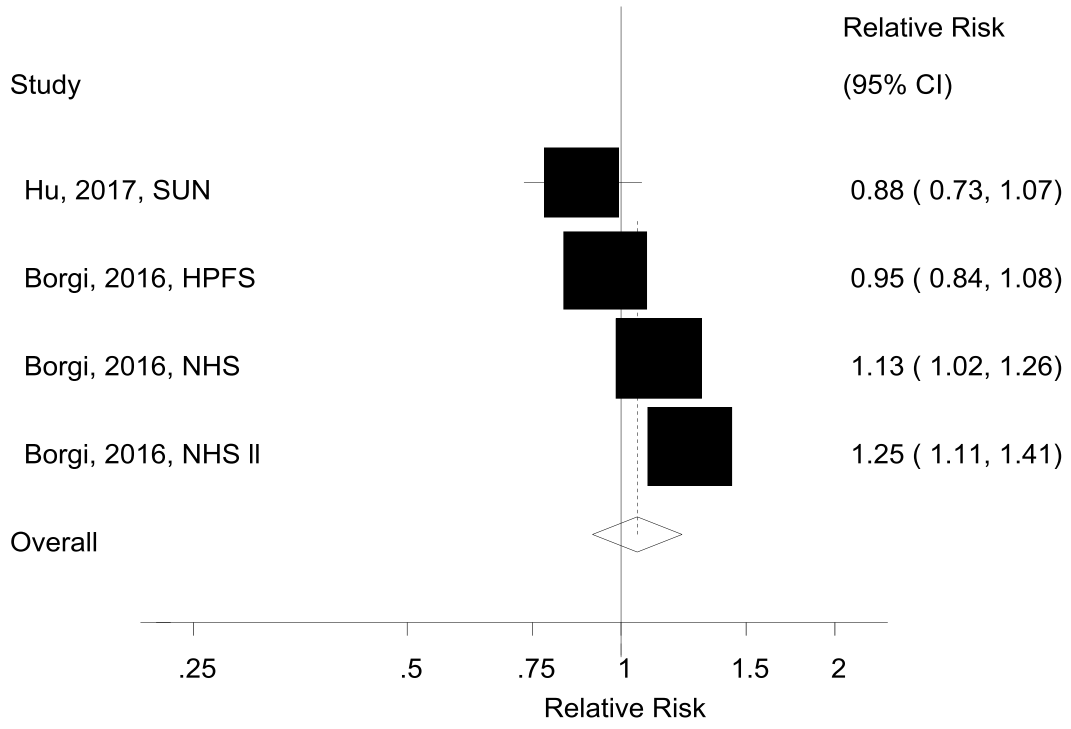

Supplementary Figure 71. Potatoes (non-fried) and hypertension, dose-response per 100 g/d

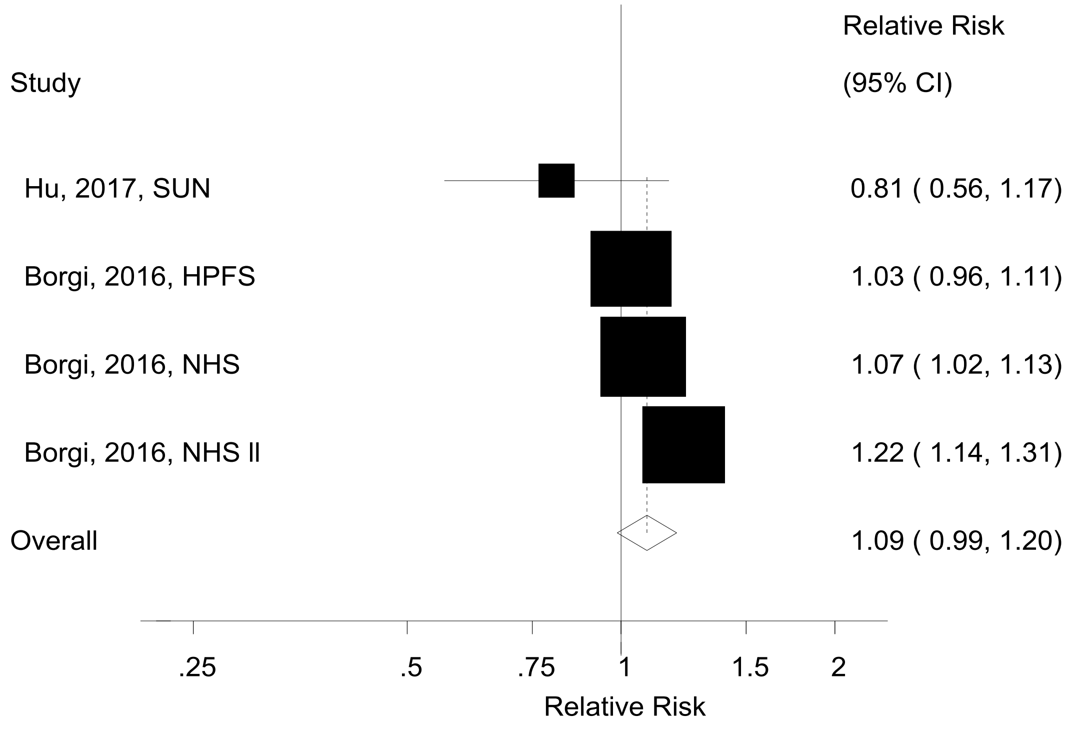

Supplementary Figure 72. Potatoes (non-fried) and hypertension, nonlinear dose-response analysis

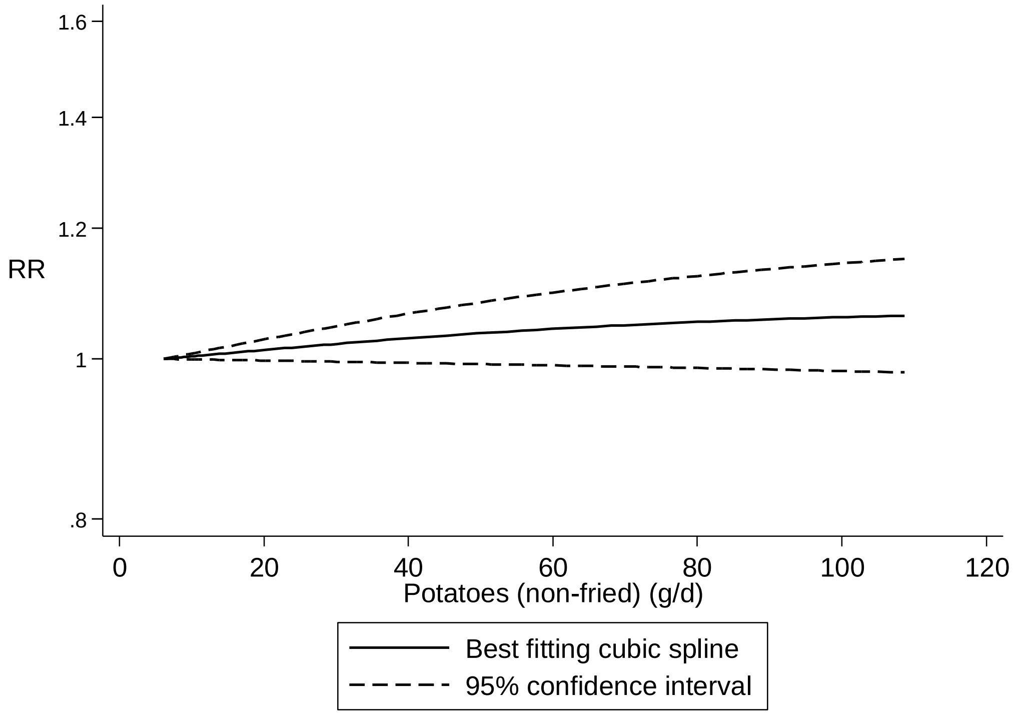

Supplementary Figure 73. Tomatoes and hypertension, high vs. low

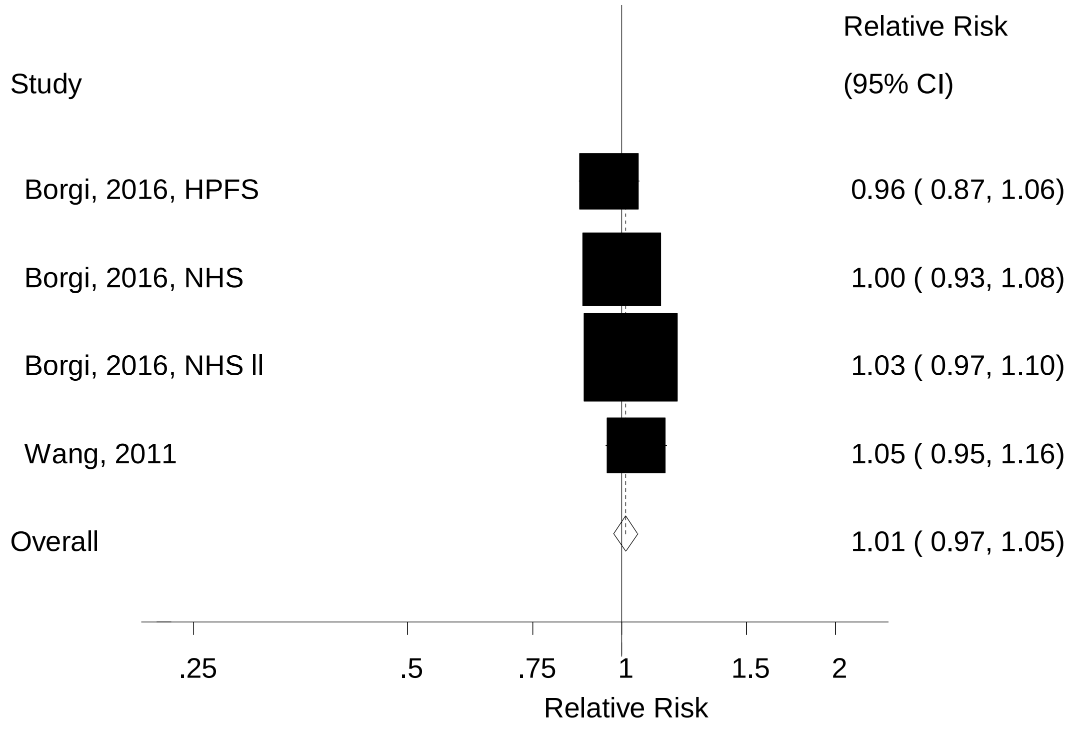

Supplementary Figure 74. Tomatoes and hypertension, dose-response per 100 g/d

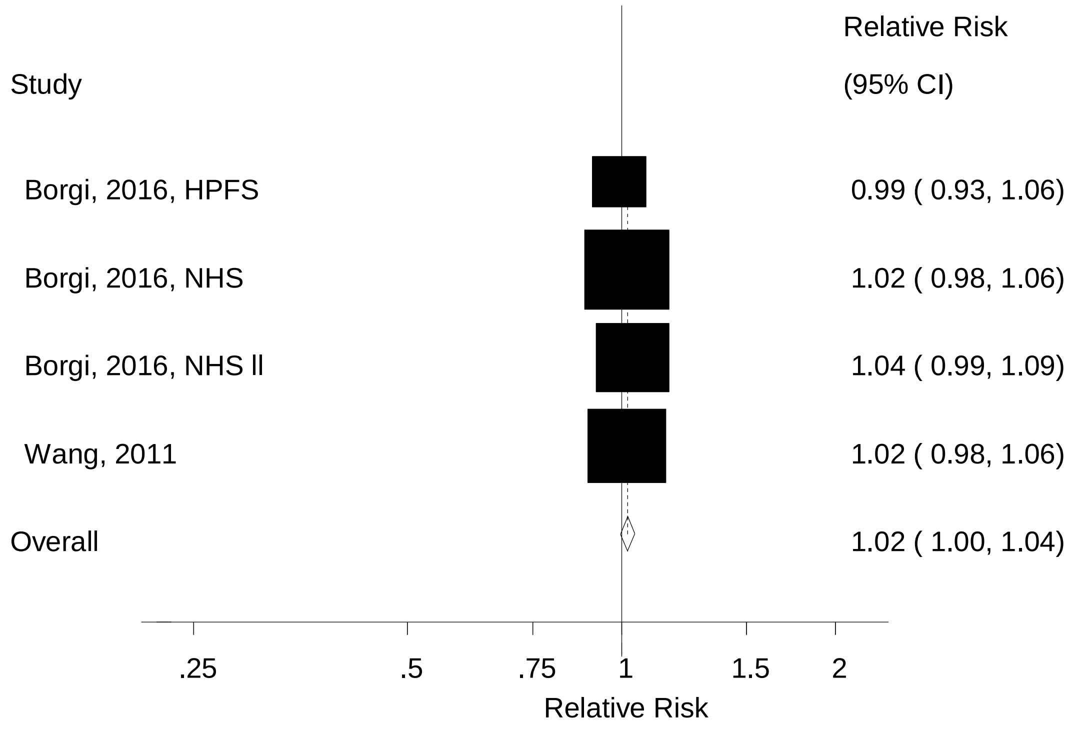

Supplementary Figure 75. Tomatoes and hypertension, nonlinear dose-response analysis

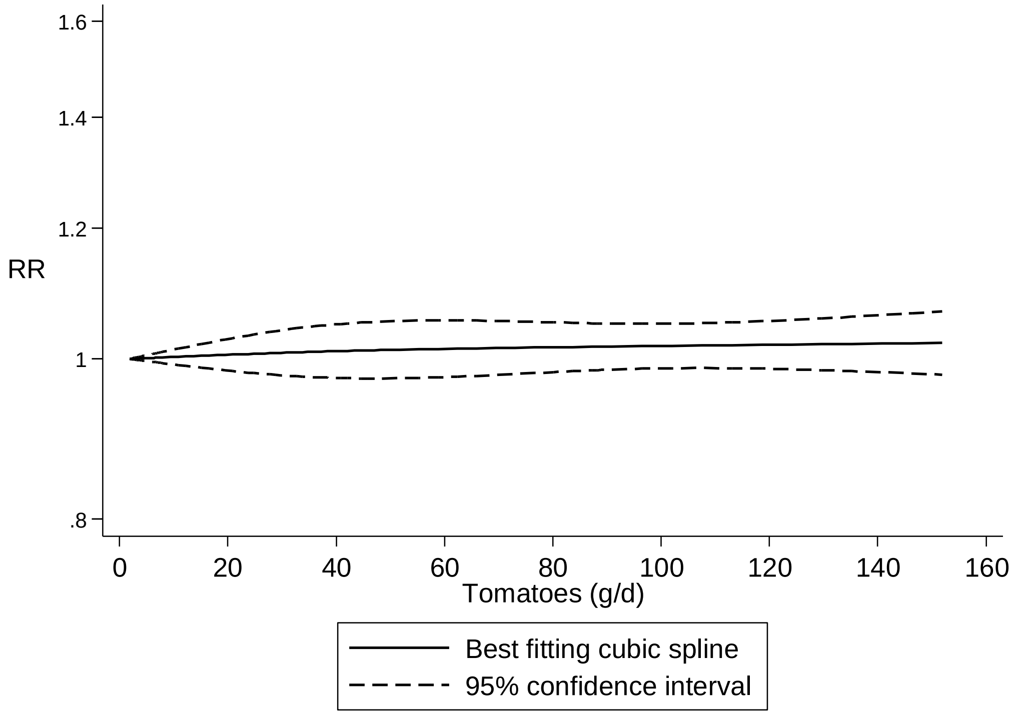

Supplementary Figure 76. Yams or sweet potatoes and hypertension, high vs. low

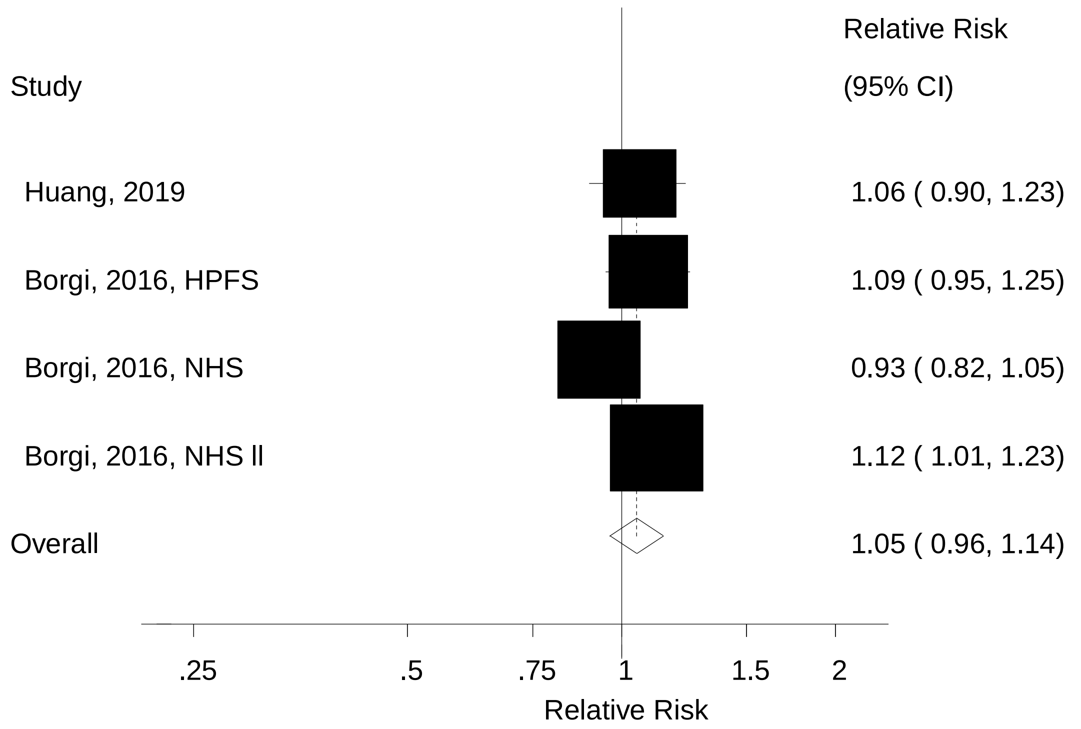

Supplementary Figure 77. Yams or sweet potatoes and hypertension, dose-response per 100 g/d

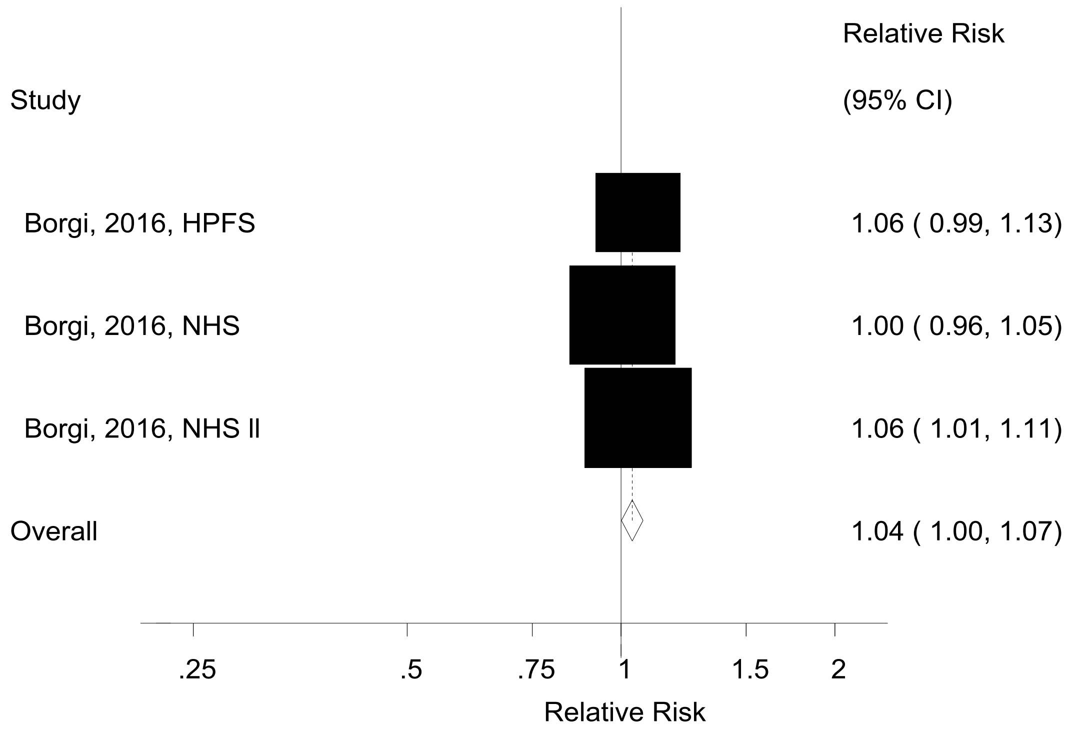

Supplementary Figure 78. Yams or sweet potatoes and hypertension, nonlinear dose-response analysis

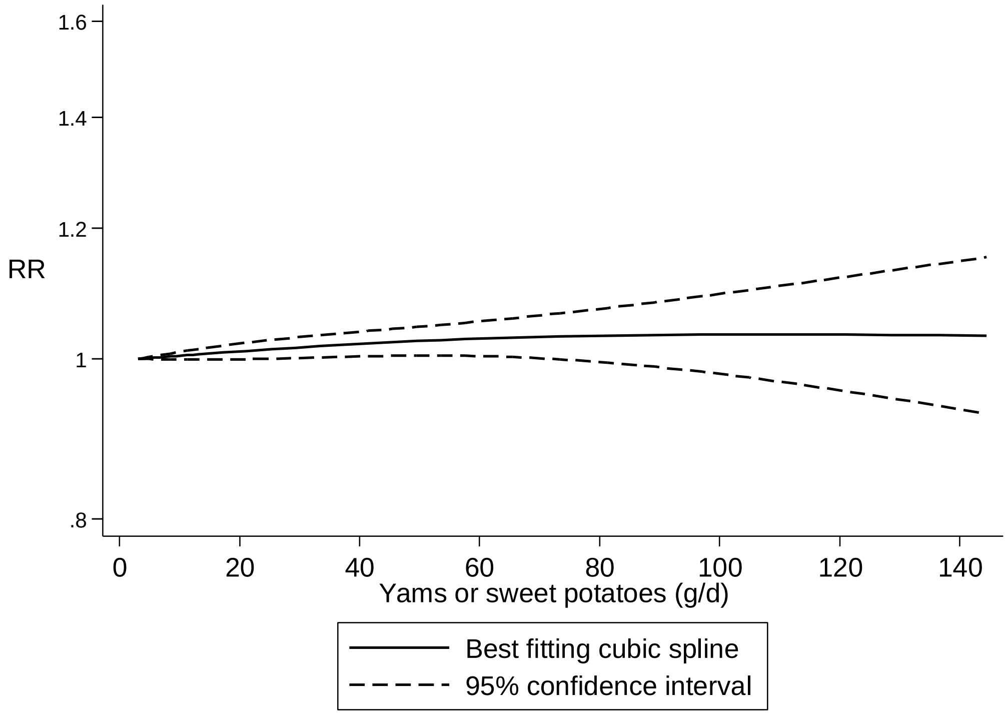

Funnel plots

Supplementary Figure 79. Funnel plot of fruits, vegetables and hypertension

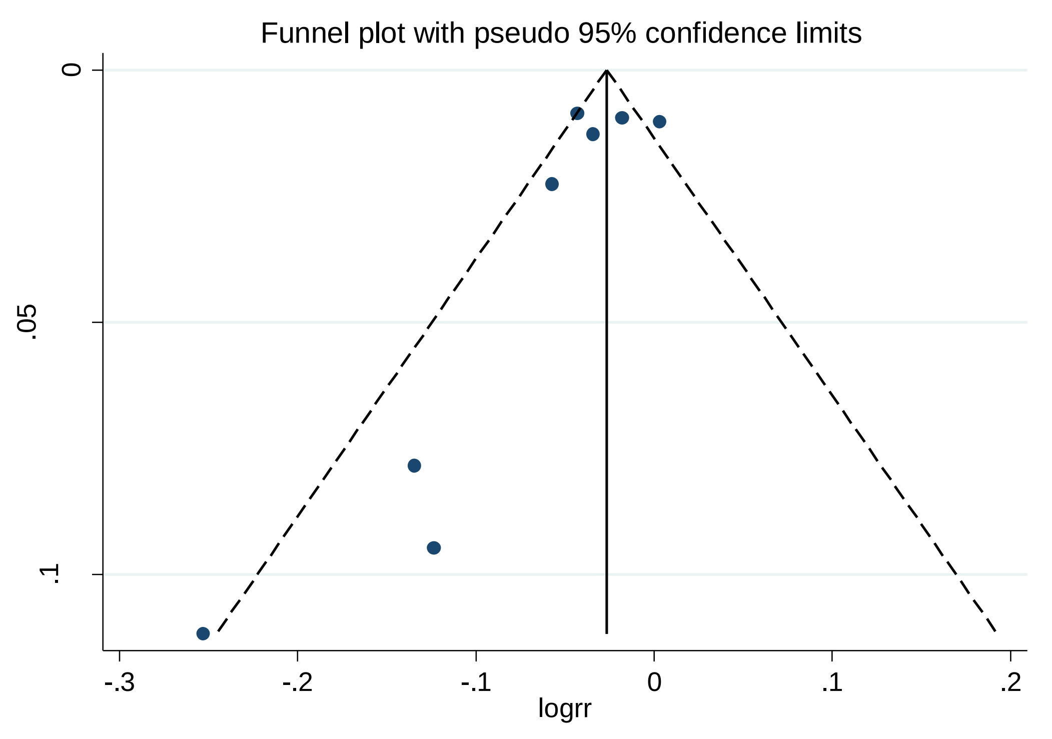

Supplementary Figure 80. Funnel plot of fruit and hypertension

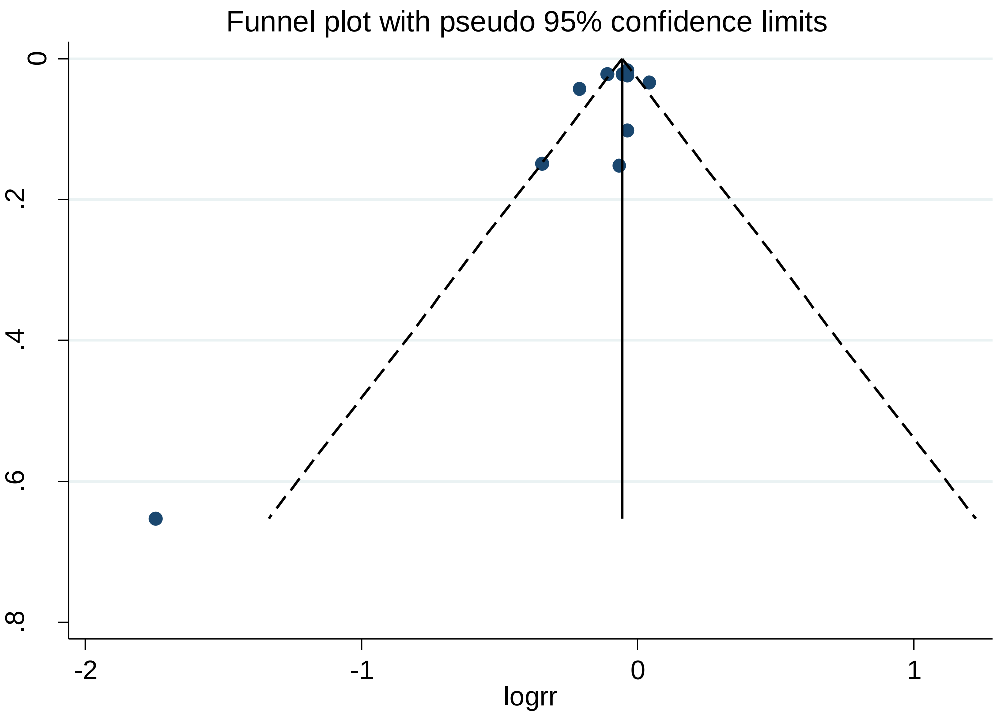

Supplementary Figure 81. Funnel plot of vegetables and hypertension

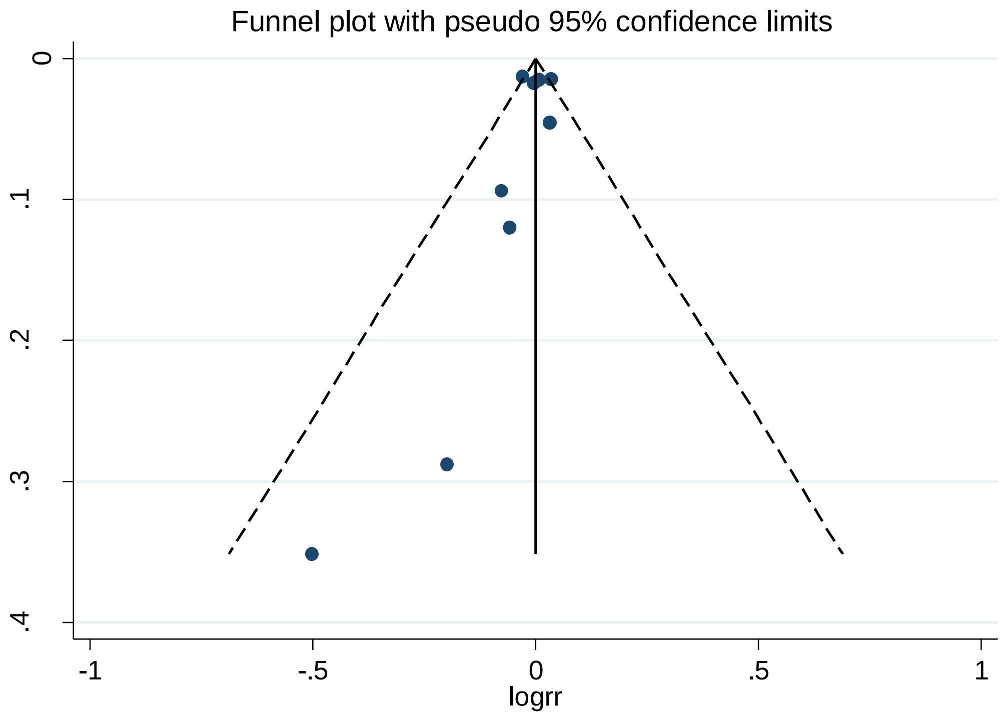

Sensitivity analyses

Supplementary Figure 82. Sensitivity analysis of fruits, vegetables and hypertension

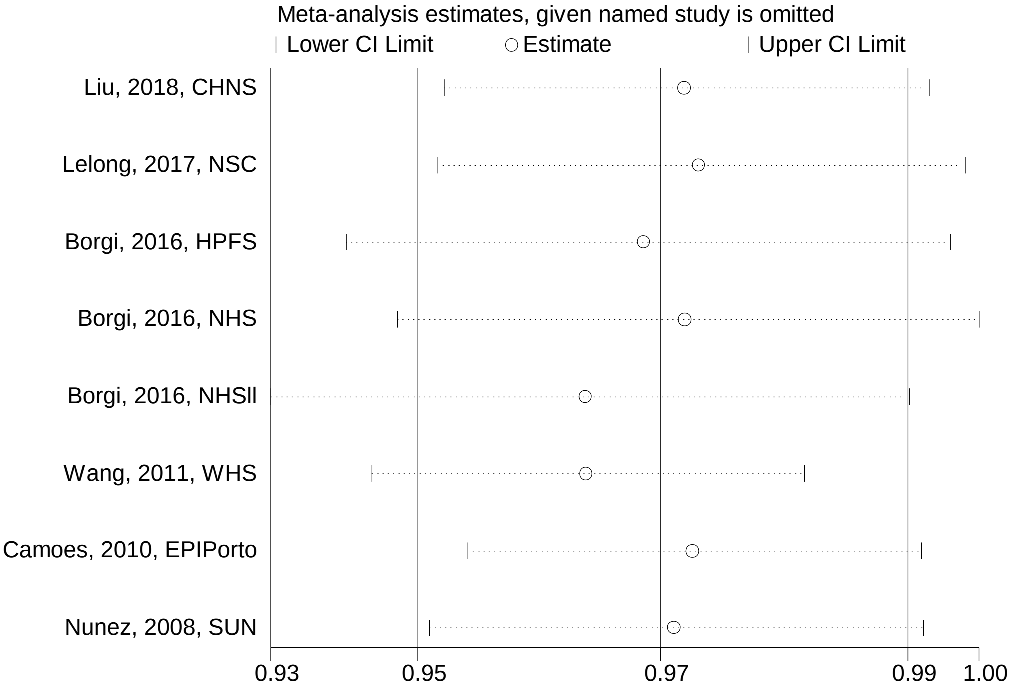

Study omitted	Estimate	[95% Conf.	Interval]
Liu, 2018, CHNS	0.96992177	0.94951165	0.99077058
Lelong, 2017, NSC	0.97115278	0.94896632	0.99385792
Borgi, 2016, HPFS	0.96650964	0.94116724	0.9925344
Borgi, 2016, NHS	0.96995783	0.94553989	0.99500638
Borgi, 2016, NHSll	0.96155089	0.93478274	0.98908556
Wang, 2011, WHS	0.96158999	0.94337302	0.98015875
Camoes, 2010, EPIPorto	0.97062004	0.95152235	0.99010104
Nunez, 2008, SUN	0.96903634	0.94826919	0.99025822
Combined	0.96790122	0.94729542	0.98895523

Supplementary Figure 83. Sensitivity analysis of fruit and hypertension

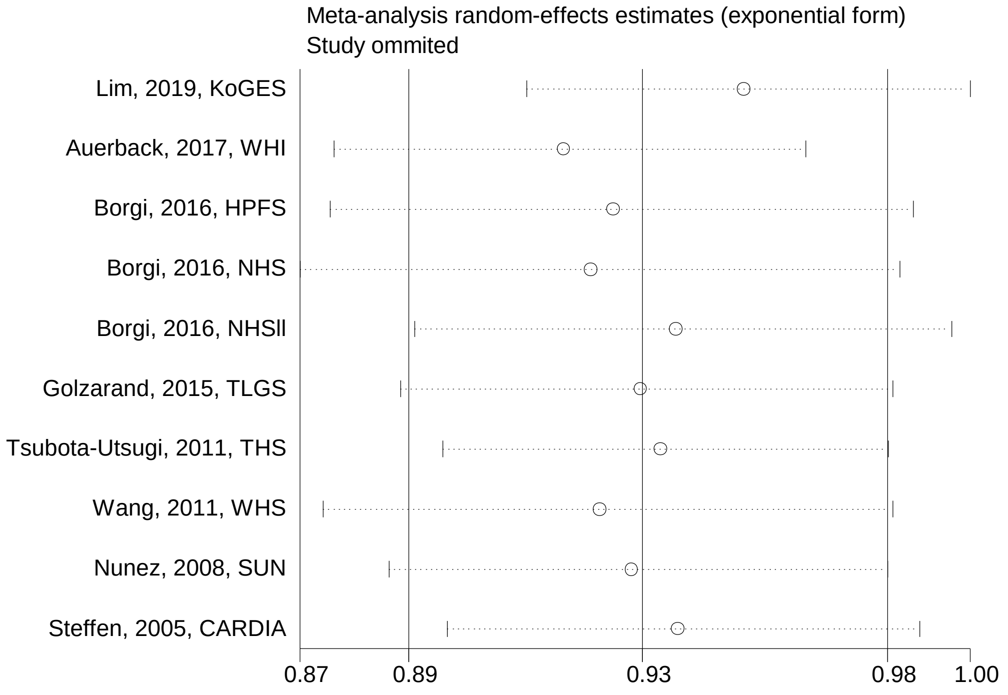

Study omitted	e^coef.	[95% Conf.	Interval]
Lim, 2019, KoGES	0.95135158	0.90953809	0.99508739
Auerback, 2017, WHI	0.91671884	0.87240541	0.96328318
Borgi, 2016, HPFS	0.92618054	0.87163538	0.98413903
Borgi, 2016, NHS	0.9218424	0.86578923	0.98152459
Borgi, 2016, NHSll	0.93827534	0.88796544	0.99143565
Golzarand, 2015, TLGS	0.93142581	0.88514787	0.98012334
Tsubota-Utsugi, 2011, THS	0.9353146	0.89336491	0.97923416
Wang, 2011, WHS	0.92353719	0.8702516	0.98008543
Nunez, 2008, SUN	0.92977113	0.88293815	0.97908825
Steffen, 2005, CARDIA	0.93867707	0.89423448	0.98532844
Combined	0.93182693	0.88674098	0.97920525

Supplementary Figure 84. Sensitivity analysis of vegetables and hypertension

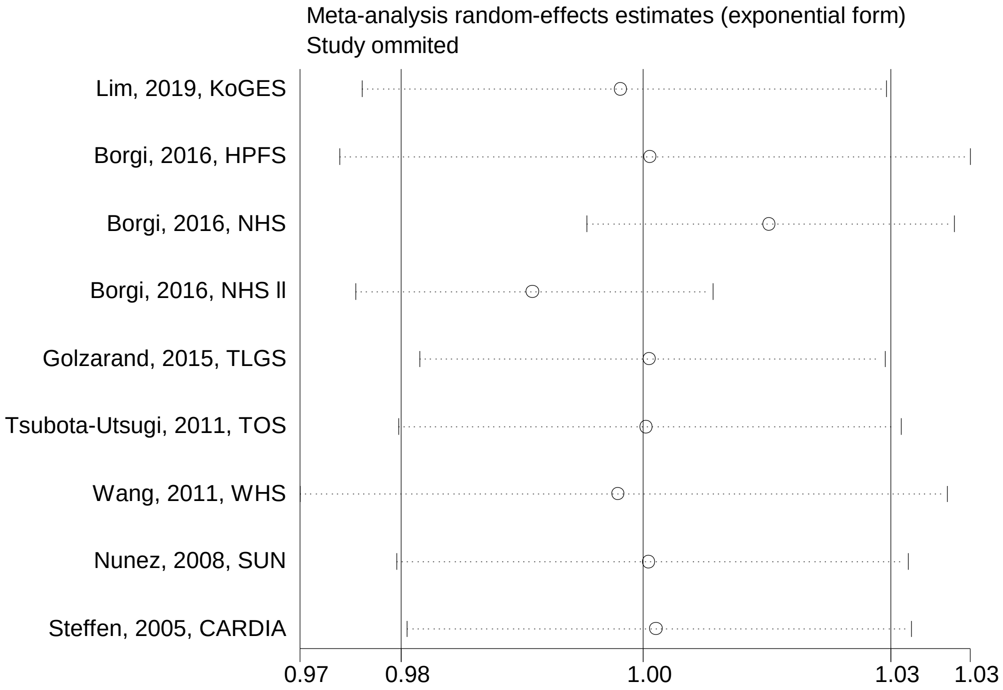

Study omitted	e^coef.	[95% Conf.	Interval]
Lim, 2019, KoGES	0.99886721	0.97290188	1.0255255
Borgi, 2016, HPFS	1.0018164	0.97067493	1.033957
Borgi, 2016, NHS	1.0137668	0.99549472	1.0323743
Borgi, 2016, NHS ll	0.99002546	0.97227407	1.008101
Golzarand, 2015, TLGS	1.0017784	0.97870123	1.0253997
Tsubota-Utsugi, 2011, TOS	1.0014621	0.97655869	1.0270005
Wang, 2011, WHS	0.99862725	0.96668977	1.0316199
Nunez, 2008, SUN	1.0017072	0.9763726	1.0276992
Steffen, 2005, CARDIA	1.0024327	0.97743982	1.0280647
Combined	1.0011179	0.97684287	1.0259962
